# Exploring the Potential of Nonclassical Crystallization Pathways to Advance Cementitious Materials

**DOI:** 10.1021/acs.chemrev.3c00259

**Published:** 2024-06-14

**Authors:** Cristina Ruiz-Agudo, Helmut Cölfen

**Affiliations:** Physical Chemistry, Department of Chemistry, University of Konstanz, Universitätsstr. 10, 78457 Konstanz, Germany

## Abstract

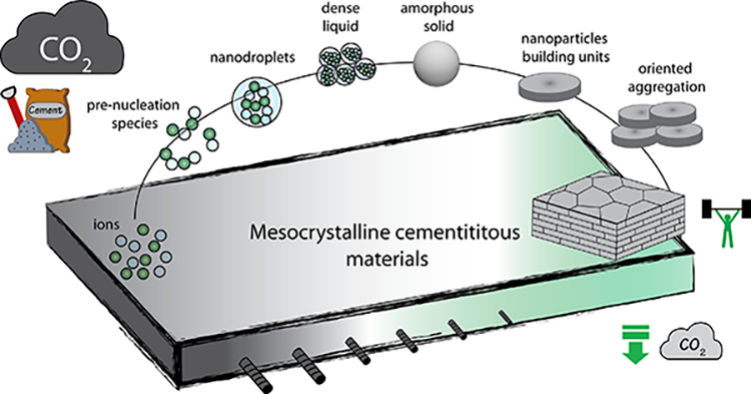

Understanding the crystallization of cement-binding phases, from basic units to macroscopic structures, can enhance cement performance, reduce clinker use, and lower CO_2_ emissions in the construction sector. This review examines the crystallization pathways of C–S–H (the main phase in PC cement) and other alternative binding phases, particularly as cement formulations evolve toward increasing SCMs and alternative binders as clinker replacements. We adopt a **nonclassical crystallization** perspective, which recognizes the existence of critical intermediate steps between ions in solution and the final crystalline phases, such as solute ion associates, dense liquid phases, amorphous intermediates, and nanoparticles. These multistep pathways uncover innovative strategies for controlling the crystallization of binding phases through additive use, potentially leading to highly optimized cement matrices. An outstanding example of **additive-controlled crystallization** in cementitious materials is the synthetically produced mesocrystalline C–S–H, renowned for its remarkable flexural strength. This highly ordered microstructure, which intercalates soft matter between inorganic and brittle C–S–H, was obtained by controlling the assembly of individual C–S–H subunits. While large-scale production of cementitious materials by a bottom-up self-assembly method is not yet feasible, the fundamental insights into the crystallization mechanism of cement binding phases presented here provide a foundation for developing advanced cement-based materials.

## 1 Introduction

### 1.1 Climate Change and the Role of Cementitious Materials

The consequences of global warming and climate change have become dramatically apparent in recent years, emphasizing the need for urgent global action to reduce CO_2_ emissions. Specific industries, such as the construction sector, face unique challenges that require improvements or replacements while maintaining immense production volumes. Cement and concrete are primary materials at the core of the construction industry. Concrete is produced by mixing cement, aggregates of various sizes, sand, and water. Cement is the key component of concrete, binding the other ingredients together to produce this ubiquitous, stone-like material that has shaped our world in unprecedented ways. The cement reacts with water to precipitate hydration products that bind the sand and aggregates, resulting in this versatile construction material. The advantages in terms of moldability, mechanical properties, durability, cost, and global availability have made concrete the most consumed material worldwide in terms of volume.

Cementitious materials are literally the foundations of our modern society, providing simple solutions to housing and infrastructure needs. Even though construction technologies are progressing toward more resource efficiency, concrete and cement will remain indispensable building materials, and hence, the cement industry faces the difficult challenge of decarbonization while meeting the inevitable growing demand for this product. In fact, cement and concrete are environmentally friendly compared to other building materials due to their relatively low embodied energy^1^ and the ability to use locally available raw materials, reducing transportation needs and environmental impacts. However, due to the immense amount of cement and concrete produced annually, the cement industry is one of the largest contributors to anthropogenic CO_2_ emissions. In 2021, the International Energy Agency (IEA) estimated that the cement industry was responsible for emitting almost 7% of total anthropogenic carbon dioxide emissions.^2^ As global concrete (and cement) production increases in response to the world’s ever-growing population, CO_2_ emissions will inevitably continue to rise.

Total cement production in 2021 was approximately 4.30 Gt and is expected to increase to approximately 4.68 Gt by 2050. This led to the burden of about 2.53 Gt of CO_2_ emissions in 2021 due to the large volumes of cement and concrete produced.^2^ With the cement demand projected to grow by 12–23% by 2050,^2^ the cement industry is under pressure to implement strategies to limit global warming to about 1.5 °C to stay on track for net zero greenhouse gas emissions by 2050. Nevertheless, decarbonizing the cement industry presents a unique challenge because the vast majority of CO_2_ emissions come from the production of Portland cement (PC) clinker, which relies on limestone (CaCO_3_) and clays as raw materials. Therefore, modifying the current composition of PC would require a significant transformation of the construction industry as we know it.

### 1.2 Portland Cement and Related CO_2_ Emissions

Today’s construction industry originates in the second quarter of the 19th century with the birth of the so-called Portland cement.^3^ PC is manufactured by heating a mixture of limestone and clays to a temperature of about 1450 °C in a rotary kiln. Ordinary PC consists of approximately 95% of ground clinker and approximately 5% of gypsum. The clinker phases are synthetic calcium silicates produced by controlled sintering of calcium carbonate (CaCO_3_), alumina (Al_2_O_3_), silica (SiO_2_), and iron oxide (Fe_2_O_3_) in cement kilns at high temperatures. The primary raw materials used are crushed limestone (approximately 80–90%) and clay, mudstone, or shale (approximately 10–15%). Limestone, the calcium oxide source, is primarily composed of CaCO_3_. The clayey materials, which are the source of silicate, consist mainly of sheet silicates containing Al, Fe, and Mg. At a temperature of 1450 °C, CaCO_3_ is converted to CaO and CO_2_, and then, CaO further reacts with Al_2_O_3_, SiO_2_, and Fe_2_O_3_ to form the calcium silicate, aluminate, and ferrite phases. The resulting clinker typically consists of 67% CaO, 22% SiO_2_, 5% Al_2_O_3_, 3% Fe_2_O_3_, and 3% other components. It is composed of several phases, including alite (∼C_3_S, 48–68%), belite (∼C_2_S, 6–27%), calcium aluminate (∼C_3_A, 0–12%), and calcium aluminoferrite (C_2_(A, F), 4–13%).^4^ During the final stage of the manufacturing process, a low percentage of sulfate-bearing minerals, such as gypsum, bassanite, and anhydrite, are added to the clinker and ground together to produce the final fine cement powder, which is ready for use. The addition of calcium sulfate helps to control the rate of strength development by slowing down the rapid reaction of aluminate and ferrite phases, which can reduce the workability of the paste.^5^

CO_2_ emissions from cement manufacturing emissions can be divided into calcination-related emissions and fuel-related emissions. The calcination process involves the decomposition of limestone (CaCO_3_) into CaO and CO_2_, while the combustion of fossil fuels generates the high temperatures required for the process (ca. 1450 °C). These two processes are the main sources of CO_2_ emissions during the production of PC. Approximately two-thirds of total direct emissions are caused by CO_2_ released by raw materials, while the rest is mainly due to the combustion of fossil fuels.^2^ Lowering the emissions from cement manufacturing is, therefore, a challenging task compared to other materials, as energy efficiency improvements and switching to alternative fuels do not contribute as much as reducing the calcined limestone used to produce the cement clinker.

In 2021, the “Global Cement and Concrete Association (GCCA)” became the first global industry to present a detailed roadmap for decarbonizing the cement and concrete industry by 2050. The leading companies have pledged to achieve a reduction of 25% in the case of concrete and 20% in the case of cement by 2030. To achieve the ambitious goal of reducing emissions by five billion tonnes between 2021 and 2030,^6^ it is necessary to promote innovation in developing new cement chemistries, processes, and technologies.

From a purely material standpoint, the most effective way to reduce emissions is by lowering the clinker-to-binder ratio. This reduction impacts both CO_2_ emissions from CaCO_3_ calcination and emissions related to fossil fuels.^7^ However, reducing the clinker factor is only possible by boosting the material efficiency, which ensures adequate material performance even if the clinker content is reduced. This could be accomplished by optimizing the structure of the cement matrix at various hierarchical levels, ranging from the nano- over the meso- up to the macroscale. To achieve this challenge, it is essential to thoroughly comprehend the molecular and nanoscale mechanisms that govern the formation of the relevant cement binding phases. Understanding the crystallization mechanism of the relevant cement matrix’s phases can enable deliberate control over the hydration process and material properties. This is critical for producing efficient cement-based materials with a lower PC content while still meeting the requirements for adequate performance.

### 1.3 Toward Controlling the Crystallization of Cement Hydrates

In the context of PC, the hardening of cementitious materials occurs because of the reaction between the different clinker phases and water, resulting in the formation of hydrated products (Section 2). The hydration of calcium silicate phases results in the formation of two predominant hydrates: calcium silicate hydrate (C–S–H) and calcium hydroxide (CH), which characterize the cement matrix (Section 2.1). The properties of cement-based materials are primarily determined by the micro- and nanostructure of the network of hydrated solid phases, which is dominated by the C–S–H phase. This review provides a concise overview of the *atomic*, *nano-, and microstructure of C–S–H*, which has been extensively investigated and discussed for over a century, given its importance for paste cohesion and early strength development of cementitious materials (Section 2.2). The most accepted model proposes that cement hydrates form by self-assembling individual C–S–H nanoparticles with a defective tobermorite crystal structure. Furthermore, we include insights into the alterations in the C–S–H gel in blended cements with high aluminum content. Due to the current increase in the use of supplementary cementitious materials (SCMs), *calcium aluminate silicate hydrate (C-A-S-H)* emerges as the primary phase in blended cement, exhibiting distinct characteristics when compared to plain C–S–H (Section 2.3). Operating at various levels, these modifications can significantly influence blended cement’s performance, mechanical properties, and durability. Thus, uncovering the properties of the C-A-S-H phase has become a top priority in the field.

To regulate the properties of micro- and nanostructure of the network of hydrated solid phases, controlling their formation at the molecular and nanoscale levels from the onset of precipitation is essential. Section 3 aims to provide a comprehensive overview of the early crystallization of C–S–H due to its significant role. Many relevant material properties are determined from the onset of precipitation (e.g., size, morphology, crystallinity, polymorphism). Therefore, *nucleation* (Section 3.1) is considered the first crucial step in forming any solid substance. Beyond the conventional classical crystallization model,^8^ which only considers ions and the final crystalline material as the existing entities, recent research has revealed the formation of numerous minerals as a multistep process (Section 3.2). These models, commonly referred to as *nonclassical crystallization* pathways, diverge significantly from classical theories and challenge the conventional understanding of crystallization by introducing a new perspective on the transitional phases. The existence of novel intermediate stages, identified some decades ago for synthetic and biogenic minerals, also manifests during the early stages of C–S–H crystallization, providing new means to influence and ultimately control its formation process (Section 3.3).

Besides homogeneous nucleation, *heterogeneous nucleation* of cement hydrates can significantly impact the early strength development of cementitious materials (Section 3.4). Limited hydrate formation at early ages results from partial clinker replacement, which leads to reduced early strength. It is crucial to address this issue to enable higher levels of clinker substitution. One way to accelerate early age mechanical properties without compromising long-term strength and durability is to reduce the energy barrier for hydrate nucleation by introducing carefully selected surfaces.

Section 4 examines the hydration products, microstructure, early crystallization stages, and phase transformations of three major types of alternative binders proposed to replace PC in line with the IEA technological roadmap for reducing CO_2_ emissions in the cement industry.^2^ Our analysis centers on alkali-activated (AA) binders in Section 4.1, carbonatable calcium silicate cement (CCSC) Section 4.2, and magnesium oxide cements derived from magnesium silicates (MOMS) Section 4.3. We prioritize these alternative binders because in-depth academic research can substantially benefit their performance. To bring more sustainable alternatives closer to practical implementation, material challenges such as extended setting times, rapid setting, and hydrated phase instability can be addressed through controlled crystallization. Herein, we adopt a nonclassical perspective on the formation of the relevant binding phases, considering the growing body of experimental evidence suggesting multistep crystallization pathways in these alternative binders. Same as for C–S–H, nonclassical crystallization has great potential for regulating the formation process of alternative binders due to various intermediate stages that can be influenced by, for instance, using additives.

Section 5 presents the fundamental principles of *additive-controlled crystallization*, giving readers a basic understanding of how the intentional use of additives can influence and direct crystallization processes. This approach mirrors nature’s mastery in biominerals, where organic substances are employed to meticulously regulate the precipitation and structure of biomaterials, resulting in hierarchical hybrid structures known for their exceptional performance. In additive-controlled crystallization, the initial stages of particle formation are crucial, as this is where additives can significantly influence and regulate the final material obtained. Thus, the manipulation of the binding phase and overall properties of the resulting material depends on the interplay between additives, mineral precursors, intermediates, and emerging particles. For C–S–H, organic additives have been demonstrated to exert substantial influence on its formation by stabilizing amorphous precursor phases^9,10^ and interacting with prenucleation species present in the solution.^11,12^ Exploring nonclassical crystallization pathways for the relevant binding phases will certainly improve our understanding of how additives affect cement hydration. These insights can be extended to regulate the crystallization process of specific binding phases through careful selection of additives, opening new ways to control the nanostructure of the cementitious matrix from a bottom-up perspective.

In Section 6, we advance to a higher level of complexity in additive-controlled crystallization by focusing on the development of *bioinspired organic/inorganic composite materials*. This strategy aims to create more efficient, durable, and consequently, more sustainable cementitious materials. In pursuit of material efficiency, we draw inspiration from biominerals, considering them prototypes to imitate. Nature often employs the close association of inorganic and organic compounds in biological structures, resulting in outstanding materials (Section 6.1). In this context, we discuss the achievements to date and explore future possibilities of using organic additives to produce bioinspired C–S–H inorganic–organic nanocomposites (Section 6.2). We begin by summarizing the most relevant publications that focus on the incorporation of organics in C–S–H to explore later the molecular interactions that occur at the organic–inorganic interface. This discussion addresses a critical challenge, as mastering these interactions is vital to achieving the ultimate goal of cement material scientists: the precise control of the C–S–H nanostructure.

Finally, Section 7 presents the synthesis of mesocrystalline C–S–H/organic superstructures characterized by a high degree of organization, resulting in enhanced properties. If C–S–H building units are arranged in a brick-and-mortar structure, the resulting material will have a higher density with few or no pores, and consequently, a significant improvement in mechanical properties would be expected. Understanding the self-assembly of C–S–H platelets requires a grasp of mesocrystals and particle-based nonclassical crystallization concepts, introduced in Section 7.1. This bottom-up approach to obtaining ordered and defect-free C–S–H structure possesses a notorious challenge, which was overcome in the groundbreaking work led by the group of Helmut Cölfen (Section 7.2).^13^ A mesocrystalline C–S–H brick-and-mortar structure was fabricated, taking inspiration from the highly ordered nanoparticles found in the sea urchin backbone. The biomimetic composite includes adsorbed polymer functions that act as the ductile phase between the brittle and hard C–S–H ordered nanoplatelets, resulting in remarkably high flexural strength. This approach has the potential to considerably improve the fracture toughness and elasticity of brittle cementitious materials. From a sustainable perspective, it could facilitate the reduction of clinker content and, in some cases, even eliminate the need for steel reinforcement.

In summary, this article aims to illustrate how examining the early stages of crystallization of relevant cement binding phases, combined with structural investigations, can lead to innovative synthetic strategies for controlling the cement matrix’s structure, ranging from the atomic to the microscale. To accomplish this, we have compiled information from 621 studies selected for their relevance, coherence, and appropriateness of the observed results. It is important to note that, due to the vast amount of literature on this topic and the rapid developments in the field, there is a possibility that some studies may have been inadvertently overlooked.

## 2 Cement Hydration

**Figure 1:**
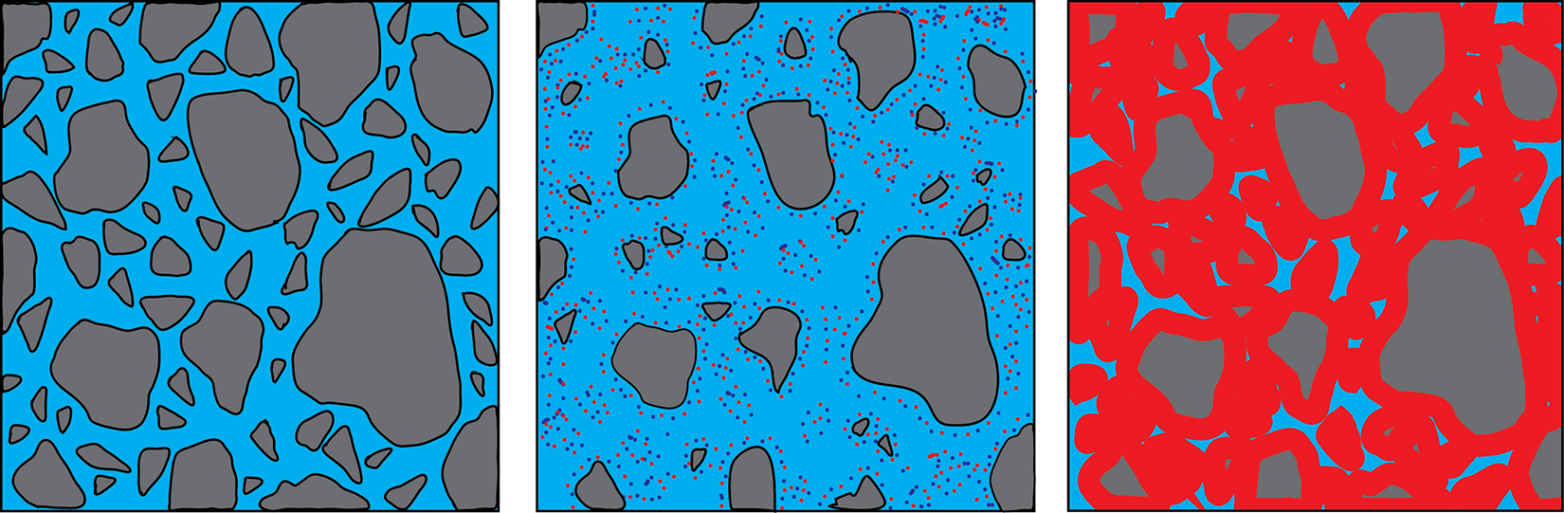
Schematic representation of cement hydration. Gray = unreacted cement grains, blue = water, and red = cement hydrated phases. Modified with permission from ref (16). Copyright 2018 Elsevier.

When cement powder is in contact with water, hardening results from the reactions between the clinker phases and water, which produces the hydration products (i.e., hydraulic reaction). The larger volume of the hydrated products compared to the unreacted grains and water and their lower solubility result in a stable solid matrix in which the unreacted grains and the rest of the mortar components are held together (Figure 1). Cement hydration can be understood as a coupled dissolution–precipitation reaction^14^ in which less stable phases (clinker phases) dissolve and more stable phases precipitate from the fluid phase (hydrated phases). The main physicochemical processes involved, which are analogous to the ones used to describe mineral replacement reactions,^15^ are summarized below:

1. *Dispersion* of the cement fine particles in water.
2. *Hydrolysis* of cement surfaces.
3. *Dissolution* of the cement particles, releasing to the pore solution their constituent ions, i.e., calcium, silicate, and aluminate. The reaction of these building units with water produces the hydrated phases.
4. *Transport or diffusion* of the building units across the solid–liquid interface.
5. *Supersaturation with* respect to hydrated phases is reached due to the increase in the concentration of the building units in solution as dissolution of the cement grains proceeds.
6. *Nucleation* of hydrated phases on the surface of the cement grains (heterogeneous nucleation) and in the pore solution (homogeneous nucleation).
7. *Growth* of the hydrates by *adsorption* and incorporation of the building units to the surface of the hydrated phases.
8. Other reactions, such as the formation of ion complexes, are also involved in the precipitation of the hydrated products, theoretically inhibiting the formation of those if nucleation is understood from a *classical* perspective^8^ since they will effectively lower the supersaturation.

**Figure 2:**
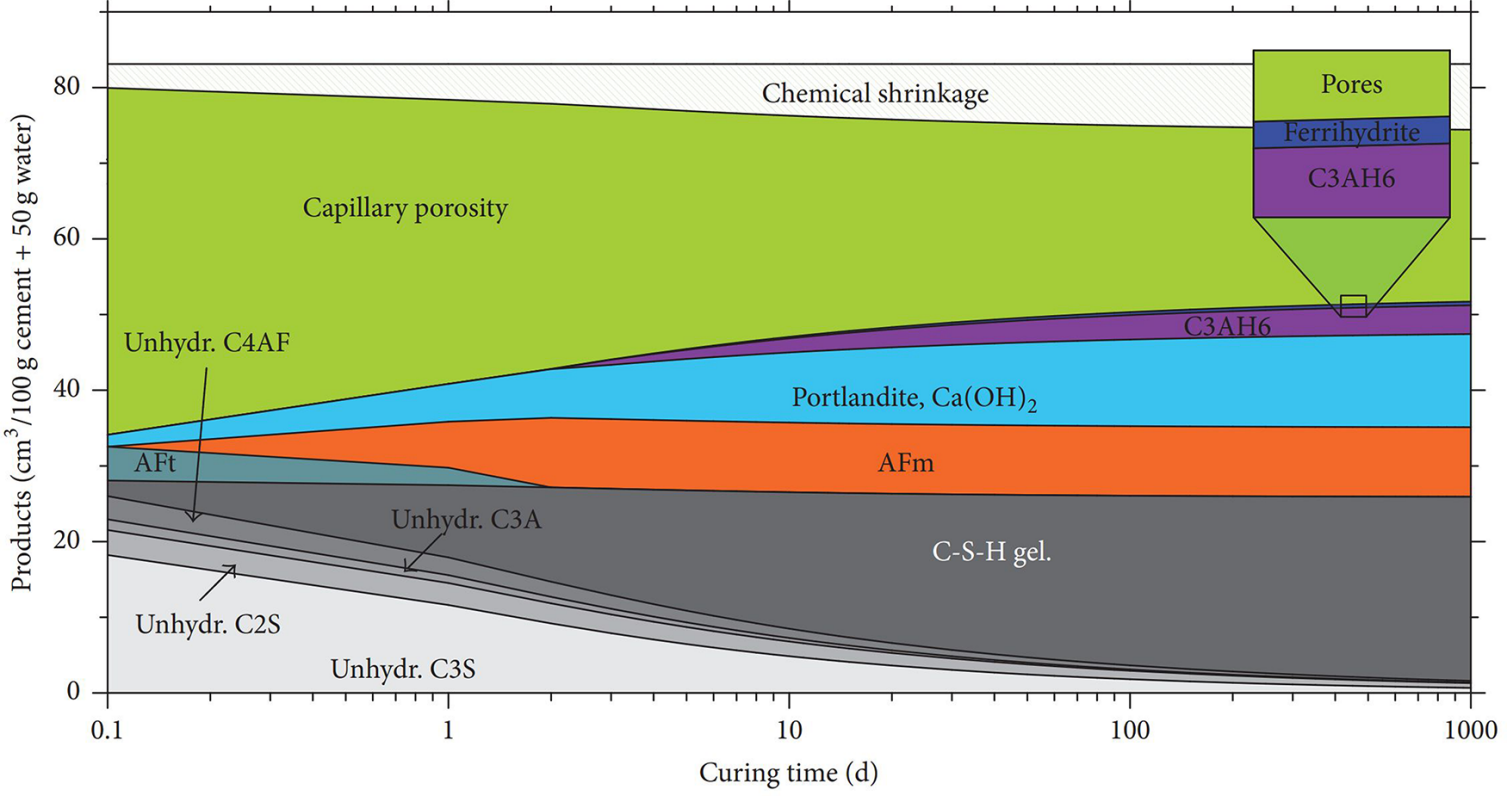
Hydrates evolution at 20 °C based on the thermodynamic simulation. Reproduced with permission from ref (17) under a 4.0 Creative Commons Attribution License (CC BY 4.0 DEED). Copyright 2017 Hindawi. https://creativecommons.org/licenses/by/4.0/.

The phases in equilibrium with the solution vary because the pore solution’s chemistry evolves during hydration. This changes the phase assemblage since the initially created solid phases dissolve, and new phases precipitate. The spatial distribution of the phases formed initially and the later redissolved-reprecipitated ones in the cement matrix yields complex microstructures. In Figure 2, the evolution of PC hydration over three years is depicted based on thermodynamic calculations at 20 °C.^17^ Hydration of the calcium silicate phases (C_3_S and C_2_S) produces calcium silicate hydrate (C–S–H) and calcium hydroxide (CH). In contrast, trisulfoaluminoferrite or ettringite (AFt) and monosulfoaluminoferrite hydrate (AFm) are the hydration products of the aluminate phases. Among the formed hydration products, CH can further react with other siliceous, aluminosiliceous, or calcium aluminosiliceous materials that lack hydraulic activity to form additional C–S–H (i.e., pozzolanic reaction).

**Figure 3:**
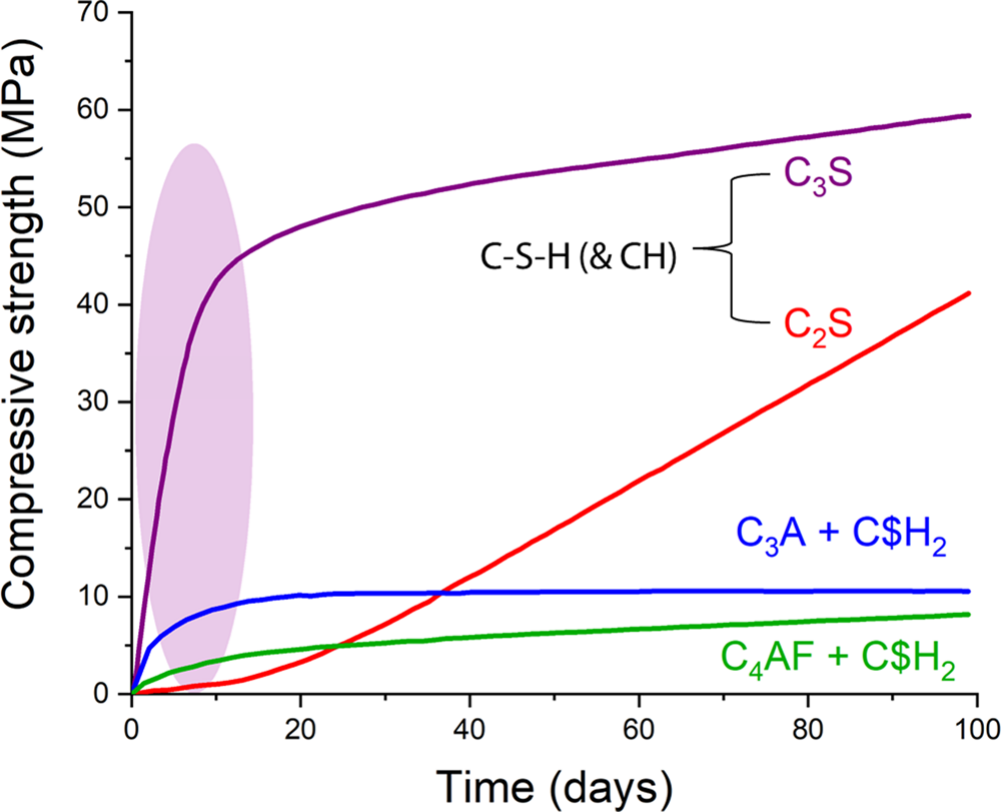
Compressive strength development in pastes of pure cement compounds over 100 days. Data from ref (19).

The formation of hydrated phases from the calcium silicates (C_3_S and C_2_S) gives rise to two types of hydrates: calcium silicate hydrate (C–S–H), which represents around 50–65 vol % of the final paste, and calcium hydroxide (CH), which corresponds to ca. 10–20 vol %.^4^ C–S–H, written with hyphens, is the generic term to refer to any amorphous or poorly crystalline calcium silicate hydrate with no “particular” composition, (CaO)_*x*_-(SiO_2_)_*y*_-(H_2_O)_*z*_.^4^ The cement community widely acknowledges C–S–H as the key contributor to cement’s early strength development, setting, and hardening,^18^ which subsequently influences the mechanical performance of mortars and concretes (Figure 3).^19^

### 2.1 Hydration Reaction of C_3_S/Alite

C_3_S hydration is the most relevant process since this calcium silicate polymorph represents 50–70 wt % of ordinary PC clinker.^20^ Due to its importance for the setting of PC-based materials, the hydration of the tricalcium silicate phase has been investigated for more than 100 years.^21^ Hence, it is a daunting task to make a complete examination of the topic, which the authors do not intend to accomplish with this review. We recommend that the readers consult the most recent reviews about alite/C_3_S hydration if a deeper understanding of the most plausible theories developed to describe this process is required.^5,15,18,22^ Most studies focusing on cement hydration restrict their attention to the hydration of alite/C_3_S because of its prominent role, and due to the increased complexity of the chemistry of multicomponent systems such as PC. Here, we briefly present the fundamental steps made so far in comprehending the hydration mechanism of alite/C_3_S, as understanding this process is crucial to improve the properties of cementitious materials. The overlap of the multiple processes involved in cement hydration and the heterogeneous character of cementitious materials make the investigations of the underlying mechanism of C–S–H formation not easily accessible.

**Figure 4:**
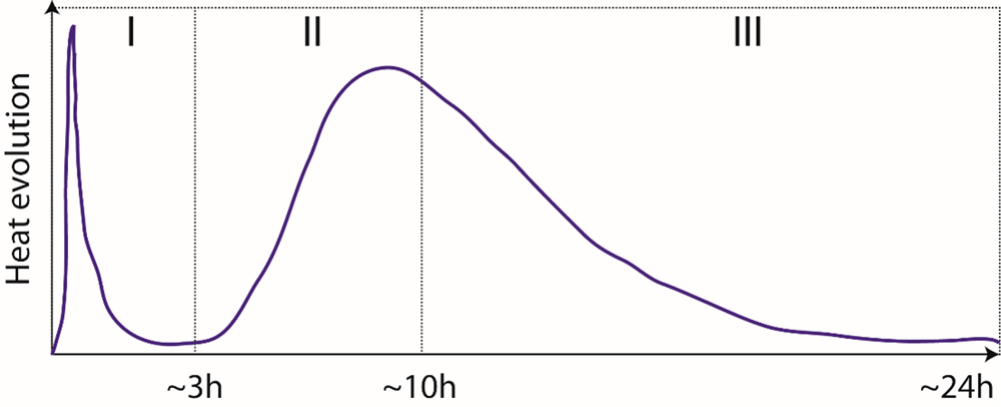
Typical heat evolution curve of alite is divided into three main periods, as discussed here.

Heat evolution over time has been traditionally used to follow the progress of the kinetics of the cement hydration reaction. Focusing only on alite (impure C_3_S), three main parts are distinguished in the calorimetry curve versus time based on the changes in the reaction rate (Figure 4). The **initial stage** (I) covers about 3 h and consists of a large exothermic signal lasting a few minutes, followed by a sharp decrease in heat and a period of low chemical activity called the “induction period”. The exothermic peak has been consensually attributed to the wetting of the cement grains and the dissolution of alite (exothermic process) in a highly undersaturated solution.^23,24^ The dissolution rate of the cement grains would be controlled primarily by the undersaturation of the solution with respect to the dissolving phase.^25^ The sudden slowdown in the reaction is then assigned to reducing the driving force (i.e., undersaturation) for alite dissolution caused by the increasing concentration of ions in solution as dissolution proceeds.^23^

This geochemistry-inspired mechanism of cement hydration states that etch tip formation dominates the dissolution of cement grains at the very beginning of the hydration (i.e., further from equilibrium). Step retreat governs the dissolution closer to equilibrium but still in a significantly undersaturated scenario.^23^ Key experimental evidence linked the duration of the induction period to the rate of dissolution of alite grains. As it is widely known in geochemistry, dissolution rates exhibit a strong dependence on the particle size and the presence of crystal defects.^26,27^ This was also the case for alite grains as higher specific surface areas (i.e., smaller particle sizes)^28^ and higher density of crystallographic defects resulted in a shorter induction period, confirming that the dissolution rate of the cement grains is affecting the launch of the acceleration period.^26,29^ During this first stage, C–S–H has been shown to nucleate after some minutes but does not grow extensively, most likely due to the lack of available C–S–H surfaces, as demonstrated by the shift on the onset of the acceleration period in the presence of C–S–H seeds.^30^

During the ***acceleration period (II)***, C–S–H and CH form massively, leading to the hardening of the cement paste or concrete.^4^ The most recent experimental evidence suggests that the rate of hydration during the main peak is governed by the nucleation and growth of needle-like C–S–H.^15,31,32^ During the acceleration period, the amount of needles increases, reaching a particular length rapidly. Afterward, the growth rate of the needles is reduced significantly. Most of the C–S–H needles have nucleated at the main peak, and the surface is mostly enclosed.^33^ The exothermic peak measured is again attributed to increased alite dissolution caused by C–S–H precipitation.^34^ The removal of ions from solution by precipitating hydrated phases would increase the undersaturation with respect to C_3_S and thus its dissolution, which raises the concentration of ions and the supersaturation with respect to hydrates again. In the *initial stage (I)*, it is suggested that the dissolution rate of C_3_S is controlled by the concentration of ions in solution since precipitation of C–S–H is scarce and does not remove a considerable number of ions from the media to influence the level of undersaturation with respect to alite greatly. In contrast, during the *acceleration period (II)*, precipitation of the hydrates and C_3_S dissolution are tightly linked, impacting each other significantly.^24^

**Figure 5:**
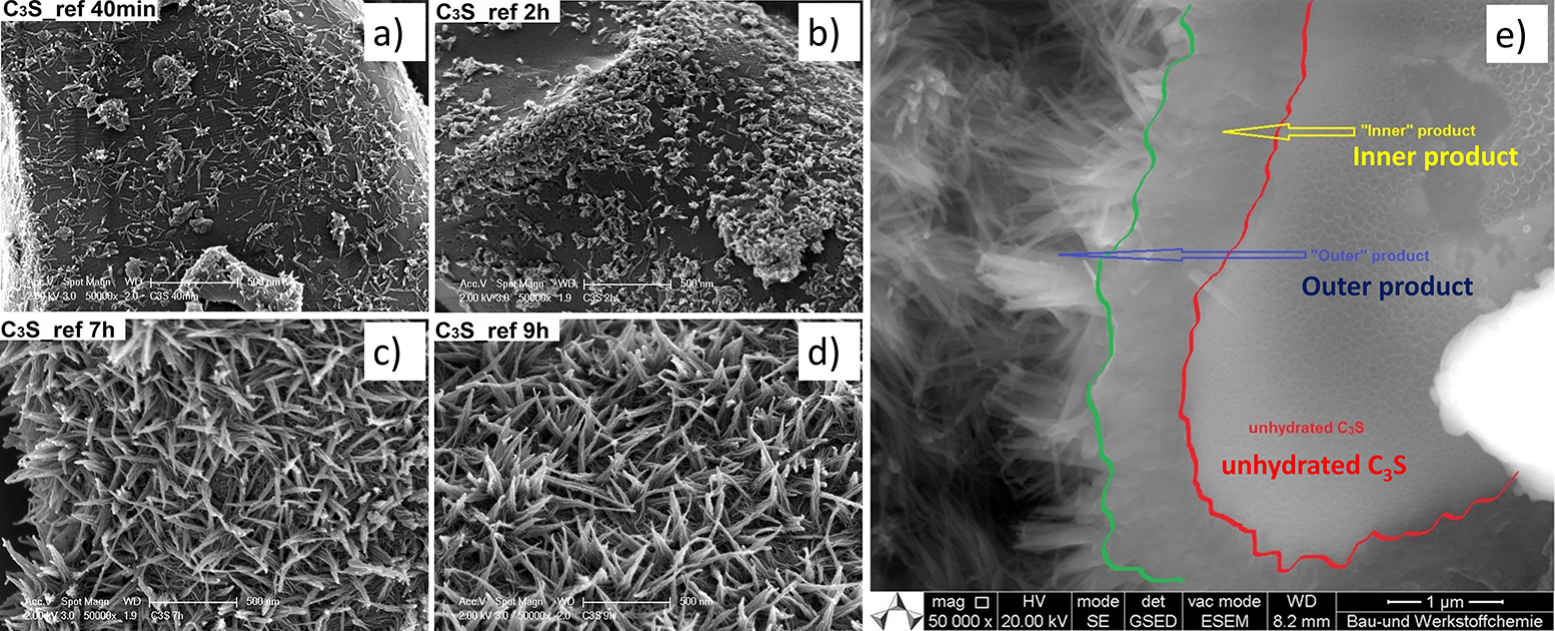
SEM images of C_3_S hydrated grains at different times: a) 40 min, b) 2 h, c) 7 h, and d) 9 h. Reproduced with permission from ref (36) under a 4.0 Creative Commons Attribution License (CC BY 4.0 DEED). Copyright 2022 Elsevier. https://creativecommons.org/licenses/by/4.0/. e). Environmental SEM image of a C3S grain after 96 h hydration. Reproduced with permission from ref (35). Copyright 2015 John Wiley & Sons.

The **deceleration period (III)** corresponds to the period after the main hydration peak, where the heat release decreases (Figure 4). The transition from the acceleration to the deceleration period is probably the most controversial topic in the hydration process.^22^ One of the recently proposed reasons for this transition is the change in the growth mode of C–S–H from needles (outer product) to a denser, more granular C–S–H (inner product) when the surface is completely covered with the needles (Figure 5).^35^ The formation of the inner C–S–H could be triggered by the reduction in the growth of the outer product mentioned above. C–S–H needles might form much faster in the *acceleration period (II)*, but afterward, their growth would slow down, and both products would form at a similar rate during the *deceleration period (III)*.^32^ Simulations have supported these experimental observations.^33^ This strengthens the hypothesis that the nucleation and growth of the needles (so-called 'needle-model') is sufficient to explain the *deceleration period (III)* in the heat evolution curve. However, the suggested mechanism leaves unaddressed points, such as the factors that restrict the growth of the C–S–H needles and, more fundamentally, how C–S–H grows.^22^

In the last decades, a deeper understanding of cement nano and microstructure has been achieved, considerably boosted by the creation of the NANOCEM consortium (https://www.nanocem.org/). NANOCEM brought together academic and industrial partners aiming to conduct fundamental research on the nano and microscale processes occurring during cement and concrete formation. Despite advancements in the comprehension of cement hydration, intensive research is still needed. One vital aspect concerns the mechanism of C–S–H nucleation and growth since most of the properties of cementitious materials are connected to the C–S–H nanostructure; hence, this is critical knowledge for developing more sustainable PC-based cementitious materials.

### 2.2 Calcium Silicate Hydrate (C–S–H) Nano- and Microstructure

**Figure 6:**
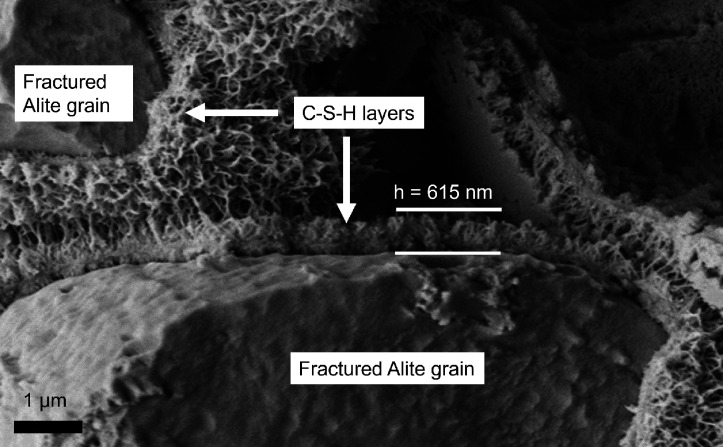
C–S–H formation on alite grains after 4 h of hydration imaged by cryo-SEM. Reproduced with permission from ref (40). Copyright 2016 Elsevier.

Considerable efforts have been devoted to uncovering the C–S–H nanostructure for more than a century,^37^ leading to significant advances in understanding this technologically relevant binding phase. C–S–H emerges from the surfaces of the nonhydrated calcium silicate grains acting as a nanoglue in concrete and mortars (Figure 6). It is a noncrystalline or poorly crystalline material of variable composition, (CaO)_*x*_-(SiO_2_)_*y*_-(H_2_O)_*z*_, that tends to incorporate impurities and contains chemically and nonchemically bound water. This material is especially difficult to characterize due to its amorphous nature, heterogeneity across several orders of magnitude, sensitivity to temperature and humidity conditions, and the variability of the clinker phase composition.^38^ In this regard, the implementation of the most advanced experimental techniques to study cementitious materials (electron microscopy with X-ray energy dispersive spectroscopy, atomic force microscopy, nuclear magnetic resonance, neutron scattering, and computational methods) has made a pivotal contribution to the understanding of C–S–H at the nano- and microscale. Nevertheless, many questions are still debated, such as the colloidal or continuous character of the C–S–H structure, the role of water, or the amorphous/very poorly crystalline/nanocrystalline character of the material.^39^

**Figure 7:**
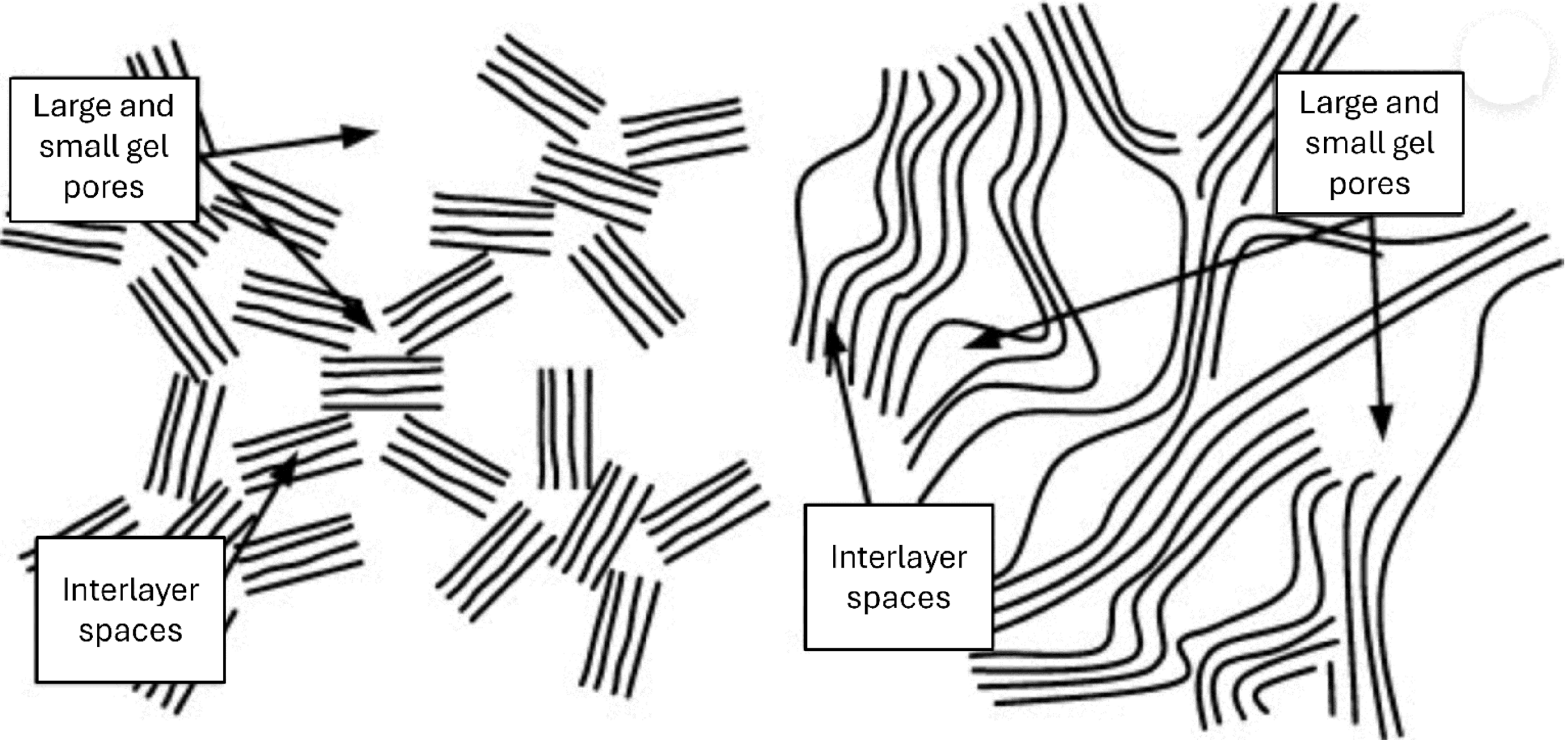
Schematic of the two dominant C–S–H models. Left: the colloid model is a hierarchical packing of nanosized layered particles. Right: the layered model describes C–S–H as disordered quasi-continuous sheets. Reproduced with permission from ref (44) under a Creative Commons Attribution License (CC BY 3.0 DEED). Copyright 2014 Creative Commons Attribution License. https://creativecommons.org/licenses/by/3.0/.

The structure of C–S–H was first discussed at the Faraday Society meeting in London in 1918.^37^ This was the first instance where the theory of colloids versus crystalloids was used to describe cement hydrates. Almost 30 years afterward, Powers and Brownyard published the first model for hydrated cement paste, which suggested the colloidal character of C–S–H based on experimental data of water vapor isotherms.^41^ Following, Feldman and Sereda challenged this notion of colloidal C–S–H. They proposed a C–S–H irregular structure consisting of crumbled tobermorite sheets,^42^ based on N_2_ adsorption isotherms.^43^ Although a plethora of models of the C–S–H nanostructure has been proposed in the last century, it is striking that the modern models are still constructed on the colloidal and layered models (Figure 7) developed more than 60 years ago, when highly advanced characterization techniques were not available. A critical analysis of the most relevant experimental and computational models, their main characteristics, and the experimental evidence on which they are based can be found in the review by Papatzani et al.^39^

#### 2.2.1 Colloidal Model for Describing C–S–H

The current most acknowledged model to describe C–S–H and its characteristics (i.e., density, specific surface area, porosity, and water content) is Jenning’s model, or the so-called colloid model (CM). It was founded on packing C–S–H particles as the mechanism to generate the cement hydrates.^45^ Here, the C–S–H gel is described as a colloidal or nanogranular material based on its extensive local deformations upon drying, which would not be feasible if the gel structure were related to a continuous porous material. The large deformations were ascribed to the collapse of the C–S–H network, where C–S–H behaves analogous to a clay-like material instead of a continuous material containing pores.^46^ Furthermore, in terms of aging, the resemblances of the C–S–H gel with colloidal systems have been highlighted.^47^

**Figure 8:**
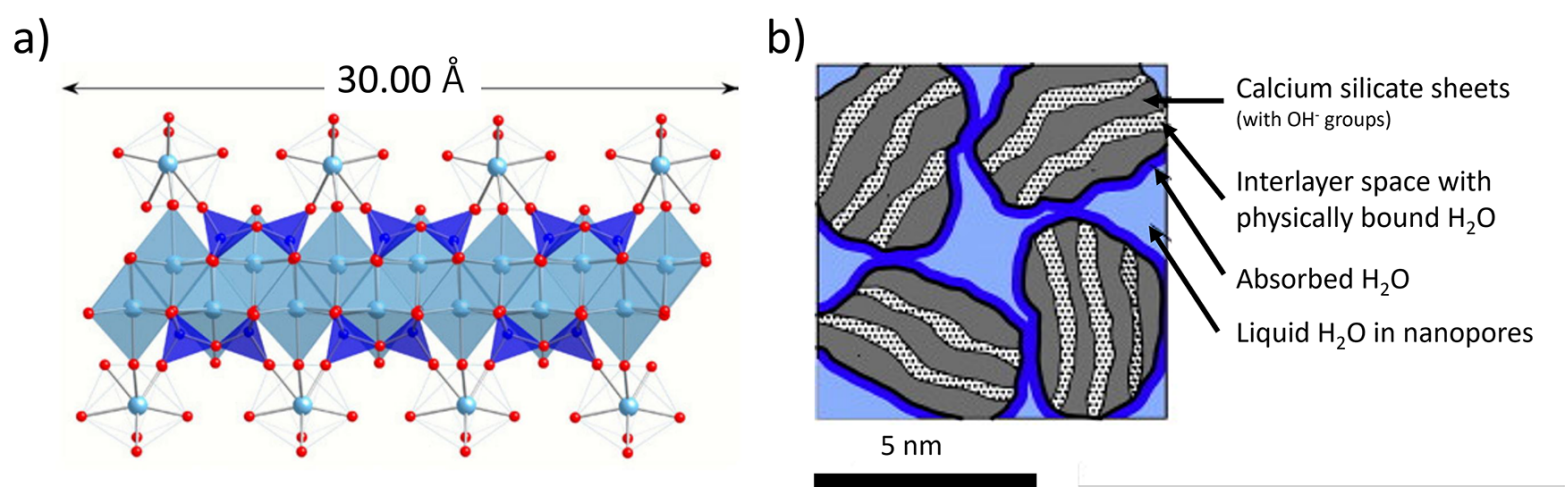
a) Proposed C–S–H structure with a calcium-to-silicon ratio of 1.5:1. Reproduced with permission from ref (53). Copyright 2004 Elsevier. b) C–S–H building blocks as described by ref (55) and reproduced with permission from ref (39). Copyright 2015 Elsevier.

The basic C–S–H unit within this model consists of lamellae with a thickness in the nm range, composed of silicate-calcium-silicate layers separated by water molecules and zeolitic calcium ions, which are held together by strong ionic–covalent bonds.^48^ It is generally accepted that the C–S–H crystalline structure is based on the layer structure of defective tobermorite (Ca_4_(Si_6_O_18_H_2_)·4H_2_O),^49,50^ first proposed in the 1950s.^51^ Tobermorite is a natural calcium silicate mineral consisting of CaO layers sandwiched between parallel infinite silicate chains. In the C–S–H gel, those layers contain shorter oligomers arranged in the so-called “Dreierketten” motif.^4^ This motif is based on two pairing silicate tetrahedra linked to the CaO central layer (Q^2^) and one bridging tetrahedron pointing to the interlayer space (Q^2p^).^52^ In real cement hydration (high Ca/Si ratios), bridging tetrahedra may frequently be missing and exchanged for Ca^2+^ ions, resulting in a C–S–H structure dominated by silicate dimers (Figure 8a).^53^ By small-angle neutron and X-ray scattering methods, a density of 2604 kg/m^3^ with the chemical formula (CaO)_1.7_(SiO_2_)(H_2_O)_1.80_ has been determined for the basic C–S–H layered bricks (cross-section of 5 nm)^54^ present during the hydration reaction (Figure 8b).^55^ These platelets seemingly do not evolve to larger dimensions at longer hydration times, but they instead increase in number and aggregation state.^56^

#### 2.2.2 C–S–H Assemblies

**Figure 9:**
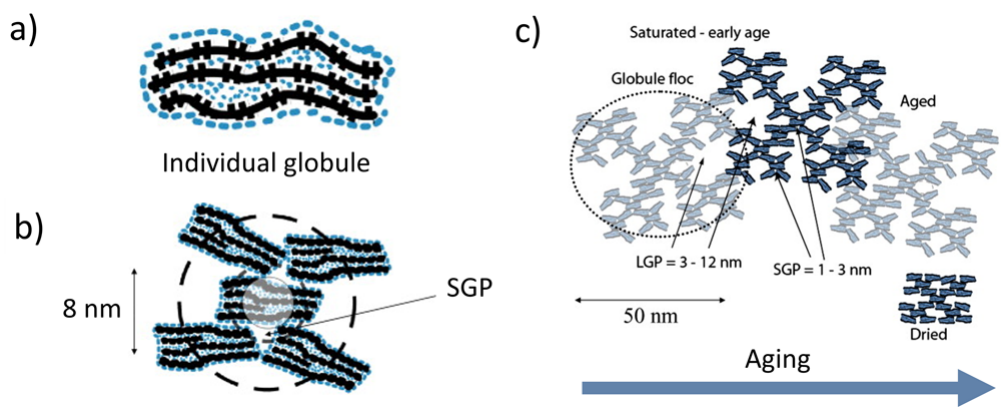
a) A schematic of a globule. b) Packing of globules showing small gel pores (SGP). c) The aging process is schematically represented as progressing from left to right. The large gel pores are reduced in size and volume during aging, and the globules align. Modified with permission from ref (54). Copyright 2008 Elsevier.

The colloidal model envisions hierarchical features within the C–S–H gel. The first level relates to the silicate-calcium-silicate layers separated by water molecules and zeolitic calcium ions, which assemble, constituting the C–S–H primary building blocks of nanometer dimension (Figure 9a). These building units are referred to as “globules” in the literature, despite of their proven nonspherical nature.^55^

**Figure 10:**
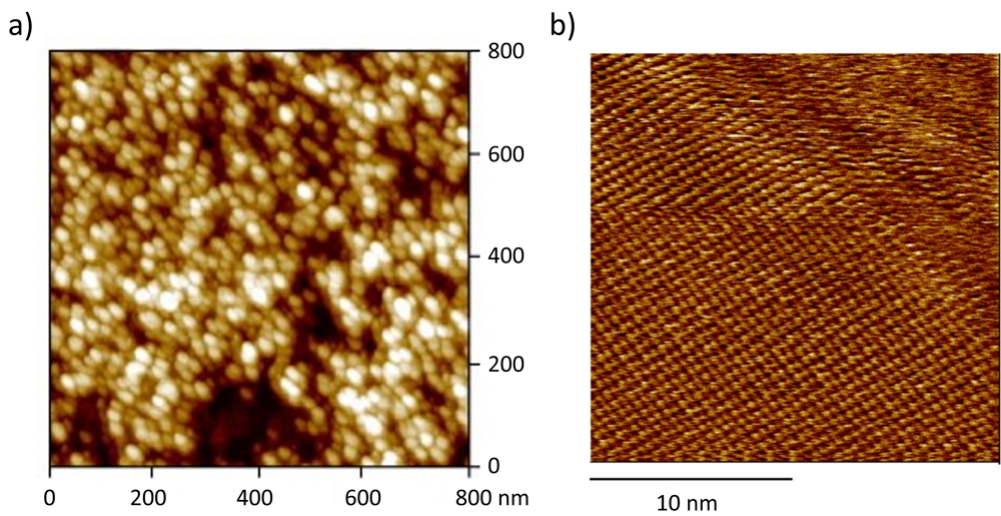
a) AFM image of hydrated cement paste on a calcite single crystal. C–S–H nanoparticles are clearly identified (60 × 30 nm^2^ and 5 nm thick). The darker zones correspond to pores. b) Atomic scale AFM image of a C–S–H (Ca/Si = 1.5) crystallized on a calcite single crystal. Reproduced with permission from ref (57). Copyright 2004 Elsevier.

In the second level, C–S–H nm-units assemble into statistically well-defined fractal patterns, which contain small gel pores (Figure 9b) and form larger entities with sizes between 30 and 60 nm, referred to as “globule flocs” by the authors (Figure 9c).^54^ Similar sizes have been observed in atomic force microscopy investigations of C–S–H growth on calcite surfaces, where individual 5 nm-thick platelets with lateral dimensions of approximately 60 nm were identified (Figure 10a). Atomic resolution imaging of the platelets shows a well-ordered structure over a micrometer where no sign of an aggregation-based process can be distinguished (Figure 10b).^57^ However, it should be noted that C–S–H formation on calcite surfaces is crystallographically controlled (see Section 0), and thus, the C–S–H product, in this case, could be unique.

In the third level, the “globule flocs” pack together and overlap, reducing the porosity and increasing the average density compared to a single “globule floc”.^58^ A hierarchy of nanoscopic pore spaces is formed upon packing. Apart from the intraglobule spaces (IGP) and interlayer spaces, two other types of pores are generated from the different stacking of the globules: the large gel pores (LGP) of 3–12 nm and the small gel pores (SGP) of 1–3 nm. There is also speculation that the interpenetration between the globule flocs might increase over time, yielding a reduction of the LGP at later ages (Figure 9c).^58^ This building-*block*-based approach for describing cement hydrates creates new possibilities for the development of innovative cementitious materials by levering the fundamental principle of hierarchical organization seen in biological materials.

**Figure 11:**
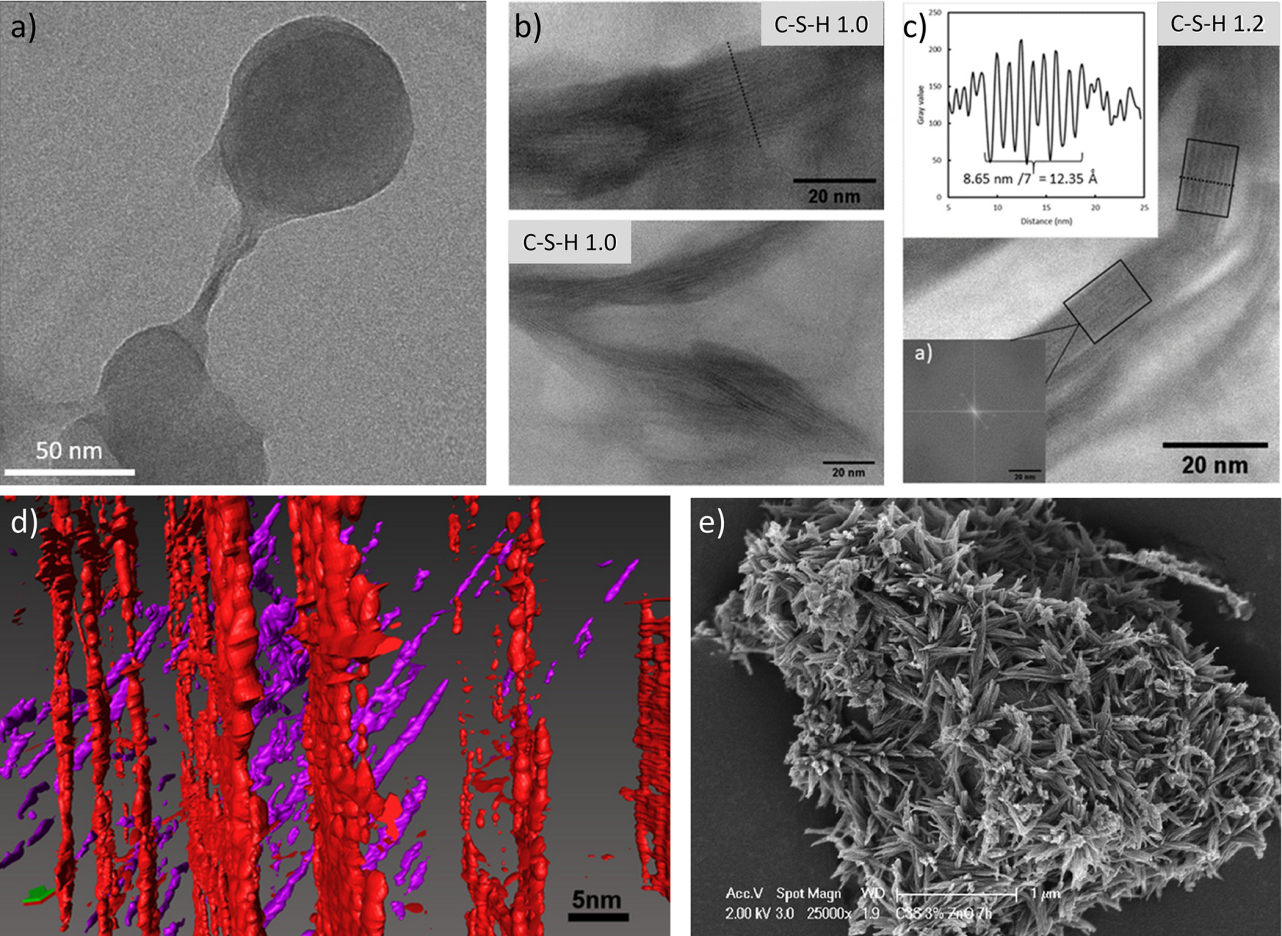
TEM and SEM images of different C–S–H morphologies a) Globular particles obtained in the presence of PCE. Reproduced with permission from ref (11). Copyright 2018 Elsevier. In situ STEM bright field images of b) C–S–H 1.0 and c) C–S–H 1.2. Inset: layer-to-layer distance measured along the dotted lines and fast Fourier transform of the areas denoted with black rectangles. Reproduced with permission from ref (61). Copyright 2020 American Chemical Society. d) Enlarged 3D reconstruction of C–S–H interstitial pore network formed in hydrated C_3_S obtained by computer tomography. The two separate networks of pores (red and magenta) are attributed to two particles of the same phase in alternate orientations. Reproduced from ref (63) with permission. Copyright 2015 The American Ceramic Society. e) SEM image of C–S–H needles on alite grains during acceleration. Reproduced from ref (33) with permission. Copyright 2019 Elsevier.

The study of the in situ growth of C–S–H poses significant challenges, as it is difficult to follow the formation of assembled structures over time. Hence, researchers have employed electron microscopy techniques such as transmission electron microscopy (TEM), scanning electron microscopy (SEM), and their cryogenic variants. These methods are essential for exploring the nanoscale and microscale morphology of C–S–H, providing a detailed view of its structural features. An extensive collection of C–S–H morphologies collected by electron microscopy is summarized in a 2024 review by Yan and Geng.^59^ In general, the morphological attributes of C–S–H can be categorized into three main types: globular, sheet-like, and needle-like structures (Figure 11).

*Globular* C–S–H particles are mainly observed in synthetic C–S–H systems,^9^ and in most cases, in the presence of organic additives.^10,60^ It is important to note that these globular structures are distinct from the “globule” term used in literature to refer to the individual C–S–H building block. The size of these particles is around 50 nm (Figure 11a), unlike the basic C–S–H unit, which is approximately 5 nm. Regarding their crystallinity, these globular particles have been characterized as amorphous or poorly crystalline, while the C–S–H basic unit exhibits short-range order, as we saw earlier. They tend to form larger agglomerates.^10^

*Sheet-like* morphology is the most reported for C–S–H (Figure 11b and c).^61^ However, it does not appear as a flat surface of well-packed 5 nm building blocks but as wavy or buckled sheets or ribbons.^62^ The nanocrystalline layered structure of the sheets at two synthesized C–S–H with different Ca/Si ratios (1.0 and 1.2) was beautifully imaged using in situ STEM by Gaboreau (Figure 11b and c).^61^ Individual C–S–H layers were stacked coherently, with the number of stacked units correlated with the Ca/Si ratio. Four to eight layers are stacked for C–S–H 1.2, while three to six layers form the crystallites for C–S–H 1.0. The layer-to-layer distance varied between 14 and 12.2 Å.^61^ Taylor et al. imaged the 3D network of hydrated C–S–H sheets and its pore network using computer TEM tomography in both synthetic C–S–H and C–S–H obtained from C_3_S hydration.^63^ They showed that the pore network is composed of a “sheet of voids” (Figure 11d) with an estimated diameter of around 1.68–2.4 nm. The distance between voids, which corresponds to the thickness of the C–S–H sheets, was approximately 5–8 nm, which agrees with their SAXS measurements,^63^ the results of Gaboreau,^61^ and several other publications.^59^

As introduced above, the needle-like C–S–H appears at the end of the induction period during real cement hydration, growing from the cement surfaces (Figure 11e). The aspect ratio of these needles typically exceeds 10, and they still retain a defective tobermorite structure.^53^ They consist of aggregates of primary elongated particles of approximately 1.5 to 3 nm in thickness and ranging from a few nanometers to several tens of nanometers in length, which aligned along their longitudinal direction.^53,64^ The alignment of the primary particle aggregates in an oriented fashion yields the thickening of the needles as hydration proceeds. After 50 years of hydration, the needles have been shown to reach diameters of ∼200 nm.^64^

Examination of hydrated cement grains revealed the coexistence of two types of C–S–H products with varying densities when needle-like C–S–H morphologies are present. These two distinct agglomerates, namely high-density (HD) and low-density (LD) C–S–H, have been experimentally identified through nitrogen adsorption,^65^ neutron-scattering,^66^ and nanomechanical measurements.^67^ The different packing densities of C–S–H building blocks have been hypothesized as responsible for the observed variations in the two products.

**Figure 12:**
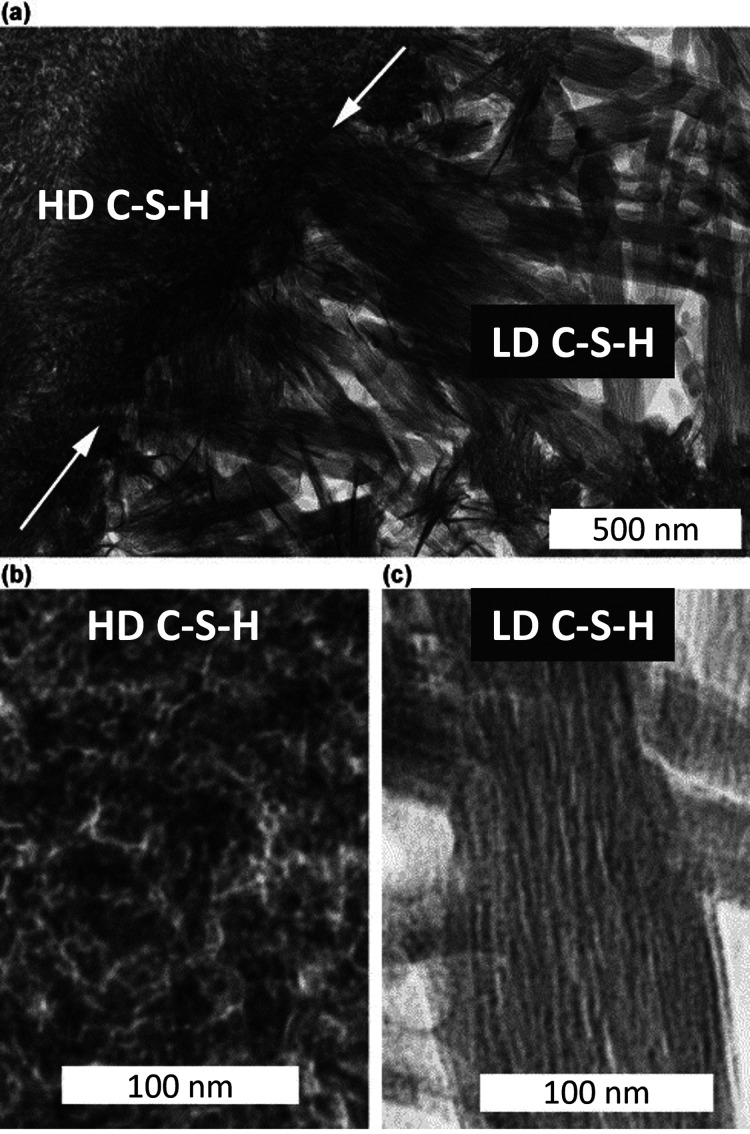
(a) TEM image showing HD C–S–H and LD C–S–H present in a hardened C_3_S paste with w/c = 0.4 hydrated at 20 °C for 8 years. White arrows indicate the boundary between the HD C–S–H and the LD C–S–H. (b) An enlargement of a region of HD C–S–H. (c) An enlargement of a fibril of LD C–S–H. Reproduced with permission from ref (53). Copyright 2004 Elsevier.

The distinct nanostructures of both products can still be seen in the cement paste after eight years (Figure 12).^53^ The LD C–S–H, denoted as the outer product, is mainly characterized by C–S–H needle-like structures that grow radially throughout the matrix. On the contrary, the HD C–S–H, referred to as the inner product, forms at the interface with unreacted grains, has been suggested to be the result of the aggregation of globular particles, and appears to be featureless.^64^ The transition in the growth mode of C–S–H from needles (1) to a denser and granular C–S–H (2) has been linked to a reduction in the growth rate of the needles, which occurs about 20 h of hydration. The reason for this transition is still under debate in the cement scientific community. Several factors have been proposed as possible causes, including changes in the Ca/Si ratio, variations in the supersaturation between the inner gap solution and the pore solution, or a slowdown in needle growth attributed to defect accumulation.^33^

**Figure 13:**
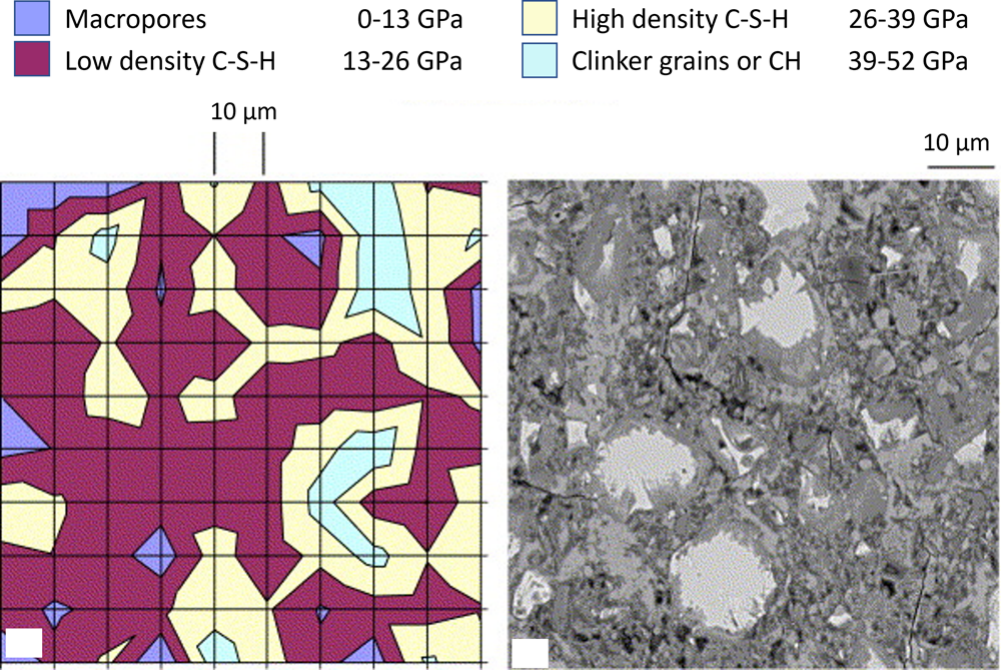
On the right, plan views of mechanical maps of indentation modulus. On the left, a similar magnification of an SEM image is also shown for comparison (courtesy of K. Scrivener). Reproduced with permission from ref (67). Copyright 2007 Elsevier.

The statistical analysis of hundreds of nanoindentation measurements conducted by Constantinides and Ulm showed that the stiffness and hardness increase with the packing density where HD C–S–H present higher values than LD C–S–H (Figure 13).^67^ The authors proposed that the mechanical behavior relates to nanogranular material, indicating the presence of a distinct C–S–H nanoparticle subunit. This subunit is conceived as the basic building block in all C–S–H cementitious materials and is believed to consist of stacked C–S–H sheets exhibiting 18% nanoporosity.

Although the colloidal notion of C–S–H is widely acknowledged, and the concept of two C–S–H products with two densities (i.e., two distinct surface areas and internal porosities) has “solved” the problem of inconsistent literature values for the specific surface area of C–S–H,^65^ this model has major limitation(s) since it fails to describe the observed kinetics of hydration.^44^ Therefore, several authors, like Gartner et al., developed the concept that C–S–H grows basically as two-dimensional crystalline elements,^62^ closer to the “*Feldman-Sereda* school”. He proposed a growth mechanism for C–S–H sheets involving the attachment of silicate tetrahedra into growing silicate chains and the incorporation of calcium and hydroxyls in the layers in between, resulting in either a tobermorite-like or jennite-like C–S–H.^68^ As mentioned above, including the large number of variants of these two main models developed over the past century and discussing their validity is beyond the scope of this review.

Investigating the formation process of C–S–H assemblies from its basic building unit is a difficult experimental challenge, and thus, nanoparticle-based simulations have emerged as a valuable tool to examine the formation of C–S–H structures at the nano and micro scale.^69^ Coarse-grained modeling tools enable the simulation of building block assemblies on the order of hundreds of nanometers. Thus, they have been widely used in the biological materials community for simulating hierarchical structures such as collagen fibrils.^70^ While the implementation of atomistic simulations in the cement field is relatively recent compared to other disciplines, it is growing rapidly. These simulations have significantly contributed to enhancing our understanding of the complex chemistry of cementitious materials, complementing and, at the same time, making use of experimental results.^71^ For instance, the knowledge concerning the atomic structure of the C–S–H building blocks,^50,72,69^ and its surface characteristics,^73^ the dissolution of clinker phases,^74^ the physicochemical properties of admixtures,^75^ the drying shrinkage in cementitious materials^76^ and the mesoscale structure and properties,^69^ have all greatly benefited from the application of computational models.

**Figure 14:**
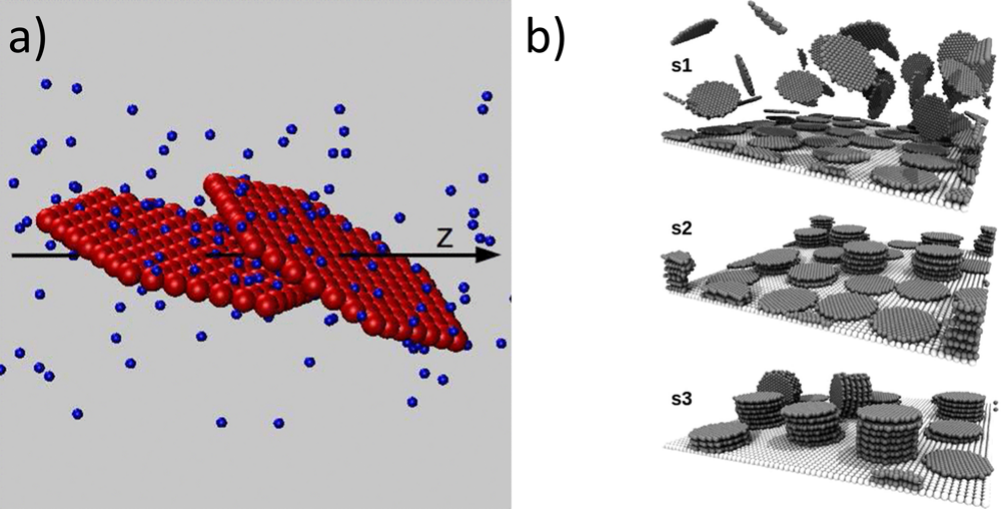
a) Schematic representation of two platelets in a salt solution. The sites of the platelets are shown as red spheres, while the divalent counterions are shown as blue spheres. b) Snapshots from MD simulations of C–S–H platelets adsorbed on a C_3_S surface. Ions are omitted for clarity. From top to bottom snapshots for C–S–H particles bearing a weak (s1), medium (s2), and strong (s3) negative charge density. The surface charge densities of the C–S–H platelets and the C_3_S surface are set equal. Reproduced with permission from ref (79). Copyright 2016 American Chemical Society.

At the nanoto-micro mesoscale, the most relevant one for this review, the formation of C–S–H assemblies has been simulated by the physicochemical interactions of discrete nanoparticles akin to those of atoms (Figure 14). Here, the electrostatic attraction arises from the extremely high surface charge density of the individual subunits in the presence of Ca^2+^ in an alkaline media.^77^ At high pH (ca. 13), deprotonated silanol groups (Si–O^–^) on the platelet’s surface interact strongly with the calcium counterions in solution,^78^ leading to a net attractive interaction between the C–S–H nanoplatelets that fosters their irreversible aggregation^79^ creating disordered assemblies.^80^ This unique combination of a high specific surface area of the C–S–H networks (i.e., large contact area between the building blocks) and the strong attraction between the nanoplatelets has been proposed to be responsible for the cohesion of cementitious materials.^81^ We recommend the recent review paper by Ioannidou et al. for an excellent overview of the current advancements in coarse-grained and mesoscale simulations concerning the formation of C–S–H mesostructure.^69^

To conclude this section dealing with the C–S–H nanostructure, it is important to note that in a polymer-free system, exerting any control over the formation of ordered C–S–H structures is not feasible due to the strong attraction between individual platelets, which results in the irreversible and disordered aggregation of the C–S–H building blocks.^54^ To achieve an ordered structure, the C–S–H subunits would first have to be stabilized, and then their aggregation would have to be controlled. This complex bottom-up approach certainly requires a thorough understanding of the formation of the C–S–H platelets, their interaction with organic additives, and their structure at various length scales, which will be discussed in sections 6 and 7.

### 2.3 Characteristics of Calcium Aluminate Silicate Hydrate (C-A-S-H) in Blended Systems

From an environmental perspective, replacing clinker with minerals additions (i.e., supplementary cementitious materials (SCMs), and fillers) is one of the best approaches to reduce the CO_2_ emissions from concrete and other cement-based materials,^82^ as it hardly affects clinker production (i.e., no new kilns are needed).^83^ However, the hydrates formed in the presence of SCMs differ from the ones resulting from the exclusive hydration of PC (due to the lower calcium content of most SCMs), which influence the volume, porosity, and microstructure of the paste and ultimately affect the strength and durability of the material.^84^ In Section 2.1, we described the main phases formed during PC hydration: C–S–H, portlandite, ettringite, and monosulfoaluminoferrite hydrate. However, the hydration process in the case of blended systems is more complex since the hydraulic/pozzolanic reaction of the SCM and the PC hydration occur simultaneously, eventually influencing each other. This section aims to emphasize the major alterations of the principal binding phase, C–S–H gel, which transforms into calcium aluminate silicate hydrate (C-A-S-H) in cement formulations incorporating Al-containing SCMs.

Most SCMs introduce additional Al and Si during the hydration process, leading to notable alterations in the composition of the C–S–H phase compared to plain PC. For instance, the utilization of Si-bearing SCMs has been demonstrated to reduce the Ca/Si ratio in the C–S–H phase, extending the mean silica chain length.^85−87^ This promotes the incorporation of the other major component of SCMs, Al, in the C–S–H gel, yielding C-A-S-H.^88^ C-A-S-H stands as the primary binding phase in blended cement concrete, and thus, a substantial surge in research attention has been dedicated to this hydrate over the past two decades.

**Figure 15:**
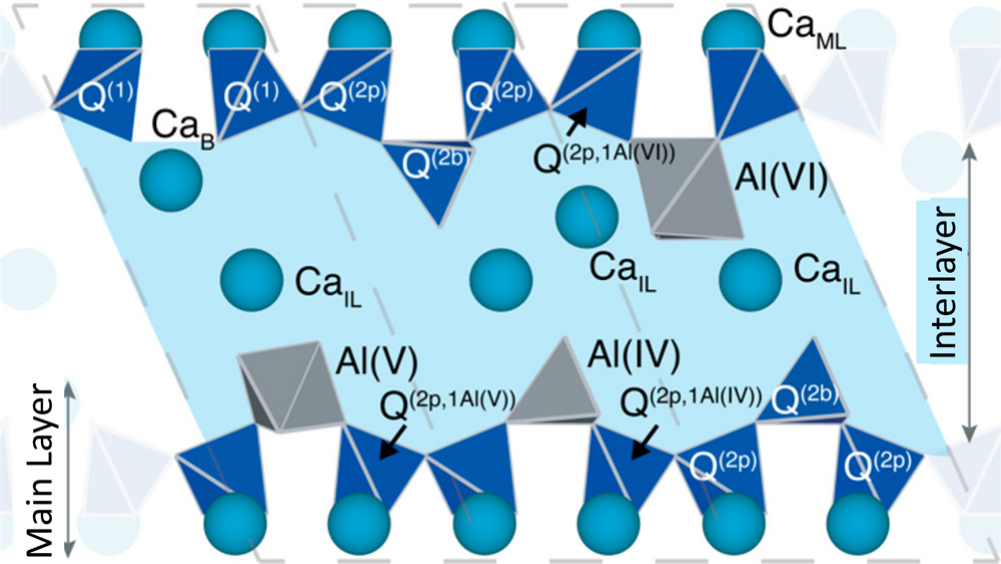
Schematic showing three C-A-S-H structural units representing the layered bulk structure of C-A-S-H. The dashed lines indicate the structural unit cell boundaries. The calcium atoms are shown as turquoise spheres. The interlayer is shown in light blue color, depicting the presence of water and hydroxyl ions, which are omitted for clarity, in addition to interlayer calcium ions (CaIL). The silicate species (dark blue) are labeled based on their connectivity and position. The polyhedral shapes of aluminum are shown in gray. Reproduced with permission from ref (95). Copyright 2020 American Chemical Society.

C-A-S-H denotes a calcium silicate hydrate phase incorporating Al into its structure, typically with an Al to Si atomic ratio equal to or less than 0.25. Like in C–S–H, the structure of C-A-S-H also consists of linear silicate chains on both sides of the CaO central layer (Figure 15). More than two decades of research have unveiled three distinct types of aluminates in C-A-S-H samples: four-coordinate Al^IV^, five-coordinate Al^V^, and six-coordinate Al^VI^ species.^89−91^ The Al^IV^ arises from the substitution of Si^4+^ by Al^3+^ in the silicate chains. Notably, at low Ca/Si ratios, NMR measurements on a pure C-A-S-H phase have demonstrated that most of the Al^IV^ substitute the bridging silicates,^88^ which is the thermodynamically favored state.^92^ This incorporation results in an increased average aluminosilicate chain length.^93,94^ The introduction of Al^V^ substitutions has been attributed to the replacement of Ca^2+^ by Al^3+^ in the interlayer, maintaining charge balance within the Al^3+^ sites in the silicate chain structure.^90^

At high Ca/Si ratios (above 1.5), Al^VI^ with an octahedral geometry emerges as the dominant species. However, the exact position of its incorporation is still the subject of ongoing debate within the research community. Initially, it was hypothesized that the incorporation of Al^VI^ species, identifiable by a distinctive NMR peak at 5 ppm, occurred within the CaO layer of C–S–H.^89^ Later, this peak was assigned to a separate phase termed “third aluminate hydrate”.^90,96^ However, recent findings have disproved the existence of this separate phase, instead identifying the Al resonance at 5 ppm as belonging to bridging Al^VI^ species in bridging positions.^95^

The presence of distinct Al species in the gel seems to be a key factor affecting the nanomechanical properties of C-A-S-H. Recent studies have reported that C-A-S-H predominantly exhibits tetrahedral coordination (Al^IV^) over extended curing periods, whereas Al^IV^ and Al^V^ are observed at shorter ages.^97^ Remarkably, long-cured C-A-S-H samples (Al/Si = 0.1) exhibit greater stiffness in comparison with C–S–H and short-cured C-A-S-H samples (Al/Si = 0.05). This hints at a possible enhancement of the mechanical properties of the gels with Al^IV^ incorporation.^97^ Including Al tetrahedral has also been shown to facilitate the cross-linking of adjacent aluminosilicate chains in low-calcium cements (Ca/Si < 1),^98^ resulting in also in increased stiffness of C-A-S-H gel.^64^

The crystallinity, nano and microstructure, and water content of the C-A-S-H phase differ from C–S–H.^99^ Al typically promotes the amorphous character of the gel^100−102^ and increases the interlayer spacing.^93,100^ Moreover, the presence of Al appears to influence the size and morphology of the C-A-S-H gel. In synthetic C-A-S-H gels prepared through precipitation from solution, a predominant morphology known as “nanofoils” has been observed, characterized by a compacted, foil-like microstructure.^103^ High Al content (Al/Si ratio of 0.20) results in thicker, larger, and more compact foils.^102,103^

**Figure 16:**
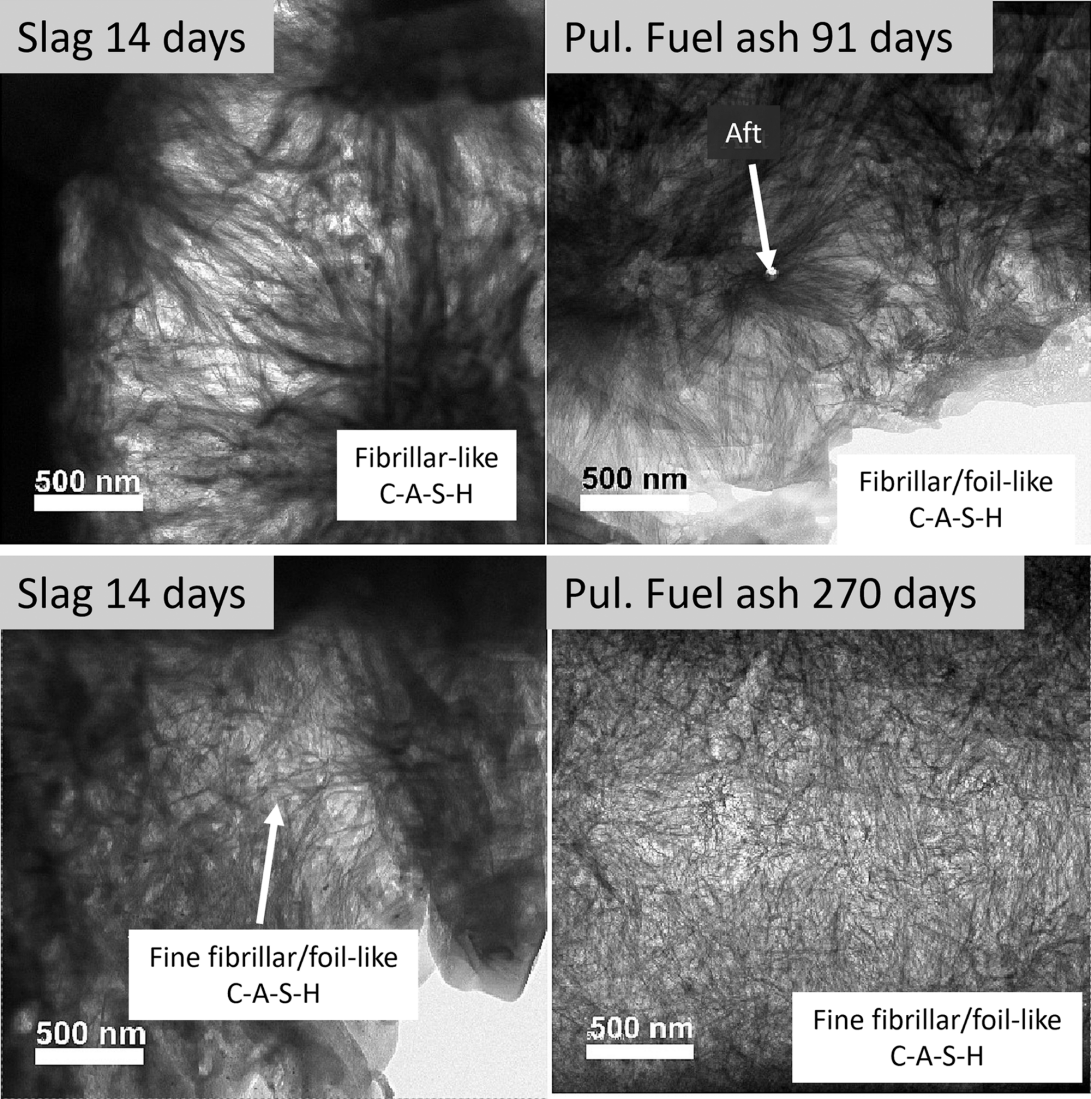
Bright-field TEM images that show the morphology of C-A-S-H in different samples: slag-containing blends at 14 and 28 days (left side) and pulverized fuel ash at 91 and 270 days. Reproduced with permission from ref (107) under a 4.0 Creative Commons Attribution License (CC BY 4.0 DEED). Copyright 2023 Elsevier. https://creativecommons.org/licenses/by/4.0/.

The microstructure of the hydration products in blended cements pastes has also evidenced differences between C-A-S-H and C–S–H.^104^ While typical “fibrillar-like” morphologies are observed for the outer product C–S–H gel in either C_3_S or PC,^53,104,105^ in cement-blended slag, the outer product C-A-S-H displays “foil-like” morphologies.^105,106^ These alterations in morphology observed in the pastes have also been associated, similar to the synthetic system,^103^ with changes in the composition of C-A-S-H, such as an increase in the Si/Ca and Al/Ca ratios, longer mean chain length of the aluminosilicates,^105^ and changes in the pore solution chemistry.^106^ Interestingly, the foil-like C-A-S-H gel in cements incorporating pulverized fuel ash or slag does not form right from the beginning of hydration (Figure 16). Instead, it undergoes a transformation from an initially fibrillar-like gel (characterized by low Si/Ca and Al/Ca ratios) into a foil-like morphology (associated with high Si/Ca and Al/Ca ratios). This conversion occurs relatively quickly, within 14–28 days for slag-containing blends, and more slowly, spanning 91–270 days, in pastes containing pulverized fuel ash.^107^ This change in morphology is likely a key factor contributing to the enhanced durability observed in systems containing slags,^104^ by reducing the permeability of the cement matrix.

Researchers have also examined C-A-S-H gel formation in the presence of calcined clays because of the increasing interest in limestone calcined clay cement (LC^3^).^108^ Their findings led to the conclusion that the presence of calcined clay does not significantly alter the morphology of C-A-S-H (fibrillar morphologies) compared to noncontaining clay blends.^109^ However, it should be mentioned that the samples were only cured for 28 days. It might be possible that the transformation into a foil-like C-A-S-H structure occurs at later stages, as observed in the previously described investigations by Zhu and Richardson.^107^

Due to the significance of blended cements, it is necessary to conduct additional research to identify the essential features, such as size, shape, or surface charge, of the primary building blocks that form when Al is incorporated into the C–S–H structure. We already have fundamental information at the molecular scale, such as the increase in the mean chain length (MCL) and the formation of more interconnected structures as the Ca/Si ratio decreases in C-A-S-H. However, there is a notable gap in the fundamental knowledge concerning the early crystallization of C-A-S-H and the effect of additives on it, which has received much less attention than that of C–S–H. This information can be utilized to accelerate the formation of C-A-S-H in these less reactive systems, ultimately reducing their setting time.

Considering the challenges outlined in the previous section regarding the exploration of the growth of cement hydrates, computational approaches should also be directed toward the investigation of the C-A-S-H system. Most existing studies have been atomistic, providing insights into differences between both hydrates at the smallest scale. In terms of large-scale studies (coarse-grained models), research has been predominantly centered on the assembly of C–S–H platelets,^69^ and thus, it is essential to broaden this focus to explore the primary unit of C-A-S-H and its subsequent assemblies.

### 2.4 Mesoscale of Cement Paste

**Figure 17:**
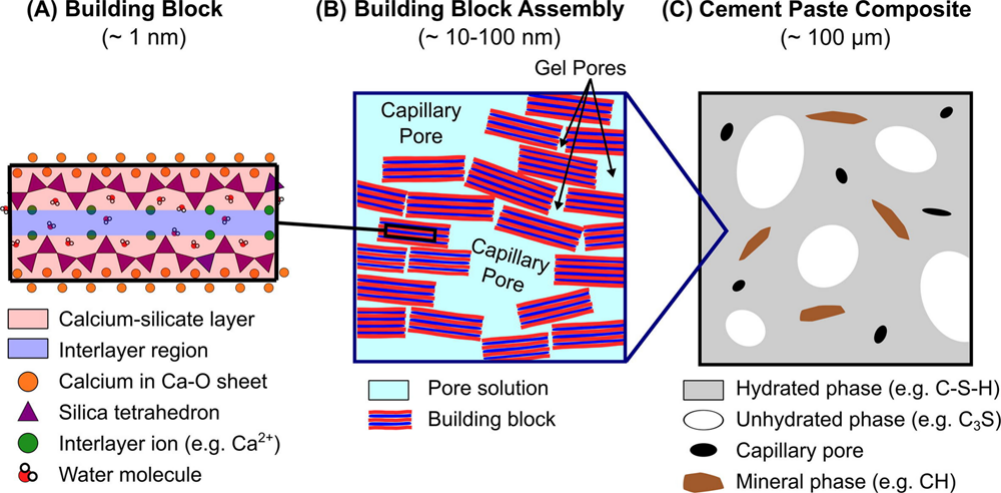
Multiscale nature of cement-based materials. Reproduced with permission from ref (111). Copyright 2016 Elsevier.

The mesoscale for a particular system must be defined in terms of the specific material and the property of interest.^110^ Cement-based materials are complex multiphase systems where structures of multiple length scales coexist and evolve. The properties that control hydration, strength, fracture resistance, durability, creep, shrinkage, aging, and resistance to chemical attack of cement pastes find their origin in different lengths and time scales. Therefore, a multiscale approach must be adopted when referring to the mesoscale of cement (Table 1),^111^ to connect the lower length scale entities with the macroscopic properties of cement paste. As mentioned previously, the smallest building blocks in the colloidal model are the approximately 5 nm C–S–H nanoparticles (Figure 17a) that aggregate, forming a disordered material with a hierarchical pore network ranging from nanometers to millimeters (Figure 17b).^54^ As in the case of most materials, the porosity of cement paste significantly influences its performance, for example, in terms of strength, shrinkage, creep, permeability, and ion diffusivity. Beyond the total porosity or the gel-space ratio, the characteristics of the pore network, such as size, shape, distribution, and connectivity, dictate the macroscopic properties of the final material.

**Table 1:** Definition of the Representative Multiscale Elements in Cement Paste and the Relationship between Key Features of a Material Microstructure and the Properties of Cement Paste (Adapted from Ref (111))

Length scale	Description	Key features	Durability properties
Building block (∼1 nm)	Quasi-layered nanoscale structure of hydrated gels	Ionic-covalent bonds and interlayer water interfaces	Unit processes, chemical reactions
Building block assembly (∼10–100 nm)	Building blocks arrange to form particulates of various sizes and shape	Gel and capillary pores, charged pore solution, particle packing and density	Shrinkage, creep, setting and aging, chemical attack
Cement paste composite (∼100 μm)	Continuous hydrated matrix with randomly dispersed inclusions (voids, unreacted materials, secondary products)	Volume fraction, position, and morphology of phases, interfaces, pore network, microcracks	Elastic stiffness, ultimate strength, fracture toughness

Powers first determined the structure–property relationships of cementitious materials.^112^ He established that the relationship between the gel-space ratio (*X*) and the strength of concrete (σ_c_) could be expressed empirically as follows (eq 1):
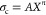
1“*A*” and “*n*” are distinct constants for each mix and cannot be calculated or predicted, which is a major shortcoming. This approximation promoted the concept of creating ultrahigh-strength cement-based materials with minimal porosity by optimizing the packing of the cement grains in the starting mix.^113^ However, as early as the 1970s, it was suggested that the relationship between compressive strength and porosity was much more complex and unlikely to be described by this simple empirical law.^114^ An illustrative example (among many) that demonstrates the risk of relying on simplified empirical laws based on macroscopic values to calculate macroscopic material properties was presented by Thomas et al.^30^ They investigated the hydration of different types of cements containing a small mass fraction of C–S–H seed particles in the mixing water. Although typical macroscopic values characterizing hydration progress were nearly the same for seeded and unseeded pastes (i.e., degree of reaction, C–S–H content, total porosities, and gel–space ratios), the compressive strength was lower for unseeded samples. These differences cannot be understood with accepted engineering equations, such as eq 1, which rely on macroscopic variables like gel-space ratio. The main variation between both samples was only the pore network of the paste. Microstructural analysis by electron microscopy showed that, with seeds, the pore size distribution of the cement paste is finer than in the unseeded sample.^30^ This underscores the importance of implementing a nano- and microscale approach to establish the appropriate links between the structure of the material at different scales and the related engineering properties.

The macroscale mechanical properties are also governed by the response of the multiphase paste at larger length scales where the binder phase (C–S–H) coexists with randomly dispersed voids, secondary reaction products, and unreacted clinker phases (Figure 17c).^111^ Similar difficulties are encountered for other properties like creep and drying shrinkage. While the permeability of the material mainly governs the resistance to chemical attack and degradation, deformation and shrinkage mechanisms are more complex and depend primarily on the presence and distribution of water in the pore network.^115^ In conclusion, the properties of cementitious materials cannot be linked to a single length scale or structural element and, analogous to biomineral composites,^116^ distinct features at multiple length scales must be considered to develop scientifically grounded models that accurately predict macroscopic properties, thereby, replacing empirical equations.

## 3 Understanding and Controlling C–S–H Nucleation

### 3.1 Nucleation

Nucleation represents the initial stage in the creation of a new thermodynamic phase and is crucial to explore when trying to understand the formation and characteristics of a solid phase. In principle, controlling nucleation allows to govern the shape, structure, and inherent properties of the resultant crystalline material, and therefore, substantial dedication has been directed toward understanding this process for almost 150 years, given its importance for academia and industry.

The first description of nucleation dates back to 1876 in the work of Gibbs.^117,118^ Subsequent advancements were made by Vollmer and Weber^119,120^ and Becker and Döring, who formulated the classical crystallization theory (CNT) for nucleation from supersaturated vapor using continuum thermodynamics.^8^ Nucleation is a random process, which implies that it occurs with the same probability and independently in each volume element of a chemically homogeneous medium. It is defined as the first irreversible formation of a nucleus of the new (equilibrium) phase, and it is required to bring the system to an unstable thermodynamic state temporarily.

For nucleation in solution, the conversion of the crystallizing species (i.e., atoms, ions, or molecules) to a solid is driven by the excess in free energy of the initial solution phase compared with combined free energies of the crystalline and the final solution phase. This surplus in free energy is expressed as the change in chemical potential Δμ per individual crystallizing species during the transition from the solution phase to the solid phase (eq 2).
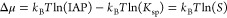
2with *k*_B_ = Boltzmann constant, *T* = temperature, IAP = ion activity product of the reactants, *K*_sp_ = activity product of those reactants in equilibrium with the solid phase (i.e., solubility), ln(*S*) = supersaturation.

**Figure 18:**
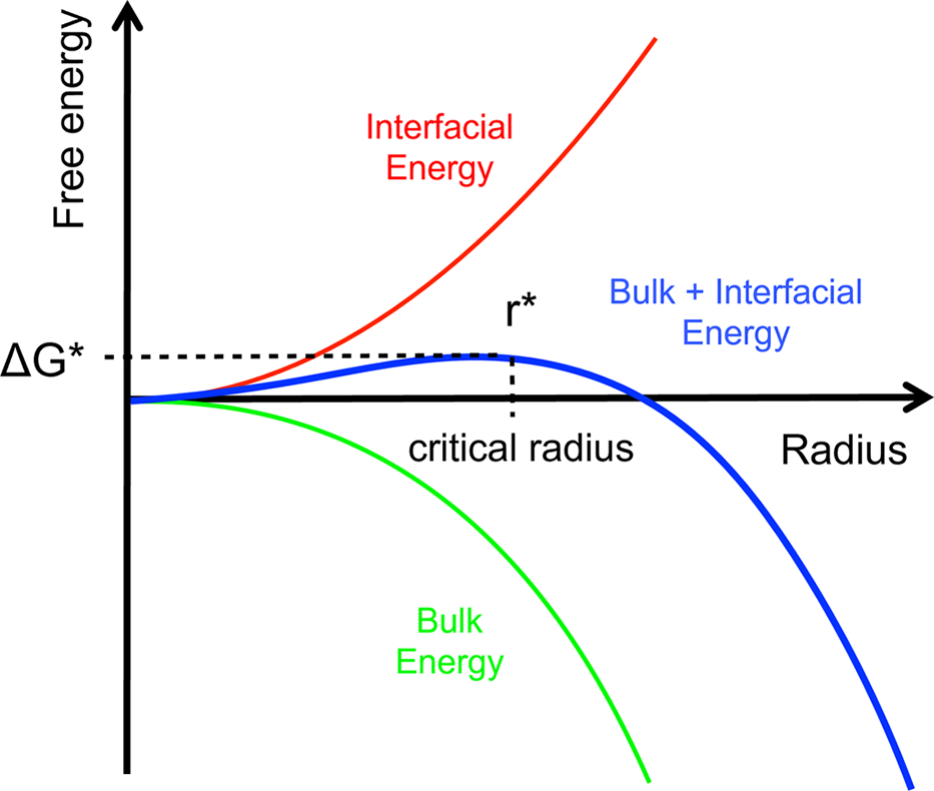
Schematic representation showing the dependence of the nucleation barrier ΔG* on the radius *r* according to classical nucleation theory. Reproduced with permission from ref (121). Copyright 2011 Elsevier.

If ln(*S*) respect to any phase is positive, even at infinitesimal levels, nucleation must occur from a purely thermodynamic point of view. However, the formation of the solid is hindered by a free-energy barrier, dependent on the interfacial free energy of the nucleus and supersaturation. Considering a spherical nucleus with the same structure as the final crystalline phase, the interplay between the energy gained by the formation of the crystal lattice (blue curve) and the energy cost caused by the formation of a new interface (red curve) will determine if a nucleus grows or dissolves (Figure 18). These two contributions are directly proportional to the number of monomers transitioning from solution to the solid phase (scaling with the particle’s volume) and the surface area of the emerging nucleus (scaling with the particle’s surface), respectively (eq 3). Thus, the unfavorable surface contribution dominates Δ*G* at small sizes, leading to nucleus dissolution, and the favorable bulk term prevails at large sizes, resulting in unlimited growth.
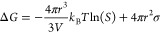
3with Δ*G* = free enthalpy, *r* = particle radius, *V* = volume of single molecule and σ is the specific surface energy.

The addition of the interface and volume energy contributions yields the blue curve depicted in Figure 18, describing the size-dependent free energy of the species. Notably, this curve exhibits a maximum corresponding to the so-called critical radius (r*), which entails an energy barrier to nucleation, expressed as ΔG*. The critical radius represents the minimum size that must be reached, beyond which the formed species can continue to grow further without disintegrating, as is the case below the critical size. ΔG* is obtained for a spherical nucleus by maximizing eq 3 and setting the derivative of Δ*G* equal to 0. This yields r* = 2σ/*k*_B_*T* ln(*S*) and ΔG* equal to (eq 4):
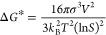
4The energy barrier ΔG* can be implemented to express the rate of nucleation *J* (number of nuclei per unit of time per unit of volume) in the terms of the classical rate equation of a chemical reaction (eq 5).^122^
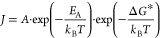
5

*A* is the pre-exponential factor, dependent on the characteristics of the formed phase. Next, we find two different types of barriers. The first exponent, which is difficult to quantity, represents the kinetic barrier with activation energy *E*_A_. It is associated with atomic processes essential for the formation of the nucleus, such as desolvation, bonding, or structural reorganization of the monomers. The second exponent, as derived above, constitutes the thermodynamic barrier dependent on interfacial tension, volume of the nucleus, temperature, and supersaturation.^122^

In the case of heterogeneous nucleation on external substrates, the energy barrier for nucleation is reduced. This reduction is attributed to the different interfacial energy between the crystallite and the solid substrate compared to that between the crystallite and the solution. The equations for homogeneous nucleation with CNTs remain unchanged. However, the interfacial tension (σ) must be adapted to an “effective” value, noted as σ_het_. This adjustment accounts for the interfacial energies between crystal–substrate, crystal–liquid, and liquid–substrate. This approach allows for the determination of the energy barrier for heterogeneous nucleation (Δ*G*_het_*) by multiplying the nucleation barrier for homogeneous nucleation with a factor Φ (eq 6),^123^ which is dependent on the contact angle θ and is less than the unity (eq 7). Hence, by controlling the wettability of the surface, one can alter the free energy barrier for heterogeneous nucleation and, subsequently, the nucleation rate.
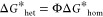
6
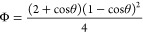
7Based on the above-discussed, one can theoretically predict nucleation rates. However, the predictive power is limited, with discrepancies in calculated nucleation rates spanning orders of magnitude. This demonstrates that CNT can only describe nucleation in real systems to a limited extent, so this theory is restricted to qualitative predictions. The limitations of CNT arise from its foundational assumptions, which include a spherical nucleus, a sharp interface, and reliance on the capillary assumption. This assumption presumes that the properties of the nucleus are bulk properties, enabling the application of continuum thermodynamics.

In the atomic/molecular size regime, the parameter σ becomes size-dependent, and thus, assigning the appropriate interfacial tension poses a problem. Additionally, the curvature of the nucleus varies with size, leading to an increased pressure Δ*p* (Laplace pressure) as particle size decreases, according to the Young–Laplace equation (eq 8). The force generated in this manner, pointing outward from the nucleus, acts in opposition to the surface tension and represents an additional contribution, which is not considered in CNT.
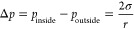
8Furthermore, the nucleus typically consists of only a few building units, likely possessing atomic/molecular characteristics rather than the bulk properties considered in CNT. In this sense, fundamental problems will arise, as exemplified by the varying solubility (*K*_SP_) of different polymorphs with particle size.^124^ Thus, supersaturation values calculated using bulk *K*_SP_ might not be representative of the system. The last point we wanted to highlight, which leads us to the next section, is that the nucleated phase often has a different structure than the final crystallization product. This makes it difficult to assign a meaningful *K*_SP_ to describe the nucleation process in real systems. Despite its shortcomings, CNT remains a valuable and simple tool for describing nucleation by using only the surface and bulk-free energies of the formed nuclei in solution. However, as we will explore in the next section, experimental observations have indicated that this classical model often fails to describe real systems accurately.

### 3.2 Multistep Nucleation Processes

CNT hypothesizes that the nucleation and the growth of a nucleus occur only via the addition of monomers to the nascent particle. However, modern experimental methods have revealed that the formation of crystals often involves the attachment of considerably larger entities that expand from ion complexes to fully formed nanoparticles.^125^ These observations, which cannot be reconciled with CNT, have laid the foundation for nonclassical crystallization pathways, encompassing nucleation and growth mechanisms that do not proceed exclusively by atomic/molecular building units.^126^ The intermediate stages between the ions in solution and the thermodynamically stable phase provide additional ways to control its formation and properties, e.g., by adjusting experimental parameters or adding additives that can influence different steps of this process.^127−129^

**Figure 19:**
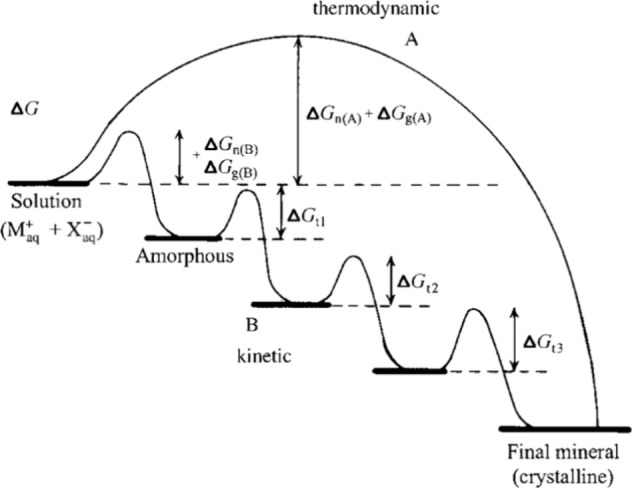
Crystallization pathways under thermodynamic and kinetic control. Whether a system follows a one-step route to the final mineral phase (pathway A) or proceeds by sequential precipitation (pathway B), depends on the free energy of activation (Δ*G*) associated with nucleation (n), growth (g), and phase transformation (t). Amorphous phases are common under kinetic conditions. Figure reproduced with permission from ref (130). Copyright 2003 John Wiley & Sons.

Another crucial consideration is that the initial phase formed may not necessarily be the most stable, leading to the emergence of metastable phases and their subsequent transformation into more stable counterparts. These multistep pathways would be favorable when there are kinetic restraints on the formation of the thermodynamically stable phase from the dissolved monomers (pathway A in Figure 19). The kinetic route (pathway B in Figure 19) reveals the possible existence of multiple intermediate stages, each characterized by significantly smaller activation energies Δ*G* compared to the primary barrier in the direct thermodynamic pathway, leading to the most stable mineral product (Figure 19).

This is known as the Ostwald rule of stages,^131^ which states that the emerging phase has the smallest energy barrier relative to the preceding one.^132^ Following this pathway, if we have a highly supersaturated solution where the IAP surpasses the *K*_SP_ of all the possible phases, every one of them should nucleate but at different rates (eq 5). Supersaturation will be the highest with respect to the most stable phase; however, metastable phases, especially amorphous and hydrated phases, are likely to have lower interfacial free energy and kinetic prefactor values,^122^ which makes their formation rate higher. Once a more stable phase nucleates, the previous one will dissolve as the IAP drops below its respective *K*_SP_. This process will proceed until the most stable phase appears, as it is not subject to transformation.

In kinetically controlled crystallization, it is common that an initial amorphous phase forms, which might be characterized by nonstoichiometry, strong hydration, and instability. These crystallization pathways are frequently operative in biomineralization, where the presence of amorphous precursor phases is crucial to regulate the formation process and create unique structures adapted to their specific functions.^133^ For instance, during the mineralization of sea urchin spicules, amorphous calcium carbonate (ACC) nanoparticles form, aggregate, and convert into a calcite single crystal.^134^

**Figure 20:**
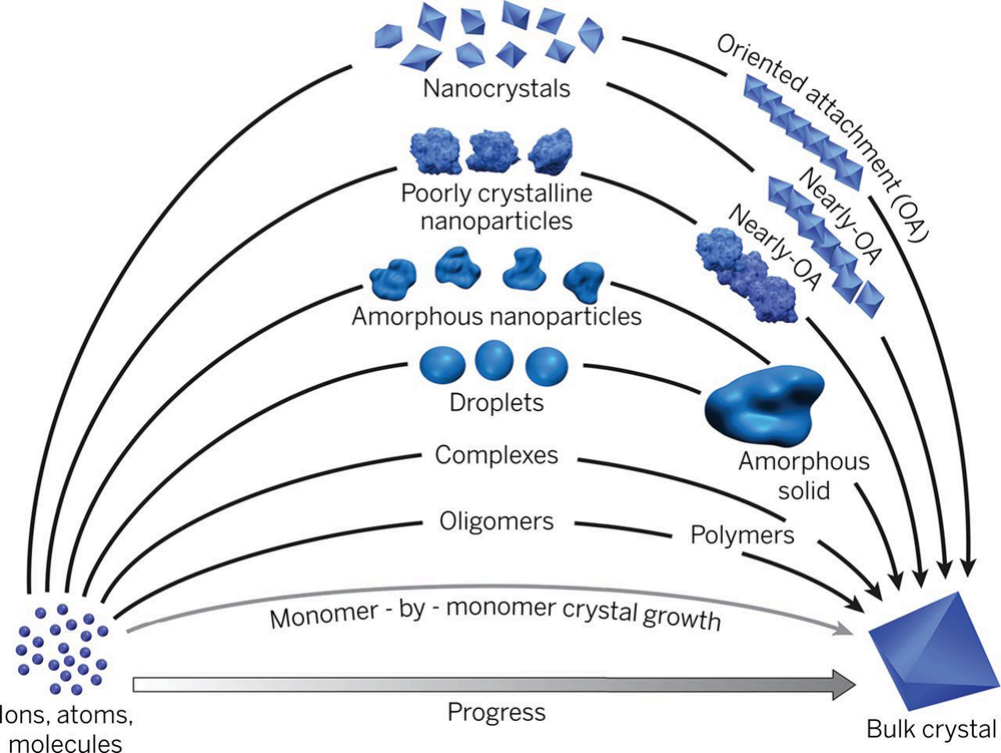
Pathways to crystallization by particle attachment. In contrast to monomer-by-monomer addition as envisioned in classical models of crystal growth (gray curve), particle attachment crystallization occurs by adding higher-order species ranging from multi-ion complexes to fully formed nanocrystals. The final faceted bulk crystal is a schematic representation of a final single-crystal state. The final crystal can have more complex morphologies, including spheroidal. Reproduced with permission from ref (125). Copyright 2015 American Association for the Advancement of Science.

Considering the competition between thermodynamic and kinetic multistep pathways shown in Figure 19, the nonclassical multistep routes are favored when the sum of the energy barriers in a multistep pathway is less than the single energy barrier of the thermodynamic pathway considered in CNT. These precursor phases, which transform into more stable ones, can include multi-ion complexes,^135−138^ dense liquids,^139,140^ solid amorphous phases,^133,141^ or even crystalline nanoparticles.^142^ As mentioned above, apart from nucleation, crystal growth can follow nonclassical pathways that involve attachment processes of various entities in the formation of crystalline material (Figure 20).^125^ Numerous experimental observations in various systems suggest that crystals can grow not only through the addition of atoms/ions/molecules, according to classical models, but also through the attachment of particles.^143^ This will be explored in detail in Section 7.

**Figure 21:**
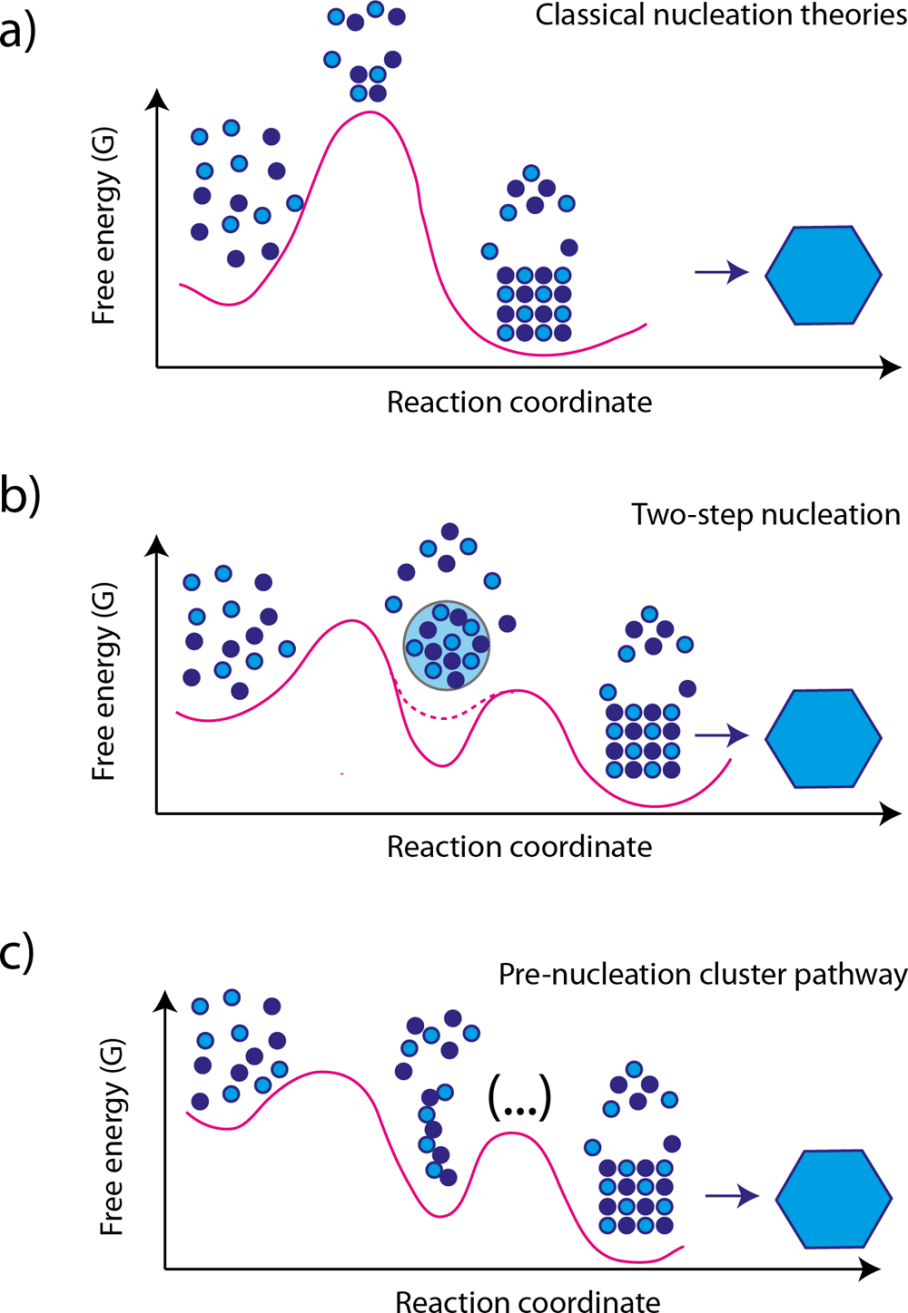
Mechanisms for crystal nucleation: a) classical nucleation theory, b) two-step nucleation mechanism, and c) prenucleation clusters concept.

Two main mechanisms for the multistep nucleation processes have been suggested: two-step nucleation and prenucleation clusters pathway (Figure 21). In the two-step nucleation mechanism, the intermediate formed is a stable/metastable dense liquid-like phase,^144^ whereas prenucleation clusters are solute entities characterized by thermodynamic stability, in both cases, with respect to the initial solution.^145^ In the following, we present these two distinct multistep nucleation pathways.

#### 3.2.1 Two-Step Nucleation

The two-step nucleation mechanism was initially proposed theoretically^146^ and subsequently observed in proteins,^147,148^ organic molecules,^149^ and also in inorganic substances.^140,139^ In this mechanism, a dense liquid phase is formed first through phase separation (i.e., phase boundary exists between the two phases), followed by crystal nucleation within the dense liquid phases in a second step. The formation of a metastable dense liquid phase reduces the barrier for nucleation of the solid phase due to an increased supersaturation level in the intermediate. The nucleation of the solid within the intermediate adheres to the principles of CNT, characterizing two-step nucleation as a classical mechanism.^137^

Through the application of dynamic light scattering and taking advantage of proteins’ macromolecular size, researchers directly observed the two-step nucleation mechanism by tracking cluster evolution.^150^ In this mechanism, the dense droplet is considered metastable with respect to the crystalline state but can be either stable or metastable with respect to the parent liquid (Figure 21b). Dense liquid droplet sizes can vary from several tens to several hundreds of nanometers but are, by far, the minor phase with a volume fraction well below 10^–3^ of the solution. Because of the high density of the solution inside the droplets, the nucleation rate increases, and the fluid layer acts as a buffer between the original fluid and the growing crystal. Therefore, by adjusting the solvent conditions, nucleation can be selectively enhanced without increasing the crystal growth rate.^151^ It was found that not all droplets nucleate crystals and some of the droplets dissolve again. Thus, the overall nucleation rate is also determined by the nucleation rates of the crystals in the droplets.^152^

**Figure 22:**
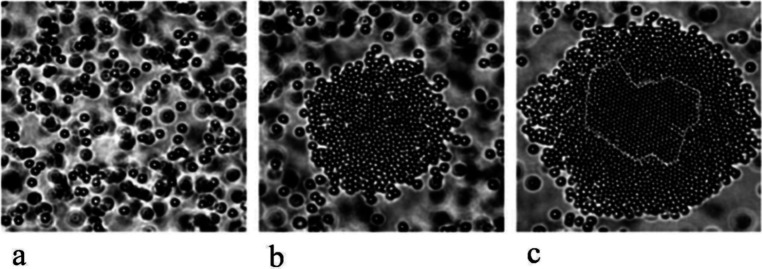
Nonclassical pathway to nucleation in the colloidal model system (polystyrene particles of diameter 0.99 μm) via amorphous dense phase (a) dilute mother phase (b) 2D amorphous dense droplets formed on glass surface (c) crystal nucleation from the amorphous phase. Reproduced with permission from ref (155). Copyright 2007 American Chemical Society.

Further evidence for the two-step nucleation mechanism was obtained by investigating colloidal model particles, which are large enough to be in situ observable by a light microscope.^153,154^ An example is given in Figure 22, where all individual phases in the two-step nucleation mechanism could be observed. Figure 22b shows that a highly concentrated phase showing relatively high local order serves as the precursor for the nucleation of the ordered (crystalline) phase.^155^ This mechanism is also consistent with a recent NMR study conducted by our group on the nucleation of Ibuprofen, a low molecular weight molecule. We revealed the formation of a dense liquid phase, as proven by ^1^H NMR PFG-STE self-diffusion experiments, but with a remarkably small intermolecular distance between the individual molecules (determined by ^1^H–^1^H NOESY NMR). This distance is almost as small as that in the crystal, and the generation of structural order follows densification in the dense liquid phase.^149^

Two-step nucleation has also been documented for many inorganic systems.^156^ Of particular interest are calcium carbonate^157^ and calcium phosphate^158^ biominerals, where the initial formation of the amorphous solid or liquid phase precedes the appearance of the crystalline phases.^133^ Similar results have also been reported for relevant construction materials such as calcium sulfate,^159^ portlandite,^160^ and more recently for calcium silicate hydrate (C–S–H).^9^ Nonetheless, there is little evidence that the respective crystalline phases are formed directly from the amorphous precursor rather than via an independent nucleation event.

#### 3.2.2 Prenucleation Cluster Pathway

Besides the two-step nucleation, the PNC pathway emerged as an important multistep pathway in nucleation, first described in a seminal paper by Gebauer et al.^145^ The essential difference between the PNC pathway and CNT is that nucleation is not driven by a size-dependent interplay between surface and bulk energies but by the dynamics of the involved species as will be further detailed below.

**Figure 23:**
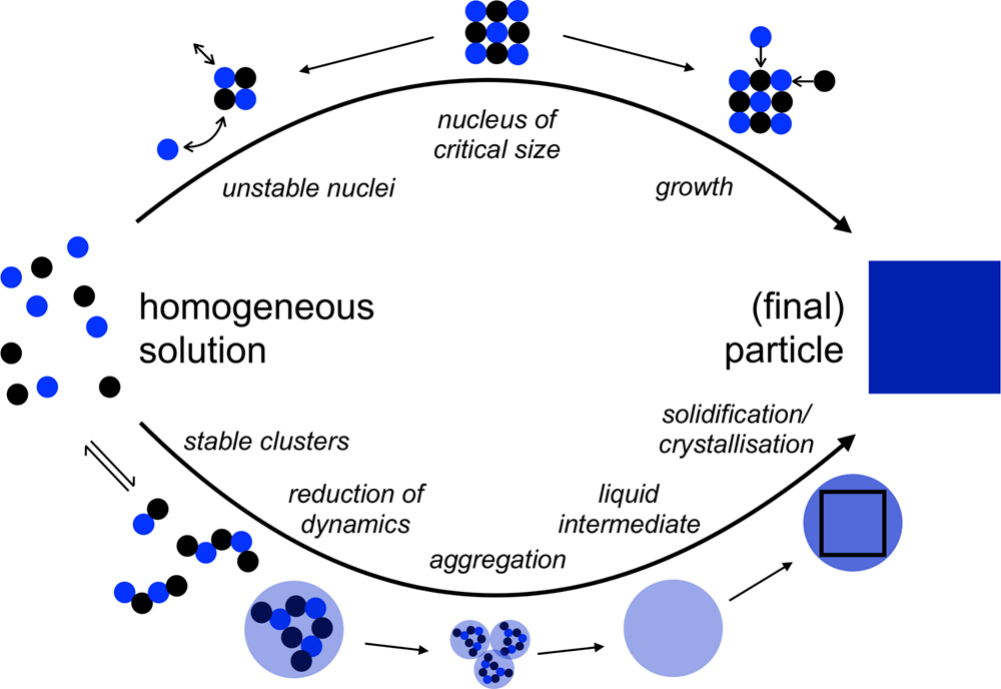
CNT (upper image part) vs the prenucleation cluster pathway (lower part). The PNC pathway involves PNCs, which reduce their dynamics, leading to the nucleation of nanodroplets. The nanodroplets aggregate, coalesce, and form liquid droplets. These can eliminate further water to form amorphous particles in which the final crystal starts to form. Reproduced from ref (128) under a 4.0 Creative Commons Attribution License (CC BY 4.0 DEED). https://creativecommons.org/licenses/by/4.0/.

This pathway has been intensively investigated for CaCO_3_, making it the system with the most reliably established experimental data. Figure 23 shows that multiple species are present in the PNC pathway, in contrast to CNT. Besides the multiple intermediates involved in this pathway, in which PNCs are the central species, what is immediately apparent from Figure 23 is that water plays a very important role, which is not considered in CNT.^135,136,138^ When comparing the energy diagrams of the CNT and the PNC pathway, a remarkable difference becomes clear (Figure 21). CNT shows one energy maximum corresponding to the metastable critical nucleus, while the PNC pathway presents a negligible energy barrier toward the formation of PNCs, so they spontaneously form and are in equilibrium with the dissolved ions.

**Figure 24:**
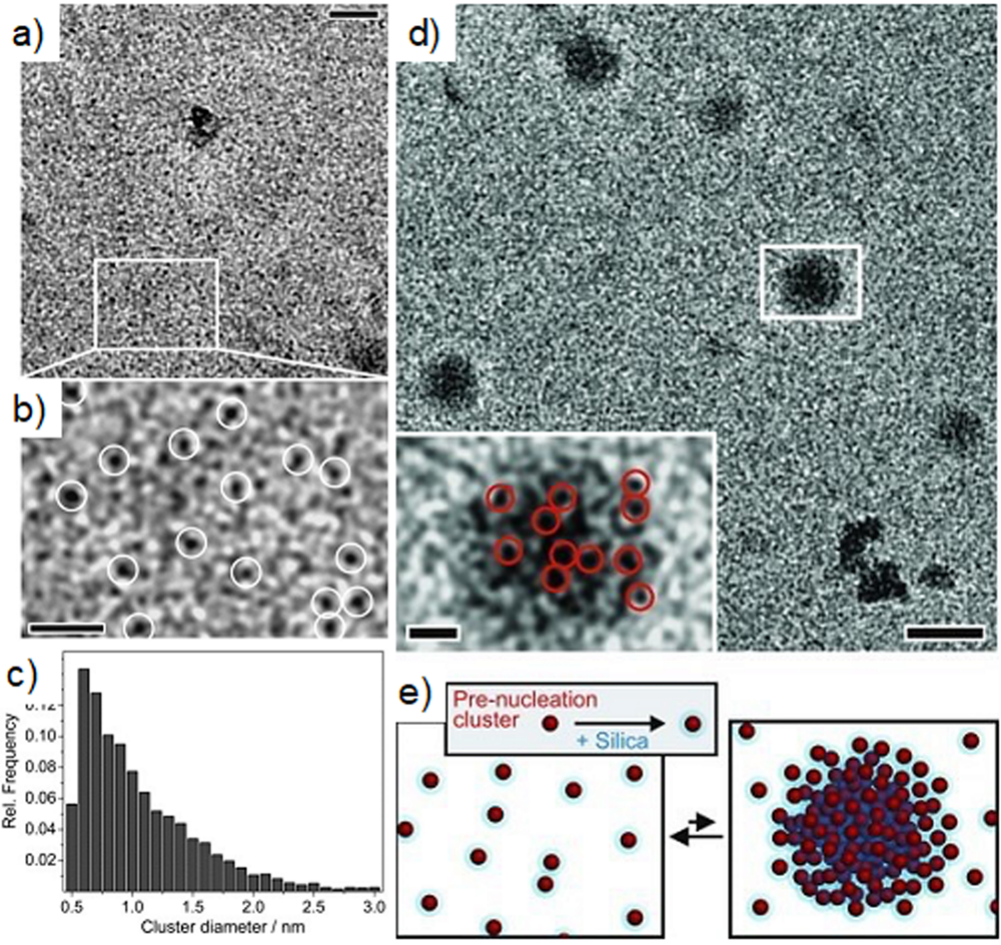
Nanoclusters in silica-rich supersaturated solutions of calcium carbonate. a) Cryo-TEM image of a sample containing 5 mM CaCO_3_ and 3720 ppm of SiO_2_ at pH 11, quenched in liquid ethane 3 min after mixing reagents. Scale bar: 20 nm. b) Enlargement of the area delimited by the white rectangle in (a), illustrating the presence of individual nanoclusters (highlighted by white circles). Scale bar: 10 nm. c) Size distribution diagram derived from cryo-TEM image for individual cluster species. The apparent average cluster diameter is 1.1 ± 0.6 nm. Dynamic cluster aggregation. d) Lower-magnification cryo-TEM image of a sample vitrified after 3 min, showing larger objects next to myriad single cluster species. Scale bar: 50 nm. Inset: Close-up view of the marked area, suggesting that the dark domains are loose aggregates of clusters (individuals are highlighted by red circles). Scale bar: 10 nm. e) Schematic drawing visualizing cluster agglomeration under the influence of silica at pH 11. Note that structures are not drawn to scale and that the blue halo in the scheme is meant to generally indicate the presence of silicate species in the periphery of prenucleation clusters, rather than necessarily representing a skin of silica around them. Reproduced with permission from ref (162). Copyright 2012 John Wiley & Sons.

PNCs can be considered highly dynamic polycondensation polymers of ion pairs with an average size of 1–2 nm.^136,161^ They are solutes and, therefore, do not have a boundary with the solution. Kellermeier and colleagues stabilized the PNCs and their aggregates by encapsulation in silica. This facilitated their observation via electron microscopy and enabled them to determine the particle sizes (Figure 24).^162^

The nucleation toward liquid droplets can take place if the binodal limit is passed, which was found to be the solubility product of amorphous CaCO_3_.^163^ Terahertz spectroscopy following the water dynamics during the formation of CaCO_3_ revealed that the water mobility increases due to water release from the ion hydration shells upon PNC formation until the binodal is reached.^163^ The water release from the ion hydration shell was found to be the entropic driving force for the formation of PNCs by calorimetric and temperature-dependent titration experiments.^164^ A further evidence for the important role of water in the formation of PNCs could be found by the influence of cosmotropes and chaotropes species on the PNCs. These substances modify the water structure within the solution, thereby influencing the hydration of PNCs and leading to changes in their stability.^165,166^

Once the binodal limit is overcome and liquid nanodroplets nucleated, the water mobility decreases due to the reduced motion of the molecules in the dense nanodroplets compared to the surrounding diluted solution phase.^163^ Simulations have shown that the decrease in the PNC dynamics upon the nucleation of the liquid nanodroplets can be understood as an increase in the coordination number relative to the initial chain-like structures.^136,167^ Although the calcium carbonate nanodroplets are intrinsically stabilized by bicarbonate ions at near neutral pH, as demonstrated by NMR,^140^ the nucleated liquid nanodroplets are not colloidally stable. With increasing concentration, they start to aggregate to form larger droplets, which subsequently transform into amorphous particles with the further release of water.

**Figure 25:**
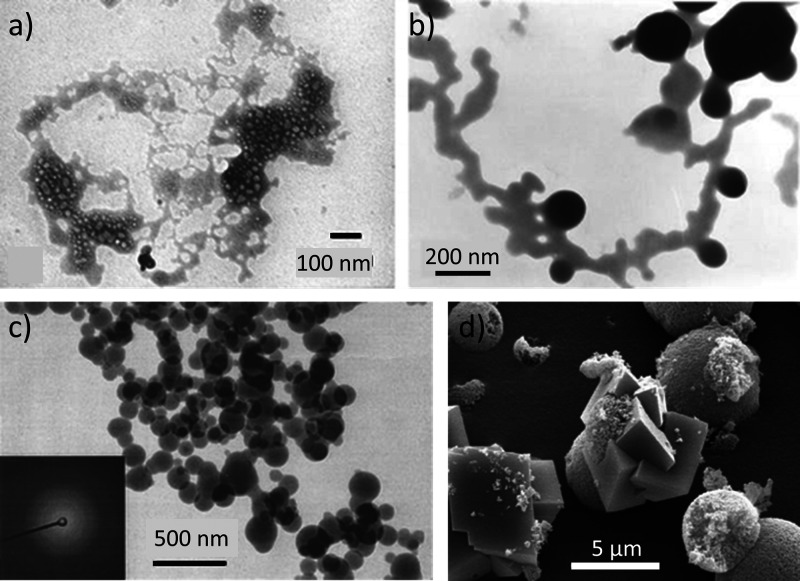
Early stages of CaCO_3_ precipitation by rapid mixing of reactants, without additives. (a) Cryo-TEM shows an “emulsion-like” precipitate at 100 ms. (b) After minutes, the densification and breakdown into nanoparticles, which, as shown in part (c), are nondiffracting ACC. (d) SEM of the products at 60 min shows vaterite spheres and calcite rhombohedral. Reproduced with permission from ref (168). Copyright 2007 Royal Society of Chemistry.

The formation of liquid-like precipitates in the early stages of CaCO_3_ precipitation (milliseconds to minutes) was experimentally demonstrated using cryo-TEM and in situ X-ray microscopy Rieger et al., 15 years ago.^168^ It was suggested that the initial liquid-like products were similar to an emulsion (Figure 25a), which exhibited short-term stability in the absence of additives. This liquid-like phase appeared to dehydrate (Figure 25b) and convert to amorphous CaCO_3_ nanoparticles (Figure 25c), which subsequently aggregate to form micrometer-sized vaterite spheres. Finally, vaterite spheres transform to the most stable calcite rhombohedra via a dissolution-recrystallization process (Figure 25d). These findings highlight that the resultant crystal shape provides little insight into the formation mechanism, as a faceted crystal with an equilibrium-like morphology can form via a complex process involving several intermediate phases.

If ppm amounts of acidic polymers like poly(aspartic acid) or poly(acrylic acid) are added to the initial ion solutions,^140,168^ the liquid droplets are kinetically stabilized by the polymers, leading to the so-called Polymer Induced Liquid Precursors (PILPs),^139^ which can yield minerals with either elaborate or, as we saw earlier, entirely conventional shapes.^133^ PILPs, as discussed in Section 5, have been proposed as relevant precursors in biomineralization processes.^169^ Their liquid-like character facilitates the infiltration of nanoscale organic templates with mineral precursor phases,^170^ which are subsequently transformed into crystalline biomaterials such as calcium phosphate (bone) or calcium carbonate (nacre).^171−175^ The bioinspired approach of using liquid mineral precursor phases in materials synthesis offers some key two advantages. It allows efficient delivery of mineral precursors to inaccessible parts of a matrix, while the moldability of the liquid-like phase can generate nonequilibrium morphologies. Surprisingly, this strategy has not been widely explored in the field of materials chemistry. A recent review focused on the development of materials for hard tissue repair highlights that most of the studies are still in the proof-of-concept stage, and the real application of those materials is still far.^176^

In the context of utilizing liquid-like mineral phases for construction materials, our collaboration with the Gower group introduced the pioneering use of CaCO_3_ PILP as a cementing method for quartz sand.^177^ Nevertheless, it is important to note that this process was based on a continuous flow mineralization setup and proved relatively slow. Therefore, achieving industrial applicability would require several modifications toward upscaling. Recently, the Gebauer group has developed an easily scalable method that facilitates the synthesis and use of the CaCO_3_ liquid precursor on a large scale.^178^ This method will enable the application of CaCO_3_ PILP phases on larger scales, including uses in construction materials, such as cultural heritage restoration,^179^ production of molded CaCO_3_ mineral,^180^ and mineral coatings.^181^

Although the PNC pathway is still highly debated^137,182−184^ and a unification of classical and nonclassical mechanisms was suggested,^185^ the experimental evidence for the PNC pathway is steadily rising, with an increasing number of systems exhibiting its involvement in nucleation, including calcium phosphate,^186^ CoFe_2_O_4_,^187^ iron oxides,^188^ InAs^189^ but also organic molecules like amino acids.^190^ In the context of relevant phases for construction materials, multistep crystallization pathways involving PNCs have been demonstrated for several phases, and the number of identified cases continues to increase. Examples include calcium sulfate,^191^ portlandite,^160,192^ geopolymers (N-A-S-H gel),^193^ magnesium carbonate,^194^ magnesium silicate hydrate,^195^ magnesium phosphate,^196^ magnesium oxychloride.^197^

**Figure 26:**
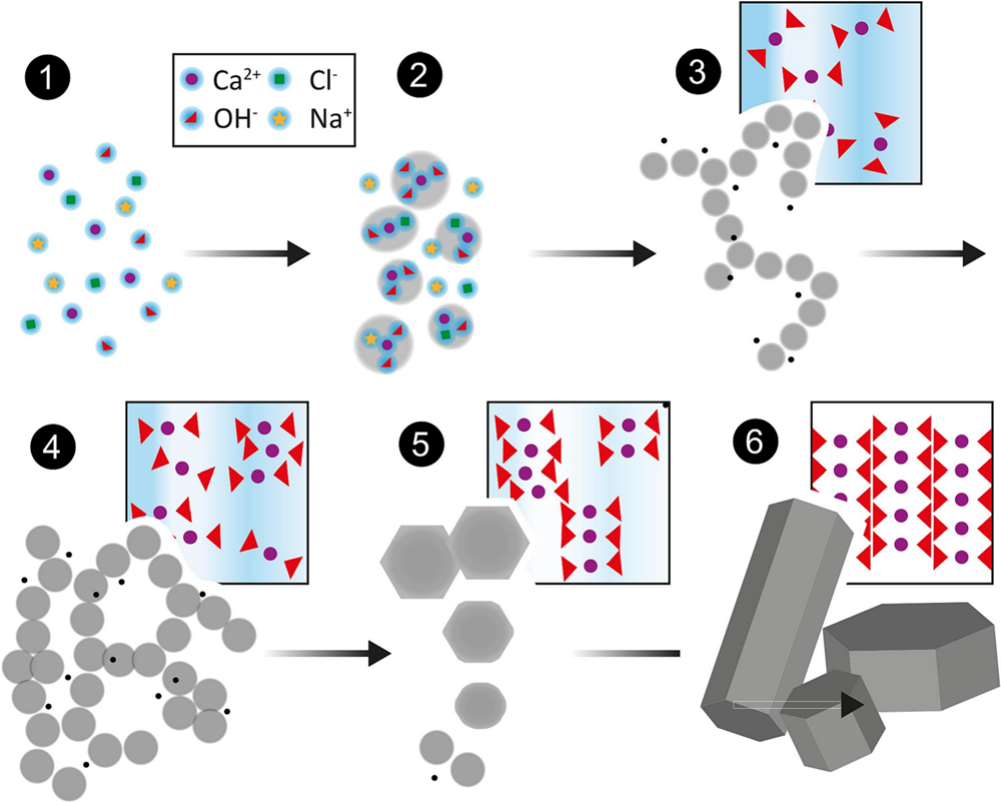
Schematic illustration of the sequential stages underlying the formation of crystalline calcium hydroxide from solution in the absence of any additives, as envisaged based on the data collected in ref (192). Stage 1: Single constituent (Ca^2+^ and OH^–^) and “spectator” (Na^+^ and Cl^–^) ions dissolved in water. Stage 2: Ion association into complexes (and clusters) of varying stoichiometry. Stage 3: Coalescence of ion complexes/clusters into larger entities (100–200 nm, presumably of initial liquid-like nature), which immediately aggregate into loose networks to minimize interfacial tension. At the same time, smaller species (1–4 nm, indicated by black dots) coexist, as shown by SAXS and AUC, and likely act as building units for the larger structures. Stage 4: Densification of the networks, concurrent growth, and progressive solidification of individual particles into amorphous calcium hydroxide while primary building units coexist. Stage 5: Structural reorganization within the aggregated ACH networks and incipient evolution of more ordered matter. Stage 6: Completion of amorphous-to-crystalline transformation and growth of portlandite crystals with regular hexagonal habits. Note that the structures in the different stages are not drawn to relative scale. Reproduced with permission from ref (192). Copyright 2023 Elsevier.

Concerning portlandite formation, which is a highly relevant phase during the hydration of PC, a nonclassical crystallization mechanism has been proposed based mainly on two recent studies.^160,192^ Both studies found that the formation of portlandite crystals can be described by a multistage process where transient species and metastable phases are participating. This mechanism involves the presence of nanoclusters (black dots in Figure 26), which coalesce to yield amorphous nanoparticles of presumably liquid nature. These amorphous globular entities further aggregate into networks, densify and dehydrate, and finally convert to hexagonal Ca(OH)_2_ crystals (Figure 26). The final particles showed nanogranular features of the crystal surfaces, confirming the particle-based growth mechanism.^192^ However, how amorphous particles can aggregate, adopting a ring-like hexagonal shape, has not yet been unveiled.^160^ In the presence of anionic additives, the pathway seems slightly affected. As Madeja observed, the amorphous phase experiences an extension in its lifespan, leading to a delay in the transition to crystalline calcium hydroxide. Additionally, the additives contribute to the formation of larger crystals with more developed {001} faces, most likely due to the preferential absorption of the anionic molecules onto the positively charged faces of the crystals.^160^ The additives seem to be occluded into the structure, modulating the mechanical properties of the resulting solid,^192^ which stands as a prominent strategy in the context of organic–inorganic hybrid cementitious materials.

The applicability of the nonclassical nucleation routes in describing C–S–H formation will be discussed in the next section.

### 3.3 C–S–H Nucleation

Investigating the nucleation of C–S–H experimentally in alite or PC systems is an overwhelming task due to the concurrence of many processes (already mentioned in Section 2.1), which hinders their isolation. Hence, simplified C–S–H nucleation scenarios must be used to gather fundamental knowledge regarding the early stages involved in C–S–H formation. Most available investigations dealing with C–S–H nucleation use computational approaches, and experimental approaches are scarce. Nevertheless, over the past few decades, certain pivotal studies have significantly contributed to understanding the synthetic formation of C–S–H.

Garrault-Gauffinet and co-workers presented the first study investigating the homogeneous and heterogeneous nucleation of C–S–H.^198^ In their work, they explored different supersaturations and determined the induction time for nucleation with conductivity measurements. By correlating the measured induction time with the degree of supersaturation with respect to C–S–H (calculated by a speciation model), the authors concluded that C–S–H nucleation could be interpreted within the framework of the classical nucleation theory (CNT). However, it is worth noting that the authors did not provide a detailed characterization of the obtained C–S–H precipitates. They concluded that at low supersaturation values, C–S–H nucleation was a heterogeneous process, while at higher values, homogeneous nucleation predominates. Furthermore, they also determined that the introduction of calcite and calcium silicate heterogeneous surfaces significantly reduced the free energy barrier for nucleation.^198^

**Figure 27:**
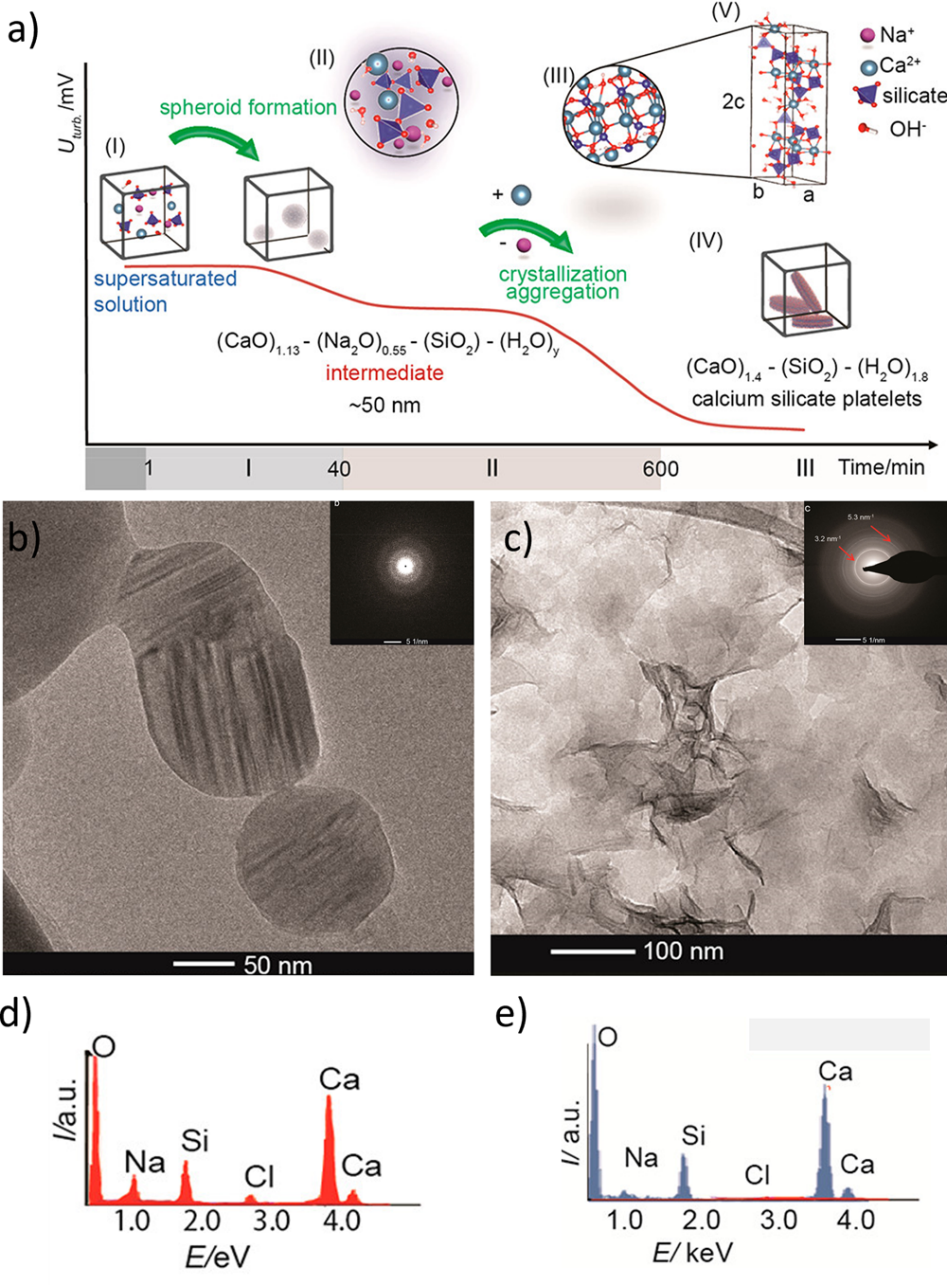
a) Two-step pathway proposed for C–S–H nucleation by ref (9). Four different stages are distinguished in the evolution of the transmittance of the solution (red curve) versus time and assigned to the emergent different species: (I) supersaturated solution, (II) liquid amorphous intermediate, (III) crystalline domains, and (IV) final β-C–S–H platelets. In (V), the model of the C–S–H structure is represented by 14 Å tobermorite. The first chemical step (first drop in the turbidity) has been assigned to the formation of the spheroid intermediates (size ca. 50 nm) rich in silicate dimers and calcium. The intermediate composition in silicate and calcium is like that of β-C–S–H with additional sodium ions and water molecules. The second drop (stage II to stage III) on the transmittance is related to the formation and aggregation of β-C–S–H crystallites. A Ca/Na exchange accompanies this step. b) Cryo-TEM image of the spheroids formed after 300 min (Inset corresponds to the SAED pattern of the spheroid). c) TEM micrograph of the collected solid phase (inset SAED pattern of the final product). d) EDX spectrum of the spheroid and e) EDX spectrum of the final C–S–H reaction product. Reproduced with permission from ref (9). Copyright 2018 American Chemical Society.

A more recent study by Krautwurst and co-workers revealed that the synthetic formation of C–S–H is the result of a complex two-step nucleation process.^9^ Employing time-resolved potentiometry and turbidimetry, dynamic light scattering (DLS), small-angle X-ray scattering (SAXS), and cryogenic transmission electron microscopy (cryo-TEM), the authors concluded that amorphous spheroids with a diameter of ca. 50 nm form first, which later crystallize to tobermorite type C–S–H. These spheroids seemingly result from the aggregation of charged silicate dimers surrounded by Ca^2+^. Cryo-TEM and scattering (SLS, DLS, SAXS) data suggest that the spheroids do not serve as a substrate for heterogeneous nucleation. In the second stage of this process, the spheroids aggregate and undergo changes in stoichiometry, characterized by the substitution of Na^+^ by Ca^2+^, alongside alterations of the surface chemistry. These processes ultimately result in C–S–H crystallization, as illustrated in Figure 27.^9^ Additionally, the authors also discussed the previous data of Garrault–Gauffinet. They suggested that at low supersaturations, the change between amorphous intermediate-driven and crystalline phase-driven nucleation could be the cause of the observed “quasi” constant values of induction times rather than a shift from homogeneous to heterogeneous processes.

**Figure 28:**
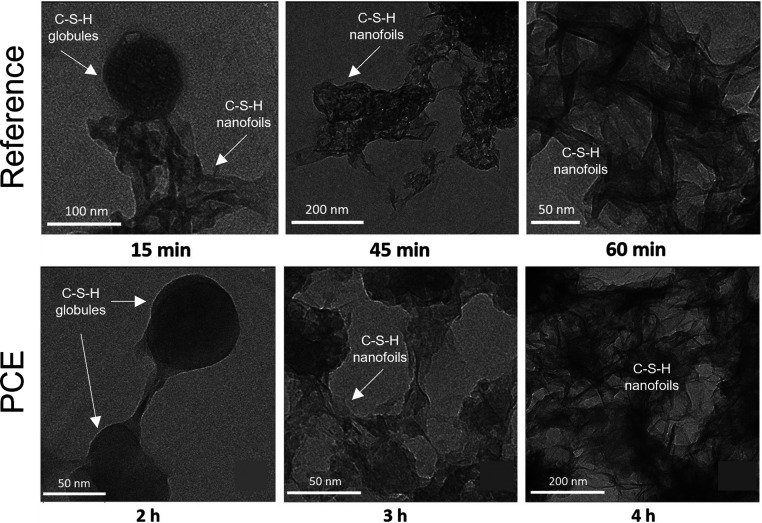
Upper row, TEM images of C–S–H precipitated from Ca(NO_3_)_2_ and Na_2_SiO_3_ solutions after 15, 45, and 60 min of crystallization in the absence of PCEs. In the lower row, TEM images of C–S–H precipitated from Ca(NO_3_)_2_ and Na_2_SiO_3_/PCE solutions after 2, 3, and 4 h of crystallization. Reproduced with permission from ref (10). Copyright 2018 Elsevier.

Electron microscopy investigations conducted by Schönlein and Plank further demonstrated the formation of amorphous C–S–H globules with diameters between 20 and 60 nm, which convert to nanofoils (Figure 28).^10^ Their TEM data revealed that the nanofoils grow from the surface of the globules. At later times, the globules dissolve entirely, giving rise to a dense network of C–S–H nanofoils (thickness of ca. 5 nm). Notably, these two distinct forms, the C–S–H globules and the C–S–H nanofoils, exhibit differences in their molecular structures, as it was uncovered using ^29^Si MAS NMR spectroscopy.^199^ In the globular precursor, branched chains of C–S–H are present, but upon transformation to the nanofoils, only linear chains of C–S–H are detected. The Ca/Si ratio of the foil structures is approximately 0.7, lower than the initially used solutions (Ca/Si = 1.0).

Moreover, organic additives’ influence on the C–S–H precursor’s stability was evidenced (Figure 28, lower row). In the presence of polycarboxylate ethers (PCE), the conversion of the globules to the nanofoil structures is significantly delayed (i.e., approximately 2 h). This delay was attributed to a polymer layer absorbed around the nano globules, effectively slowing down their dissolution and, hence, the transformation into crystalline nanofoils (Ca/Si = 0.8). The charge density of the polymer was shown to be a critical factor in stabilizing these globules. Highly charged anionic PCEs, with a higher adsorption capacity, were found to cause a more significant delay in the transformation into foils compared to weakly charged counterparts, which exhibited a lower adsorption capacity. This pronounced impact of highly charged anionic polymers on delaying foil formation might be the cause of the well-known retarding effect of some PCEs on cement in the early hydration phase.^10^

In the context of C_3_S hydration, it has been demonstrated that C–S–H formation also occurs in the aqueous phase and is not only limited to the surfaces.^200^ Thus, the experimental investigations focused on understanding the homogeneous nucleation of C–S–H are relevant. More specifically, understanding the conditions under which amorphous solid (or liquid) C–S–H precursors form and unraveling the chemical reactions between the dissolved species that govern the initial stages of C–S–H formation in solution is of great importance. The stability and properties of these precursor phases, along with their transformation into more stable phases, can greatly influence cement behavior.

Regarding the C-A-S-H phase, the nucleation mechanism has not been yet unveiled, and thus, fundamental questions such as the influence of aluminum on the distinctive C-A-S-H clusters and whether the two-step nucleation process observed by Krautwurst et al. for the C–S–H system^9^ also applies to C-A-S-H formation, need to be explored. Dedicated research in this area could provide insights into whether amorphous globules are indeed present within the C-A-S-H system and clarify the mechanisms governing their transformation into foil-like structures, should such a conversion occur.

#### 3.3.1 C–S–H Prenucleation Clusters

Mass spectrometric analyses by Picker et al. evidenced an increase in the amount of larger species before nucleation with the continuous addition of calcium to a pure silicate solution.^11^ They suggested that this phenomenon may correspond to the initial stage of C–S–H formation, characterized by the calcium-induced condensation of silicate species, leading to the formation of larger oligomers. However, determining the C–S–H clusters’ composition and structure remained arduous until very recently. A breakthrough in this endeavor came with the identification of C–S–H PNCs through analytical ultracentrifuge (AUC) experiments. These experiments were conducted on C_3_S suspensions and within the C_3_S paste without any additives at different hydration times between 0.5 h–24 h.^201^

It was possible to observe the transient species through a methodical approach of dividing the experimental data into discrete time intervals (packages). These entities underwent a transformation process, evolving into larger species. However, they reached a stage where AUC could effectively observe them. This was primarily due to their fast sedimentation and the increasing polydispersity of these species. Consequently, they no longer settled with a distinct sedimentation boundary. As a result of using this approach, we successfully identified ten distinct species, denoted as s1–s10, based on their sedimentation coefficients. The results presented in Table 2 show remarkable details on the early species in C–S–H nucleation.

**Table 2:** Ten Main Hydrated C–S–H Species as Identified via Their Sedimentation Coefficients (*s*) from AUC Experiments with Their Average Values in the Aqueous Phase of C_3_S Suspension Samples Hydrated over 0.5, 1.0, 3.0, 4.5, 6.0, 15.0, 18.5, 21.5, and 24.0 h^a^

species	*s*_av_	*D*_av_	c*_av_	*d*_D,av_	*ρ*_D, av_	*M*′_av_
group	(S)	(cm^2^/s)	(%)	(nm)	(g/mL)	(g/mol)
s1	0.20	4.59 × 10^–06^	57.02	1.05	1.45	3.99 × 10^02^
	±0.00	±8.83 × 10^–07^	±7.45	±0.25	±0.15	±1.65 × 10^02^
s2	0.57	2.72 × 10^–06^	12.40	1.95	1.44	2.13 × 10^03^
	±0.10	±4.67 × 10^–07^	±12.22	±0.69	±0.11	±1.58 × 10^03^
s3	1.40	1.36 × 10^–06^	4.97	3.59	1.28	1.72 × 10^04^
	±0.19	±4.35 × 10^–07^	±3.47	±0.84	±0.15	±8.91 × 10^03^
s4	2.30	7.45 × 10^–08^	5.83	59.58	1.00	7.02 × 10^07^
	±0.13	±1.09 × 10^–08^	±1.98	±6.54	±0.00	±1.82 × 10^07^
s5	2.49	1.19 × 10^–06^	1.48	4.26	1.38	2.08 × 10^04^
	±0.21	±2.28 × 10^–07^	±0.53	±1.29	±0.14	±8.04 × 10^03^
s6	2.84	2.16 × 10^–07^	3.18	24.47	1.02	4.93 × 10^06^
	±0.25	±6.23 × 10^–08^	±1.14	±5.26	±0.02	±2.86 × 10^06^
s7	4.12	3.87 × 10^–07^	3.08	11.55	1.06	5.23 × 10^05^
	0.47	3.83 × 10^–08^	±1.25	1.60	0.01	2.43 × 10^05^
s8	5.54	9.81 × 10^–07^	4.55	4.44	1.53	4.08 × 10^04^
	±0.27	±7.33 × 10^–08^	±3.41	±0.37	±0.08	±8.00 × 10^03^
s9	7.25	9.27 × 10^–07^	6.00	4.62	1.61	5.02 × 10^04^
	±0.27	±1.82 × 10^–08^	±2.28	±0.09	±0.00	±2.77 × 10^03^
s10	10.40	7.74 × 10^–07^	1.49	5.53	1.61	8.60 × 10^04^
	±0.17	±6.75 × 10^–09^	±0.57	±0.05	±0.00	±2.19 × 10^03^

a The values were averaged over all hydration times and three observation time scan packages.^201^c* is the mean concentration of the species normalized to 100% for all species. *D*_av_ is the average diffusion coefficient from AUC experiments, *ρ*_D,av_ is the average species density, and *M*′_av_ is the molar mass of the hydrated species. Data adapted from ref (201).

Notably, s1 stands out as the smallest among these species, with M’_av_ = 400 g/mol. Intriguingly, this aligns remarkably well with the proposed model of silicate dimers coordinated with Ca^2+^ ions and hydration water, as originally proposed by the group of Wolfgang Tremmel.^9^ Species s2 and s3 exhibit characteristics consistent with PNCs, with the typical sizes and sedimentation coefficients akin to those observed for CaCO_3_.^145^ Species s4, s6, and s7 are liquid droplets of different sizes, evident from their low density, closely resembling that of the surrounding aqueous solvent. Among those, the largest droplets (s4) fit in size to the spherical amorphous particles of around 50 nm that Krautwurst^9^ and Planck previously observed.^10^ However, very small, likely amorphous species with a diameter of only 4 nm (s5) is also noteworthy. Finally, the larger species, namely s8-s10, were attributed to amorphous C–S–H according to density. This study underscored the wealth of information accessible through AUC experiments. AUC has several advantages, including its ability to directly characterize hydrated species in solution, a very high resolution in the Angström range,^202^ and a high statistical significance because it detects every particle.

**Figure 29:**
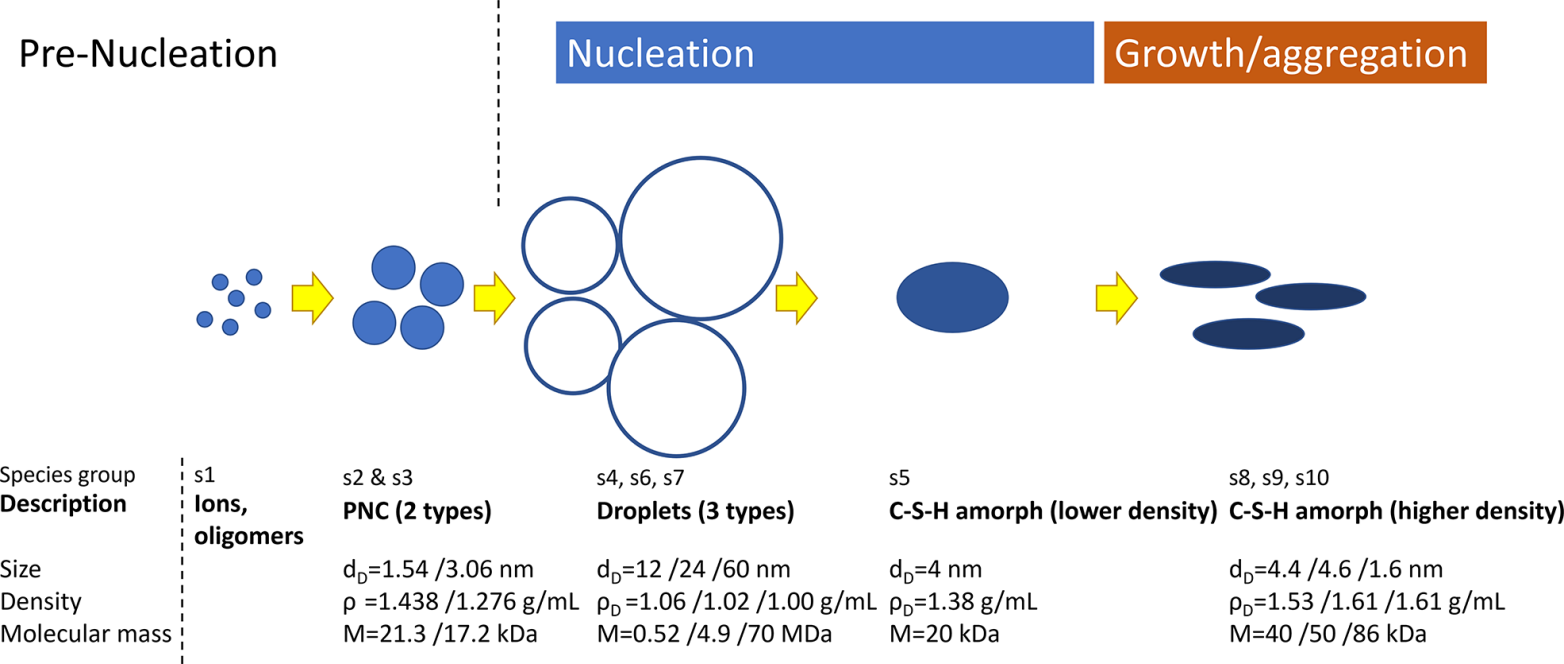
Scheme of the nucleation and growth processes involved during the formation of C–S–H based on the results of our AUC study. Reproduced from ref (201) under a Creative Commons CC BY license. Copyright 2023 American Institute of Physics. https://creativecommons.org/licenses/.

As the sedimentation coefficient (*s*) and diffusion coefficient (*D*) are experimental data for each detected species in AUC, it is possible to derive relevant parameters such as hydrodynamic diameter (*d*), species density (ρ) and molar mass (*M*) of the hydrated species. These parameters provide an unsurpassed level of insight into the early stages of C–S–H formation, facilitating the precise assignment of the different species.^201^ In this context, the results of the AUC investigations have laid the groundwork for proposing a nonclassical mechanism for C–S–H formation, as illustrated in Figure 29. Interestingly, preliminary findings from our research group suggest that the presence of aluminum may impact the stability of C-(A)-S-H clusters, promoting the nucleation of the C-A-S-H phase to some extent, influencing the crystallization pathway compared to the C–S–H system.^203^

The increasing interest in C–S–H prenucleation species has led researchers to use atomistic computational studies to explore the early stages of C–S–H formation. This methodology has provided important insights into the formation of C–S–H species in solution, akin to investigations carried out for other systems like calcium carbonate,^161^ calcium sulfate,^204^ aluminosilicates,^205^ and geopolymers.^206^ Nevertheless, the constraints about the intrinsic time and the length scale of the simulations limit the number of existing studies focused on the atomic-scale nucleation mechanism of C–S–H.^72^ Recently, Yang et al. employed density functional theory (DFT) to simulate the very first stages of C–S–H formation. This was accomplished by calculating interaction energies (Gibbs free energies of chemical reactions) between the monomeric building blocks of the different cement hydrates (C–S–H, C-A-S-H, and C-(N)-A-S-H).^207^ Their study revealed that the most favorable interactions, from a purely thermodynamic perspective, were related to known motifs in the tobermorite-like structures of the different precipitated phases. In all the systems, the calcium–calcium interactions were the most favorable reactions. In contrast, the condensation reactions (i.e., silicate-silicate and silicate-aluminate species) were the weakest interaction.

**Figure 30:**
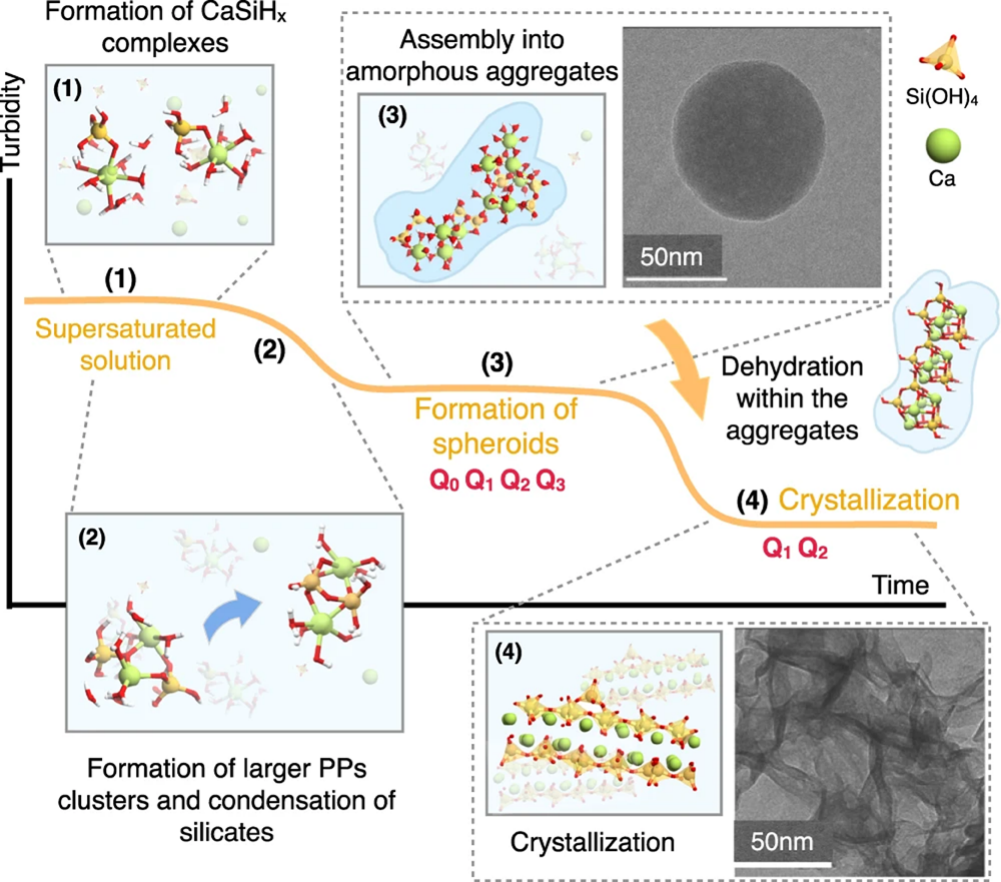
Schematic representation of the turbidity evolution during C–S–H formation (based on ref.^9^) and the proposed mechanism for the early stage formation of C–S–H. (1) Formation of Ca(OH)-O-Si(OH)_3_ complexes from Ca^2+^ and SiO_4_^2–^. (2) Formation of C_2_S_2_H_20_ clusters. (3) Formation of amorphous aggregates upon assembly of larger and highly hydrated elongated clusters. (4) Formation of final C–S–H layers upon dehydration. The experimental TEM image in step 3 shows an amorphous C–S–H globule,^10^ and the TEM image in step 4 the formation of C–S–H layers. The Q_n_ signals in steps (3) and (4) represent the connectivity of the silicate groups.^210^ Reproduced from ref (208) under a 4.0 Creative Commons Attribution License (CC BY 4.0 DEED). Copyright 2023 Springer Nature. https://creativecommons.org/licenses/by/4.0/.

Interestingly, also using DFT simulation methods, Aretxabaleta and collaborators proposed a nucleation pathway for C–S–H that involves four stages (Figure 30), aligning with the available experimental data.^208^ Initially, Ca^2+^ and SiO_4_^2–^ form Ca(OH)-O-Si(OH)_3_ complexes.^209^ Subsequently, these complexes merge, yielding highly dynamic dimers C_2_S_2_H_20_, consistent with the s1 species detected by AUC.^201^ In the third stage, these dimers further aggregate, resulting in elongated and highly solvated clusters, which contribute to the formation of the amorphous globules, as observed in experiments.^10,9^ The density measurements by Krautwurst (ρ = 1.02 g cm^–3^)^9^ and Sowoidnich (ρ = 1.03 g cm^–3^, i.e., s4, s6, s7 species in Table 2)^201^ suggest that these globules could be liquid-like structures. Finally, following the dehydration process, the C_4_S_4_H_2_ structures emerge, and subsequent rearrangement within the aggregates leads to the formation of C–S–H layers.

Although these results provide valuable information about the interactions occurring at the atomic level before C–S–H nucleation, the computational demand of these simulations currently restricts their application to pairwise interactions between the monomeric dissolved species, excluding larger multibody interactions. Nonetheless, as was mentioned in Section 2.2, the latest developments in this field applied to cement research expand the prospect of computational studies as a powerful technique for understanding the nucleation processes occurring during cement hydration,^72^ and as a method for providing reliable parameters for larger scale models.^71^

Concluding this section dedicated to homogeneous nucleation, we trust that the reader now recognizes the importance of unraveling the mechanism of C–S–H formation at the molecular level, which is indispensable for shaping the next generation of sustainable cement-based building materials. The formation of C–S–H and many other relevant phases for the construction industry can be elucidated using concepts originally created to describe the formation of other minerals. These include prenucleation clusters, liquid or amorphous intermediates, nanoparticle building blocks, or aggregation-based pathways. However, there is still much work to be done to solve the complex puzzle of the mechanisms that govern the nucleation of cementitious phases. This exploration is essential for controlling and enhancing the hydration reaction, ultimately improving the properties of cementitious materials, and contributing to their sustainability.

### 3.4 C–S–H Heterogeneous Nucleation

The chase of sustainability in the cement industry, often achieved by reducing the amount of PC, delays the early hydration, leading to slower strength development. The deceleration in the hydration activity is due to the partial replacement of clinker with SCMs or alternative binders with lower reactivity, which is unfavorable within the current construction industry practices. One strategy to accelerate the evolution of mechanical properties without compromising cementitious materials' long-term strength and durability is to promote C–S–H heterogeneous nucleation (see Section 3.1) by controlled seeding. This method facilitates the hydration reaction, compensating for the delay caused by reducing the clinker proportion,^211^ often involving adding nanomaterials with the advantage of significantly higher reactivity.

#### 3.4.1 Common Nanoparticle Additions

Recent attention has been given to the addition of nanoparticles to cementitious systems to promote C–S–H formation.^212,213^ While the acceleration effect of nanometer additions such as nano-SiO_2_ was noted as far back as 1964,^214^ the widespread use of nanoparticles as additives in cement started the 2000s with the reintroduction of nanosilica particles.^215,216^*Nanosilica* is one of the most advantageous nanomaterials that can be added to cement-based systems. In addition to its accelerating effect, it improves the performance of cement and concrete in terms of mechanical behavior, durability, and Ca leaching.^217^ Compared to microsilica particles (silica fume), a more pronounced acceleration effect of nano-SiO_2_ and, consequently, higher compressive strength has been observed, especially at early ages.^218^ A systematic study determined the correlation between the total surface area of the added silica particles and the acceleration effect, where faster hydration originates from the use of smaller sizes (i.e., larger surface areas).^219^ The rapid hydration of cementitious materials in the presence of nanoadditions is generally attributed to one or more of the following mechanisms: 1) the large surface area that the particles provide for the heterogeneous formation of hydration products, 2) the pozzolanic reactivity, and 3) the filling of the gaps between larger clinker particles, resulting in a denser microstructure.^213^

Besides nanosilica, a large variety of nanoparticle additions has been investigated to enhance cement hydration.^213^ Based on their reactivity, there are two major classes of nanoparticle additions. Physical accelerators include nanoparticle oxides such as TiO_2_,^220^ ZrO_2_, Cu_2_O, CuO,^221^ and Fe_2_O_3_,^222^ or other types of nanoparticles like carbon nanotubes.^223^ They primarily function by providing additional surfaces for the hydrates to grow heterogeneously.^224^ Therefore, they are considered inert fillers as they do not chemically intervene in the hydration process. In cases where physical effects dominate, the effectiveness of these additions is dictated by the fineness and dosage of the particles.

The second category includes reactive additions that not only provide sites for heterogeneous nucleation of hydration products but also contribute to hydrate formation by reacting in the pore solution. Thus, besides the particle size of the additions and their content, the composition and crystallography significantly influence the acceleration effect. Prominent examples of reactive additions are the nanosilica particles described earlier, calcium carbonate, and nanoclays.

**Figure 31:**
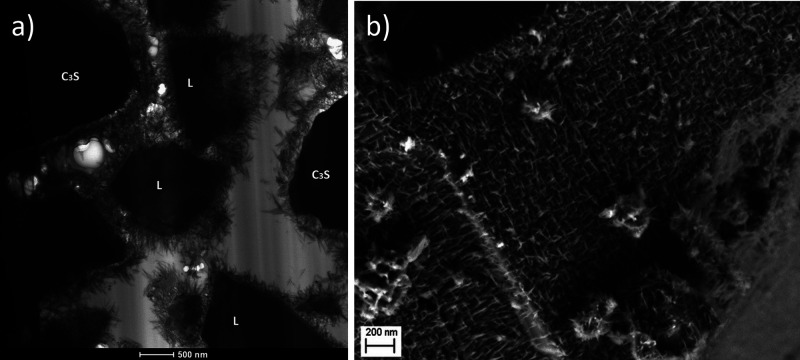
a) STEM image of hydrated C_3_S + limestone. b) SEM image of a limestone grain in limestone–C_3_S paste at 3 h. C–S–H precipitates are aligned on the surface along the calcite in the same direction. Reproduced with the permission of Karen Scrivener from ref (32).

*Limestone* (calcium carbonate) has long been used as SCM in the construction industry at different scales, affecting the properties of cementitious materials through physical and chemical effects.^225^ In the case of micro- and nano-CaCO_3_ seeds, both are reactive additions that accelerate hydration, with more effective behavior of nanosized particles.^226^ Microstructural observations on C_3_S hydration with micro-CaCO_3_ additions illustrated that C–S–H nucleation occurs preferentially on limestone surfaces than on cement surfaces at the end of the induction period.^32,226^ C–S–H needles grow perpendicular to the surface, while on the cement grains, different growth orientations were identified (Figure 31a). The length of the needles is comparable for both, suggesting that CaCO_3_ does not influence the growth of C–S–H.^32^ This boosted formation of C–S–H on limestone was attributed to calcite (the major CaCO_3_ polymorph in limestone) being a favorable template for C–S–H precipitation.^226,225^ The arrangement of the C–S–H precipitates along specific directions on the calcite surfaces (Figure 31b) and the fact that the aragonite polymorph does not accelerate the hydration,^227^ evidence the strong dependence of the templating effect on the crystallographic structure of the used seed.^226^ Apart from the templating effect, CaCO_3_ can react with aluminate phases, creating “extra” hydrated products (calcium aluminate carbonate hydrates) that impact the development of the paste microstructure and contribute to space-filling.^228^

The classification of *nanoclay* particles as reactive or nonreactive additions is ambiguous. Nanoclays consist of crystalline layers of aluminum phyllosilicates with thicknesses of about 1 nm, produced from natural clay particles (e.g., kaolin and montmorillonite) by separating the interlayers by mechanical shearing, thermal and/or chemical modifications. The exfoliation of the clay particles plays an essential role in their reactivity, as it determines the number of accessible surfaces available for reactions.^229^ In the absence of thermal or mechanical treatments, the acceleration effect of nanoclays has traditionally been attributed to the templating of C–S–H nucleation, as no increase in portlandite consumption was detected when added.^230^ However, even without reacting, nanoclays seem to influence the formation of C–S–H hydration products significantly. Solid-state ^29^Si MAS NMR revealed that the C–S–H obtained with bentonite and kaolinite additions exhibits shorter and longer average chain lengths of SiO_4_-tetrahedra, respectively.^230^ Investigations with atomic force microscopy indicated that the surface templating effect of clay particles depends on their size, shape, and negative charge,^231^ which offers new possibilities for engineering cement matrix using different nanoclays. In terms of reactivity, nanomontmorillonite has been observed to exhibit a templating effect and a pronounced pozzolanic behavior, which promotes the formation of calcium silicate/aluminate hydrates.^229,232^ The reported discrepancies in reactivity could originate from the differences in the level of exfoliation of the clays used for the different studies, which would significantly affect their pozzolanic activity.^229^

#### 3.4.2 C–S–H Particles as a Template for C–S–H Nucleation

**Figure 32:**
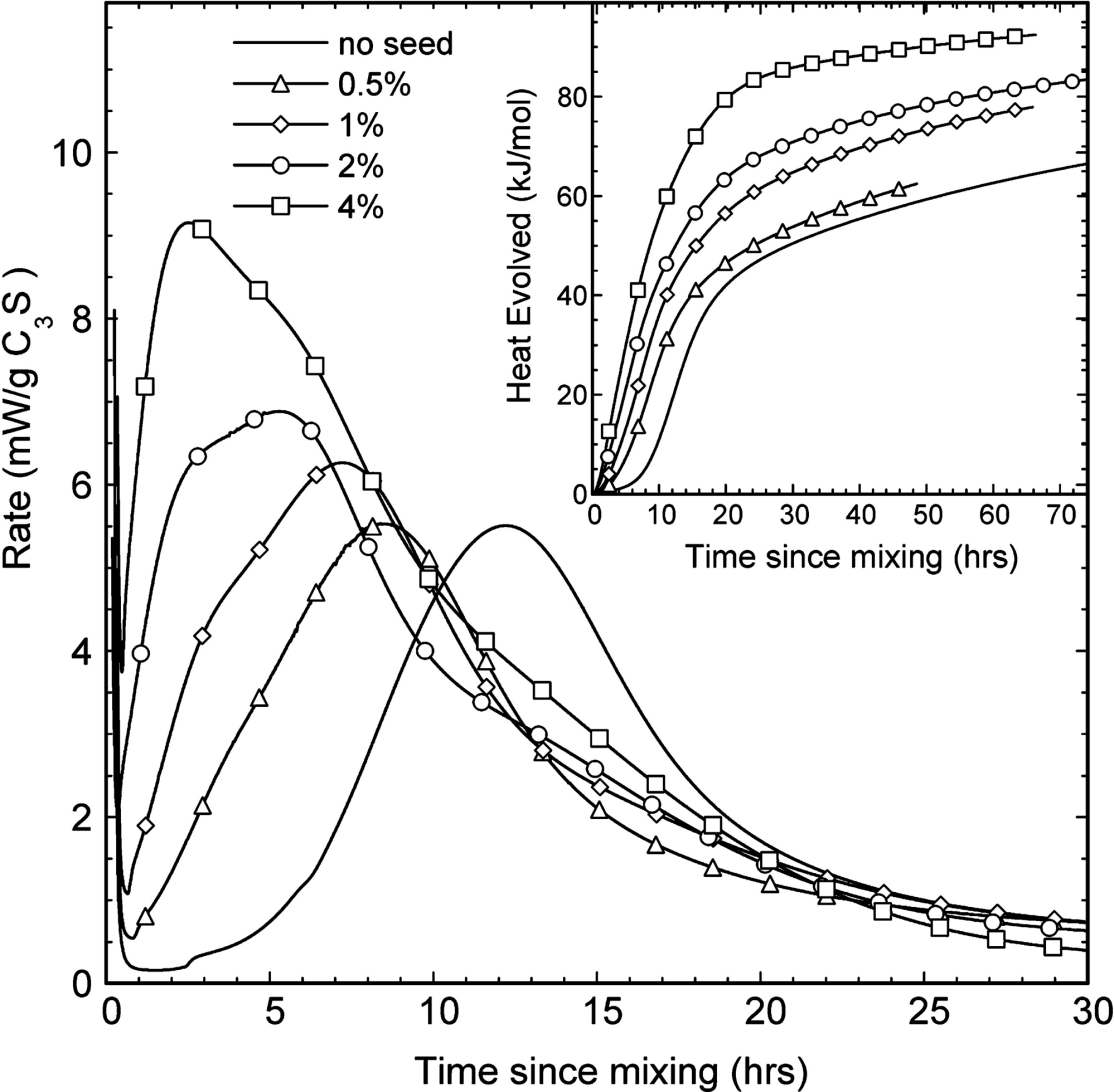
Kinetics of C_3_S hydration with different amounts (mass of solid C–S–H per mass of C3S of C–S–H seeds). The C–S–H seeds were made with a molar Ca/Si ratio 1. Reproduced with permission from ref (30). Copyright 2009 American Chemical Society.

The use of C–S–H seeds is the most effective way to accelerate the hydration reaction of cement and compensate for the slow strength development resulting from the reduction of PC content.^233^ C–S–H seeds are exceptional templates for the nucleation of C–S–H, and therefore, their incorporation to enhance the early strength development in cementitious materials has been a subject of study for several decades.^234,235^ Notably, well-dispersed nanometer-sized C–S–H in cement pastes increases the intensity of the maximum hydration peak and shifts it to earlier times.^212^ This effect is particularly pronounced in the hydration of C_3_S compared to PC. The presence of nanosized C–S–H shortens the induction time and increases the total heat released during the first 60 h, as shown by calorimetry curves obtained during C_3_S hydration (Figure 32).^30^

**Figure 33:**
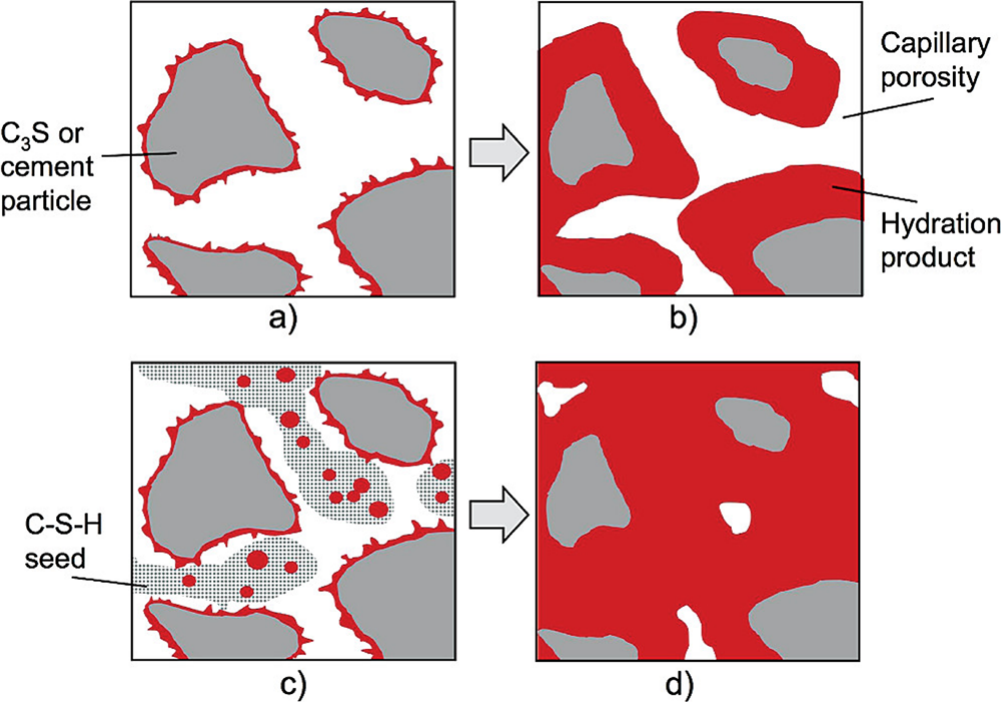
Schematic of the hydration process in the absence (a and b) and presence (c and d) of C–S–H seeds as suggested in ref (30). (a) Normal unseeded paste a few minutes after mixing. The hydration product nucleates on particle surfaces and grows out into the pore space. (b) After several hours, further nucleation and growth is limited by the thickness of the C–S–H layer, leaving significant capillary porosity. (c) Paste with C–S–H seed a few minutes after mixing. The hydration product nucleates both on the particle surfaces and within the seeds, increasing the rate of early hydration. (d) After several hours, the overall extent of early hydration is greater, and there is much less capillary porosity. Reproduced with permission from ref (30). Copyright 2009 American Chemical Society.

The suggested mechanism behind the faster hydration states that seeds act as nucleation sites for C–S–H in the pore space away from the unreacted grain surfaces, and this reduces the inhibition of C_3_S dissolution.^236^ Thus, C–S–H formation occurs simultaneously on the seeds and the clinker surfaces, as illustrated schematically in Figure 33). These two processes can be discerned by calorimetry, where an additional shoulder appeared, with higher intensity correlating to a higher content of C–S–H seeds (Figure 32).^30^ The most widely accepted explanation for the increase in early strength is the change in the microstructure of the cement paste. A denser and more homogeneous paste develops due to the formation of “extra” C–S–H in the pore space, reducing porosity.^237^

**Figure 34:**
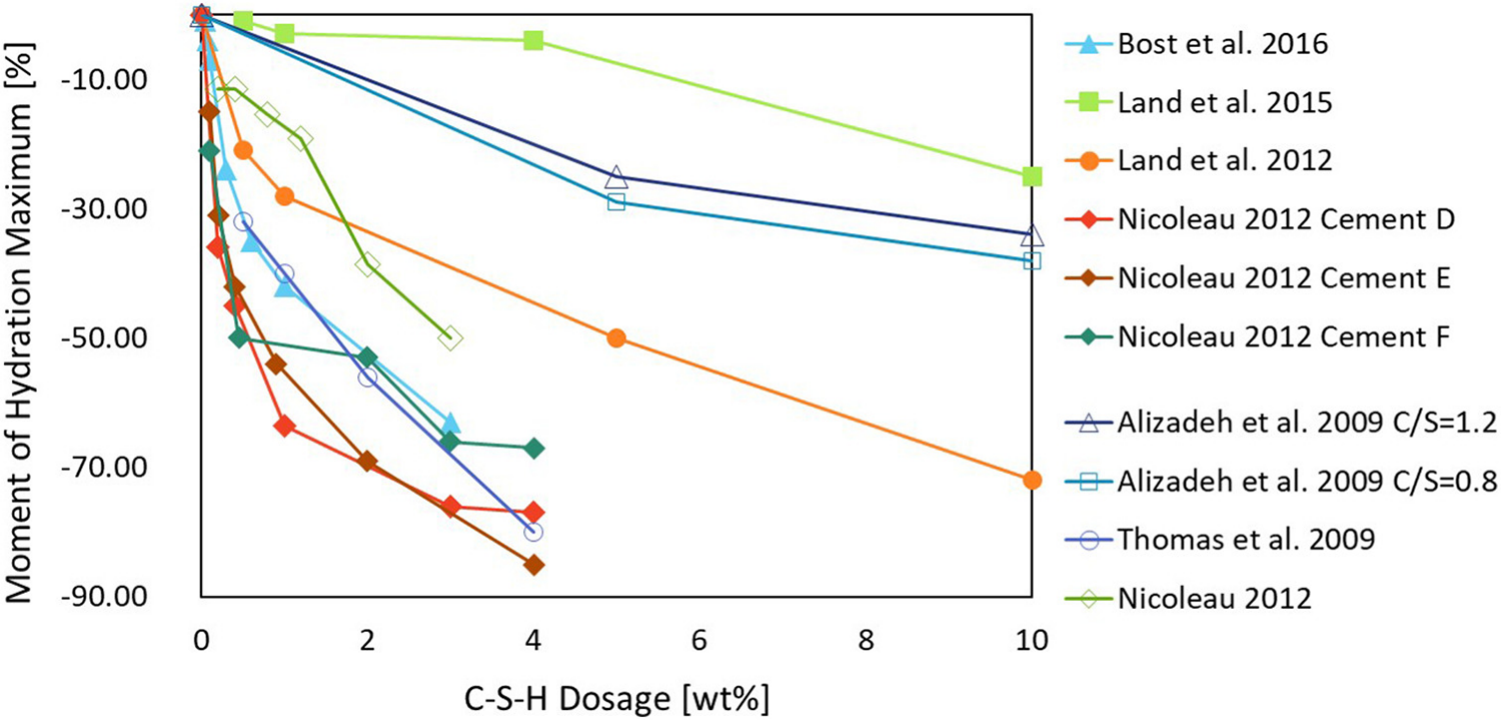
Relative acceleration of hydration (defined by the shift of the time of the maximum) as a function of the added weight percentage of C–S–H seed. For original sources of the data in the legend, the reader is referred to the reference list of ref (212). Reproduced with permission from ref (212). Copyright 2018 Elsevier.

Many studies have demonstrated the accelerating effect of nanosized C–S–H in various systems such as C_3_S, PC, and PC-containing SCMs (Figure 34).^212^ The proportion of C–S–H used ranges from 0.04 wt % to a maximum dose of 10 wt %, beyond which no further accelerating effect was observed.^212^ For a glimpse of the most notable acceleration effects, Thomas et al. reported 80% earlier hydration and 67% higher maximum hydration using 4 wt % C–S–H (prepared by coprecipitation) in C_3_S hydration.^30^ The most remarkable effect has been documented by Nicoleau, who added 4 wt % of X-Seed to PC, resulting in 85% earlier and 120% higher hydration maximum.^238^ The X-Seed consists of polymer-stabilized C–S–H nanoparticles patented by Construction Research & Technology GmbH, a subsidiary of BASF.^239,240^ This marked the beginning of the current family of concrete accelerators known as Master X-Seed, based on C–S–H nucleation seeding technology. It is worth noting that in the case of PC hydration (and not only C_3_S), the optimal dosage also depends on the mineralogical composition of the cement used, particularly the amounts of alkali sulfate and gypsum present.^238^

The characteristics of C–S–H seeds have been identified as significant factors influencing their acceleration capability, with the Ca/Si ratio being a key determinant.^241^ Alizadeh et al. found that the hydration process of C_3_S is greatly accelerated when C–S–H seeds with a higher silicon content are used.^242^ These results were confirmed in a recent and systematic study by John et al.^241^ They also noted that acceleration did not evolve linearly with Ca/Si of the seed. Changes in the hydration products compared with the unseeded system were also observed, and the long-term strength was decreased at low water-to-cement ratios.^241^ These studies did not explore the influence of seeds on microstructure, which could provide valuable information for optimizing seed composition and morphology. Understanding this aspect could contribute to improved performance, especially in scenarios with low water-cement ratios.

#### 3.4.3 Synthesis of C–S–H Seeds

The efficacy of C–S–H seeds is primarily determined by their size, shape, and chemical composition (Ca/Si ratio), which are highly dependent on the synthesis method.^212^ The development of numerous innovative synthesis procedures for C–S–H particles over the past few years (e.g., pozzolanic synthesis, precipitation method, sol–gel, mechanochemical synthesis, hydrothermal synthesis) has opened up many possibilities for tuning their properties.^212,243^

The precipitation method has achieved particle size control in the presence of polymeric additives.^13,244,237^ This method is notable for being straightforward, fast, and inexpensive. It simply consists of mixing Ca- and Si-bearing precursor solutions, varying the reaction conditions (i.e., concentrations and type of precursors, pH,^245^ additive types^246,247^) to modify the characteristics of the seeds. Incorporating polymers during the precipitation reaction generally allows better control over the precipitation process and stabilization of the seeds against agglomeration. A prominent example is particle size adjustment by varying the side chain length of poly(ethylene glycol) methacrylic-*co*-ω-methoxy acid methacrylate esters.^248^

**Figure 35:**
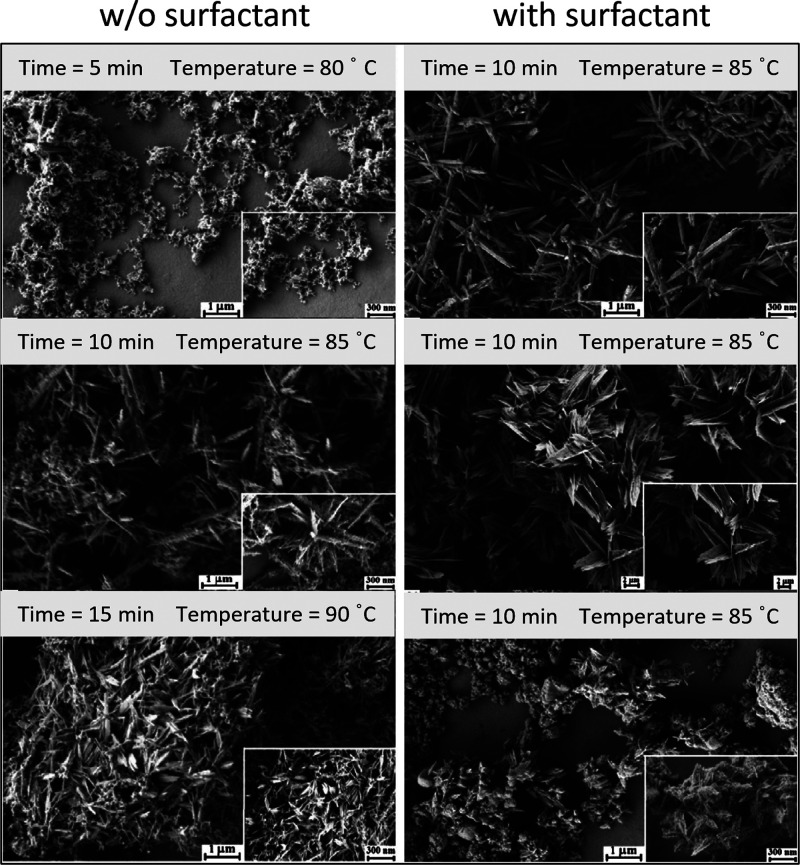
SEM micrographs of the C–S–H particles precipitated without sodium dodecyl sulfate (left column) and with sodium dodecyl sulfate (right column). The duration of the ultrasound treatment and temperature are included in the graph. In sodium dodecyl sulfate’s presence, the concentration increases from top to bottom. Reproduced with permission from ref (249). Copyright 2014 Elsevier.

Tailored shapes can be prepared by mixing the precursor solutions and subjecting them to ultrasonic irradiation. This method has also established organic additives as a key shape-controlling factor. Adjusting the ultrasound irradiation time and the surfactant concentration effectively regulated particle size and morphology (Figure 35). While needle-shaped C–S–H was obtained in the absence of additives, adding a surfactant (sodium dodecyl sulfate) led to the formation of nanosheets.^249^

**Figure 36:**
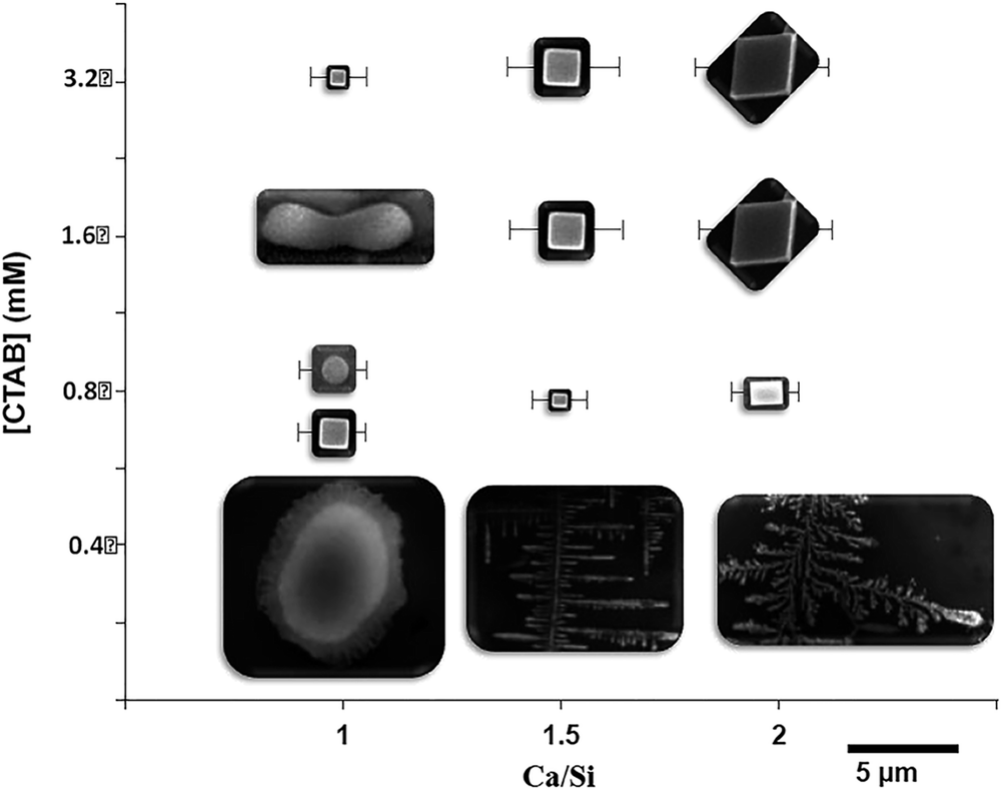
Morphology diagram of calcium silicate hydrate particles obtained via in situ seed generation and seed-mediated overgrowth. The particle size of the particles is in scale. The error bars indicate upper and lower particle sizes. Reproduced with permission from ref (251). Copyright 2017 Royal Society of Chemistry.

Distinct C–S–H hollow microspheres with large surface areas have also been produced using ultrasound irradiation and cetyltrimethylammonium bromide (CTAB) as surfactant. The authors claimed that apart from the ultrasound treatment and the surfactant, the Ca source also influences the formation of the C–S–H hollow spheres.^250^ It is even possible to obtain C–S–H particles with unusual well-defined morphologies (i.e., cubic, rhombohedral, dendritic, and core–shell particles) with the ultrasound method by first generating CaCO_3_ seeds and following using seed-mediated growth that facilitates precise shape control. The variation of Ca/Si ratio, surfactant type and concentration, mixing method, and counterions in the precursor appeared to be sufficient to control the morphology of the C–S–H particles (Figure 36).^251^

Other methods like sol–gel^252^ or mechanochemical^253^ synthesis have been used to produce C–S–H particles. However, control over either size or morphology cannot be easily achieved, as the sol–gel method in calcium-containing systems typically leads to a gel and not to colloidal C–S–H.^252^ In the case of mechanochemical synthesis, silicon oxide particles react with Ca(OH)_2_ solution, forming C–S–H at their surface, which is then removed during the grinding process. Grinding again allows the SiO_2_ particles to be exposed, making them available for further reaction with the calcium hydroxide solution so that more C–S–H can be formed. The process is repeated until complete turnover of the silicon oxide particles is achieved. With mechanochemical synthesis, the Ca/Si ratio of the seeds can be easily manipulated, but in contrast, neither the sizes nor the morphologies can be efficiently targeted.^212^

In summary, promoting the heterogeneous nucleation of calcium silicate hydrate using seeds is a relatively inexpensive method to improve the early hydration of cementitious materials. Among the possible surfaces, nucleation seeding with C–S–H has been shown to have enormous potential to accelerate cement hydration. The low cost of most starting materials and the simplicity of the synthesis procedures have enabled the industrial application of C–S–H seeding in cement production (MasterX-Seed). Although controlling the size and morphology of C–S–H seeds involves using expensive organic additives, the resulting benefits of the final material, such as higher early strength, make it well-suited for low-carbon cements containing SCMs.

Current research is already exploring how C–S–H seeds impact blends with reduced PC content due to the incorporation of SCMs, like LC^3^ blends.^254,255^ From our perspective, it is crucial that future research looks closely at how acceleration works on a smaller scale, not just sticking to the usual compressive strength and calorimetric measurements. When dealing with blended systems or alternative binders, we suggest studying C–S–H seeds and also other types, such as calcium carbonate, because the different hydrates formed in those cases may require different seeds. Furthermore, it is important to emphasize that the existing research on the production of C–S–H seed particles with specific sizes and shapes is applicable to the production of well-ordered materials with better properties using a bottom-up approach.

## 4 Crystallization of Hydrated Phases in Alternative Binders

Using alternative binders also represents a central strategy to lower the CO_2_ of the cement industry in the technological roadmap developed by the IEA.^2^ The term alternative binder refers to fine mineral materials that set relatively fast (but not too fast to remain castable) when in contact with water and/or CO_2_ and can be considered surrogates for PC in concrete or mortars.^256^ The reduction in CO_2_ and greenhouse gas (GHG) emissions of an alternative binder is typically due to lower process-related emissions and/or lower production energy requirements than PC.^257^ For nearly two centuries, the construction industry has been dominated solely by PC, and thus, introducing new binders is a major challenge, as it could imply an integral transformation of the entire business. Traditional cement manufacturing companies do not welcome this prospect. Moreover, the vast amount of research devoted to investigating PC hydration, mechanical properties, and durability cannot be directly extrapolated to these new binding materials, making their rapid implementation impossible.

**Figure 37:**
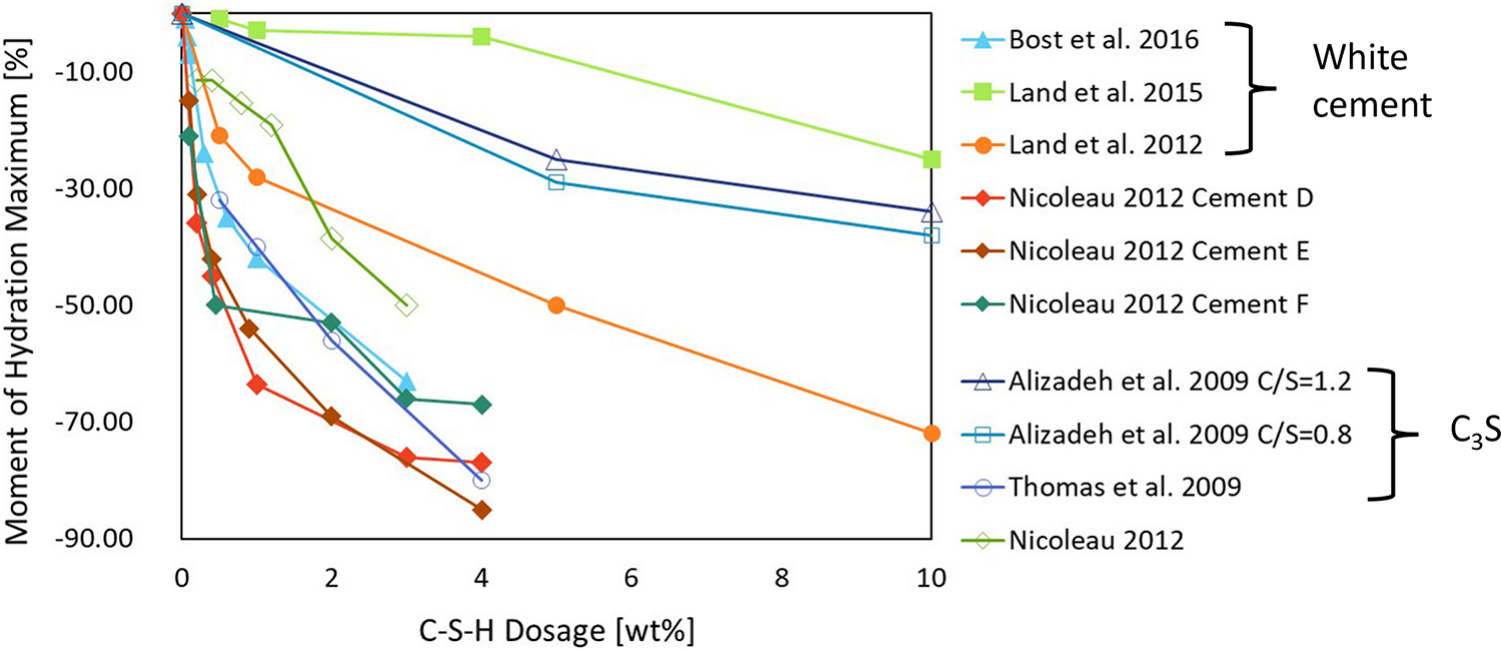
Process CO_2_ emission generation intensity for selected alternative binders and their application stage. PC = Portland cement, CSA = calcium sulphoaluminate, BCSA = belite calcium sulphoaluminate, CACS = carbonatable calcium silicates, MOMS = magnesium oxide derived from magnesium silicates. Reproduced from ref (2) under a 4.0 Creative Commons Attribution License (CC BY 4.0). Copyright 2018 IAE. https://www.iea.org/terms/creative-commons-cc-licenses.

Six categories of alternative binders have been identified as crucial in global efforts to reduce CO_2_ emissions associated with cementitious materials (Figure 37): alkali-activated binders, reactive belite-rich Portland cement binders (RBPC), calcium sulphoaluminate binders (CSA), belite calcium sulfoaluminates (BCSA), carbonatable calcium silicate cements (CASC), and magnesium oxide cements derived from magnesium silicates (MOMS). While innovation is essential to improving the sustainability of PC, even after 150 years of dedicated research, substantial groundwork is required in alternative binders. In this review, we provide a concise description of three selected alternative binders: binders based on alkali-activated (AA) materials, carbonatable calcium silicate cements (CCSC), and magnesium oxide cements sourced from magnesium silicates (MOMS). Compared to the other three alternative binders, they are further away from commercial applications. In our opinion, these emerging binders would significantly gain from fundamental academic research, as they possess high potential for CO_2_ reduction.

Although most of the literature on cement-based materials and concretes tends to emphasize technological aspects rather than microstructural or compositional properties of the binding phases, in this review, we focused on examining the hydration products, microstructure, crystallization mechanisms, and phase transformations of alkali-activated materials, CACS and MOMS binders, which are key determinants of their overall performance. We also include some relevant literature on the influence of organic additives in forming and stabilizing the major binding phases formed in these systems. This is important because additives-controlled crystallization (Section 5) could address significant material challenges of these binders, including extended setting times, rapid setting, and the instability of the hydrated phases. Each section dedicated to a specific binder concludes by identifying areas necessitating intensive research within the scope of this review. This includes a focus on crucial aspects such as the crystallization mechanisms of primary binding phases and the role of organic additives.

### 4.1 Alkali-Activated (AA) Binders and Geopolymers

The working principle of alkali-activated (AA) binders is to mix an aluminosilicate source (precursor) and an alkaline activator (alkaline solution) to form insoluble hydrous alkali-aluminosilicate and/or alkali–alkali earth-aluminosilicate binding phases. Aluminate solid sources, such as calcined clays, coal fly ash (FA), blast furnace slag (BFS), and natural pozzolans, yield cementing phases when activated by alkali metals in the form of hydroxide or aqueous silicates. The main difference with PC is that AA binders require an alkaline component to raise the media’s pH, allowing the precursor’s dissolution, whereas PC hardens upon mixing with water. The strongly alkaline medium is necessary to release the silicate and aluminate monomers from the precursor, enabling the subsequent formation of the binding phases.

#### 4.1.1 N-A-S-H and C-A-S-H Binding Phases

**Figure 38:**
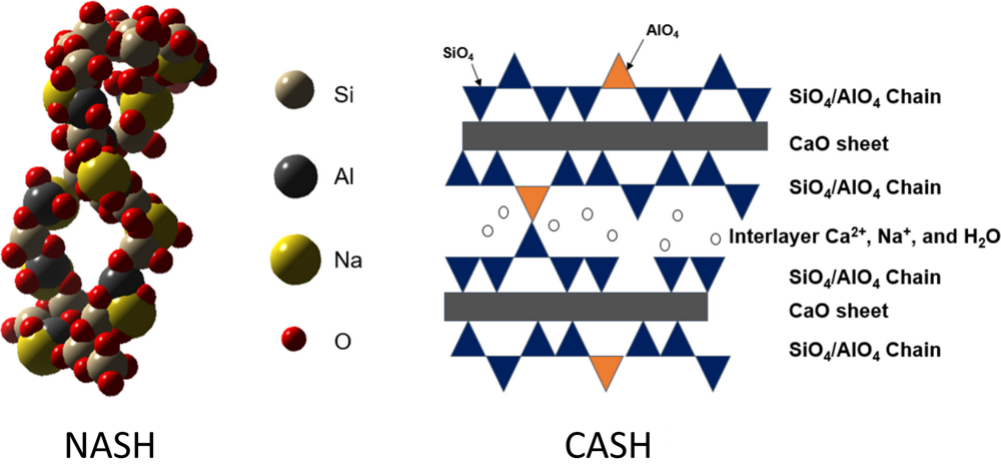
Schematic representation of sodium aluminosilicate hydrate (N-A-S-H) and calcium aluminosilicate hydrate (C-A-S-H) gel structure. On the left is the three-dimensional structure of N-A-S-H, and on the right is the linear chain structure corresponding to C-A-S-H. Reproduced with permission from ref (258). Copyright 2018 John Wiley & Sons.

The relevant phases in AA binders are mainly sodium aluminosilicate hydrate (N-A-S-H) or calcium aluminate silicate hydrate (C-A-S-H), depending on the calcium content of the aluminosilicate precursor. Structurally, N-A-S-H and C-A-S-H are significantly different, as N-A-S-H has a three-dimensional structure of linked aluminate and silicate tetrahedra, whereas C-A-S-H presents a layered structure comparable to C–S–H as it was explained in Section 2.3 (Figure 38). Therefore, the aluminosilicate precursors are normally characterized by high and low calcium oxide content, which determines the gel type that will dominate the cement paste.

The *low-calcium content AA binders* were termed “geopolymers” because their hydrates formed by a polymerization reaction comparable to that which produces polymeric substances.^259^ Common precursors for geopolymers include calcined kaolinitic clays (metakaolin, MK) due to their high reactivity. Upon alkali activation, the resulting binder consists of agglomerates of nanocrystalline zeolites compacted by an amorphous and highly cross-linked aluminosilicate gel phase, N-A-S-H.^260^ The N-A-S-H gel has been structurally described as a 3D network of aluminate and silicate tetrahedra linked with shared oxygen atoms (Figure 38a). Alkali metal ions (e.g., Na^+^) compensate for the negative charge of the framework introduced by the linked tetrahedral aluminosilicate units.

The pH of the media plays a crucial role in influencing the reaction of the precursors.^261^ Elevated pH levels have been demonstrated to enhance the dissolution of the precursors, resulting in a higher concentration of free silicon and aluminum ions.^262^ This higher Al concentration has also been associated with improved mechanical properties.^263^ In FA geopolymers, a notable increase in compressive strength is observed when raising the pH from 12 or 13 to 14. This was attributed to the exponential rise in free aluminum derived from FA in solutions with increasing alkalinity.^263^ The effect of pH on N-A-S-H formation has been studied using a synthetic approach to avoid the dissolution step of the precursors and thus investigate any additional effect of the alkalinity on the gel properties.^264^ Observations revealed a correlation between alkalinity and the size of globular particles within N-A-S-H gels. As pH levels rose, there was a concurrent decrease in the Si/Al ratios of the gels, coupled with a higher polymerization degree. This is attributed to the increased presence of Al(OH)_4_^–^ units, which promotes their condensation, resulting in larger particle sizes in the gel, even at a microstructural scale. These findings highlight the potential for morphological control of the gels by adjusting Si/Al ratios during their formation.

**Figure 39:**
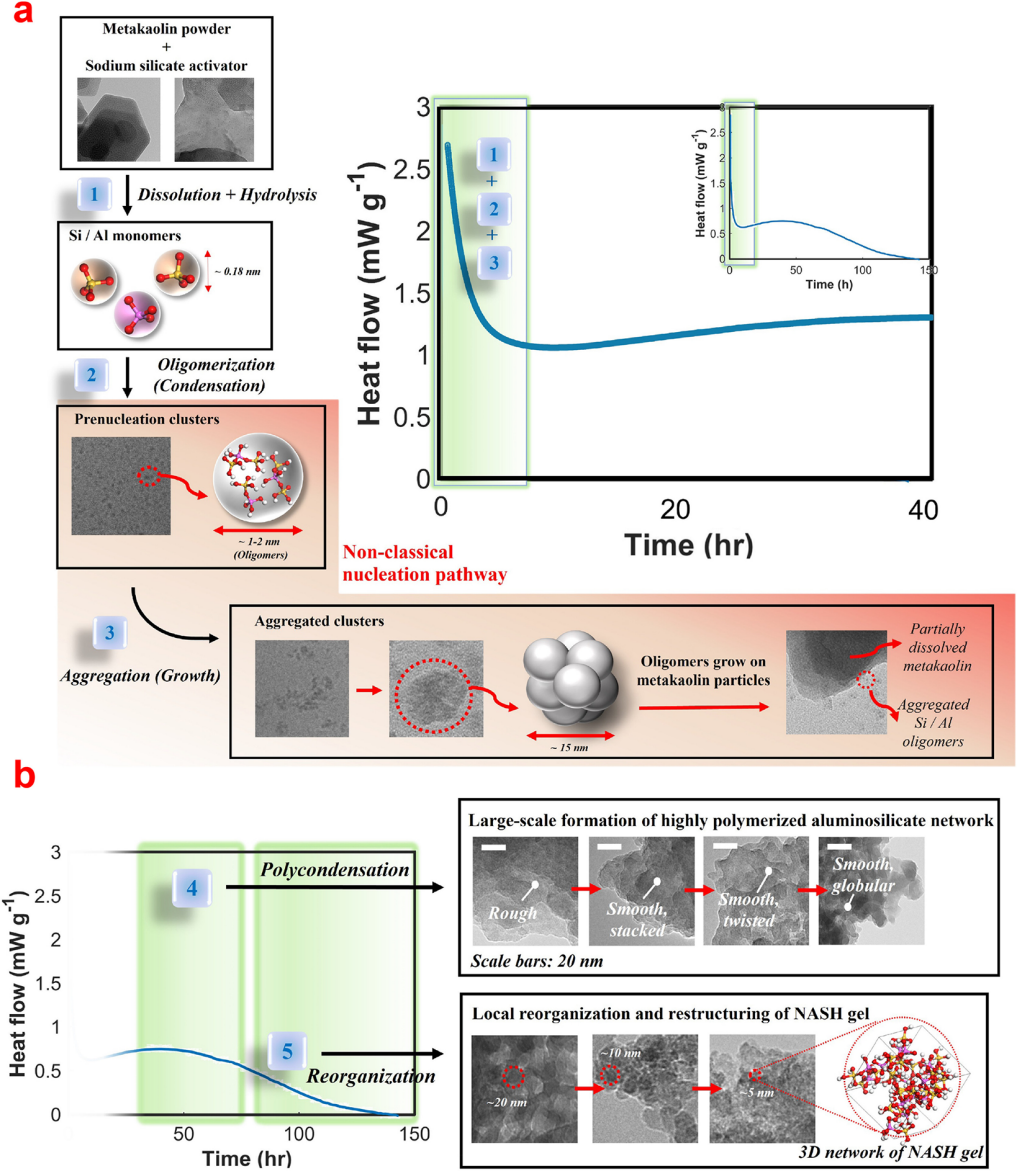
Schematic representation of the formation mechanism of geopolymers as proposed in ref (193). a) Dissolution/hydrolysis (1), oligomerization (2), and aggregation (3) are the three main reactions controlling the early age geopolymerization. A nonclassical mechanism describes the nucleation pathway of the geopolymer according to reactions 2–3. Prenucleation clusters of Si/Al monomers (ca. 1–2 nm) are suggested to form in the solution during step (2). Upon aggregation, they form larger clusters (ca. 15 nm), which subsequently grow on the metakaolin particles (step (3)). b) Polycondensation reaction 4 leading to the formation of mature N-A-S-H gel, illustrating the transformation from a rough sheet-like structure to a smooth, globular structure. The reorganization reaction 5 causes the globular size of the N-A-S-H gel to become smaller (from ca. 20 nm to ca. 5 nm) and the formation of a final 3D network of N-A-S-H gel. Reproduced with permission from ref (193). Copyright 2022 Elsevier.

While the N-A-S-H gel primarily dictates the physicochemical characteristics of geopolymers, our understanding of its nucleation and growth processes is limited. In a recent investigation focused on the early stages of N-A-S-H formation, a multistep formation pathway consisting of various intermediate stages before the final polymeric network has been proposed (Figure 39). The authors suggested a nonclassical nucleation mechanism in which prenucleation clusters (ca. 1–2 nm) form in the first minute of the reaction and agglomerate into partially polymerized globules (ca. 15 nm). After 6 h of reaction, a marked event in the calorimetry curve highlighted the conversion of the low polymerized globules to the more stable, highly polymerized N-A-S-H gel. As the geopolymer aged, the N-A-S-H gel rearranged, and the aluminosilicate network showed reduced porosity, with more globular and smaller particles. Their results demonstrate another system following a nonclassical crystallization pathway.^193^

**Figure 40:**
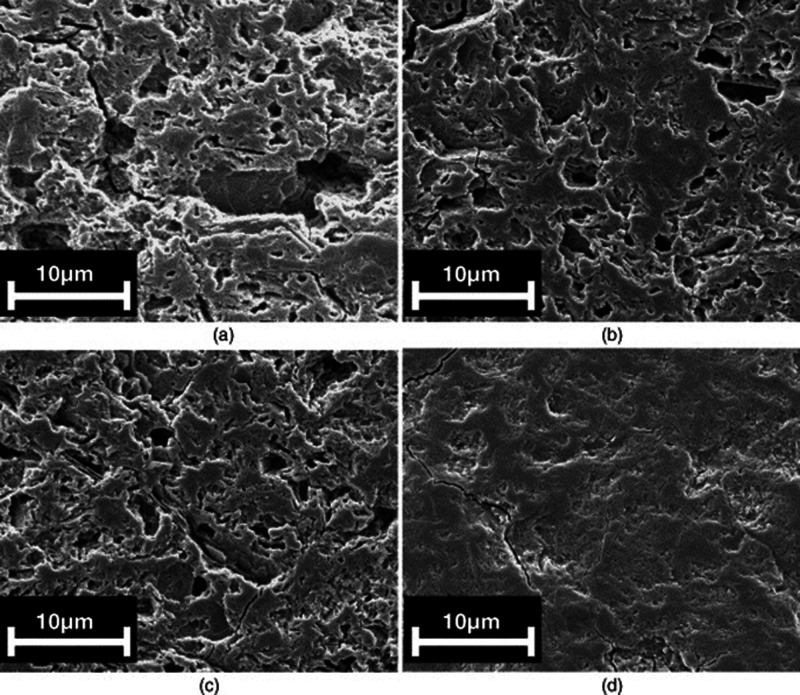
SEM images of geopolymer microstructures with Si/Al = (a) 1.45, (b) 1.50, (c) 1.55, and (d) 1.60. Reproduced with permission from ref (265). Copyright 2005 Elsevier.

The microstructure of the final gel (Figure 40), dictated by the Si/Al ratio, has been shown to regulate the material’s mechanical properties, as in many other systems.^265^ For gels with Si/Al ≥ 1.65, more homogeneous and less porous microstructures correlate with enhanced mechanical properties, whereas the worse mechanical behavior at Si/Al ratios ≤1.40 is explained by the higher porosity of the formed gel (Figure 40d). The highly cross-linked (Q^4^) aluminosilicate “geopolymer” gel^266^ provides good properties like chemical resistance (to acids) or high-temperature resistance, attributed to N-A-S-H’s low bound water content compared with C–S–H gel. As a drawback, it should be noted that geopolymers tend to crack and shrink because of the high-water demand,^267^ and are therefore, usually blended with high-calcium AA materials such as slags or PC to reduce the water requirements.

The *high-calcium AA binders* typically result from the reaction between BFS and an alkaline solution, having a Ca/(Si + Al) ratio of approximately 1. The products formed are considerably different from geopolymers, comparable to PC hydration products, but with a considerably lower calcium content (i.e., Ca/Si ratio between 0.8 and 1.8).^268^ The incorporation of significant amounts of Na to balance the charge and/or adsorbed onto the gel makes it sometimes referred to as C-(N)-A-S-H. The composition and the structure of the obtained gel C-(N)-A-S-H varies depending on the type and concentration of the activator,^268^ the characteristics of the precursor in terms of composition and reactivity,^269^ the binder synthesis procedure,^270^ and the paste curing conditions.^271^ For instance, when silicate-based activators are used, the C-A-S-H gel has a lower Ca/Si ratio and lower structural order, while NaOH induces a higher crystallinity and higher Ca/Si ratios.^268^

*Blended systems* that combine less reactive aluminosilicates (i.e., MK or FA) with a more reactive calcium precursor (i.e., BFS or PC) and alkali activators allow using of low-reactive wastes or byproducts. In these systems, the coexistence of N-A-S-H and C-A-S-H enhances the mechanical performance. C-A-S-H has been suggested to fill the pores and voids of the N-A-S-H gel, resulting in a homogeneous and denser microstructure and increased strength.^272^ In contrast to the plain PC binders, significant changes in the gels and the crystalline phases occur in these systems for up to 180 days.^273^ Over time, C-A-S-H gels become more homogeneous in composition, while N-A-S-H gels maintain a variable composition. A hybrid C–N-A-S-H gel substituted with Al and Na has been identified with C-A-S-H and N-A-S-H. Some hypotheses claim that C–N-A-S-H gels could be formed by calcium substitution in N-A-S-H gels or by sodium sorption or substitution in chain silicate C-A-S-H gels, or they may even coexist at an indistinguishable scale.^273^

Regarding future research efforts, in our opinion, the co-occurrence of multiple gels presents new possibilities for tailoring the chemical and physical properties of these blended cementitious systems following a bottom-up approach. From an academic level, the complex chemistry that dictates the formation and the properties of the distinct gels in blended systems must be uncovered, prioritizing the investigation of molecular interactions of the ionic components of the gels (i.e., sodium, calcium, aluminate, silicate, and water) under controlled precipitation conditions, and the characterization of the nanostructure of the coexistent gels.

Furthermore, the careful selection of the precursors and activators, together with the development of novel activation methods, is important to advance AA binders. For instance, recently, mechanical activation of CaCO_3_ and Na_2_SiO_3_, yielding an amorphous solid solution of both components, has been proven beneficial.^274^ In this approach, molecular dispersion of the ionic components in the solid phase reduces transport distances, promoting the precipitation of the Na-containing gels (C–N–S–H) during subsequent chemical activation.^274^ The exploration of new strategies to control the reactivity of the precursors and the subsequent precipitation of the binding phases is essential, so the formation pathway of gels from a single precursor with different activators should be carefully studied to allow direct comparison. Additionally, researchers should continue their seek for environmentally friendly alkali activators derived from silica or alkaline-rich waste materials (e.g., rice husk ash, brine-derived alkali hydroxides, glass powder, sugar cane and bamboo ashes, diatomaceous earth, and silica), and also pursue the reduction of the doses employed.^275^

### 4.2 Carbonatable Calcium Silicate Cements (CASC)

Mineral carbonation stands out as an effective technology for CO_2_ storage,^276^ and has been proposed as a pivotal strategy to deal with the CO_2_ emitted during calcination in cement kilns.^2^ CO_2_ sequestration in the cement industry involves various methods, such as carbonation of demolished concrete and carbonation hardening. In the context of recycled concrete, carbonation serves to improve the quality of aggregates for incorporation into new concrete.^277^ Additionally, carbonating the recycled cement paste opens the possibility of utilizing it as an SCM.^278^ The carbonation hardening of cement-based materials is a particularly interesting approach because of the rapid strength development in cementitious matrixes when cured in the presence of CO_2_, along with the potential use of calcium silicate phases with a low hydraulic activity that can harden by carbonation.^279^ The wealth of research on the carbonation of cementitious materials leads us to focus here on the nature of the mineral products formed during carbonation. We consider this essential, as the complex interaction between these mineral products within the cement matrices governs the progress of carbonation and, consequently, the strength development.

Carbonatable calcium silicate cements (CASC) differ significantly from hydraulic binders in that they do not harden by reaction with water but by reaction with CO_2_. In this respect, carbonation of low-lime calcium silicate phases is very attractive due to the double effect of 1) CO_2_ savings in the production of the clinker phases (i.e., lower amount of limestone and lower clinkering temperatures) and 2) CO_2_ fixation during the hardening process. The replacement of alite by low-lime calcium silicate phases in the clinker such as wollastonite (CaO·SiO_2_, CS), rankinite (3CaO·2SiO_2_, C_3_S_2_) and belite (2CaO·SiO_2_, C_2_S) is not possible due to their limited hydraulic reactivity. Consequently, the development of the strength in nonhydraulic calcium silicate cements through carbonation has opened new possibilities for their incorporation in cement-based materials. This concept is not novel; lime-based binders were widely used by ancient civilizations such as the Mayas, more recently by the Greeks and Romans, and still have applications today.^280^ Recent findings suggest that lime clast inclusions typically found in Roman mortars may serve as a calcium reservoir facilitating the formation of CaCO_3_. This process is triggered by the reaction with atmospheric CO_2_ during the crack opening, providing these ancient structures with advanced self-healing functionalities.^281^

The fundamental principle underlying carbonation in cementitious materials is analogous to the natural weathering of silicate minerals or carbonation of basaltic rocks, where CO_2_ fixation occurs through the formation of stable inorganic carbonates.^282,283^ The CO_2_ can be incorporated at different stages: 1) during the carbonation of the raw materials, such as recycled aggregates or cement residues; 2) by injecting CO_2_ into the cement-based material in its fresh state; 3) through the curing of the cement-based composite in a CO_2_-rich environment.^284^ Carbonation of recycled aggregates has demonstrated an improvement in mechanical performance and a reduction in water adsorption,^285^ while carbonation of fresh cement paste has similarly resulted in enhanced mechanical properties.^286^ Nevertheless, both methods possess lower CO_2_ capture efficiency compared to carbonation curing,^287^ which involves the direct reaction of the anhydrous calcium silicate phases to form the carbonate phases, which act as binders.

Developing low-lime binders (γ-C_2_S, C_3_S_2_, CS, etc.) activated by carbonation only took off recently.^288^ In general, during carbonation hardening, the calcium silicate phases are initially mixed with water to form a paste, which is then molded and cured in CO_2_-rich environments at different temperatures (T) and relative humidities (RH). During this process, carbonation appears to be initially regulated by the dissolution of the calcium silicate phases, followed by the diffusion of ions through the microstructure of the products formed.^284^ Hydration (i.e., formation of C–S–H and Ca(OH)_2_) and carbonation (CaCO_3_) reactions occur simultaneously in the presence of CO_2_ and H_2_O. Interestingly, the effectiveness of the latter is more pronounced under identical carbonation conditions, even for the C_3_S phase, which has the highest hydraulic activity.^288^ Under specific curing conditions, it was observed that the carbonation rate of γ-C_2_S, C_3_S_2_, and CS closely approximated that of C_3_S. The calcium carbonate phases formed originate from three distinct sources: direct carbonation of calcium silicates (eq 9), dissolution of portlandite (Ca(OH)_2_) (eq 10), and from C–S–H, which eventually transforms into silica gel and CaCO_3_, in a process called decalcification (eq 11).^289^
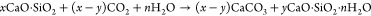
9
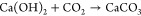
10
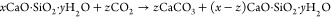
11The carbonation mechanism of nonhydraulic calcium silicates (γ-C_2_S, C_3_S_2_, CS) differs from that of hydraulic calcium silicates (C_3_S and β-C_2_S) since Ca(OH)_2_ is not formed as an intermediate product.^290^ Regarding the C–S–H gel formed during carbonation, it seems to be similar to that formed purely by hydration reactions, and it was identified in the early stages of curing for C_3_S, β-C_2_S, γ-C_2_S, and C_3_S_2_. This initially formed C–S–H is transformed into a highly polymerized silica gel (low calcium content) at longer times. In the case of the CS, the silica gel seems to form directly and not from altering C–S–H.^288^

The kinetics of the carbonation reaction and the characteristics of the crystalline phases, including polymorph, morphology, habit, or size, are highly influenced by the curing conditions such as temperature, *p*CO_2_, pH, [Ca^2+^]/[CO_3_^2–^], supersaturation, and the presence of additives.^291,292^ Furthermore, the carbonation products formed vary depending on the crystal structure of the silicate phases, a factor that impacts their dissolution rate, i.e., the rate of calcium and silicate release to the solution. For instance, in the case of CS, which has a chain-like structure, carbonation leads to calcite and amorphous silica. In contrast, the carbonation of pseudowollastonite, characterized by a ring-like silicate structure, results in the formation of aragonite as the primary carbonate phase, accompanied by calcium silicate crystalline phases in the form of platelets.^293^ Noncrystalline CaCO_3_ has also been identified as a carbonation product during the accelerated carbonation of calcium silicates.^294−296^ It is suggested that a silica coating layer could stabilize amorphous calcium carbonate (ACC), hindering its transformation to the most stable polymorphs.^296^ This observation aligns with the findings of Kellermeier et al., who observed a similar phenomenon during the precipitation of calcium carbonate in the presence of silica.^297,298^

The mechanical performance of the carbonated cement pastes has been correlated with the crystallinity of the CaCO_3_ products.^299,300^ When the matrixes were dominated by metastable calcium carbonate products, i.e., ACC, vaterite, and aragonite, the elastic modulus was reduced, while the flexural strength was increased in comparison with the matrixes where calcite was the predominant precipitated phase.^300^ Poorly crystalline phases appear to have beneficial effects on the increase in the strength of the matrix,^301^ and the reduction of the atmospheric carbonation,^302^ due to alterations of the pore size distribution of the paste.^278^ However, this reduction in pore size obviously hinders the progress of the diffusion-based carbonation reaction. This was confirmed in the work of Asraf et al., where ACC was formed in all the calcium silicate phases investigated (C_3_S, γ-C_2_S, C_3_S_2_), except for CS, where carbonation was observed to be highly efficient, and calcite was the primary reaction product.^296^

From the above points, achieving polymorphic control during carbonation hardening is a key factor in producing a satisfactory performance carbonated material. Recent strategies highlight the use of organic additives, which have demonstrated the ability to promote the formation of ACC during carbonation of wollastonite, together with vaterite and aragonite.^303^ The matrixes obtained in the presence of amino acids exhibit a lower critical pore size than plain CS, which translates into increased flexural and compressive strength. This highlights the potential of biomolecules to enhance the performance of CASC.^303^ Simple inorganic additives, such as magnesium ions, have also been shown to play a relevant role in preventing the decalcification of the C-A-S-H gel during slag carbonation by stabilizing ACC.^302^ However, care must be taken regarding the long-term stability of these binders. For example, the transformation of ACC into more stable polymorphs occurs via interface-coupled dissolution–precipitation reaction,^304^ which compromises the stability of the paste over time.

**Figure 41:**
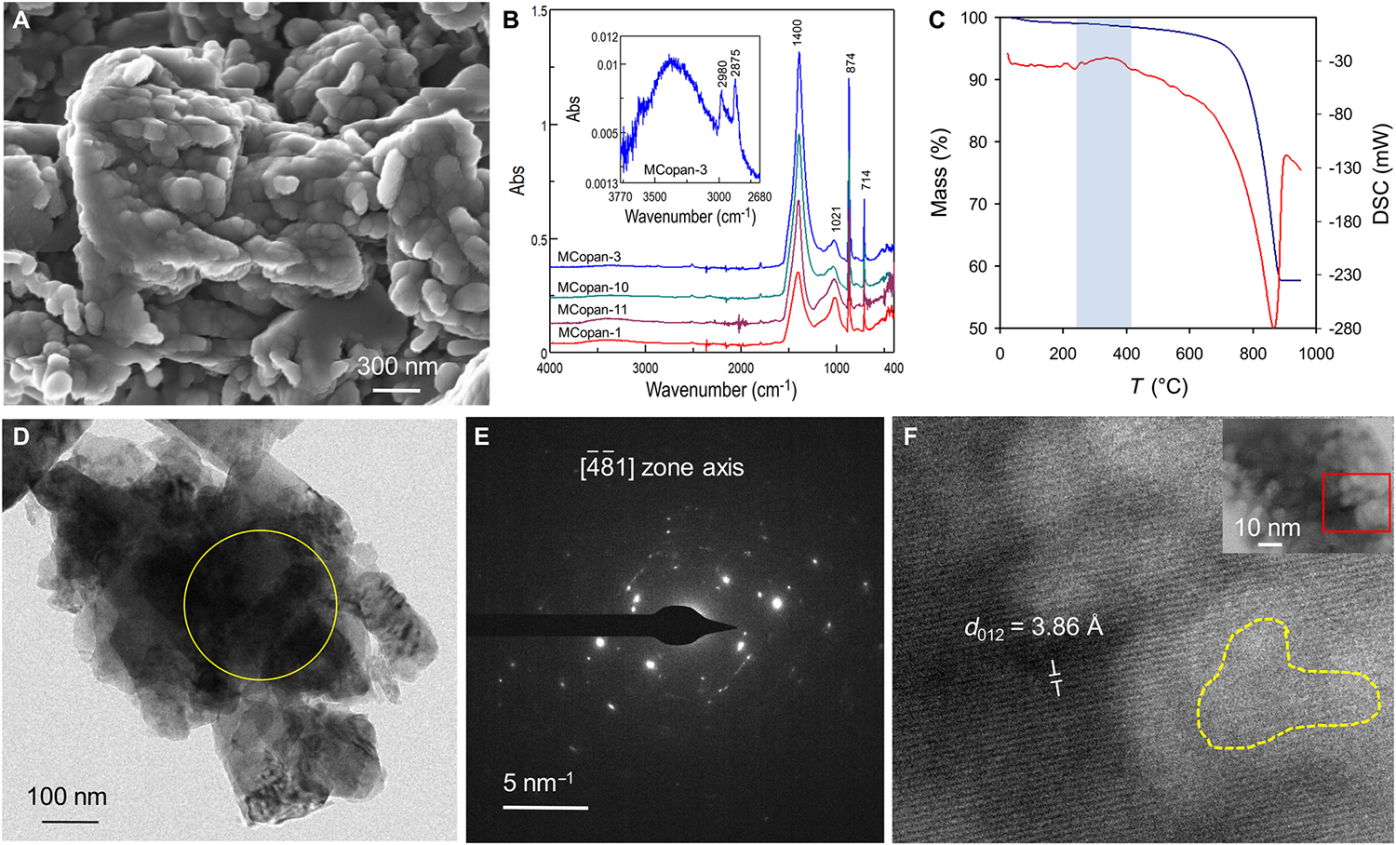
Composition and microstructure of Copan lime-based plasters. (A) Representative FESEM image of the nanogranular structure of calcite cement including organics. (B) FTIR of Copan plasters shows intense bands corresponding to calcite (1400, 874, and 714 cm^–1^) and a small broadband at 1021 cm^–1^ corresponding to silicate phases (aggregate). The inset shows a detail of the spectra, which includes small amounts of polysaccharides as demonstrated by the C–H stretching bands at 2980 and 2875 cm^–1^. Abs, absorbance (arbitrary units). (C) TG (blue) and DSC (red) curves show an exothermic band (shaded rectangle) corresponding to organic thermal decomposition in air. (D) TEM image of calcite crystal. (E) SAED pattern of the yellow circled area in (D). The spots display an angular spreading of ∼4°. (F) HRTEM image of calcite in (D) (overall area in inset) showing lattice discontinuity (yellow dashed area) due to an amorphous inclusion. Reproduced from ref (305) under a Creative Commons Attribution Non-Commercial License 4.0 (CC BY-NC). Copyright 2023 American Association for the Advancement of Science. https://creativecommons.org/licenses/by-nc/4.0/.

In addition to exerting polymorphic control, the occlusion of organics within the carbonated products could be exploited to create biomimetic organic–inorganic hybrid structures, which exhibit superior mechanical performance compared to pure calcium carbonate phases (see Section 6.1). Interestingly, this phenomenon has been observed in the lime-based mortars of Copán (Honduras), relics of ancient Mayan craftsmanship, which stand out as some of the most durable lime plasters on the planet.^305^ There is evidence that Maya masons used plant extracts rich in polysaccharide,^306^ that could be the key behind the incredible durability of their lime-based materials. Rodriguez-Navarro et al. have shown that, most likely without knowing, the Mayas masons used a biomimetic strategy to produce superior binders involving the incorporation of organics in between and inside the calcite crystals (Figure 41). This gave the mortar a plastic behavior and a higher toughness while increasing its resistance to weathering processes.^305^ This biomimetic approach is really promising for improving the properties of CASC.

In addition to the CaCO_3_ phases, a pivotal component of CASC is the silica gel (Q^4^ silicate), also known as Ca-modified silica gel. Characterized by a higher polymerization degree compared with C–S–H, this gel mainly consists of Q^3^ and Q^4^ species.^307,308^ Morphologically, it appears as a sheet-like structure, denoting its layered character. The silica gel was also intermixed with calcium carbonate, forming a composite phase identified by considerably higher Ca/Si ratios than the silica gel alone. This composite phase, identified by the absence of Q^1^ and Q^2^ peaks in NMR, constitutes the highest volume fraction of the products.^307,308^ In terms of mechanical properties, the silica gel presents elastic modulus and hardness close to those of the high-density C–S–H,^67^ while the composite phase surpasses it.

In the natural alteration of silicate minerals, similar highly polymerized silica gels have been identified, known in the geochemistry community as surface altered layers (SALs) or surface leached layers.^309^ Extensive research has focused on the study of SALs, exploring their impact on the dissolution rates of silicate minerals and their reactivity in CO_2_ sequestration through the precipitation of secondary minerals. The formation of SALs has been traditionally described by incongruent dissolution models,^310^ which propose preferential leaching of cations based on the nonstoichiometric release of the elements during dissolution of multicomponent silicates.^309^ Nevertheless, experimental evidence, such as the existence of an extremely sharp interface between the parent mineral and the altered layer,^311^ cannot be adequately explained by incongruent dissolution models.

In situ AFM observations further revealed that during the dissolution of CS, etch pits form and spread,^312^ which implies that stoichiometric amounts of ions must be released to the solution.^313^ Subsequently, a new phase, identified as silica gel, was observed to nucleate on the surface along the step edges.^312^ These observations supported the idea that SALs form via an interface-coupled dissolution–precipitation model. In this model, dissolution is stoichiometric, but supersaturation at the interface of the dissolving phase (calcium silicate) can be achieved, enabling the precipitation of a new mineral phase (silica gel) even if the bulk is unsaturated with respect to the new phase.^314^ Indeed, recent studies have shown the existence of strong gradients in the composition of the fluid during wollastonite dissolution.^315^

**Figure 42:**
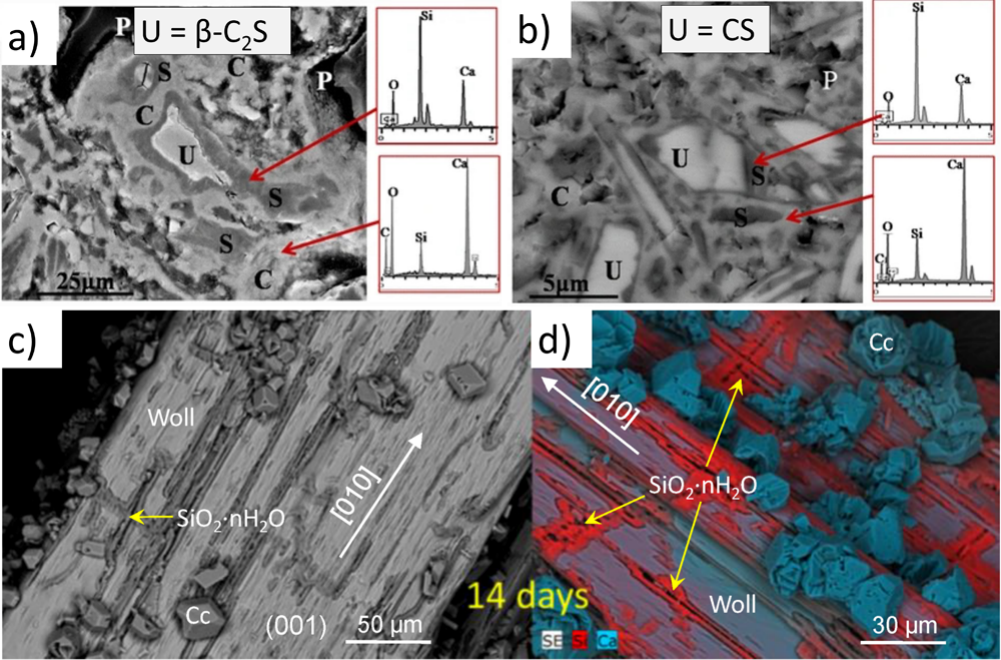
SEM images of carbonated calcium silicate phases. Backscattered electron image (BSE) and energy dispersive X-ray (EDX) analysis of carbonated a) β-C_2_S and b) CS samples. “S” indicates Ca-modified silica gel, “U” indicates unreacted β-C_2_S, “C” indicates CaCO_3_, and “P” indicates pores. Reproduced with permission from ref (288). Copyright 2016 Springer Nature. c) BSE image and d) EDX compositional of wollastonite crystals reacted for 14 days. Crystals are elongated along [010], showing marked cleavage and, in some sections, less marked {001} cleavage. Note the preferential nucleation of calcite (*Cc*) on the areas where amorphous silica forms at {100} cleavage planes and fractures nearly parallel to [100]. Yellow arrows point to modified silica gel. Reproduced from ref (319) under a 4.0 Creative Commons Attribution License (CC BY 4.0). Copyright 2018 MDPI. http://creativecommons.org/licenses/by/4.0/.

The total amount of CaCO_3_ generated has been reported to reach a plateau with prolonged CO_2_ exposure, attributed to the development of a passivating layer around the unreacted calcium silicate grains formed by CaCO_3_ (with the relevant role of ACC discussed earlier) and silica gel.^316−318,288^ It has been suggested that the silica gel layer initially facilitates the dissolution of the pristine silicate mineral, but over time, it becomes a denser, less porous layer that potentially acts as a diffusion barrier^318^ along with the carbonate phases (Figure 42a, b).^319^ Notably, the formation of the silica gel and the carbonation products is anisotropic. In both cases, it preferentially proceeds along the [010] direction (Figure 42c, d), in agreement with the fastest dissolution rate of wollastonite.^320^ This anisotropic deposition of products may impede further wollastonite dissolution along the most preferential directions, hindering the release of Ca and the subsequent carbonation. This passivation process, well-documented in the geological context, likely plays a crucial role in impeding complete carbonation in cementitious systems.

CASC based on nonhydraulic phases bears the potential to reduce the carbon dioxide emissions related to the cement industry, up to approximately 30% of CO_2_ avoidance compared to PC,^257^ which is a promising value. In the context of this review, research efforts aimed at maximizing CO_2_ fixation in CASC should prioritize two primary objectives:

•*Characterizing Carbonation Products*. Thoroughly examining the characteristics of carbonation products that form under varying curing conditions is essential. A considerable body of literature has extensively explored the carbonation of calcium silicates,^321^ yet the precise control of the phases obtained and the systematic identification of parameters governing their formation remain areas requiring more attention, since most of the existing data are not comparable. Research should study the obtained phases regarding composition, crystallography, morphology, size, and their correlation with mechanical properties. In our view, one promising avenue is the biomimetic approach of using organic or inorganic additives capable of regulating carbonation products and even incorporating them into the crystals, thus improving their mechanical performance. The extensive knowledge from the well-explored precipitation of CaCO_3_, as discussed in Section 3, should be applied to control and optimize the formation of calcium carbonate in CASC. This includes the application of additive-controlled crystallization techniques, as elucidated in Sections 5 and 6.

•*Understanding Silica Gel Formation*. A comprehensive understanding of the formation mechanism of the silica gel from the C–S–H and directly from the calcium silicate unreacted phase is also crucial due to its discussed relevant role in hindering the progress of carbonation. The formation of the silica gel phase seems to be linked to the dissolution rate of calcium silicate phases,^293^ and its occurrence might be mitigated in instances where the calcium silicate phases exhibit faster dissolution rates. This aspect deserves further examination, especially since incongruent dissolution has been proposed as the mechanism behind silica gel formation, a perspective that we consider inaccurate based on AFM investigations, which suggest an interface-coupled dissolution–precipitation reaction.^312^ Moreover, efforts should be directed toward regulating the porosity, connectivity, and nanostructure of this layer to facilitate the progression of carbonation, thereby enhancing the overall performance of CASC.

### 4.3 Magnesium Oxides Derived from Magnesium Silicates (MOMS)

**Figure 43:**
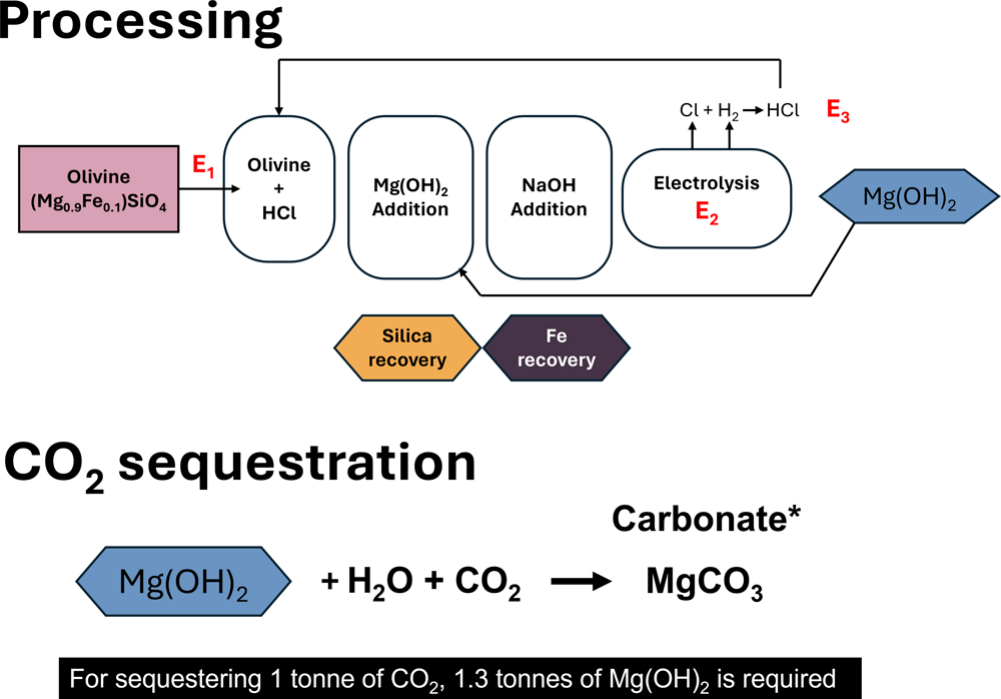
Simplified Mg(OH)_2_ extraction from olivine-rich rocks and carbon sequestration. Reproduced from ref (324) under a 4.0 Creative Commons Attribution License (CC BY 4.0). Copyright 2021. http://creativecommons.org/licenses/by/4.0/

Among the alternative binders discussed in this review, MgO-based cements offer the most significant potential for CO_2_ reduction, even though they are currently furthest from practical application, despite theoretically having the highest CO_2_ reduction potential if they were to replace PC (Figure 37). The slow development of these cements can be attributed to two primary factors: their internal pH, which is not sufficiently high to passivate steel reinforcing bars, and the lack of a sustainable long-term supply for MgO, especially in the European Union.^322^ The sustainability of MgO-based binders depends on the use of raw materials such as magnesium silicate rocks,^323,324^ or low-value waste streams such as Mg-rich desalination brines^325^ or mine tailings for obtaining MgO.^256^ In this context, the extraction of MgO from ultramafic rocks that do not contain chemically bound CO_2_ emerges as a promising strategy, provided an energy-efficient process can be implemented to separate MgO from the rest of the components. In particular, the feasible production of Mg(OH)_2_ from globally abundant serpentine- and olivine-rich rocks, developed recently (Figure 43),^323,324^ has launched new possibilities for the future use of MgO-based cements.

This family of binders uses MgO instead of CaO as the main building block, whose chemistry is very different from that of CaO. Unlike the CaO–SiO_2_–Al_2_O_3_, no magnesium silicate phases formed at higher temperatures that possess hydraulic properties. Therefore, obtaining a cementing material is only feasible by blending MgO with oxysalts (i.e., carbonates, silicates, or phosphates to produce binding phases) since the hydration of MgO yields magnesium hydroxide (brucite, Mg(OH)_2_), a low-strength material. Importantly, it has been observed that brucite forms not only on the pore space but also on the surface of the MgO particles. This hinders, especially at alkaline pH, the dissolution of MgO and, thus, the release of Mg ions, which are necessary for the subsequent formation of binding phases.^326^

**Figure 44:**
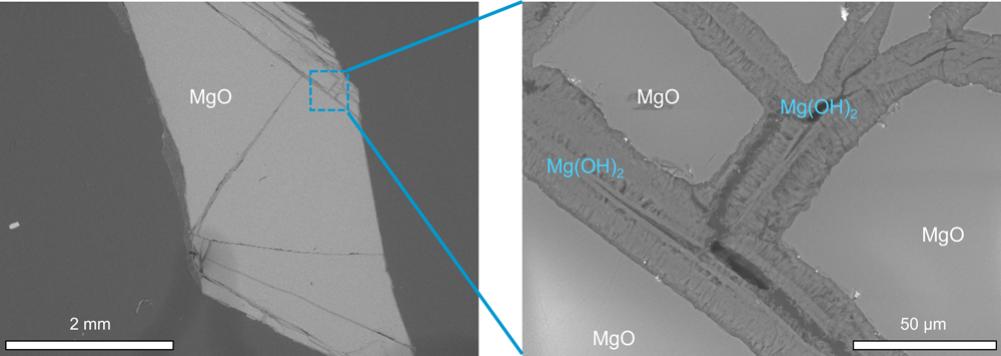
MgO crystal reacted at pH 11 and 40 °C for 40 days. The Mg(OH)_2_ layer is composed of highly oriented brucite crystals, which might be responsible for the significant slow of the hydration reaction. Images courtesy of Lisa Huber and Lars Dieminger (Master’s students at the University of Konstanz).

The limited hydration of MgO, attributed to the formation surface passivation layer of brucite,^327^ constrains the reaction with oxysalts and the associated strength gain. In our recent investigations, it was noted that at pH 11 and 40 °C, the progression of the hydration reaction significantly decelerated after 20 days. This slowdown was ascribed to the development of a layer comprising highly oriented brucite crystals on the MgO surface (Figure 44). 2D-XRD analyses revealed an epitaxial relationship between MgO and the formed brucite (unpublished results), potentially triggering surface passivation.

To tackle this problem, additives such as acetate,^328,329^ HCl, and MgCl_2_,^329^ along with high-temperature conditions, have proven effective in enhancing the hydration of MgO and promoting further reaction.^330^ The proposed mechanism for the enhanced hydration suggests that CH_3_COOMg^+^ or Cl^–^ facilitates the diffusion of Mg^2+^ ions away from the MgO surface, where they precipitate without passivating the surface.^328,331^ Additionally, nucleation seeds play a role in improving the degree of hydration. Specifically, the use of hydromagnesite has been instrumental in promoting the nucleation of Mg(OH)_2_ on the seeds, preventing the blockage of MgO surfaces.^332^

Further drawbacks emerge when considering the use of MgO-based binders, notably a higher water demand, which generally results in lower compressive strengths and a faster loss of workability compared to PC.^333^ These originate from the stronger hydration and more rigid hydration shells of Mg^2+^ ions in comparison to Ca^2+^ ions. To maintain workability and control the setting, water-reducing agents, retarders, and accelerators are mandatory when using MgO-based cements. Commercial additives like PCEs can partially mitigate these challenges but often need high doses.^334^ In this context, the scarcity of research on formulating and perfecting admixtures tailored for MgO blends is a significant impediment to their practical application, highlighting the need for increased attention in this area.

The roadmap of MgO-based cements toward broader applications depends significantly on research and development efforts, ranging from the molecular scale to macroscopic properties. In the following sections, we aim to present an overview of the most promising magnesia-based cements, including Mg-carbonate, silicate, oxychloride, oxysulfate, and phosphate. We will focus especially on the characteristics of the primary Mg-binding phases, their crystallization pathways, and microstructure while identifying areas of research that deserve attention within the scope of this review. For an in-depth understanding of the macroscopic properties, limitations, and current applications of MgO-based cements, we recommend consulting the extensive review by Walling and Provis published in this journal.^335^

#### 4.3.1 Magnesium Carbonate (MC) Cements

Magnesium carbonate-based binders (MC) derive strength from the reaction of MgO with CO_2_, transforming it into Mg-carbonate cement-based materials. MgO reacts with water and CO_2_, leading to a wide range of carbonates and hydrated carbonates phases (HCMs), which are responsible for the hardening of the material.^336^ The strength development is attributed to two factors: reduced pore volume, with HCMs having a significantly higher volume, and the formation of a well-connected network of magnesium carbonate crystals that contributes to the binding strength.^337^ This leads to good mechanical properties, typically between 20 to 50 MPa.^326,334^

The phase assemblage in MC pastes consists of nesquehonite (MgCO_3_·3H_2_O), hydromagnesite (4MgCO_3_·Mg(OH)_2_·4H_2_O), dypingite (4MgCO_3_·Mg(OH)_2_·5H_2_O), artinite (MgCO_3_·Mg(OH)_2_·3H_2_O), an amorphous phase, and a large amount of residual uncarbonated Mg(OH)_2_.^333,336,338^ The carbonation process of MgO begins, as for every MgO based cement, with the hydration reaction that yields Mg(OH)_2_ upon water contact. Subsequently, the carbonation of Mg(OH)_2_ takes place. The reaction rate, type of carbonates formed, and the extent of the carbonation determine MC’s mechanical performance. In carbonation curing at elevated CO_2_ concentrations, HMCs precipitate from the initially formed brucite, resulting in rapid strength gain.^333,336,338^ However, the need for accelerated carbonation curing, as mentioned for CASC, limits the application of MC to relatively thin precasting elements,^336^ reducing its versatility.

An important discovery was made when reactive MgO was hydrated with a high amount of hydromagnesite (up to 30%).^326^ This resulted in significant improvement in the mechanical properties of MC at ambient conditions, avoiding the need for carbonation curing.^334,339^ Blends composed of MgO and hydromagnesite seeds (9/1 ratio) can reach compressive strength comparable to PC after 1-day.^340^ However, the precise nature of the cementing products formed in these blends remains unclear. Some studies suggest a mix of poorly crystalline brucite and an unidentified amorphous carbonate hydrate,^326,334,341^^342^ while others report poorly crystalline Mg(OH)_2_, nesquehonite, and artinite as the primary HMCs phases formed.^339^ Nevertheless, what is evident is that the new assemblage formed in the presence of hydromagnesite must exhibit a high degree of cohesion, as indicated by the reported values of compressive strength.

**Figure 45:**
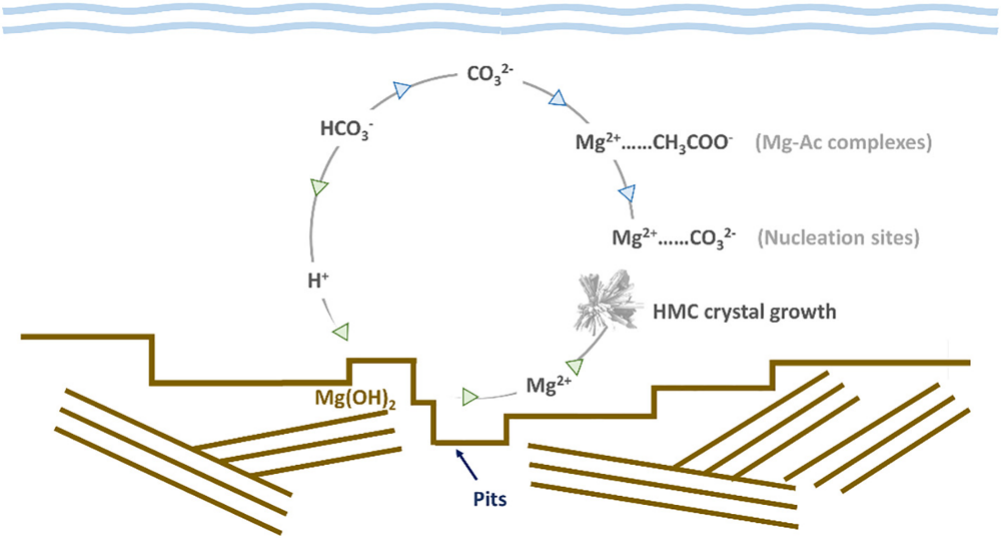
A schematic reaction pathway of brucite in aqueous environment with Mg–Acetate. Reproduced with permission from ref (343). Copyright 2022 Elsevier.

Incorporating additives, so-called hydrating agents, into MC (e.g., magnesium acetate) resulted in strengths 107% and 53% higher than those observed in MgO and PC concrete samples, respectively, during carbonation curing.^338^ The increase in the carbonation reaction was attributed to the higher degree of MgO hydration (as explained in the introductory part of this section), and to an enhancement in the CO_2_ sequestration ability of brucite. The higher amount of HMCs formed resulted in a denser matrix and an improved performance.^338^ To exclusively explore the promotion of the carbonation reaction by acetate, a recent study employed brucite as the starting material.^343^ Mg-acetate significantly accelerates carbonation, an effect attributed to the promotion of brucite dissolution^344^ and the subsequent formation of Mg-acetate complexes. These complexes would act as nucleation centers for HCMs, far from the brucite surface, allowing for the continuous progress of the carbonation reaction while preventing surface passivation (Figure 45).^343^ A more recent study by the same group has shown that the acetate alters the hydration product from MgO, yielding a nanocrystalline Mg(OH)_2_.^345^ This might be key for the enhanced effect on the subsequent carbonation, as it might be more prone to dissolution due to its high surface area. Furthermore, the carbonated products obtained in the presence of acetate were identified as amorphous (hydrated) magnesium carbonates and giorgiosite, distinct from nesquehonite, typically obtained in the absence of the ligand.^345^

Recent advancements in understanding MC at a fundamental level position them not only for utilization under carbonation curing but also in ambient conditions, broadening their potential applications.^334,339,346^ Compared to other MgO-based cements, in our view, MC and magnesium silicate cements offer the most favorable possibilities. However, much of the research on MC is relatively recent, leaving several fundamental questions unanswered. First, a critical research priority should focus on elucidating the precise nature of the HMCs phases formed, particularly regarding their amorphousness and stability. Understanding the formed phases is crucial since their transformation into more stable forms can lead to undesirable consequences such as CO_2_ and H_2_O release, causing binder weakening due to chemical composition, volume, and morphology changes. Second, the proposed mechanisms through which carboxylic-based additives influence HMCs crystallization require confirmation. Validating these mechanisms could enhance carbonation using alternative organic additives. In this sense, starting with the smallest species, thoroughly characterizing the ion associates formed in solution in the presence of organics (including density, size, molecular weight, and hydration state) would provide valuable insights to accelerate carbonation processes and, thus, the strength development of MC.

#### 4.3.2 Magnesium Silicate (MS) Cement

Magnesium silicate (MS) cement shows promise and has potential as an alternative binding material. Magnesium silicate hydrate (M–S–H), the phase responsible for the cohesion of the material, is formed through the water-mediated reaction between MgO and a silica source, which also produces brucite. The concept of this type of cement, which might be seen at first sight as analogous to calcium silicate cement, is almost as old as PC itself, existing since the end of the 1800s.^347^ In the 1950s, M–S–H was labeled as a “non-cementitious” phase due to its association with the loss of strength in degraded concrete in marine environments.^348,349^ Since the 2000s, a more systematic research approach on M–S–H has facilitated a deeper understanding of its structure and properties, revealing the conditions to obtain an adequate material. Consequently, various potential applications of MS cement have been demonstrated, including producing refractory castables,^350−352^ and the immobilization of nuclear waste due to its lower pH compared to PC (pH ∼ 10).^353−356^ However, numerous questions and challenges remain regarding the use of magnesium silicate cements. In line with the previous sections, we will focus on the formation mechanism as well as the nano- and microscale characteristics of M–S–H. For a more complete understanding of magnesium silicate cements, covering aspects not discussed here, such as mechanical properties, durability, thermodynamic modeling, and M–S–H phase stability aspects, we recommend the excellent review by Sreenivasan et al. published in April 2024.^357^

MS is produced in most studies by using MgO and microsilica as precursors and admixtures that reduce the high-water demand of Mg-based binders, as already discussed. The slow setting at ambient temperature, which prevents practical onsite and large-scale applications of magnesium silicate binders, has been attributed to the slow dissolution of brucite in the presence of M–S–H.^352,358^

In pastes created by mixing MgO and microsilica with water/solid (w/s) ratio equals 0.5, no M–S–H was identified during the initial 3 days, where brucite and MgO were the main phases. Only after 10 days M–S–H started to appear. Within 30 days, the brucite and magnesium oxide content decreased while the M–S–H phase increased. Even after 180 days of aging, traces of brucite were still detectable in the samples, where M–S–H constituted the primary phase.^352^ By increasing the w/s ratio to 5, Li et al.^359^ showed that in the initial 3 days, only brucite formed rapidly due to the faster dissolution of MgO compared to microsilica. M–S–H became detectable after 3 days, showing higher thermodynamic stability than brucite; therefore, its formation consumes brucite. After 28 days, even in samples with low MgO content, XRD analysis revealed the presence of brucite and MgO. Longer durations (90 days) indicated almost complete consumption of MgO in those samples, although traces of brucite remained. Samples with high MgO content retained high levels of periclase and brucite after 90 days.

Bernard et al.,^358^ used a w/s of 45, considerably higher than any other existing study, and in this case, the hydration reaction of periclase was not a limiting step. The authors showed that MgO reacted completely within 1 day, resulting in brucite, M–S–H, and unreacted silica. Even after 3 months, brucite persisted in both Mg/Si ratios tested, even though elemental analysis of the solutions indicated an undersaturation with respect to brucite already 2 days into the curing time. The presence of brucite in this undersaturated environment suggests the existence of a kinetic barrier that hinders its dissolution, thus limiting the formation of M–S–H. This persistence of brucite could be beneficial for capturing CO_2_ in the form of magnesium carbonates, resulting in increased mechanical properties of the magnesium silicate binders.^360^ Notably, transformations in the precipitated M–S–H with time have been observed. After 3 months, a transient phase of M–S–H was observed, which showed a lower degree of organization (assessed by NMR) than the final product and an Mg/Si ratio close to 1. This metastable M–S–H phase seems to evolve toward a more ordered structure over longer periods.^358^

**Figure 46:**
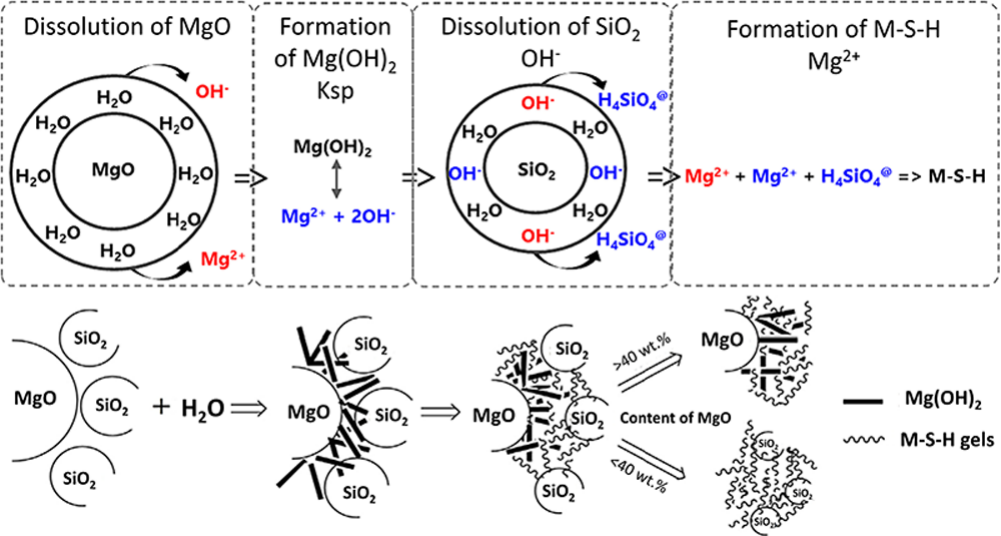
A schematic representation of M–S–H formation in pastes. Reproduced from ref (357) under a 4.0 Creative Commons Attribution License (CC BY 4.0). Copyright 2024 Elsevier. https://creativecommons.org/licenses/by/4.0/.

On the basis of the existing literature, the formation of M–S–H using MgO and microsilica solids mixed in the presence of solids can be described through the following stages: (i) dissolution of MgO, (ii) formation of brucite, (iii) dissolution of brucite in the presence of silicate species, (iv) formation of a transient M–S–H phase (lower degree of ordering), (v) transformation into the final M–S–H with a high degree of ordering (Figure 46).^357^

**Figure 47:**
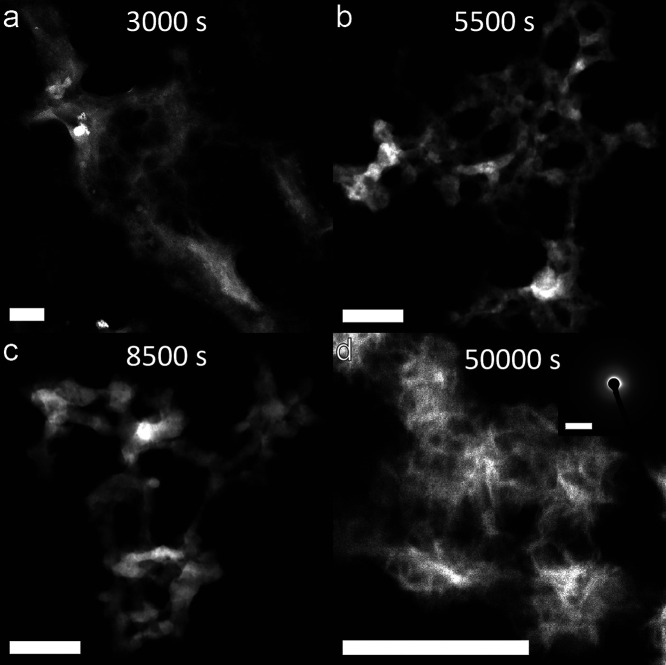
High-Angle Annular Dark-Field Scanning Transmission Electron Microscopy (HAADF-STEM) images of samples obtained at various time points during the synthetic precipitation of M–S–H using sodium metasilicate and magnesium chloride solution, depicted at (a) 3000 s, (b) 5500 s, (c) 8500 s, and (d) the final material. The scale bar in the inset is 5 nm, and the scale bars in the images represent 100 nm. Reproduced with permission from ref (195). Copyright 2023 American Chemical Society.

With respect to the nucleation of M–S–H from magnesium and silicate species in solution, the first fundamental steps have been taken to understand the pathway of M–S–H formation in synthetic systems.^195^ Our research revealed a unique multistep pathway in which a complex mixture of defined hydrated magnesium-(sodium)-silicate oligomers exist in the solution before nucleation, similarly to the C–S–H system.^9,201^ These oligomers seem to aggregate, yielding an ill-defined M–S–H precursor phase, which eventually transforms into a more compact M–S–H network consisting of nanoglobular particles with distinct sheet-like surfaces (Figure 47). Identifying these oligomeric silicate species prior to nucleation is of paramount significance for future control over M–S–H formation, including its nanostructure, through a bottom-up approach.^195^

**Figure 48:**
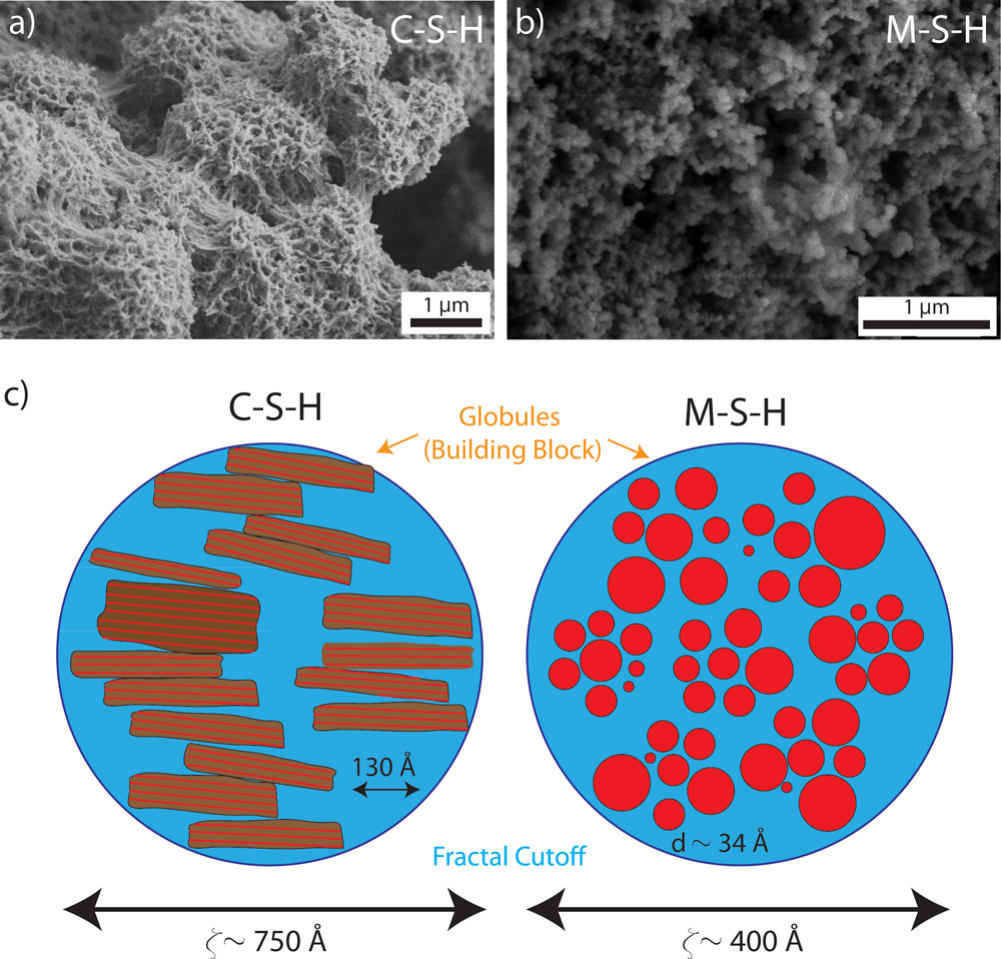
SEM images of synthetic a) calcium silicate hydrate (C–S–H) and b) magnesium silicate hydrates. Images courtesy of Christian Debus and Maximilian Marsiske (PhD candidate at the University of Konstanz). c) Comparison of microstructures of C–S–H and M–S–H. Modified from ref (364). Copyright 2014 Royal Society of Chemistry.

Regarding the chemical and crystallographic structure of this material, M–S–H is considered to consist of multiple amorphous hydrate magnesium silicate phases. It presents a layered silicate structure akin to clays, in which the silicate units are bonded to two or three neighboring ones (Q^2^ and Q^3^).^361^ This differs significantly from the C–S–H, where Q^1^ and Q^2^ dominate the structure.^53^ Whether the phase has a talc-like or serpentine-like character depends on the Mg/Si ratio in the material.^358,362,363^ Electron microscopy and wide- and small-angle X-ray scattering revealed significant differences in the nano- and microstructure of synthetic C–S–H and M–S–H.^364^ In C–S–H, a disk-like “globule” was identified as the primary unit (see Section 2.2), while for M–S–H, a spherical globule was recognized (Figure 48). This key difference in the basic building blocks results in distinct microstructures, i.e., sheet-like objects arranged in a laminar pattern in C–S–H versus densely packed spherical particles in M–S–H. Consequently, the mechanical properties and durability of both types of cements differ.^364^

The slow reaction between MgO and SiO_2_ at ambient temperatures results in slow strength development.^352,358,365^ A problem that could be partially addressed using dissolved phosphates,^366,367^ and carbonates^368^ that accelerate M–S–H gel formation. Magnesium carbonate^369^ and magnesium phosphate^367^ complexes formed in solution have been suggested as responsible for the enhanced dissolution of Mg(OH)_2_. In the case of phosphates additives, they also reduced the water demand, which translated into lower porosity and compressive strengths comparable to PC.^348,370^ Another interesting approach developed by the Unluer research group used hydromagnesite seeds to accelerate M–S–H formation, most likely by providing extra surfaces for gel formation. This method resulted in a denser microstructure and demonstrated higher compressive strength values compared to the absence of seeds.^217,369^

**Figure 49:**
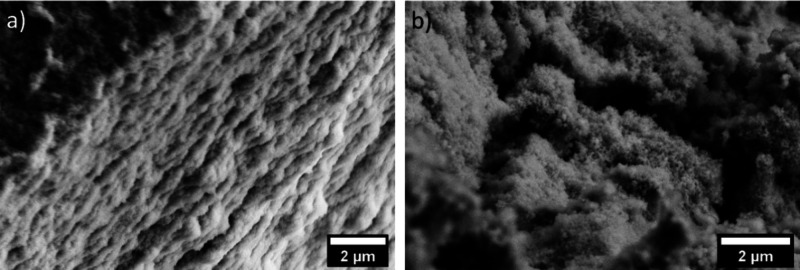
M–S–H synthetic precipitates were obtained under two conditions: a) in the presence of a phosphate-based additive (100 mg/L) and b) in its absence. The addition of the phosphate-based additive resulted in a more compact material with an apparent layered structure. Images courtesy of Annika Bastian, PhD candidate at the University of Konstanz.

Notably, in our ongoing investigations, we have observed that the introduction of dissolved phosphates slightly delays the nucleation of M–S–H, even at relatively low concentrations ranging from 10 mg/L to 200 mg/L. This validates that the improvement in M–S–H formation when using phosphate and carbonate additives is not attributed to an influence on M–S–H nucleation per se but rather to an increase in brucite dissolution. In addition, the resultant M–S–H material obtained in our study after 16 h considerably differs from the reference material (Figure 49). In the presence of the phosphate-based additive, the packing of M–S–H globular particles results in a more compact material characterized by reduced porosity, which resembles an ordered layered structure (Figure 49). This is also in line with the documented high compressive strengths of approximately 70 MPa at 28 days achieved for M–S–H through the introduction of P-based additives, effectively reducing water demand and, consequently, porosity.^353^ In this context, our recent results are promising as they serve as a proof of concept for the application of additive-controlled crystallization to the M–S–H system, potentially producing hierarchically ordered structures with reduced porosity, which could improve their compressive strength. To our knowledge, this avenue has not been explored for MS cements.

In conclusion, while MS cements show promise not only in specific applications such as hazardous waste encapsulation, refractory castables, and clay stabilization but also in the production of structural and nonstructural building components due to their reported high strength development, they still face limitations such as slow ambient setting and high-water demand, which hinder their wider use.^371^ Despite these challenges, extensive research efforts dedicated to MS cements over the past decade have significantly improved our understanding of the material and proposed potential solutions to some of these hindrances.^357^ This progress indicates that with ongoing research, investment, and exploration, MS cements could have practical applications in current construction practices.

In this sense, we propose the most important research challenges within the scope of this review. Regarding the primary stages of M–S–H formation, future research should first investigate the role and evolution of the oligomeric species, the liquid/solid character of the precursor phase, and its transformation into the final sheet-like compact material. Considering this M–S–H crystallization pathway, the acceleration of the setting by using additives needs to be revised. The nature of Mg–Si species in solution has been shown to be much more complex than simple ion pairs,^195^ and consequently, their interactions with carbonate and phosphate may also be more complex. Furthermore, the interaction between additives and the liquid/solid precursor phases of M–S–H deserves close examination. In this regard, it is critical to conduct a systematic investigation to understand the effect of additives on M–S–H formation and to identify the most appropriate additives for this system. Finally, attention should be paid to improving the nano- and microstructure, which could lead to improved mechanical performance. In this context, controlling the crystallization of M–S–H, e.g., via additives, stands as a promising strategy worthy of further investigation.

#### 4.3.3 Magnesium Oxychloride, Oxysulfate, and Phosphate Cements

In this section, we will introduce another three types of MgO-based cement: magnesium oxychloride cements, magnesium oxysulfate cements, and magnesium phosphate cements. We have decided to group them together because, despite their good mechanical performance and specific applications in fire protection, insulating materials, and repair cements, their widespread use is hindered by difficulties accessing raw materials. Furthermore, magnesium oxychloride and oxysulfate cements are susceptible to degradation in water environments, further restricting their usage. It should be noted that, unlike in the case of MC and MS cement, the reactions leading to hydrate formation generally occur at lower pH levels. This favors the dissolution of MgO while inhibiting the formation of the brucite passivation layer, thereby facilitating the formation of cementing phases.

##### 4.3.3.1 Magnesium Oxychloride Cements (MOC)

Magnesium oxychloride cements (MOC), also known as Sorel cements,^372^ are formed through the precipitation of magnesium chloride salts (xMg(OH)_2_-yMgCl_2_-zH_2_O) when MgO is mixed with filler materials and a concentrated MgCl_2_ solution. This reaction is fast and highly exothermic. Therefore, selecting an appropriate reactivity level for MgO is crucial to strike a balance between achieving high early strength and maintaining a practical working time.^373^

MOC exhibits remarkable characteristics, including rapid setting, fire resistance, low thermal conductivity, good resistance to abrasion, and compatibility with a wide range of fillers, making it a compelling material.^335^ However, they present a major drawback related to the incompatibility with humid environments.

During the setting of MOC at ambient temperatures, the phase assemblage includes brucite (Mg(OH)_2_) and two crystalline phases: phase 3 (3Mg(OH)_2_-MgCl_2_-8H_2_O) and phase 5 (5Mg(OH)_2_-MgCl_2_-8H_2_O). The binding phases formed highly depend on the reactivity of the MgO used, the concentration of the MgCl_2_ solution, the molar ratio of MgO/MgCl_2_, and the curing temperature.^374−376^ Concerning the crystallization mechanism of the hydrates, polynuclear complexes [Mg_*x*_(OH)_*y*_(H_2_O)_*z*_]^2*x–y*^ of uncertain composition are formed in solution.^377^ These complexes further react with Cl^–^ ions and H_2_O, yielding the formation of an amorphous precursor hydrogel,^378^ which is proposed as responsible for the initial setting of the paste.^379^ As this amorphous phase transforms into MOC crystalline phases, a continuous network of crystals of 5-phase and 3-phase forms, which is accountable for paste hardening.^377^

The microstructure of MOC cements is characterized by an interconnected network of bundled needle-shaped and plate-like crystals of the two phases.^380^ This network governs the strength development of the hardened material,^381^ and the consequent superior mechanical properties compared with a PC with the same porosity.^376^ The strength primarily arises from the presence of the 5-phase, which has been shown to translate into higher compressive strengths.^374,382^ In contrast, MOCs where the 3-phase dominates exhibit lower strength and higher water solubility.^383^ The 5-phase is metastable and gradually transforms into the most stable 3-phase,^376^ which subsequently decomposed into brucite, leading to volume instability in the material.^384^ This phase transition is the underlying cause of MOC’s poor water resistance, significantly limiting its application in an engineering context.

Numerous methods have been used to stabilize the 5-phase in MOC, involving the addition of trace amounts of inorganic (e.g., phosphates, gypsum, carbonates) and organic substances (e.g., tartaric acid, ethylene-vinyl acetate, and steric acid-styrene copolymers), with some of those showing promising perspectives.^384^ Notably, tartaric and phosphoric acids have been demonstrated to increase the lifespan of the amorphous phase significantly.^385^ This extension contributes to improved water resistance by interlocking the 5-phase crystals in the matrix, hindering their decomposition, and reducing the pore volume.^385^ This again highlights the importance of understanding the formation process of the different hydrates, as additives can interact with precursor phases and alter the hydration reaction. To ensure long-term stability in the MOC paste, it is imperative to address the stability of the hydration products in water. While some progress has been made in temporarily stabilizing the 5-phase, it remains susceptible to degradation, ultimately compromising the integrity of MOC-based materials.

##### 4.3.3.2 Magnesium Oxysulfate (MOS) Cement

Magnesium oxysulfate (MOS) cement, akin to MOC cement, is produced using MgSO_4_ instead of MgCl_2_ and MgO. Both share similar attributes, such as rapid hardening, good fire resistance, low thermal conductivity, and good abrasion resistance. MOS cement has the added advantage of reduced susceptibility to metal corrosion because of the exclusion of chlorides but suffers from poorer mechanical performance.^386^ Moreover, at low concentrations of MgSO_4_, only the Mg(OH)_2_ phase is present, causing limited water resistance and poor properties.^382^

**Figure 50:**
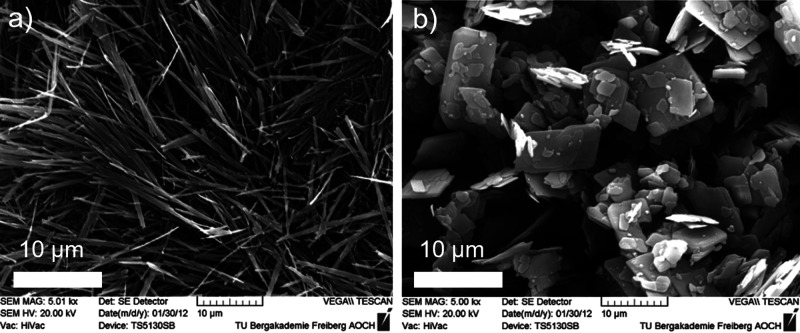
SEM images of magnesium oxysulfate a) 5–1–2 and b) 3–1–8 phases. Reproduced with permission from ref (388). Copyright 2013 John Wiley & Sons.

MOS and MOC cements have many similarities concerning their hydration process and binding phases. The 3–1–8 (3Mg(OH)_2_-MgSO_4_-8H_2_O) and 5–1–3 (5Mg(OH)_2_-MgSO_4_-3H_2_O) are the major ones formed at ambient temperatures in MOS cements without additives.^382^ However, other phases can also formed, for instance, using higher temperatures or hydrothermal synthesis conditions.^387^ Regarding stability, high-resolution synchrotron X-ray diffraction has suggested that in the case of MOS, the 3-phase is metastable at room temperature. Its transformation to the 5-phase, the most stable phase at 25 °C, may decrease the long-term strength of the cement.^388^ The microstructure of the two phases differs considerably. The 5-phase is characterized by long needles, while the 3-phase shapes as tabular crystals (Figure 50).^388^ The needle-like microstructure found in the 5-phase leads to a higher space-filling, making it the more favorable phase for strength development.

During the hydration process under ambient conditions, brucite is the major phase present at 28 days, accompanied by an amorphous phase of unknown composition and a small fraction of 5-phase.^389^ Nevertheless, when organic additives like sodium citrate or citric acid are introduced, the quantity of brucite is considerably reduced, and 5-phase becomes more prevalent. This alteration leads to the interlocking of the amorphous phase and the 5-phase, resulting in a denser matrix with improved water resistance,^389^ akin to the behavior observed in the MOC system. Various studies have consistently demonstrated that the use of organic additives such as citric acid,^390^ and tartaric acid,^391^ promotes the formation of the 5-phase, yielding to better mechanical properties, at the cost of longer setting times.^391^ To mitigate this drawback, researchers have explored using seed particles as templates for the nucleation of the 5-phase.^392^

##### 4.3.3.3 Magnesium Phosphate (MP) Cements

Magnesium phosphate (MP) cements have the best mechanical performance among known MgO-based cements.^393^ They are of interest to the construction industry for their fast setting, good bonding to old concrete, high-temperature stability, and durability.^394^ They can be used for low-volume and specialized nonsteel reinforced applications such as structural rehabilitation and repair, hazardous and radioactive waste stabilizers, industrial fire protection coatings, and dental and prosthetic cements due to their fast setting and high strength.^395^

MP hardens at room temperature through the aqueous reaction between MgO and a phosphate salt. The bonding phases form through the acid–base reaction between a metal cation (Mg) and a phosphate anion. Among MP systems, the MgO-KH_2_PO_4_ combination is preferred for its controlled reaction, yielding a more homogeneous microstructure and enhanced mechanical properties.^396^ Still, the acid–base reaction is fast and highly exothermic, necessitating the addition of retardant agents to regulate their formation.^397^

**Figure 51:**
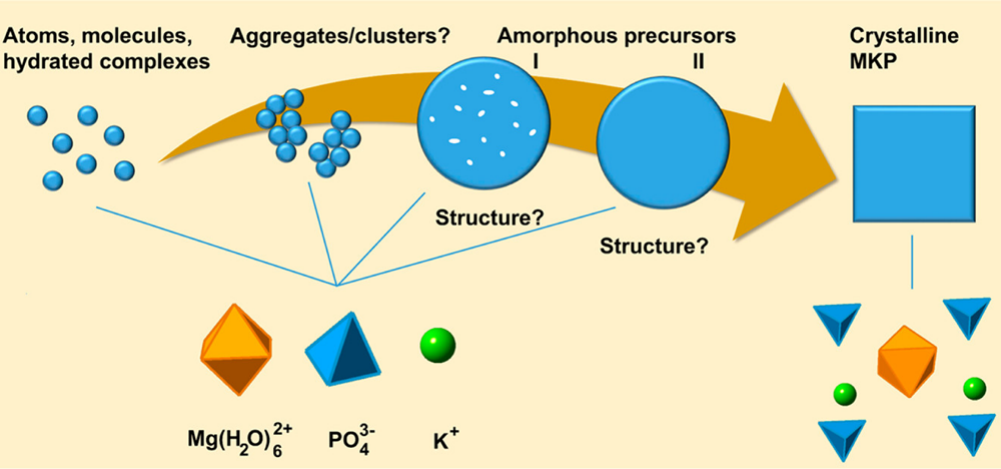
Scheme illustrating the proposed different stages of the MP setting reaction, emphasizing open questions. Reproduced with permission from ref (196). Copyright 2017 American Chemical Society.

Regarding the mineralogical composition of the hydrated phases in MP, the main phase formed appears to be struvite (MgNH_4_PO_4_·6H_2_O) or K-struvite (MgKPO_4_·6H_2_O), along with minor phosphate phases like NaNH_4_HPO_4_·4H_2_O (stercorite), MgHPO_4_·3H_2_O (newberyite), and brucite. Viani’s group has investigated the early stages of K-struvite formation in the MPC.^196,398,399^ They propose a multistep crystallization pathway (Figure 51) where hydrated complexes containing Mg^2+^ and PO_4_^3–^ are combined to form a first type of amorphous precursor (I), which converts to a second type of amorphous (II) phase with higher water content. Subsequently, this amorphous phase evolves to the crystalline K-struvite.^196^ These amorphous phases have been suggested to exist initially as nanoparticles, which further aggregate into larger domains and eventually form K-struvite.^400^ The transformation of these amorphous phases into K-struvite is highly relevant, as it seems to drive the development of mechanical properties.^401,402^ Nevertheless, this process is not yet fully understood.

**Figure 52:**
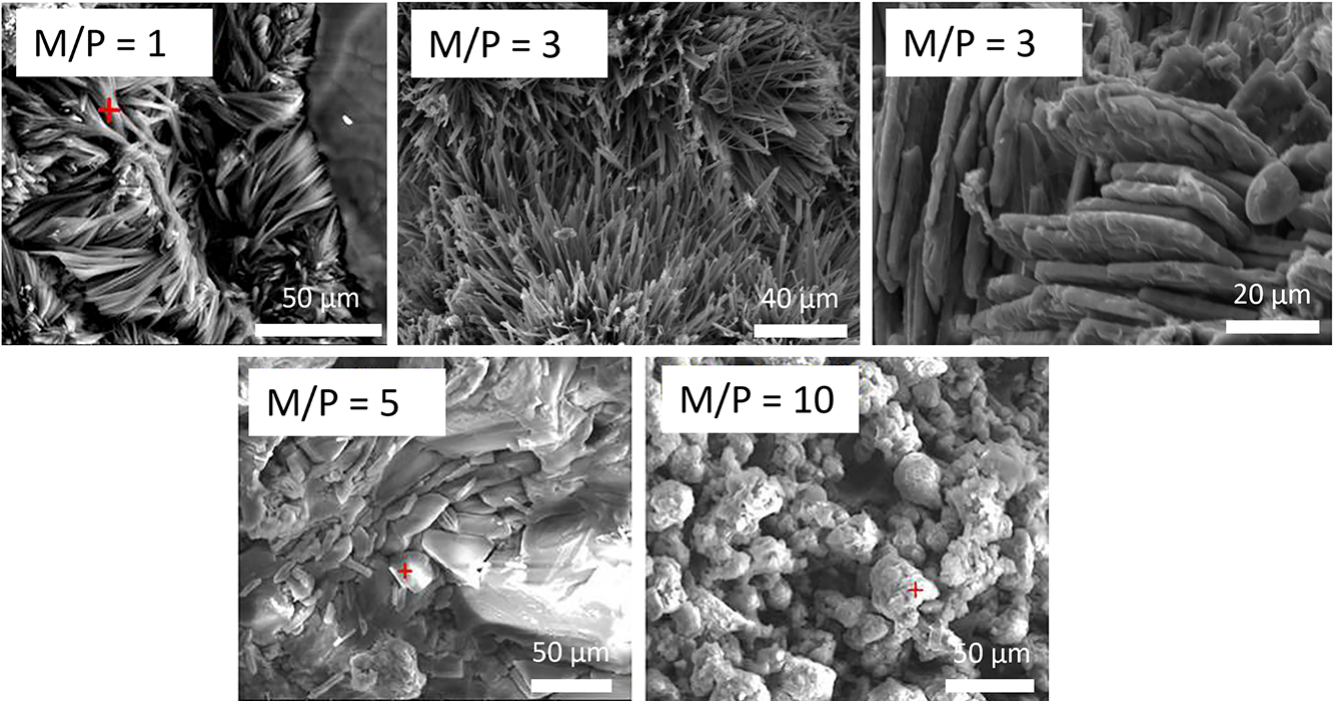
SEM micrographs of the pastes produced by mixing magnesia and potassium dihydrogen phosphate, with various M/P ratios (90 d, w/b = 0.20). Reproduced with permission from ref (403). Copyright 2017 Elsevier.

The microstructure of the cement after 28 days appears compact and dominated by a network of K-struvite crystals occupying the entire volume.^402^ Struvite typically exhibits a rod-like elongated cubical structure. Nevertheless, the morphology within the paste (Figure 52) is highly dependent on the blend’s composition, including factors such as the pH, Mg/P ratio, MgO reactivity, diluents, and setting retarders.^397^ This can lead to various crystal forms, ranging from acicular polycrystals and needle-like crystals to tabular crystals and products without a defined shape.^403,404^ Pastes produced with lower Mg/P ratios tend to exhibit lower compressive strength, higher porosity, and reduced water resistance. On the contrary, tabular crystals obtained with higher Mg/P ratios (>5) offer excellent mechanical properties and stability against water-induced degradation.^403^ This emphasizes that not only the phases formed but also their morphology influences the paste’s microstructure and, hence, its properties.

##### 4.3.3.4 Future Research Challenges for MOC, MOS, and MP

Even if their widespread adoption does not materialize due to restricted feedstocks’ material-based drawbacks, there is still potential for their future and current application in repair cement or as fire protection elements if some of the barriers explained above are addressed. This application has the added benefit of extending the lifespan of structures, reducing their overall CO_2_ footprint. In terms of material limitations, the two main drawbacks are the fast setting of the three systems and the improvement of the water resistance of MOC and MOS. In our opinion, a deeper understanding of the crystallization mechanisms of the three systems could help solve these problems.

The formation mechanism of the relevant phases in MOC, MOS, and MP cements has been revealed as a complex multistep process involving different precursor phases. Considering the presence of polynuclear complexes in MOC and their hypothetical occurrence in MP, we assume that similar complexes may be involved in the formation of MOS. A thorough characterization of these ionic associates in all three systems is recommended, as details such as charge, and water content may be critical to mitigate rapid curing with additives. Similarly, the amorphous phase identified in the three systems should be better investigated, focusing on aspects such as composition, formation process, and stability. This research is relevant for the selection of appropriate additives capable of interacting with the ionic associates and/or amorphous phases, thereby inhibiting crystallization and effectively retarding the setting of the cements.

Moreover, the transition from the amorphous precursor to the different crystalline phases is a fundamental step that deserves special attention, given its role in the formation of a continuous crystalline network that is crucial for the hardening of the paste. In the case of MOC and MOS, where metastable and stable phases coexist in the paste, the mechanical properties and water resistance of the metastable phases seem preferable. Therefore, ensuring the stabilization of these metastable phases is key to obtaining a competitive material. A promising approach that has been shown to be effective also involves additives; however, their mode of action remains poorly understood, which hinders the optimization of cement properties by selecting the most appropriate additives. To gain a deeper understanding of additive-controlled crystallization, we encourage the reader to continue reading this review as the next section provides an overview of the fundamental principles underlying this concept.

## 5 (Organic) Additive Controlled Crystallization

Many strategies exist for manipulating crystallization through additives, with a comprehensive examination of techniques to control mineral morphologies and structures provided by Meldrum and Cölfen in this journal.^405^ In this Section of the review, our focus is specifically on elucidating the fundamental principles underlying additive-controlled crystallization. It is important to highlight that within the classical view of crystallization, the effect of additives is mainly limited to interactions with the free ions in solutions, incorporation into the nascent phase, and adsorption of additives on nascent nuclei.^128^ If one considers multistep nonclassical crystallization pathways, there are several mechanisms for additive control.

**Figure 53:**
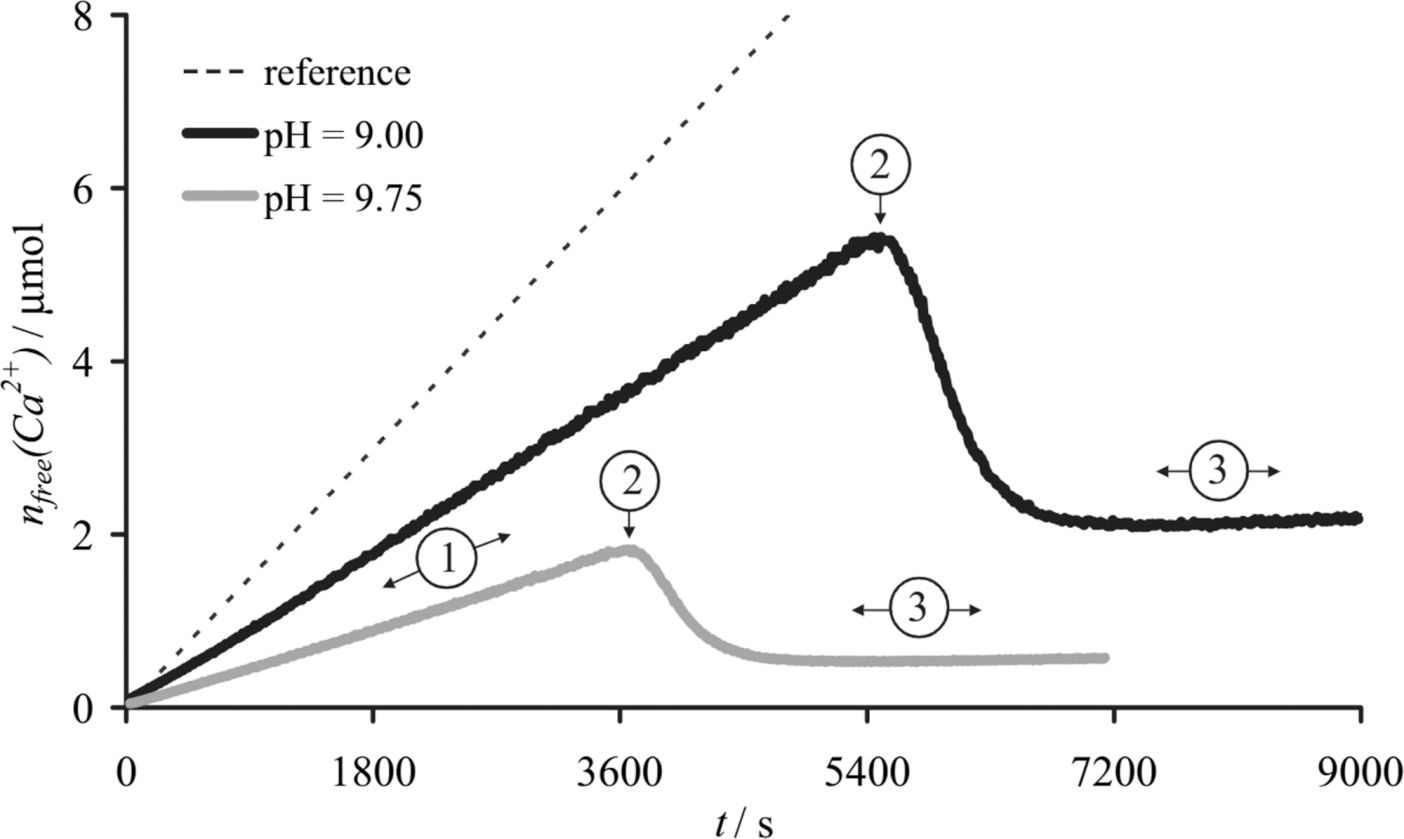
Time development of free calcium ions measured by a calcium ion-selective electrode (Ca-ISE) in carbonate buffer at pH 9.00 and 9.75. The reference line reflects the amount of calcium ions added. “1” depicts the prenucleation stage, “2” the nucleation event, and “3” the particle growth stage. Reproduced with permission from ref (129). Copyright 2009 John Wiley & Sons.

Additives exert a significant influence on the crystallization process from the very beginning. This influence can manifest itself as early as the prenucleation stage, where complexation of ions such as Ca^2+^ occurs through interaction with, for example, acidic macromolecules. In addition, additives can play a key role in influencing the prenucleation cluster (PNC) equilibrium. A detailed study of this phenomenon has been carried out in the context of CaCO_3_, using a titration methodology easily adaptable to the C–S–H system.^11^Figure 53 represents a typical titration experiment involving the dosing of Ca^2+^ into a carbonate buffer at a specified pH.^129^

This experimental approach makes the distinct stages of prenucleation (1), nucleation (2), and postnucleation or particle growth readily discernible. In the illustrated example in Figure 53, observable variations in free Ca^2+^ concentrations between the two pH values are evident. The dashed line in the graph represents the time-dependent amount of Ca^2+^ added. Notably, the detected amount of free Ca^2+^ in solution is significantly lower as a function of pH, which directly reflects the concentration of Ca^2+^ bound in ion pairs, prenucleation clusters (PNCs), and similar entities. Therefore, even in the absence of additives, this simple titration curve shows clear differences at different pH values. A more detailed analysis of these curves, taking into account the ionic activity, indicates that the CaCO_3_ species bound in the prenucleation stage cannot be considered inactive during nucleation but seem to play a key role in the phase separation process.^406^

**Figure 54:**
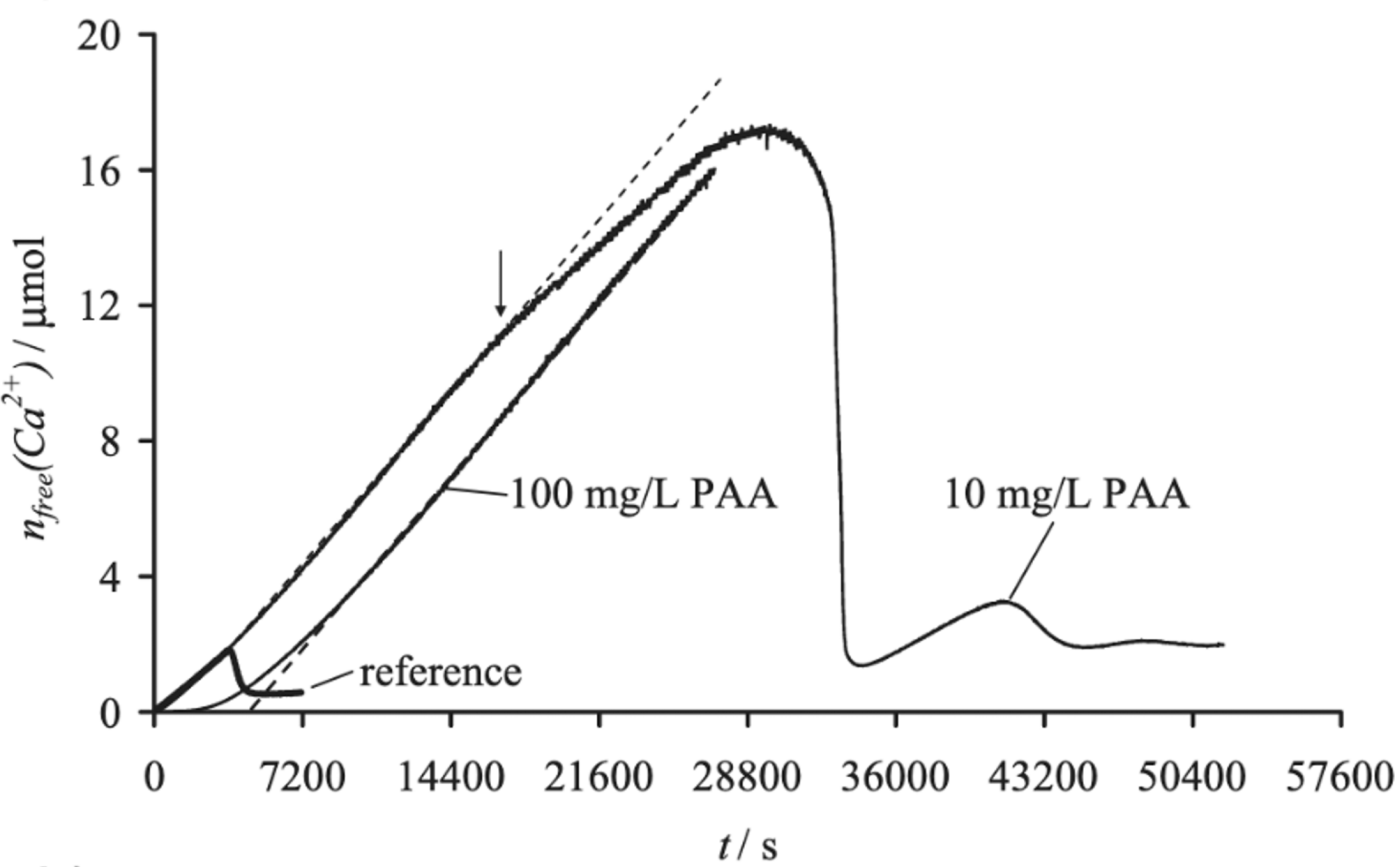
Time development of the amount of free calcium ions under different experimental conditions: in the absence of PAA as a reference and in the presence of 100 mg/L and 10 mg L^1^ PAA at pH 9.75. Reproduced with permission from ref (129). Copyright 2009 John Wiley & Sons.

The resulting curves show a marked divergence when additives are introduced into this system, as illustrated in Figure 54. Subsequent investigation revealed that these titration curves exhibit specificity corresponding to different additives. This characteristic makes them a valuable fingerprinting technique for the categorization of additives, as demonstrated by Verch et al.^407^ As an illustration, when analyzing the curves associated with poly(acrylic acid) (PAA), a recognized scale inhibitor known to interact with Ca^2+^ ions, alterations of the free Ca^2+^ curves become evident when contrasted with reference experiments performed without additive. When introduced into the system, PAA causes a substantial delay in the nucleation onset and a significant rise in the Ca^2+^ concentration at which nucleation occurs, even at low concentrations such as 10 ppm (Figure 54). This pronounced impact on nucleation aligns with the anticipated behavior of a nucleation inhibitor. Notably, a secondary nucleation event is observed in the case of the 10-ppm experiment. This suggests that after the initial nucleation event, PAA must be released to control a second nucleation event. Extrapolating the linear portion of the titration curves back to 0 Ca^2+^ makes it possible to determine the Ca^2+^ binding of the additive, given the known Ca^2+^ addition rate. Additionally, in Figure 54, the arrow highlights a sudden change in slope during the prenucleation stage. The reduced slope suggests a sudden increase in the bound Ca^2+^, which is likely to occur in prenucleation clusters (PNCs).^129^

This simple experiment within a CaCO_3_ crystallization assay facilitates the classification of additives into five distinct types, as outlined below:^129^

- Type I: adsorption/complexation of calcium ions.
- Type II: influence on soluble-cluster formation and equilibria.
- Type III: inhibition of nucleation of a precipitated nanoparticle phase.
- Type IV: adsorption on nucleated particles and their stabilization.
- Type V: influence on the local structure of nucleated particles, that is, type of amorphous phase or crystalline polymorph.

Four further types of crystallization additives characterize the later stages of crystal growth,^172,408−412^ both classical and nonclassical, which will be discussed further below:

- Type VI: influence on the nanocrystal shape by face-specific adsorption.
- Type VII: influence on the oriented attachment and vectorial alignment of nanoparticles by modifying the mutual interparticular interaction potentials.
- Type VIII: stabilization of the resulting mesocrystals^126,413^ against Ostwald ripening and recrystallization, thus stabilizing the hybrid material.
- Type IX: mechanical reinforcement or toughness increase of the crystal and modifier phase to constitute a beneficial biomaterial hybrid.^172,412,414,415^

**Figure 55:**
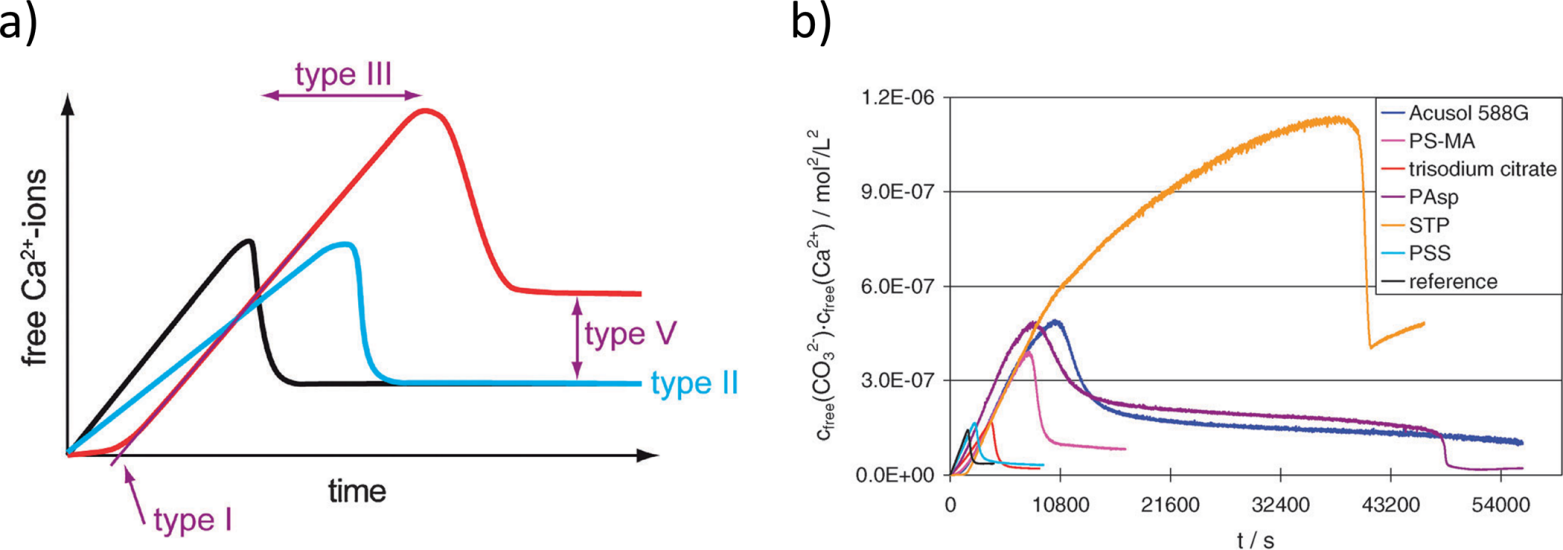
(a) Schematic illustration of the additive interactions detectable through the titration experiments. The black and blue curves represent experiments in the absence of additives and the presence of a solely type II active additive, respectively. The red graph shows the types I, III, and V additives. (b) Overlay of the developments of the amount of free Ca^2+^ ions in the presence of various studied additives at pH 9.75. All negatively charged additives have a concentration of 100 mg/L. Reproduced with permission from ref (407). Copyright 2011 Royal Society of Chemistry.

Identifying types I, II, III, and V from the titration curves, as depicted in Figure 55a, is straightforward. Figure 55b shows further distinctions between different additives, highlighting the effectiveness of these simple titration experiments in examining the role of different stages of crystallization. Notably, a single additive can play multiple roles simultaneously or at different stages of prenucleation, nucleation, and crystallization events. Consequently, these experiments serve as a robust fingerprinting technique for additive classification.^407^

The outcome of a nucleation event is not limited to a solid product; it can also manifest as a liquid, as we saw in Section 3.2, serving as an ideal precursor for infiltrating nanospaces in templates such as collagen fibrils or adapting the morphology of any given template. An insightful commentary by Gebauer discusses how additives exert control over the early stages of mineralization.^128^ In this context, we explore potential scenarios arising from the prenucleation cluster (PNC) pathway in the presence of additives. The following discussion presents the most relevant findings critical to understanding how additives affect nucleation and subsequent products.

**Figure 56:**
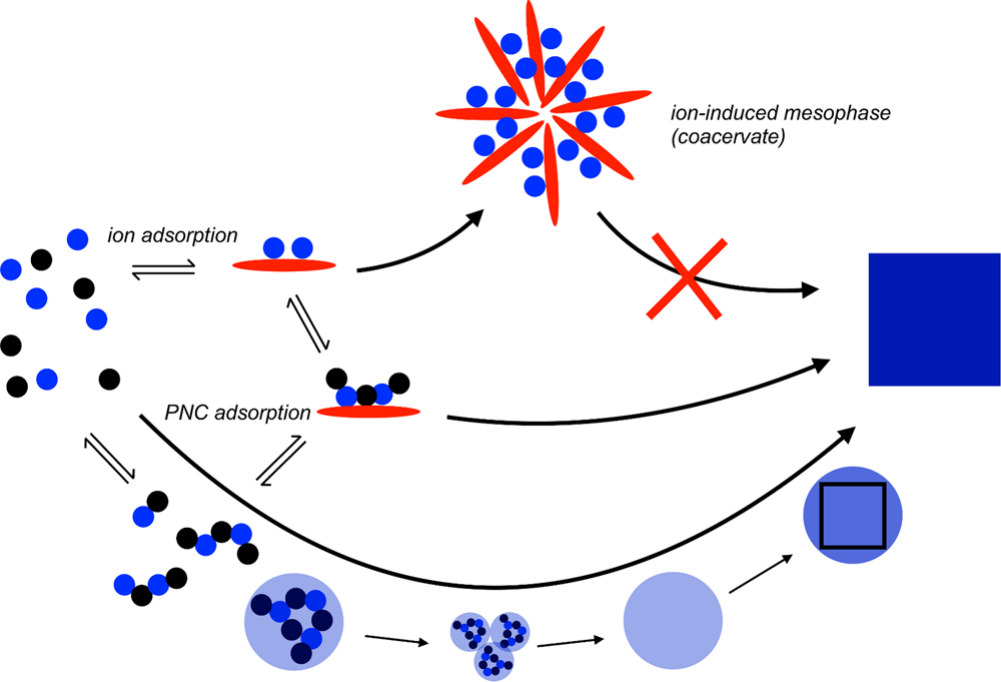
Schematic illustration of the mechanism of nucleation according to the PNC pathway with potential effects of ion (top) and PNC (middle) adsorption by an additive (red ellipsoid). For strong ion-additive interactions and/or high additive concentrations, the formation of coacervates is expected (if the additive chemistry and structure allow), which inhibits mineral formation entirely. Corresponding size regimes span the whole colloidal domain depending on the type of mesophase formed.^416^ For weaker ion-additive interactions and/or low additive concentrations, PNC binding by the additive (middle) becomes probable, and the process can proceed toward particle formation. Reproduced from ref (128) under a 4.0 Creative Commons Attribution License (CC BY 4.0). Copyright 2018 MPDI. http://creativecommons.org/licenses/by/4.0/.

Figure 56 illustrates the PNC pathway (lower part of the image), showing the potential interactions of the ions and PNCs with an additive, for example, Ca^2+^ or CaCO_3_–PNCs with PAA. While one pathway leads to the formation of the final crystal, which is also the end product of the PNC pathway, there is a notable possibility that ions, and consequently the PNCs—in equilibrium with the ions—may come together to form a coacervate. In this context, a coacervate is a liquid phase resulting from electrostatic interactions between the polymer and ions, producing a highly swollen liquid or hydrogel structure whose properties depend on the molar mass of the polymer and the ion-polymer ratio. It is important to note that thermodynamically stable coacervates lack the counterions necessary for crystallization, which distinguishes them from polymer-induced liquid precursors (PILPs).^139^ Although PILPs are also in a liquid state, they can be difficult to distinguish from coacervates when examined solely by light microscopy.

**Figure 57:**
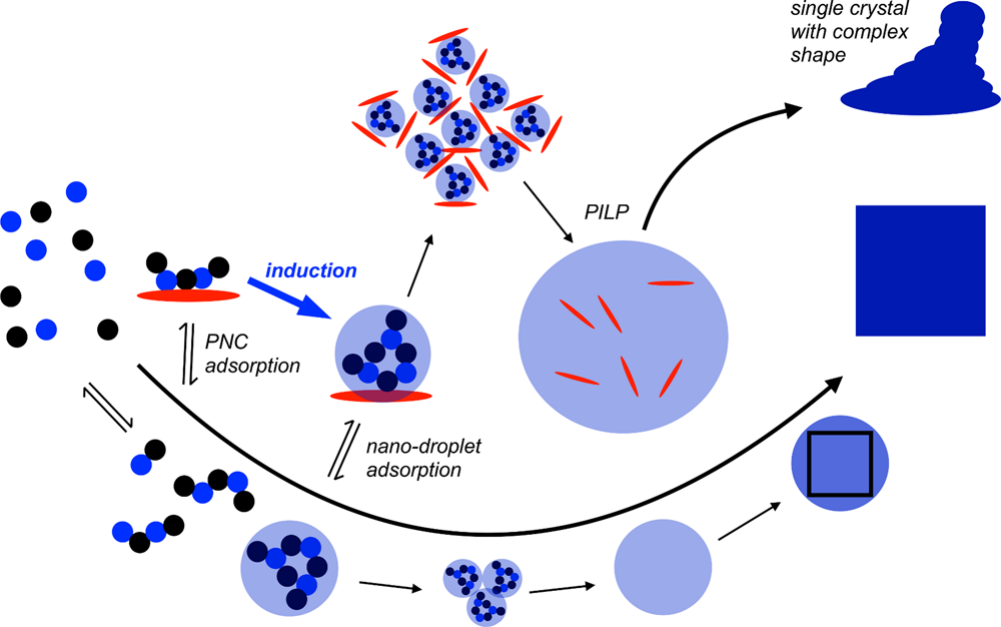
Schematic illustration of the mechanism of nucleation according to the PNC pathway (bottom) with the effects of PNC and nanodroplet adsorption by an additive (red ellipsoid). For strong ion-additive interactions and low additive concentrations (or medium-to-low-strength interaction but high additive concentrations), the additive becomes incorporated into the liquid intermediates and may kinetically stabilize these intermediate states that can grow into macroscopic “polymer-induced” liquid precursors (PILPs; note that these species are rather polymer-stabilized than polymer-induced states),^140,163^ reaching sizes of hundreds of micrometers that can be observed by using light microscopy.^133^ This pathway is expected for additives that also inhibit dehydration and/or coalescence of nanodroplets. Eventually, single- and polycrystals with complex shapes can be obtained in this PILP-mediated process.^133^ The binding of PNCs by additives in favorable configurations can also induce liquid–liquid separation, as the bold blue arrow indicates. Reproduced from ref (128) under a 4.0 Creative Commons Attribution License (CC BY 4.0). Copyright 2018 MPDI. http://creativecommons.org/licenses/by/4.0/.

Nevertheless, when CaCO_3_ nanodroplets form, which are the nucleation product upon surpassing the binodal limit, they can interact with the additive, resulting in their stabilization. This phenomenon is particularly noticeable at low additive concentrations and when interactions between the additive and the PNCs are of moderate or weak strength. Subsequently, the aggregation of individual nanodroplets may occur as a subsequent step, as depicted in Figure 57. This process can further evolve into a PILP, as experimentally demonstrated by NMR through the stabilization of a liquid CaCO_3_ phase with poly(aspartate).^140^ The polymeric additives are presumably located in the liquid phase and play a crucial role in stabilizing it kinetically. The coalescence of PILP droplets is a recognized phenomenon that indicates their lack of colloidal stability. In addition to preventing coalescence, the additive also serves to stabilize the PILP against dehydration, a process that could otherwise result in the precipitation of a solid phase. It is crucial to acknowledge that this stabilization is not indefinitely sustainable, and eventually, the metastable PILPs typically undergo precipitation, culminating in the formation of a polycrystalline solid (see also Figure 67).

**Figure 58:**
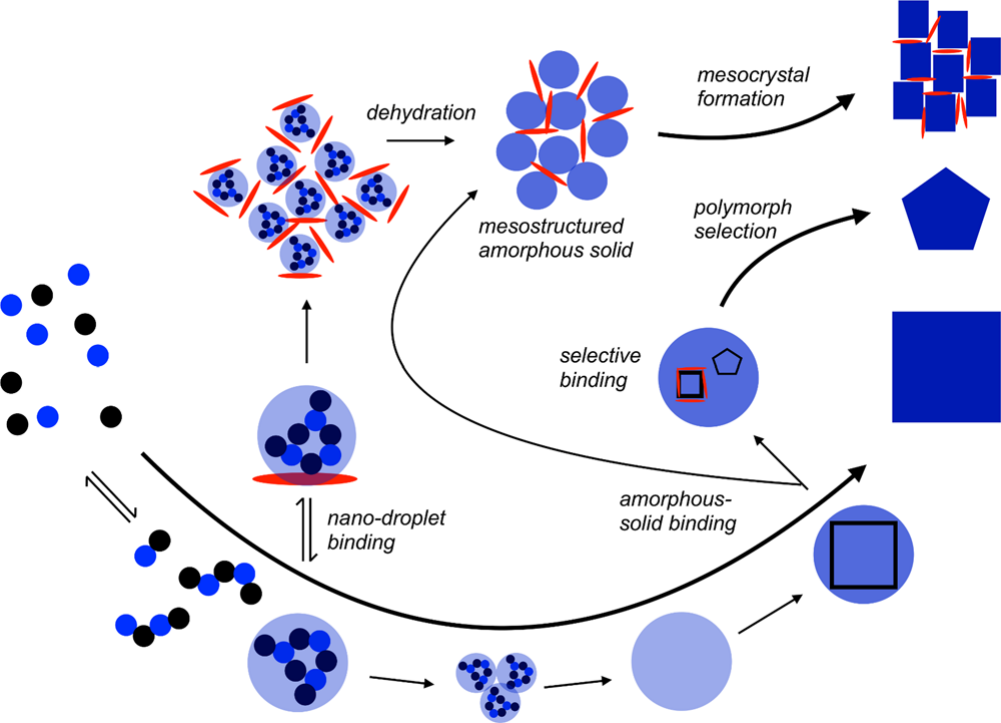
Schematic illustration of the mechanism of nucleation according to the PNC pathway (bottom) with potential effects of nanodroplet and amorphous-solid adsorption by additives (red ellipsoid) that do not kinetically stabilize liquid intermediates, at least to any significant extent. Reproduced from ref (128) under a 4.0 Creative Commons Attribution License (CC BY 4.0). Copyright 2018 MPDI. http://creativecommons.org/licenses/by/4.0/.

The dehydration process of liquid nanodroplet intermediates is illustrated in Figure 58. A mesostructured amorphous solid can form directly from the aggregated droplets or may emerge from the amorphous precursor particles within the PNC pathway (Figure 58). Experimentally distinguishing between these two possibilities is challenging. Still, the pathway based on nanodroplets would result in smaller amorphous solid structures (approximately 2–5 nm) compared to the aggregation of amorphous nanoparticles formed along the PNC pathway. Nevertheless, depending on the kinetics of dehydration, aggregation, and crystallization, it is plausible that a pathway involving both liquid and solid amorphous precursors may also be feasible.

The crystallization of the mesostructured amorphous solid via solid state transformation^417^ or a localized dissolution-reprecipitation of the amorphous nanoparticles^14^ might lead to the formation of a mesocrystal. However, if the nanoparticles do not align in a crystallographic register, a polycrystal may form instead. In particular, if the additive exhibits selectivity for a particular crystal polymorph, the crystallization process may occur under polymorphic control. As we move beyond the nucleation of a solid phase and witness crystal growth, it becomes imperative to distinguish between monocrystalline and polycrystalline structures to unravel the precise role of the additive.

### 5.1 Single Crystals

#### 5.1.1 Morphological Control

Understanding the impact of additives, particularly qualitatively, on the morphology of single crystals can be effectively approached through Wulff’s rule (eq 12).^418^
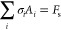
12where σ_*i*_ = interface energy, *A*_*i*_ = area of surface *i*, and *F*_s_ = surface free energy.

At thermodynamic equilibrium, *F*_s_ is minimized, implying that the sum of the products of interface energy and surface area for all exposed crystal surfaces is minimal. In simple terms, Wulff’s rule suggests that high-energy surfaces will grow rapidly, resulting in smaller surface areas. Conversely, low-energy surfaces grow more slowly and are thus exposed in the equilibrium crystal morphology. This concept is useful in elucidating the influence of additives on equilibrium crystal morphology.

**Figure 59:**
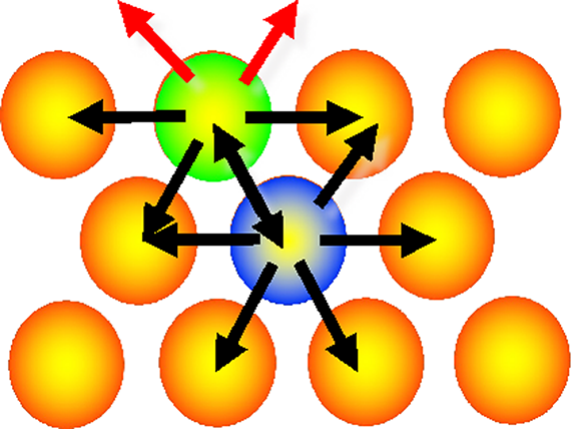
Concept of interface (surface) energy. The blue atom in the bulk has bonds to all neighboring atoms (black arrows). The green surface atom is missing parts of its neighbor atoms, and therefore, dangling bonds are introduced (red arrows), which generate surface energy.

The rationale behind surface energy at a phase boundary is unsatisfied dangling bonds, as shown in Figure 59. In this context, surface tension represents the strength of dangling bonds per unit area. Highly polarizable and high-melting substances, such as ionic crystals, exhibit elevated surface tension, whereas van der Waals solids, like organic crystals, have comparatively lower surface energy.

These dangling bonds can be satisfied, at least in part, by adsorption of additives onto the surface or by interaction with the solvent, effectively reducing the surface energy. This change in the surface energy Δσ can be estimated from the molar free energy of adsorption Δ*E*_ads_ using eq 13.
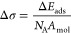
13where *N*_A_ = Avogadro’s number and *A*_mol_ = cross section per molecule.

**Figure 60:**
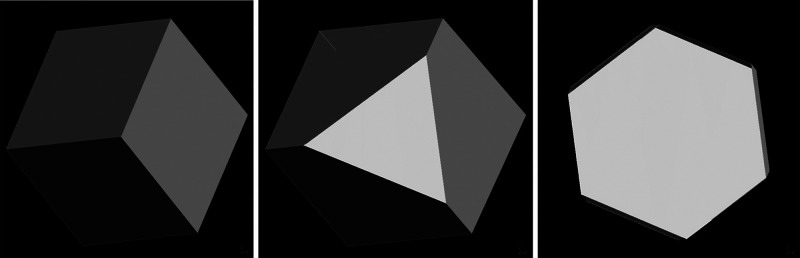
Change of the rhombohedral equilibrium CaCO_3_ morphology with six exposed {104} faces (gray) by lowering the surface energy of the {001} faces (white). The morphology change from rhombohedra to a hexagonal platelet is evident and is maintained just by face-selective interface energy decrease of the two {001} faces. Images modeled with Cerius^2^ (Accelrys). Reproduced with permission from ref (405). Copyright 2008 American Chemical Society.

According to Wulff’s rule, a reduction in surface energy results in a larger surface area for the specific crystal face. Consequently, face-selective adsorption of additives onto crystal faces provides an effective method for modifying the morphology of a single crystal. This phenomenon is evident in Figure 60, as exemplified by the calculated change in morphology of a calcite crystal, transitioning from rhombohedra to a hexagonal platelet through the selective reduction of interface energy on the two {001} faces.

**Figure 61:**
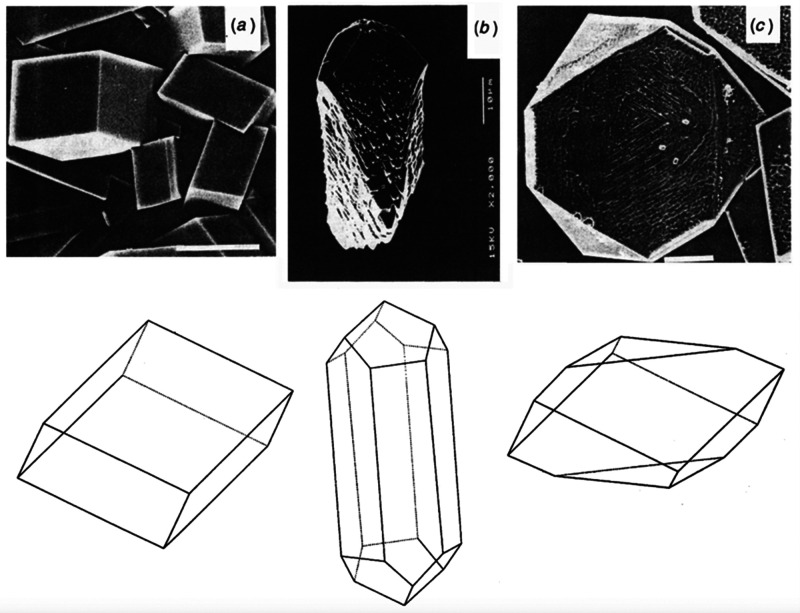
Observed (top) and calculated (bottom) calcite morphology showing (a) {104} rhombohedral faces, (b) {100} faces stabilized with Mg^2+^, and (c) {001} faces stabilized with Li^+^. Reproduced with permission from ref (420). Copyright 1991 Royal Society of Chemistry.

Experimental evidence has demonstrated that simple ionic additives can provoke a substantial alteration in the morphology of a single crystal (Figure 61). A notable example is observed in rhombohedral calcite, wherein the introduction of Mg^2+^ results in the stabilization of {100} faces, while Li^+^ leads to the stabilization of the {001} faces.^419,420^

**Figure 62:**
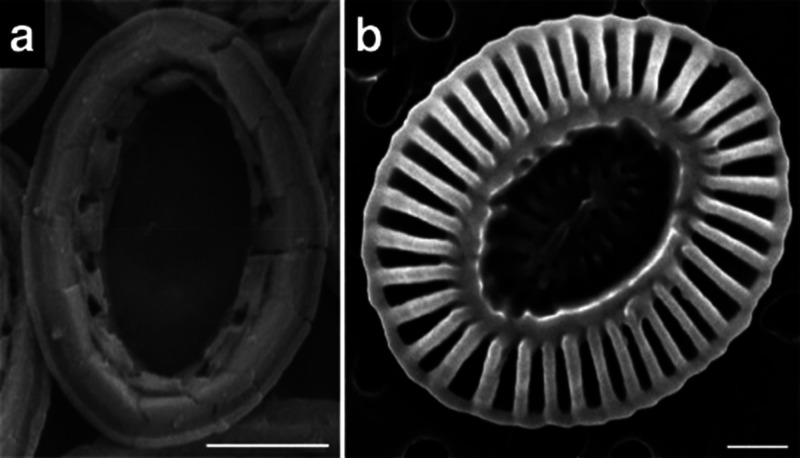
SEM images of a single coccolith from *Emiliania huxleyi*. Scale bar: 500 nm. The ring element is composed of hammer-shaped subunits. Reproduced with permission from ref (421). Copyright 2021 Elsevier.

Molecules of greater complexity, with multiple interaction sites and macromolecules with intricate functional group patterns, can have correspondingly complex interactions with a growing crystal, resulting in elaborated crystal shapes, including curved surfaces. This phenomenon is particularly evident in biominerals, such as the intricate hammer-shaped subunit of a coccolith ring shown in Figure 62.

Nevertheless, in some cases, the interactions of macromolecules exhibit surprising simplicity. For instance, it is established that acidic macromolecules, such as poly(styrenesulfonate)^409,422^ or acidic proteins,^423^ readily adsorb onto the charged calcite {001} faces, akin to the behavior observed with Li^+^ in the example mentioned earlier. Notably, complete coverage of a surface with additives is not a prerequisite to impede further growth.

**Figure 63:**
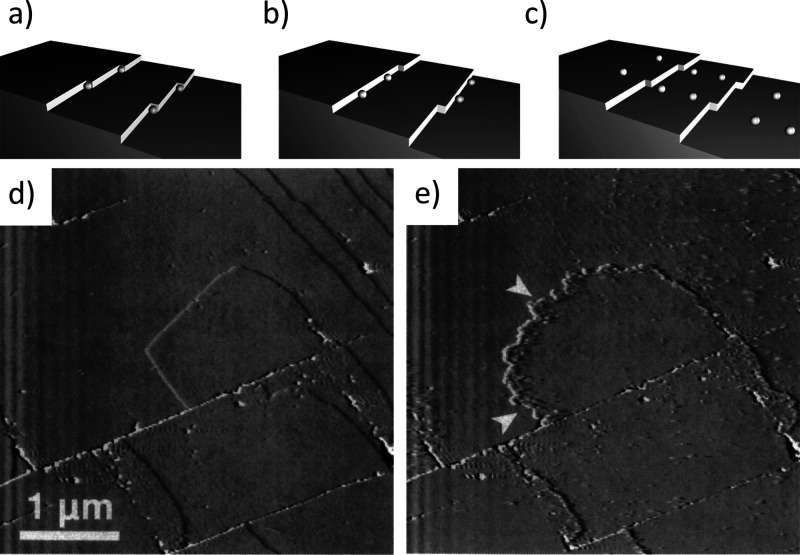
Sites for impurity adsorption on a growing crystal based on the Kossel model: (a) kink, (b) step, and (c) surface after additive adsorption.^425^ Reproduced with permission from ref (126). Copyright 2008 John Wiley & Sons. (d) Calcite (104) surface without proteins. Light and dark gray lines are obtuse and acute step edges, respectively. Step edges are generally straight and smooth, with sharp corners. Some kinks are visible in the acute step edges in the upper right corner. (e) Calcite surface with proteins. Step edges have become rounded (suggesting an isotropic step edge speed) and more convoluted. The step edge appears highlighted by a raised lip of proteins. Strong white-and-black features (that are identical in (d) and (e)) are defects in the crystal that can act as barriers to step-edge motion. Reproduced with permission from ref (426). Copyright 1997 Elsevier.

The layer-by-layer growth of a single crystal, as elucidated by Kossel’s model,^424^ holds significant implications for the influence of additives on the growth of a crystal face. Additives have the capacity to adsorb at diverse sites on the developing crystal, including surfaces, kinks, and steps (Figure 63). This dual effect involves a reduction in the surface energy of the crystal face and the hindrance of step edges from progressing in their growth. Consequently, the impact of an additive on crystallization is both thermodynamic and kinetic in nature. Adsorption of additives to steps and kinks is notably efficient, requiring less additive compared to covering an entire surface to impede its growth. This adsorption process emerges as a highly effective means of altering the morphology of a growing crystal.

**Figure 64:**
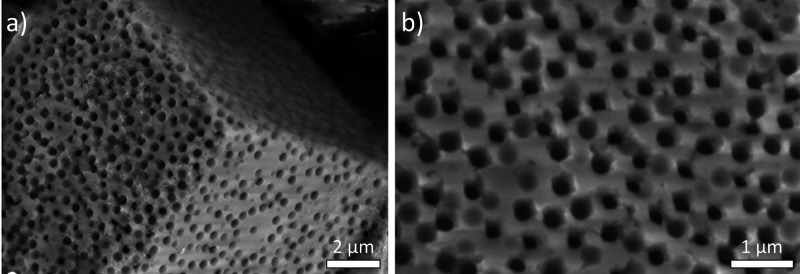
Scanning electron microscopy (SEM) images of CaCO_3_ particles with porous surface obtained by templating and tetrahydrofuran (THF) extraction of P(St-MMA-AA) latex particles with a size of 380 nm (a, b). Reproduced with permission from ref (428). Copyright 2005 American Chemical Society.

If the growth of a crystal face is not completely inhibited, additives have the potential to be assimilated into the crystal during overgrowth by subsequent layers. This phenomenon is well illustrated in the case of polymer latexes, which serve as a model for polymer additives. These additives are specifically functionalized to adsorb on specific crystal faces, facilitating their subsequent incorporation into the growing crystals.^427,428^ When removed from the crystal by dissolution or calcination, a porous crystal with a distinctive “Swiss cheese” morphology is formed, providing clear evidence of the incorporation of the latex additive. This is demonstrated in the case of calcite, as illustrated in Figure 64.

**Figure 65:**
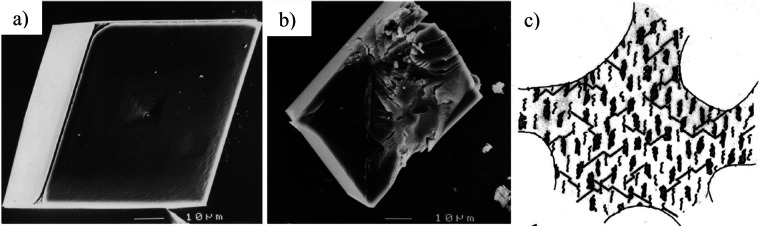
Indentations on single calcite crystals (a) with occluded sea urchin test proteins and (b) without occluded proteins. The same load 10 N/μm^2^ was applied. (c) Schematic illustration of the design strategy of an echinoderm stereom. The distribution of the cleavage planes solid straight lines and the protein-associated dislocations in the calcite of the echinoderm stereom. Scale: tens of nanometers between dislocations. Reproduced with permission from ref (430). Copyright 2000 Elsevier.

In biominerals, it has been observed that the incorporation of macromolecules into single crystals induces lattice distortions.^412,429^ This phenomenon proves beneficial in enhancing the mechanical properties of the single crystals, as evidenced by the increased hardness resulting from the inclusion of block copolymer micelles.^414^ The same is true for the incorporation of single amino acid molecules into the calcite single crystal lattice, where the amino acid content tunes the hardness.^415^ The beneficial effect of incorporating organic (macro)molecules extends beyond hardness to include an improvement in fracture toughness, as exemplified by calcite crystals incorporating proteins from echinoderm skeletal elements (Figure 65).^430^

While calcite single crystals have well-developed {104} cleavage planes, rendering them brittle (Figure 65b), the incorporation of biological macromolecules extracted from a sea urchin transforms calcite into a fracture-resistant and tough material (Figure 65a). In this scenario, an indentation does not result in crystal fracture but induces a plastic behavior, where the indent is distinctly visible on the single crystal without causing its rupture.^430^ This mirrors the behavior observed in a sea urchin spine, where the incorporation of merely 0.1 wt % of macromolecules induces a conchoidal fracture pattern instead of the cleavage plane typical of a single crystal. It is also essential to acknowledge the mesocrystalline structure of the sea urchin spine, which supports the conchoidal fracture behavior (see also Section 6.1). The experimental evidence revealed that these macromolecules preferentially adsorb onto crystal planes parallel to the crystal *c*-axis instead of the exposed low-energy {104} planes, causing a dislocation. These planes/dislocations, being oblique to the {104} cleavage planes, have been proposed to serve as a mechanism for deviating cracks (Figure 65c).^431^ The strategy of enhancing the hardness and toughness of inorganic crystals by incorporating organic (macro)molecules into their single crystal lattice proves highly advantageous. This approach is easy to implement and allows for the tuning of crystal hardness by adjusting the content of organic molecules.

**Figure 66:**
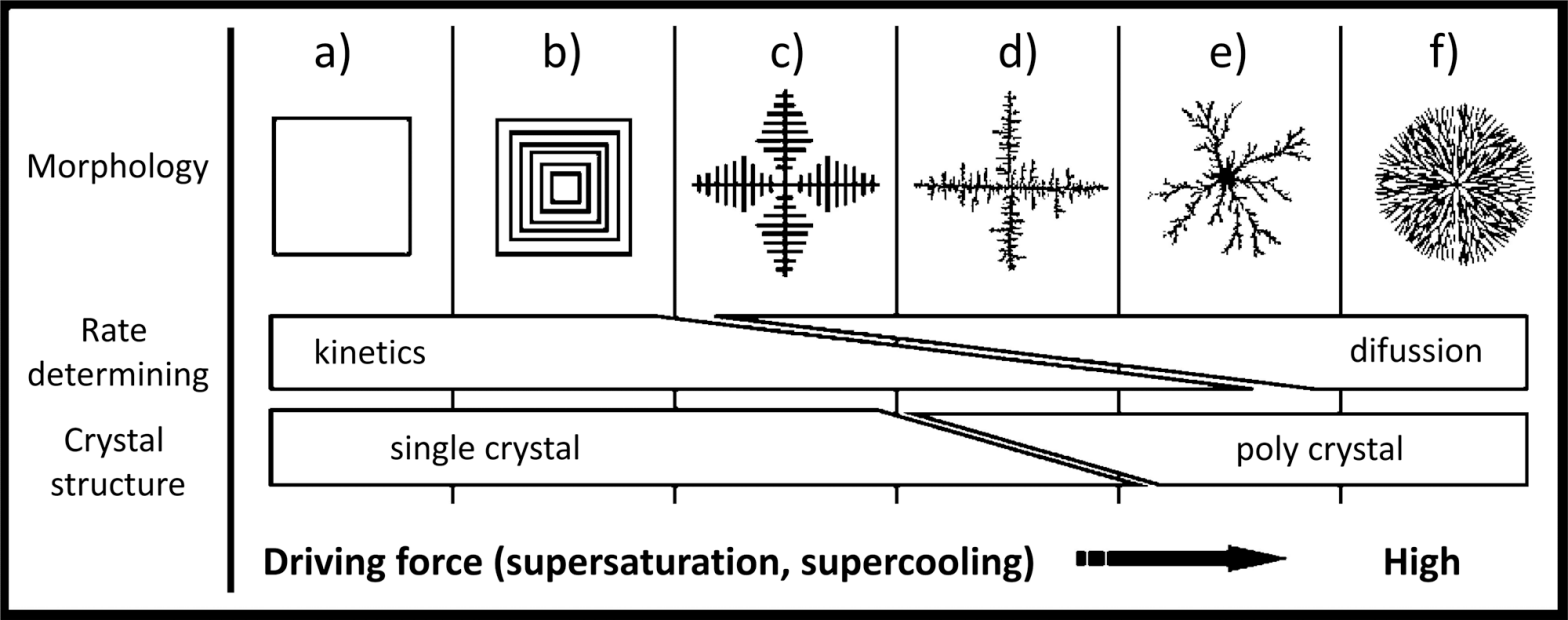
Schematic model of morphological evolution with an increase in the driving force; (a) polyhedral form produced in the kinetic-controlled system near equilibrium, (b) skeletal morphology by the Berg effect, (c) single-crystalline ordered dendrite with crystallographic symmetry, (d) partially disordered dendrite having a single-crystalline ordered trunk and disordered polycrystalline side branches, (e) disordered polycrystalline dendrite as shown in diffusion-limited aggregation (DLA), and (f) dense branching morphology (DBM). Reproduced with permission from ref (432). Copyright 2003 American Chemical Society.

Although the strategies for crystal morphogenesis discussed above are based on thermodynamic control at near-equilibrium or equilibrium conditions, it is important to recognize that many crystallization phenomena occur far from equilibrium and are governed by kinetic or diffusion processes, which are probably the dominant processes controlling the formation of cement hydrates. Therefore, it is crucial to take into account the influence of kinetics and diffusion, especially when dealing with high concentrations of reactants or substantial driving forces toward crystallization.^432^ As illustrated in Figure 66, an increase in the driving force toward crystallization leads to the development of ordered dendritic structures, accompanied by an escalation of disorder in these dendritic formations. As diffusion control becomes prominent, the disorder intensifies, eventually forming a densely branched morphology. While the results presented are derived from crystallization in gel media, their applicability extends to crystallization processes in general, with or without additives.

Achieving high supersaturations with nucleation inhibitors often leads to the formation of amorphous rather than crystalline phases, a phenomenon commonly observed in various mineralizing systems.^433^ While amorphous precursor phases are inherently less stable than their crystalline counterparts, they offer significant advantages in the creation of intricate crystalline morphologies. A compelling example of this is found in biominerals, which are characterized by complex and delicate shapes that defy conventional crystal symmetry. The flexibility of amorphous materials allows them to be molded and formed into various shapes, contributing to the unique and intricate structures observed in biominerals.

Several intricate biomineral morphologies, such as those in diatoms formed from amorphous silica,^434^ and coccoliths composed of CaCO_3_ (see Figure 62), exemplify the utilization of amorphous precursors. The use of amorphous precursors as building blocks ensures efficient transport of material to the crystallization site while preventing the buildup of high ionic strengths and associated osmotic pressures. Additionally, removing substantial solution volumes from the mineralization site is not required, as is necessary when constructing with atoms/ions/molecules directly from the solution.

Amorphous precursor particles are strategically favored as the initial species in a crystallization pathway, aligning with Ostwald’s rule of stages (see Section 3.1). This preference arises from their status as the least stable and least dense modification, resulting in the lowest activation barrier for their formation. Consequently, as the supersaturation level increases, there is a corresponding increase in the concentration of amorphous clusters, and their formation is further facilitated. Far beyond the saturation threshold, the formation of amorphous clusters and droplets in ionic solutions becomes almost inevitable.^435,436^ In such scenarios, the supersaturation diminishes much faster than the appearance of discernible crystals, allowing for the utilization of amorphous and liquid precursors for shaping before crystallization occurs. Amorphous precursor phases play a distinctive role in the formation of low-solubility minerals. The inherently lower solubility not only elevates the apparent supersaturation, even at moderate concentrations, but also imparts kinetic stability to the amorphous nanostructures, preventing their redissolution. The transformation of amorphous precursor particles into a single crystal typically occurs through two principal mechanisms: 1) they serve as material depots, undergoing subsequent dissolution and recrystallization, or 2) they fuse together, either before or after crystallization, giving rise to a single crystal in a nonclassical crystallization reaction, as elaborated below.

In the initial part of this section, we demonstrated that additives such as PAA act as nucleation inhibitors, consequently retarding nucleation and permitting it to occur only at elevated supersaturation levels. Consequently, the formation of equilibrium structures is not anticipated under such conditions. Instead, polycrystalline structures and intricate morphologies, in addition to the previously mentioned amorphous phases, are likely to emerge. This is due to the potential formation and growth of numerous crystal nuclei into nanoparticles facilitated by the high supersaturation. As a result, aggregation-based pathways assume significance in the kinetically controlled regime, emphasizing the role of multiple nucleation events rather than the formation of single crystals under thermodynamic control.

Additives play a crucial role by adsorbing onto specific faces of nanocrystals, effectively encoding them for subsequent aggregation processes, diverging from the alteration of single crystal morphology in accordance with Wulff’s rule. This scenario is of significant interest to the morphological control of crystalline structures. Should nanoparticles self-assemble and align with mutual orientation in a crystallographic register, there is the potential for them to crystallographically fuse by Oriented Attachment (OA), resulting in the elimination of two adjacent crystal surfaces. This process can potentially yield single crystals, albeit typically on the nanoscale. Micron-sized crystals, on the other hand, often do not diffuse enough to avoid collisions with other crystals, and they do not rotate enough for two identical crystal faces to align and match. Because this route to single crystals is based on nanoparticles rather than atomic or molecular building blocks as in the classical crystallization mechanism, such particle-based routes to crystallization are referred to as “nonclassical crystallization″^125,437−442^ and involve *Mesocrystals*(413,443−455) as well as OA.^456−469^ As both have developed into distinct research fields, they can only be briefly discussed here, focusing on their relevance to cement self-assembled structures. For a more in-depth exploration, the reader is encouraged to refer to the review articles cited above or consult a comprehensive book that covers nonclassical crystallization in its entirety.^126^

#### 5.1.2 Polymorphic Control

Finally, soluble additives have the potential to affect not only the morphology but also the polymorphism of a single crystal. Given that polymorphs manifest distinct properties, including mechanical strength, optical characteristics, and solubility, there is a keen interest in manipulating them with additives. One such application entails stabilizing metastable phases, as we introduced with alternative binders. This approach can yield improvements in mechanical properties or resistance against degradation. Polymorph selection often involves solvents, temperature changes, or changes in other growth conditions to control crystallization by changing from thermodynamic to kinetic control or vice versa.^470−472^ Polymeric additives are highly effective in governing the polymorphism of crystals as they exert influence over nucleation and crystallization events at multiple levels.^433,473−476^ In rare occasions, the same polymer can regulate the formation of different polymorphs. An example is seen in CaCO_3_, where sodium poly(sodium 4-styrene-*co*-*N*-isopropylacrylamide sulfonate) demonstrated control over all three anhydrous CaCO_3_ polymorphs by simple adjustments in polymer and Ca^2+^ concentrations. This was sufficient to shift the balance of the crystallization reaction between thermodynamic and kinetic control.^477^

Remarkable examples of polymorph control observed in biomineralization, particularly in organisms like mollusks, show the ability to selectively deposit a specific CaCO_3_ polymorph (aragonite or calcite). Such control is achieved under the influence of biopolymers, even when external conditions remain relatively constant.^478^ Notably, these mechanisms do not solely rely on a “magic” protein with epitaxial relations or other capabilities that induce the nucleation of a specific crystal polymorph.^479,480^ Instead, it is the physical chemistry of polymer interactions with all compounds involved in nucleation and growth at various stages of crystallization that plays a pivotal role. This interaction is then manifested in the potential formation of amorphous precursor species and the delicate balance between thermodynamic and kinetic reaction control. A similar outcome can be achieved through the variation of simple reaction parameters such as temperature, reactant concentrations, and their mixing and residence time in the reactor, as demonstrated by Nebel and Epple.^481^ They designed a continuous CaCO_3_ synthesis procedure capable of producing all anhydrous crystalline polymorphs in pure form.

Often, Ostwald’s step rule (see also Figure 19) is employed to guide the crystallization reaction along the kinetic pathway, and the reaction is then halted upon the formation of the desired polymorph. However, when this polymorph is exposed to the solvent, a transformation into a more stable polymorph may occur through dissolution-recrystallization, as observed in stearic acid,^482^ magnesium phosphate hydrates,^483^l-histidine,^484^ and CaCO_3_.^485^ If the activation energy for transformation into a more stable polymorph is sufficiently low, a solid-state transformation may also occur. This is commonly observed in systems with low cohesion energies within the crystal or at annealing temperatures slightly below the melting point. Hence, such transformations are notably prevalent in organic crystals at annealing temperatures proximate to their melting points. In contrast, for inorganic ionic systems, the activation energies for such transformations are generally very high, precluding the occurrence of solid-state transformations between polymorphs. When additives exhibit specific binding to a particular polymorph in solution, the interface energy of that polymorph is reduced. Consequently, direct nucleation of that polymorph is favored over other polymorphs that would nucleate in the absence of the additive. However, achieving this specificity is challenging, given that polymorphs are chemically identical.

Remarkably, even amorphous phases such as biogenic amorphous CaCO_3_ (ACC) can exhibit a local order resembling that of the polymorph crystallized from the ACC precursor.^486,487^ Also, synthetic ACC has been discovered to possess a proto structure resembling all three anhydrous polymorphs, contingent upon precipitation conditions such as pH or temperature.^488,489^ Nevertheless, unlike biogenic ACC, synthetic ACC does not consistently transition into the crystalline polymorph of its proto structure.

The above examples illustrate the complex interplay between thermodynamic and kinetic polymorph control, a complexity that can often be achieved through the strategic use of additives. The influence of additives on the reaction pathway can be substantial, making additive polymorph selection a challenging but promising avenue. Despite the current lack of predictability, a comprehensive characterization of the effect of the additive at all stages of the crystallization pathway, starting from the prenucleation stage, certainly provides valuable insights. This understanding will help assess whether crystallization is likely to proceed along a thermodynamic or kinetic pathway, or a combination of both, and identify the precursor species that warrant consideration.

### 5.2 Polycrystals

The field of polycrystalline structures formed in the presence of additives is vast and includes a wide range of particle-based nonclassical crystallization mechanisms.^125,126,437−439,441,442,490^ These nonclassical crystallization pathways, such as mesocrystals and oriented attachment, are becoming increasingly important in various systems. The exceptional mechanical properties demonstrated by mesocrystalline biominerals, such as sea urchin spines, nacre, and bone (see Section 6.1), suggest that additive-controlled particle-mediated crystallization will play a critical role in the development of future materials that require a combination of strength and toughness.

The prevalence of polycrystalline structures in the presence of additives can be attributed to another factor - many additives act as nucleation inhibitors, resulting in increased supersaturation (S) until nucleation occurs. The high thermodynamic driving force toward nucleation (Δ*G* = −*RT* ln *S*) favors multiple nucleation events over a single nucleation event, a prerequisite for the growth of a single crystalline structure. Thus, polymeric additives typically promote the formation of polycrystalline structures. The morphologies of these structures cover a wide range, raising the question of how much control over the morphology and internal structure of a polycrystalline phase can be exerted by additives and/or reaction conditions. Unfortunately, little is known about the control of polycrystalline structures. However, lessons can be learned from biomineralization, where a structurally insoluble polymer matrix serves as a template for the morphology of the growing (poly)crystal, while a functionally soluble polymer matrix controls the crystallization process itself.

**Figure 67:**
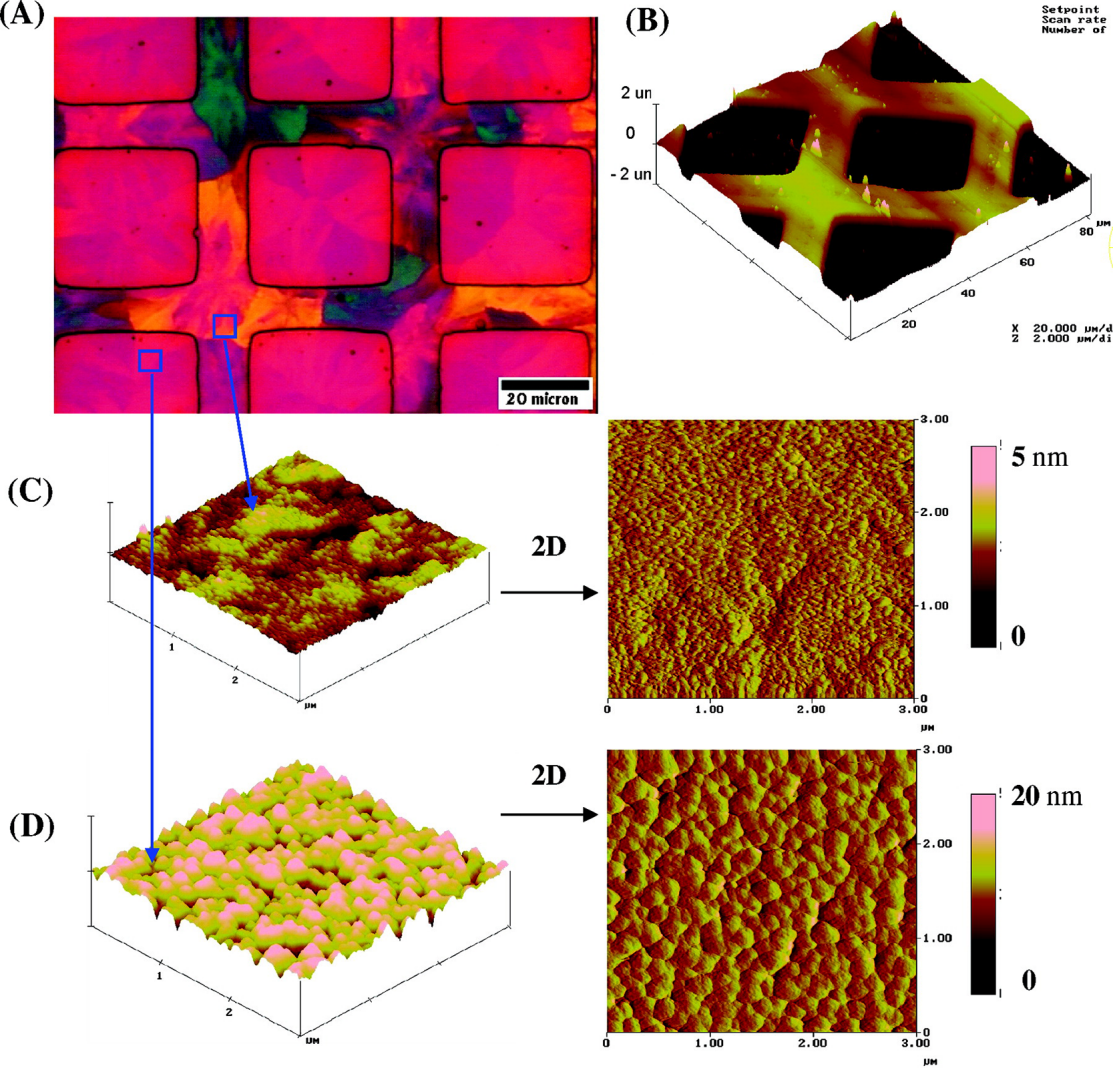
Comparison of calcite film topology when PILP phase is deposited on different surfaces. (A) Polarized light micrographs show that, in this experiment, the PILP phase deposited on both the COO^–^-terminated SAMs (grid regions) and on the bare gold surface (interior square regions) but was much thicker on the former, as indicated by the different brightness of the birefringence. (B) Three-dimensional AFM height image scanned across an 80 μm × 80 μm surface area showing different film thicknesses for PILP phase deposited on COO^–^-terminated SAMs versus on bare gold surface. The scale bar is 20 μm/division in the *x*–*y* plane and 2 μm/division in height. (C) and (D) AFM images scanned across the surface of a 3 μm × 3 μm area (as indicated with the blue boxes in (A) within each patterned region ((left) 3-D “height-mode” image to show quantitative height deviation; (right) “deflection-mode” image to show lateral dimensions of colloidal surface), showing colloidal particle morphology with different size ranges and surface roughness (rms (roughness) = 4.7 nm for film on the COO^–^-terminated region vs 17 nm on the bare gold surface). As can be seen in (C), PILP film deposited on COO^–^-terminated SAMs was formed from significantly smaller particles than that formed on the bare gold surface (D), even though the final film thickness was much greater. Reproduced with permission from ref (491). Copyright 2007 American Chemical Society.

Liquid or amorphous precursors are particularly well suited for filling a template or dictating a morphology. For example, PILPs of CaCO_3_ have been selectively deposited on carboxy-terminated self-assembled monolayers (SAMs), as depicted in Figure 67.^491^ It is evident that the polycrystalline CaCO_3_ layer adheres to the grid structure of the SAM template, with significantly smaller crystals compared to the particles deposited on the nontemplated interspaced gold regions, despite the fact that the templated layer is significantly thicker overall. This example vividly illustrates the precise control of polycrystalline structures in terms of morphology using an external template. It is worth noting that this template does not necessarily have to be a 2D structure but can take various forms as long as it allows the structuring of the liquid or amorphous phase.

**Figure 68:**
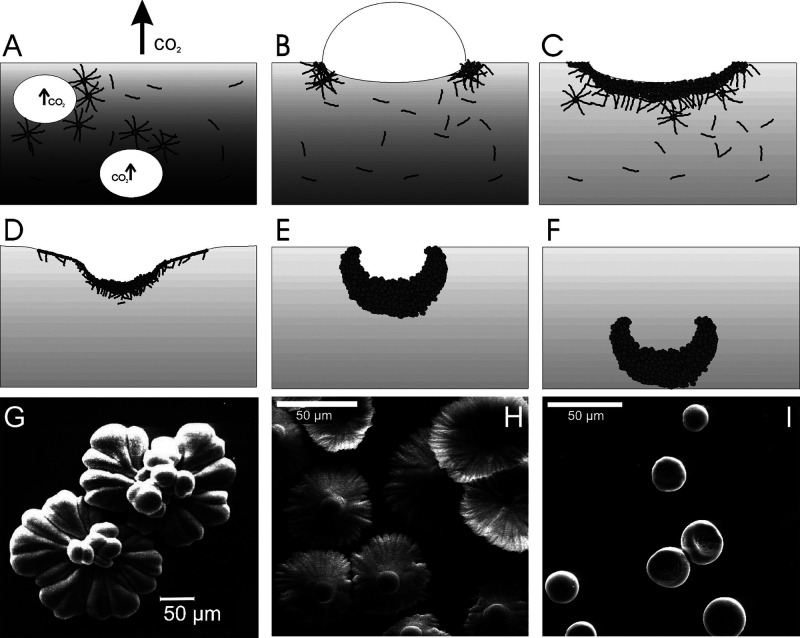
Schematic presentation of the formation of polycrystalline complex CaCO_3_ morphologies using phosphorylated block copolymers for temporary nanoparticle stabilization and CO_2_ gas bubbles as templates generated in the Kitano method for CaCO_3_ crystallization.^492^ (A) CaCO_3_ nanoparticles are generated near the air–water interface and stabilized by the block copolymer while CO_2_ gas bubbles are also developing. (B) The gas bubbles can stay at the surface, and a rim of aggregating nanoparticles forms around them. (C) With time, the rim closes, and the structure gets heavier by the attachment of further nanoparticles. (D, E, F) It is now a matter of surface tension, how big the aggregate structures can grow until they loosen from the air–water interface and sink to the bottom of the vessel. Reproduced with permission from ref (408). Copyright 2004 American Chemical Society. (G–I) SEM images of the polycrystalline CaCO_3_ structures formed with decreasing surface tension caused by a decreasing phosphorylation degree of the block copolymer. Reproduced with permission from ref (493). Copyright 2002 John Wiley & Sons.

This phenomenon is depicted in Figure 68. Small CaCO_3_ nanoparticles are generated through CO_2_ outgassing from a bicarbonate solution, creating calcium carbonate supersaturation using the Kitano method,^492^ starting at the air–water interface. Phosphorylated double hydrophilic block copolymers^476^ temporarily stabilize these nanoparticles,^408,493^ which are the building units for the polycrystalline superstructures with complex shapes. The CO_2_ bubbles formed during outgassing temporarily reside at the air–water interface and can act as templates for CaCO_3_ nanoparticle attachment, as shown in the scheme in Figure 68. Initially, they form rings around the gas bubble that close as nanoparticle attachment continues, resulting in open hemispheres of polycrystalline CaCO_3_. The surface tension of the solution influences the formation of large complex structures (Figure 68G). This factor determines how large the structure can grow until it can no longer be supported by the surface, causing it to sink to the bottom where further growth is quenched.

The hemispherical structures gradually sink under the influence of their weight but remain attached to the surface, resulting in a complicated shuttlecock morphology. Finally, this morphology detaches from the surface and sinks to the bottom, where no further particle attachment occurs. The morphology of the polycrystalline CaCO_3_ particles, which are formed from CaCO_3_ nanoparticles, is temporarily stabilized with block copolymers. The polymer plays a dual role by temporarily stabilizing the nanoparticles and simultaneously controlling the surface tension. This dual function determines the maximum size of the polycrystalline structures before they detach from the surface and descend to the bottom, reaching a point where no further growth occurs. This example illustrates how additives can give rise to polycrystalline structures with remarkable complexity, supplementing the formation of PILPs, which can adopt polycrystalline structures of virtually any shape. The underlying templating principle is the key to achieving these complex structures.

### 5.3 Additive Influence on C–S–H Homogeneous Nucleation

In the context of the application of additives in cement, the main objective has been to improve the workability of the paste during hydration by reducing the water requirement. Less water reduces the final porosity, which contributes to improved mechanical properties, as described in Section 2.4 (eq 1). Only a few studies have focused on elucidating the effect of additives on C–S–H nucleation and early growth or have attempted to control these processes through careful selection of appropriate additives. In the following, we have included the most relevant from our point of view.

**Figure 69:**
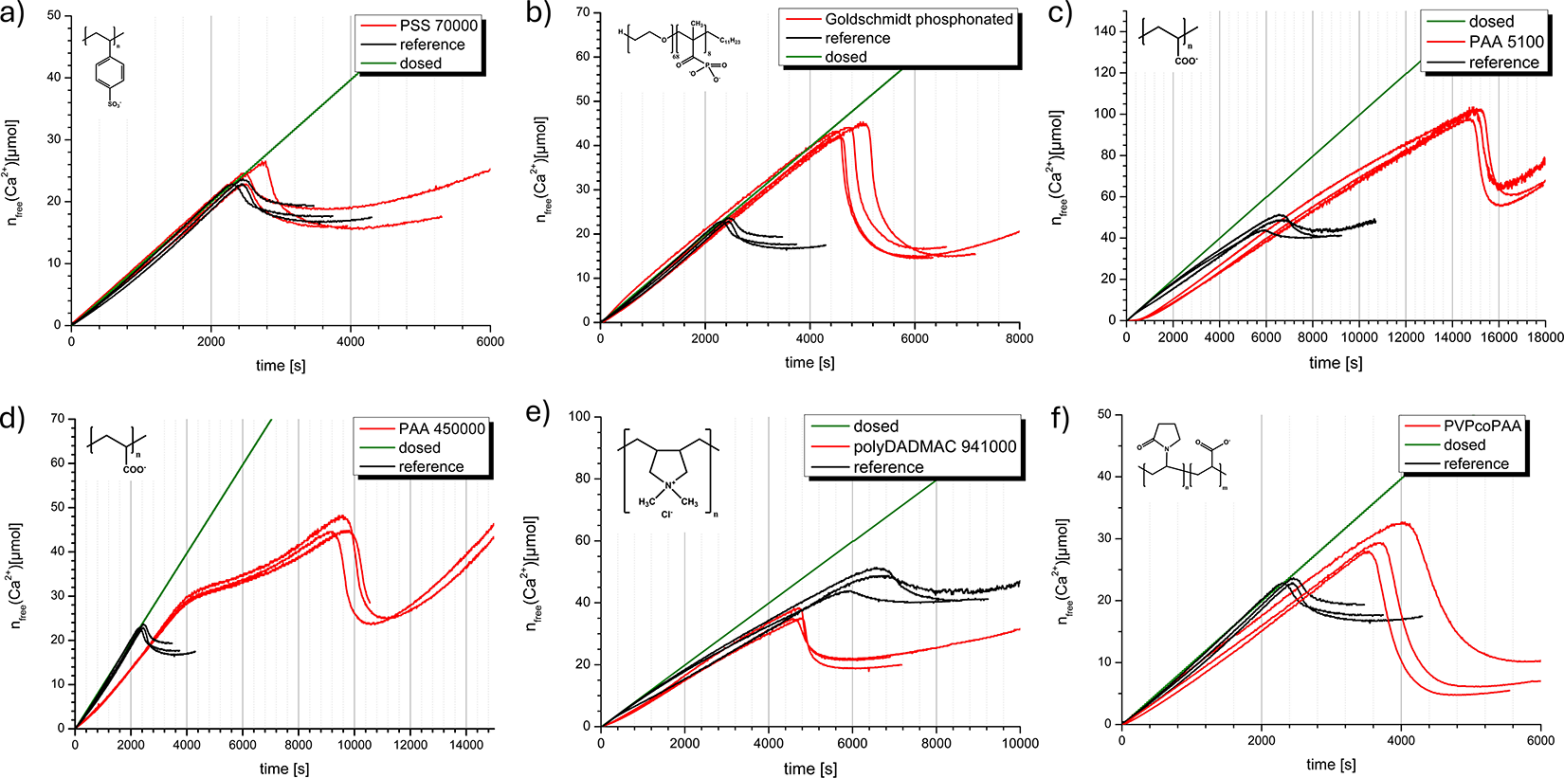
Examples of titration curves for C–S–H nucleation in the presence of different additives. Experiments in the presence of additives (gray curves) are compared to reference experiments in the absence of additives (black curves). c) and e) for pH 12, others for pH 13. a) PSS 70000, no influence; b) Goldschmidt phosphonated, nucleation retardation; c) PAA 5100, nucleation retardation and Ca2+ binding; d) PAA 450000, nucleation retardation, Ca^2+^ binding and stabilization of primary nucleated particles; e) polyDADMAC 941000, nucleation acceleration and precipitation of a calcium-rich phase; f) PVP-*co*-PAA, precipitation of a calcium-rich C–S–H. Reproduced with permission from ref (11). Copyright 2023 Elsevier.

Picker et al. conducted a comprehensive study that systematically investigated the effect of various polymeric additives on homogeneous C–S–H nucleation. Their results showed a common trend: all anionic polymers tested exhibited a retarding effect on C–S–H nucleation (Figure 69). The delay in nucleation is not related to the amount of calcium bound in Ca-polymer complexes, as some polymers cause retardation without significantly binding calcium ions. However, Ca-ISE (ion selective electrode) measurements showed that significantly more calcium is bound to the silicates, most likely via the anionic polymers. This suggests stabilization of the calcium-silicate oligomeric species against aggregation in the presence of organics and the inhibition of the formation of amorphous C–S–H spheroids.^11,12,494^ In contrast, some cationic polymers (PolyDADMAC) accelerate C–S–H nucleation at low concentrations. They suggested that this may be due to a higher concentration of silicate species in the vicinity of the cationic functions, thereby promoting oligomerization.^11^ Nonionic polymers do not exert a large effect, as expected.

Smaller anionic (gluconate) and neutral molecules (hexitols) have also been shown to retard homogeneous C–S–H formation, as demonstrated by Bouzouaid et al.^13^ The authors evaluated their data within the CNT approach. They calculated the product of the pre-exponential factor (A) times the kinetic barrier term (exp(−*E*_a_/*k*_B_*T*)), i.e. the first two terms in eq 5), for the reference system and each additive. They observed that this factor is lowest for the pure system and highest for the gluconate, with hexitols showing intermediate values. Interestingly, they are directly correlated with the complexation constants determined for each organic substance with the C–S–H surface. Based on this, the inhibition mechanism may be related to the stronger absorption of the organics to C–S–H clusters, which prevents their aggregation.^495^ Alternatively, organics may have a stabilizing effect on the silicate and calcium PNCs, which can be inferred from the flatter slope in the free-Ca^2+^ curves.^11^ Taken together, these two studies suggest that the organic additives may be interacting with the dissolved species at the prenucleation stage by inhibiting the formation of the amorphous C–S–H phase. In addition, in some cases, the additives also slowed the conversion to the crystalline phase. Sowoidnich et al. also demonstrated that PCEs interact with ions and larger calcium silicate species during C_3_S hydration using AUC. In addition to the formation of calcium-polymer complexes, AUC revealed the presence of nanoscale clusters approximately 3 nm in size. Although the composition and structure of these nanoscale clusters remained elusive at the time, the authors proposed that they may represent polymer-stabilized C–S–H prenucleation clusters.^200^

The studies discussed above, conducted in the presence of organics, demonstrate that additives can influence C–S–H formation from very early stages. Their interaction with PNCs and intermediate precursor phases should be a key consideration in our understanding of the cement hydration process. As discussed in various sections of the review, this can be extended to other cement-relevant phases (e.g., portlandite, C-A-S-H, alternative binding phases), as most exhibit nonclassical crystallization mechanisms.

## 6 Bioinspired Organic–Inorganic Hybrid Cement

### 6.1 Hybrid Organic–Inorganic Structures in Nature: Biominerals

Biomineralization serves as a rich source of inspiration for the development of crystalline organic–inorganic hybrid materials.^405,496−500^ This is attributed to the superior performance of biominerals compared to their inorganic counterparts, which are often characterized by brittleness, as observed in materials such as glass, calcium carbonate, or calcium phosphates. The key lies in the precise control of nucleation, mineralization/crystallization events, and the frequent hierarchical structuring over multiple length scales, as exemplified in natural structures such as bone,^501^ nacre,^502^ or glass sponges.^503^ Using these design principles, living organisms construct materials with exceptional mechanical or optical properties.

#### 6.1.1 Toughening Mechanisms of Biominerals

**Figure 70:**
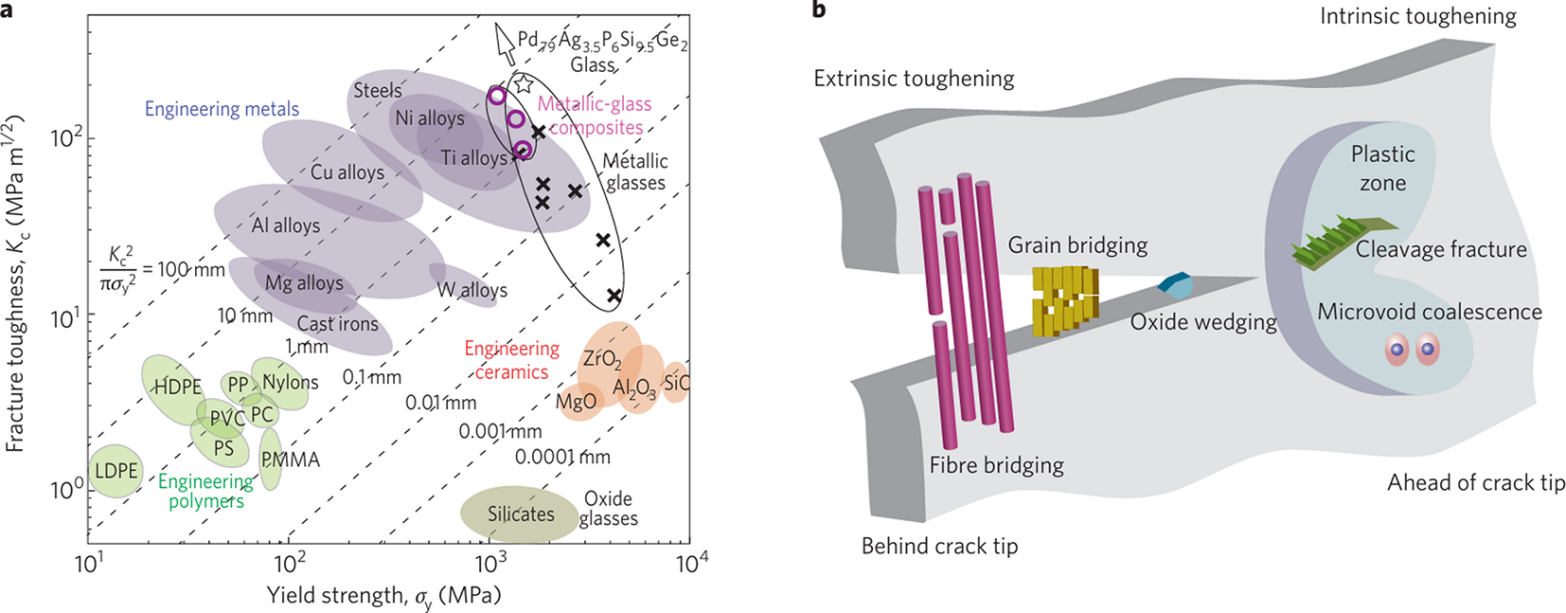
Conflicts of strength versus toughness adapted from ref (504). a) Ashby plot showing strength–toughness relationships for engineering materials. Diagonal lines show the plastic-zone size, *K*_c_^2^/πσ_*y*_^2^, where *K*_c_ is the fracture toughness and σ_*y*_ the yield strength. The white star and purple circles refer, respectively, to the Pd-glass and metallic-glass composites, as compared with monolithic glasses (black crosses). Reproduced with permission from ref (506). Copyright 2011 Springer Nature. b) Schematic illustration showing how strength and fracture behavior can be considered in terms of intrinsic (plasticity) versus extrinsic (shielding) toughening mechanisms associated with crack extension. The illustration shows mutual competition between intrinsic damage mechanisms, which act ahead of the crack tip to promote crack advance, and extrinsic crack-tip-shielding mechanisms, which act primarily behind the tip to impede crack advance. Intrinsic toughening results essentially from plasticity and enhances a material’s inherent damage resistance, increasing both the crack-initiation and crack-growth toughness. Extrinsic toughening acts to lower the local stress and strain fields at the crack tip; as it depends on the presence of a crack, it affects only the crack-growth toughness, specifically through the generation of a rising R-curve.^507^ Reproduced with permission from ref (504). Copyright 2014 Springer Nature.

The unique combination of mechanical strength and toughness, characteristics that typically exclude each other in conventional materials (see Figure 70a),^504^ is achieved through specialized design motifs and multiple toughening mechanisms (see Figure 70b for general intrinsic and extrinsic toughening mechanisms) operating at several hierarchy levels.^505^ Comprehensive reviews address these mechanisms and explore how material structure enables the combination of strength and toughness.^504,505^ Here, we provide a brief overview of these mechanisms and highlight their relevance to the advancement of cementitious materials. By designing materials with these mechanisms in mind, there is potential to reduce the reliance on Portland cement.

**Figure 71:**
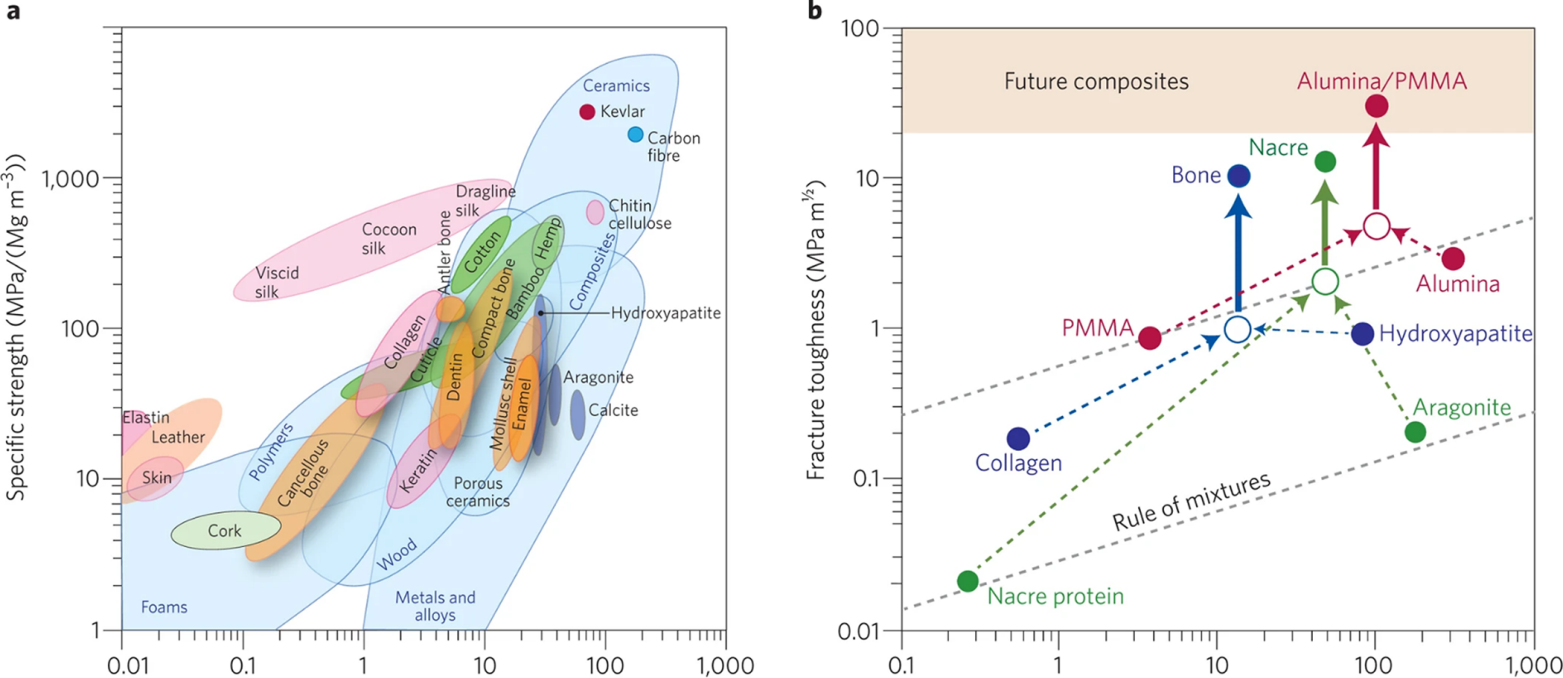
Material-property chart and projections for natural and synthetic materials. a) Ashby plot of the specific values (normalized by density) of strength and stiffness (or Young’s modulus) for both natural and synthetic materials. b) Many natural composite materials, as exemplified by bone and nacre, have toughness values that far exceed those of their constituents and homogeneous mixtures (as indicated by the dashed lines). They can sustain incipient cracking by utilizing extensive extrinsic toughening mechanisms. This results in much higher toughness for crack growth (closed symbols above the solid arrows) than for crack initiation (open symbols) and, thus, higher fracture toughness (solid arrows). By mimicking the architecture of nacre in a synthetic ceramic material (alumina/PMMA),^508^ similar behavior and exceptional toughness can be attained. Reproduced with permission from ref (505). Copyright 2015 Nature Publishing Group.

Biominerals, with their distinctive intrinsic and extrinsic toughening mechanisms, successfully reconcile the typically opposing characteristics of strength and toughness. Their hierarchical structure, with different toughening mechanisms at different levels, serves as a source of inspiration for the design of synthetic organic–inorganic hybrid materials. The remarkable combination of strength and toughness is evident from the Ashby plot shown in Figure 71a. Although synthetic materials have superior performance compared to biominerals, such as high-performance ceramics, the use of inexpensive ingredients and production under ambient conditions make biomineralization strategies highly desirable for the creation of synthetic materials.^505^ Moreover, the structural complexity of biominerals, characterized by interwoven and interlocking arrangements, significantly enhances their mechanical performance, surpassing the cumulative performance of individual constituents (Figure 71b).

**Figure 72:**
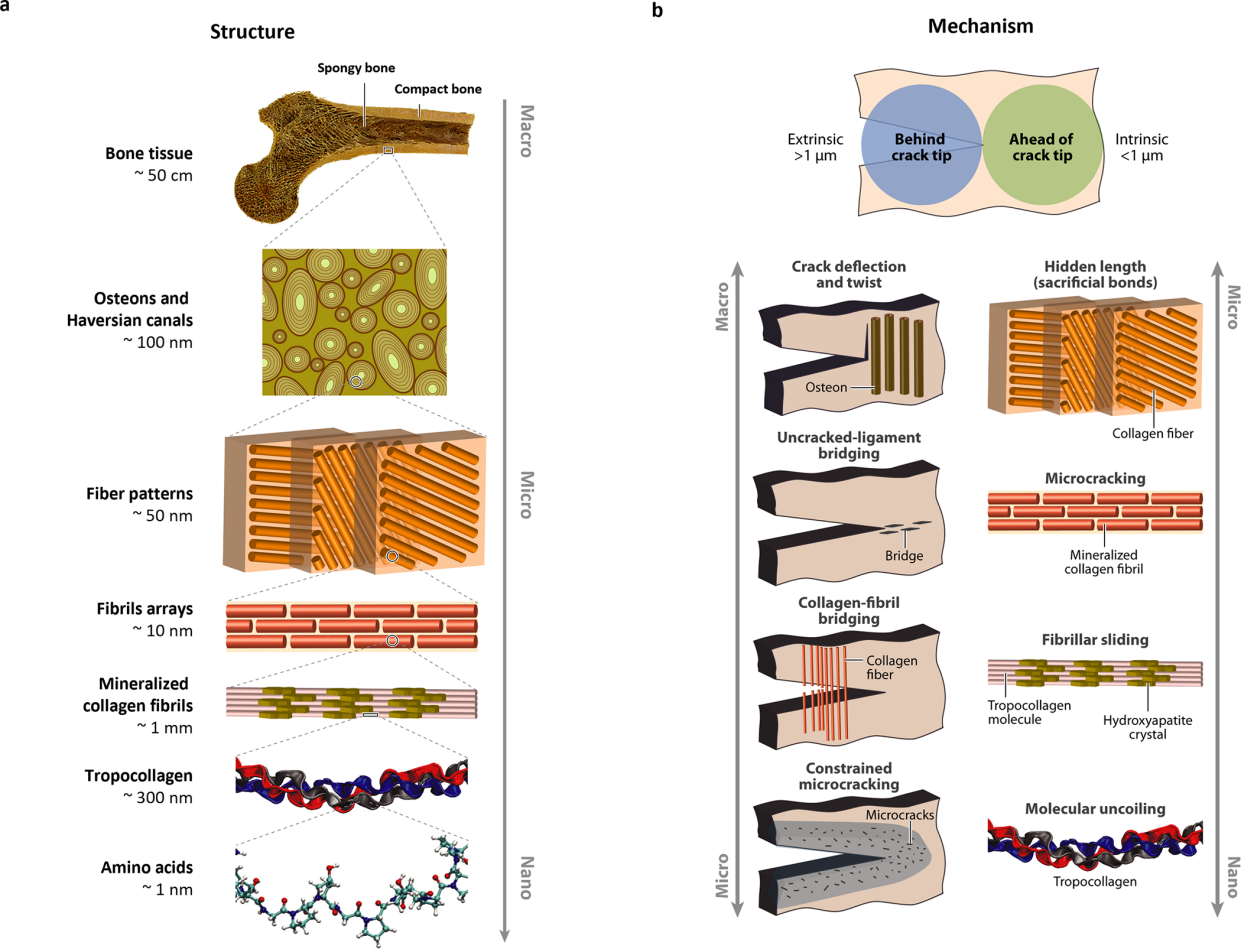
Structure of bone shows the seven levels of hierarchy with the prevailing toughening mechanisms. a) The seven levels of hierarchy.^501^ b) The prevailing toughening mechanisms.^512^ At the smallest level, on the scale of the tropocollagen molecules and mineralized collagen fibrils, (intrinsic) toughening, that is, plasticity, is achieved through molecular uncoiling and intermolecular sliding of molecules. At coarser levels, on the scale of the fibril arrays, microcracking, and fibrillar sliding act as plasticity mechanisms and contribute to intrinsic toughness. At micrometer dimensions, the breaking of sacrificial bonds at the interfaces of fibril arrays contributes to increased energy dissipation and crack bridging by collagen fibrils. On the largest length scales, in the range of tens to hundreds of micrometers, the primary sources of toughening are extrinsic and result from extensive crack deflection and crack bridging by uncracked ligaments, both mechanisms that are motivated by the occurrence of microcracking. Adapted from ref (504). Reproduced with permission from ref (512). Copyright 2010 Annual Reviews.

**Figure 73:**
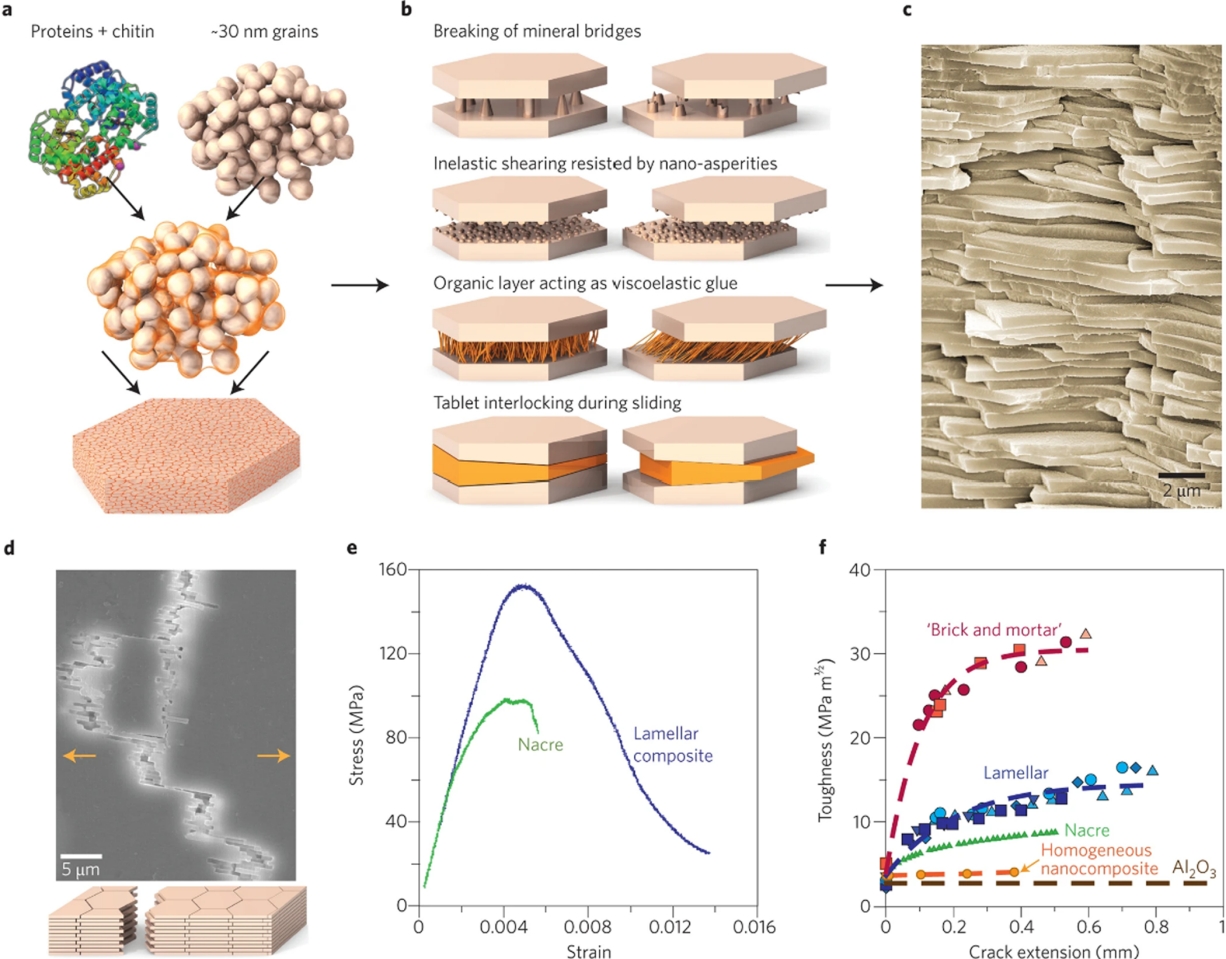
Hierarchical structure and properties of nacre adapted from ref (505). Nacre is a brick-and-mortar structure a) of CaCO_3_ mineral platelets (aragonite), which provide strength and proteins and allow for ductility and toughness. The mineral bricks, which until recently were thought to be brittle single crystals, are ∼500 nm thick and ∼5–10 μm wide and are comprised of millions of nanograins (∼30 nm) glued together by a biopolymer, forming a mesocrystalline structure.^527^ The resulting structure would be hard yet hopelessly brittle if the platelets were rigidly interlocked. Instead, the biopolymeric mortar between the bricks generates limited deformation between the mineral layers (as shown by the mechanisms depicted in b), thereby relieving locally high stresses while also providing ductility without too much loss in strength. c) SEM image of a nacre surface, depicting the highly ordered aragonite platelets. Optimum properties come about when the strength of the mortar is fractionally less than the strength of the bricks, such that toughening through d) crack bridging can occur when the bricks pull out without breaking (orange arrows indicate the direction of tension). Comparison of the e) strength and f) toughness of natural nacre and nacre-like alumina/PMMA ceramics made using freeze casting.^508,537^ Toughening is associated with brick pull-out and frictional sliding in the compliant polymeric layer. The nacre-like alumina/PMMA ceramic has exceptional fracture toughness, exceeding *K*_c_ = 30 MPa m1/2, an order of magnitude higher than the toughness of its constituent phases and homogeneous alumina/PMMA nanocomposites. Panels a–d are reproduced with permission from ref (505). Copyright 2015 Springer Nature. Panels e and f are reproduced with permission from ref (508). Copyright 2008 American Association for the Advancement of Science.

Examining bone (Figure 72a) and nacre (Figure 73) as exemplary hierarchical biominerals reveals the sophisticated hierarchical structure underlying their multiscale toughening mechanisms. It is crucial to distinguish between intrinsic toughening mechanisms, such as molecular collagen uncoiling or fibril sliding, operating at the smallest length scales to provide ductility, and extrinsic mechanisms operating at the scale of 1–100 μm that can lead to crack shielding (see Figure 70b and Figure 72b). Fibrillar sliding depends on several factors like sacrificial bonds,^509^ the hydroxyapatite/collagen interface, or intermolecular cross-linking of the collagen molecules.^505^ This ductility, due to plasticity, dissipates energy by forming plastic zones around incipient cracks, blunting crack tips, and reducing the driving force for cracking.

Significantly more energy dissipation occurs through extrinsic toughening mechanisms, such as crack bridging, where microcracks form ahead of the crack tip, and crack deflection at structural features like osteocyte-lacunae (Figure 72b).^510,511^ Diverting the crack from the plane of the maximal tensile stress leads to a notable decrease in the crack tip stress intensity.^505^

Similar intrinsic and extrinsic toughening mechanisms are at play in nacre. Its structure is comparatively simpler, with a brick-and-mortar arrangement of hard but brittle aragonite platelets and soft, ductile polymer layers in between. This simplicity has often allowed successful synthetic mimicry of the structure of mother-of-pearl, even surpassing the properties of the natural analogue^508,513−523^ (for reviews on these materials, see refs.^524,525^). The platelet structure, however, is more intricate. Nanocrystalline aragonite grains are embedded in a protein matrix in nacre, forming a mesocrystalline hexagonal nacre platelet (see Section 7.1 for mesocrystalline structures).^526^ As the platelet is deformed, intrinsic toughening mechanisms come into play, involving grain rotation and deformation that dissipate energy (Figure 73a).^527,528^

Similar to bone, the extrinsic toughening mechanisms at the microscale that resist platelet sliding play a more prominent role than the intrinsic ones, as illustrated in Figure 73b. Key mechanisms identified include the role of the organic layer between the mineral tablets as viscoelastic glue,^529^ nanoasperities preventing plate sliding,^530^ breaking of mineral bridges between the platelets,^531−535^ and platelet interlocking.^536^ In addition, toughening through crack bridging can also occur (Figure 73d). The successful mimicry of the brick-and-mortar structure is exemplified by the alumina/poly(methyl methacrylate) system in Figures 73e and f, demonstrating the potential to outperform the exceptional mechanical properties of nacre.^508^

#### 6.1.2 Construction Principles of Biominerals

**Figure 74:**
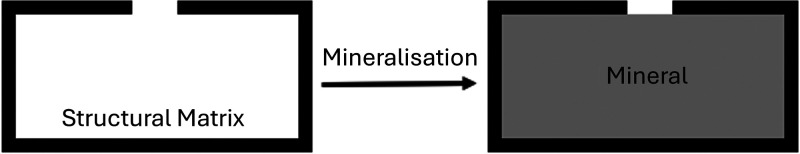
Schematic representation of a biomineralization event. First, a confined environment is built by the structural matrix. This gets filled with minerals in a second step, resulting in the organic–inorganic biomineral.

A fundamental understanding of how nature constructs biominerals is critical to the synthetic design of superior materials through bioinspired routes. The formation of these sophisticated materials involves multiple strategies, with cells playing a vital role, which may not be directly replicable in the laboratory. However, a fundamental principle used by living organisms in biomineralization that can be mimicked synthetically is the use of a structural and functional matrix (Figure 74).

Nature’s choice is wise because both structural and functional matrices consist of soluble and insoluble (macro)molecules whose structure and composition can be genetically controlled. Through the two-step biomineralization process illustrated in Figure 74, nature transforms genetically controllable soft matter into hard materials with exceptional mechanical properties. The structural and water-insoluble matrix is deposited first, providing a scaffold for subsequent mineral deposition, such as collagen in the case of bone or chitin in the case of nacre. This process is akin to the scaffolds used by humans in construction works. Additionally, it can play a role in controlling where the mineral will be deposited through molecular recognition, possibly after additional molecules such as proteins, glycoproteins, complex carbohydrates, proteoglycans, glycosaminoglycans, and sometimes lipids have been adsorbed on the scaffold surface.^538^

In addition to the structural matrix, a water-soluble functional matrix consisting of (macro)molecules is typically applied. These molecules can control the nucleation event, crystal/mineral growth, and shape through various mechanisms, as discussed in detail in references.^539,540^ For calcium-based biominerals, these molecules are typically acidic proteins,^541,542^ such as the Asp-rich proteins.^543^ This is relevant in the context of the present work because of the major role of calcium in cementitious materials, for which acidic macromolecules such as poly(acrylic acid) -based macromolecules are effective additives (see also Section 6.2.2). For silica-based biominerals, cationic (macro)molecules such as long-chain polyamines, silicateins, and silaffins are employed by living organisms.^544−554^ This is equally significant for calcium-silicate-hydrate, and potential additives, aside from those addressing the evident electrostatic interaction of polycarboxylates with calcium, can also target silica through cationic moieties.

**Figure 75:**
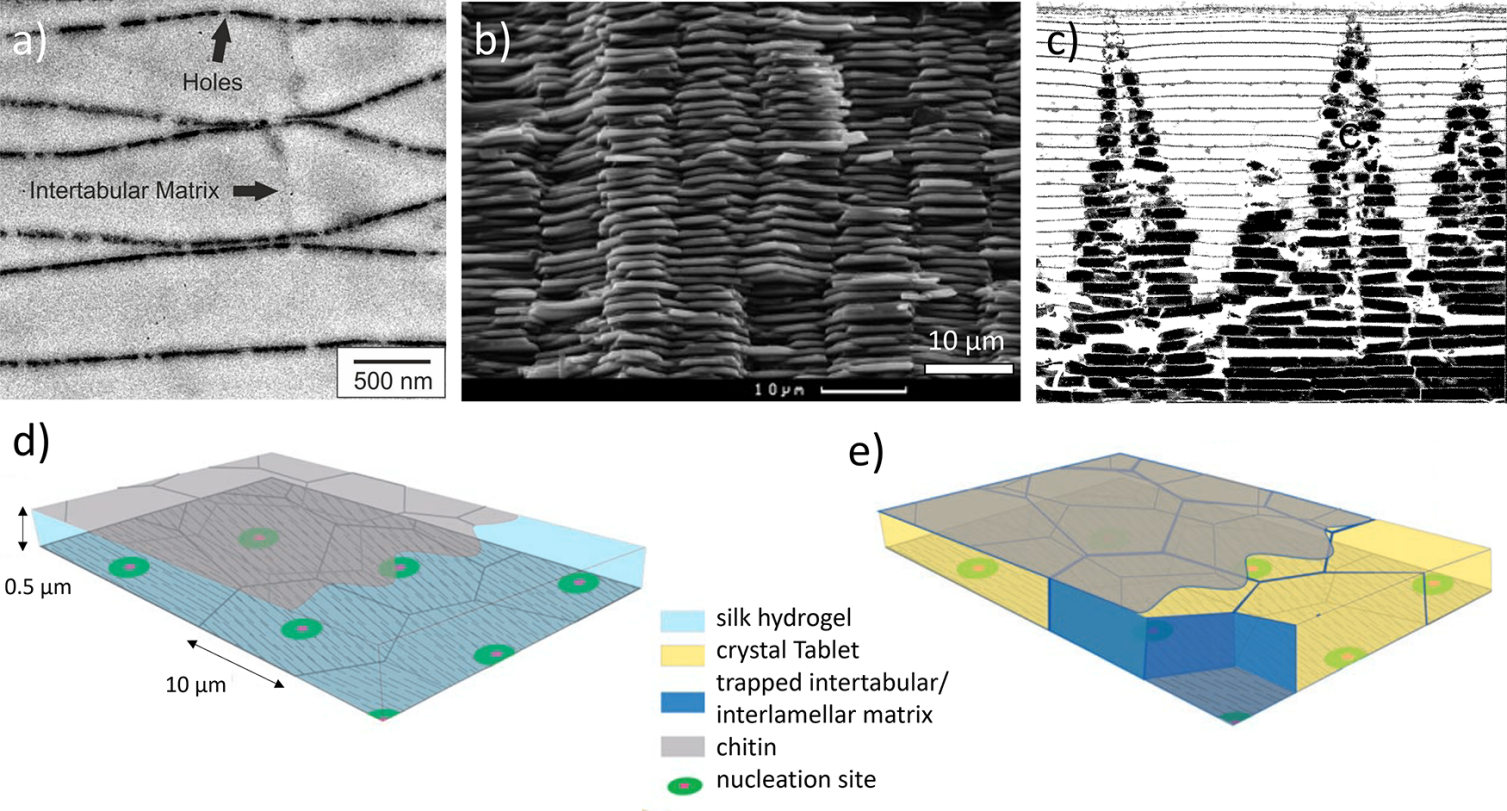
Growth of Gastropod nacre. a) TEM of the demineralized organic structural matrix without mineral reproduced with permission from ref (556) under the terms of the Creative Commons Attribution License. Copyright 2015 Beilstein Institute. http://creativecommons.org/licenses/by/2.0. b) SEM of fracture surface showing the aragonite tablets. Reproduced with permission from ref (557). Copyright 2008 Elsevier. c) TEM of growing aragonite tablets in the typical stack of coin manner, with the smallest mineral deposits on top being the most lately deposited ones in between the organic scaffold layers. Reproduced with permission from ref (558). Copyright 1983 Springer Nature. Schematic representation of the suggested nacre formation model d) before and e) after mineralization.^478^ d) The assembled organic matrix prior to mineral deposition. The microenvironment is formed by two layers of β-chitin, with a gel comprising silk-like protein filling the space in between. Part of the upper chitin layer (upper right) has been removed to show the silk-like protein gel filling. The gel phase may inhibit crystallization and act as a space filler. The silk gel may already be loaded with colloidal mineral particles. Nucleating proteins are adsorbed on the β-chitin sheet. For clarity, the proportions of the spacing between chitin layers and between nucleation sites on the chitin have been altered. Note that the polygonal outlines of imprints are created only during mineralization and have been added to this scheme for clarity only. e) Mineralized nacreous layer. The acidic proteins induce the nucleation of aragonite (from colloidal particles). As the mineral grows, water and silk are displaced. The latter is eventually trapped between adjacent tablets and between the tablet and the chitin layer. Part of the upper chitin layer has been removed with the underlying interlamellar matrix layer (upper right), to show the mineral tablet surface. A tablet fragment (front corner) was removed to allow visualization of the intertabular and interlamellar matrix. Images d and e are reproduced with permission from ref (478). Copyright 2006 John Wiley & Sons.

Examining the initial phases of nacre formation, the deposition of β-chitin layers is the first step (Figure 75a), interspaced with a silk hydrogel that prevents these layers from collapsing. This gel is thought to hinder crystallization and serves as a filler, possibly loaded with colloidal mineral particles.^478^ Nucleation of aragonite (from colloidal particles) is induced by and on the carboxylate proteins in the β-chitin layer. These proteins are surrounded by a sulfonated polysaccharide that acts as an ion sponge for calcium.^555^

During mineral growth, both water and silk are displaced. The silk is then trapped between adjacent tablets and at the interface between the tablet and the chitin layer (Figure 75c). This phenomenon would explain the organic interface between the aragonite tablets, also observed as connections between the horizontal chitin layers in Figure 75a. The structural matrix of the β-chitin layers, clearly visible as horizontal black lines in Figure 75c, directs the growth of the aragonite tablets within the confined space of the chitin layers parallel to these layers. This process leads to the layered structure of the aragonite tablets, as observed in the fracture surface (Figure 75b). The proposed formation mechanism for nacre is schematically represented in Figure 75 d and e.^478^

Drawing parallels with other biominerals such as nacre or mussel shells, the sea urchin spine emerges as another invaluable resource for guiding the formation of durable and resilient C–S–H structures. Like its counterparts, the sea urchin spine acts as a protective barrier for the organism, requiring remarkable fracture resistance—a property of great interest in the quest for high mechanical performance in cementitious materials. The formation of the sea urchin spine commences with vesicles that serve as vehicles (structural matrix) that contain amorphous calcium carbonate (ACC). These vesicles transport the ACC building material to the construction site.^559^ Initially, primary mesenchyme cells endocytose seawater from the larval internal body cavity into a network of vacuoles and vesicles.^560^ In this environment, calcium ions are concentrated until they precipitate as amorphous calcium carbonate (ACC). The mineral, in the form of aggregated 20–30 nm ACC nanospheres,^561^ is subsequently transferred to the syncytium where the spicule forms.

The hydrated phase of ACC nanoparticles transforms into the final calcite mesocrystal, progressing from the initial short-lived ACC phase to the final crystalline calcite nanoparticle building units. The ACC-calcite transformation is proposed to proceed through a tortuous path involving 40–100 nm ACC units via secondary nucleation.^141^ This process helps maintain the mutual crystallographic orientation of the nanocrystals, likely facilitated by mineral bridges or other direct contact of the nanoparticles.

**Figure 76:**
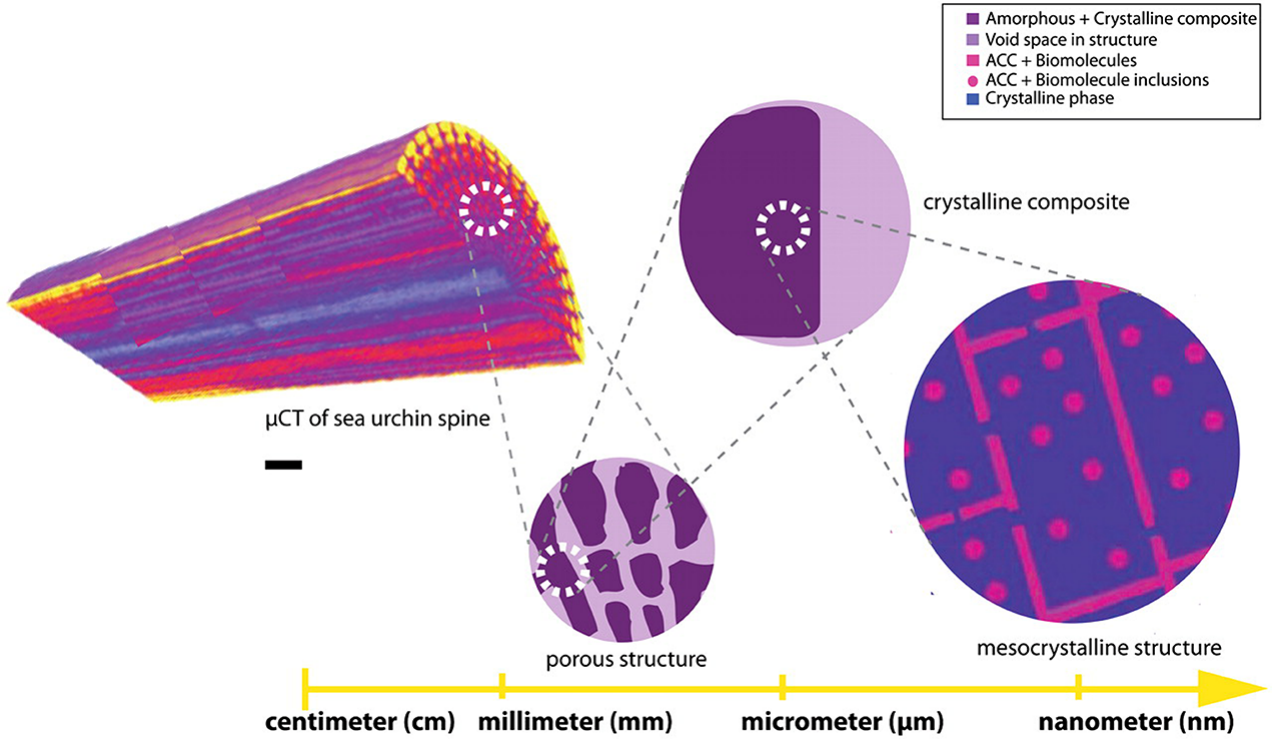
Schematic representation of structural hierarchy in the sea urchin spine. At the cm–mm scale, as visualized and rendered from microtomography measurements, the spine is shown to be a complex, porous material [scale bar = 5 μm]. When examined at the μm scale, the microstructure of the mineral becomes apparent, and rhombohedral as well as conchoidal fracture surfaces are typically observed. Light areas represent macroporosity, while dark purple areas represent the mineralized sea urchin spine. At the nanoscale, a mesocrystalline organization is apparent where the ordered nanocrystal building units are shown in blue, while ACC inclusions and surface layers are shown as pink dots and lines, respectively. Reproduced with permission from ref (562). Copyright 2012 National Academy of Sciences.

The hierarchical structure of the sea urchin, spanning from the nanometer scale to the centimeter scale, is illustrated in Figure 76. A thin nanometer scale ACC layer covers the crystalline calcite nanoparticles, which also contain amorphous regions internally.^562^ The amorphous layer between the crystallographically iso-oriented calcite nanoparticles (partly connected by mineral bridges) is similar to the organic layer between the aragonite platelets in the nacre, as it has no cleavage planes. This amorphous/crystalline nanocomposite in the sea urchin spine bears significant resemblance to other biominerals, such as bone, utilizing the hard–soft–hard layered structure for enhanced mechanical properties. This structural arrangement is critical to the role of the sea urchin spine as a protective element for the organism.

When attempting to create hybrid ordered structures at various hierarchical levels, the fundamental role of organic components becomes evident. These organic components serve as structural templates, regulate the nucleation and growth of minerals, and greatly enhance the overall strength of the resulting composites. The rigid mineral elements contribute to strength here, while the soft and ductile organic layers provide toughness. When attempting to create bioinspired PC cementitious materials, it is critical to design the interfacial interactions to achieve the desired reinforcement without compromising strength. This necessitates the use of specialized polymers capable of fostering interactions with the facets of C–S–H platelets. Additionally, these polymers should facilitate the bottom-up self-assembly of these platelets into a strong and tough material akin to the C–S–H mesocrystals explored in Section 7.2.

### 6.2 Cement Inorganic–Organic Composites

Building on the concepts introduced in the previous section, the development of bioinspired building materials could be very valuable in contributing to sustainable development in the construction industry by improving the durability and performance of cementitious materials. As explored in the earlier section (Section 6.1), the outstanding performance of biominerals is attributed to the synergistic integration of distinctly different materials, namely organic and inorganic components. This synergy depends on several mechanisms, such as the controlled control of the crystallization process by organic macromolecules, the regulation of fracture behavior through the specific distribution of organic and inorganic counterparts, and the establishment of a hierarchical organization over different length scales. In this context, the introduction of polymeric additives into concrete or mortars can impart increased flexibility to the cement matrix, effectively limiting and controlling crack propagation, analogous to natural biocomposites. Notwithstanding the higher cost of polymer-based construction materials, this could be compensated by the composite material’s long-term durability and higher strength.^563^

Polymer-cement composites with high tensile strength were produced in the early 1980s. Birchall et al. incorporated poly(vinyl alcohol) into PC blends, which resulted in a remarkable increase in flexural strength by eliminating large pores.^564,565^ However, the increase in fracture toughness still needed to be improved, leaving many unanswered questions about the interaction mechanisms between organics and cement hydrates in these composites.^113^ From that time to the present, almost every type of organic additive has been added to cement and concrete to improve their performance, so the literature on the addition of polymers to cementitious materials is simply unmanageable, with search engines returning over 700.000 publications using “polymer” and “concrete” as keywords. Of these, only a tiny fraction focuses on gaining fundamental knowledge of the mechanisms operating at the nanoscale, which is crucial for advancing our understanding of these materials. Here, we present a selection of publications that, in our view, have significantly contributed to a better understanding of these interactions. Our goal is to lay the foundation for future engineering efforts to build a better material. For a general overview of the various roles of polymers in cementitious systems, including the most outstanding findings in this field, we recommend the recently published review by Tran et al.^563^

**Figure 77:**
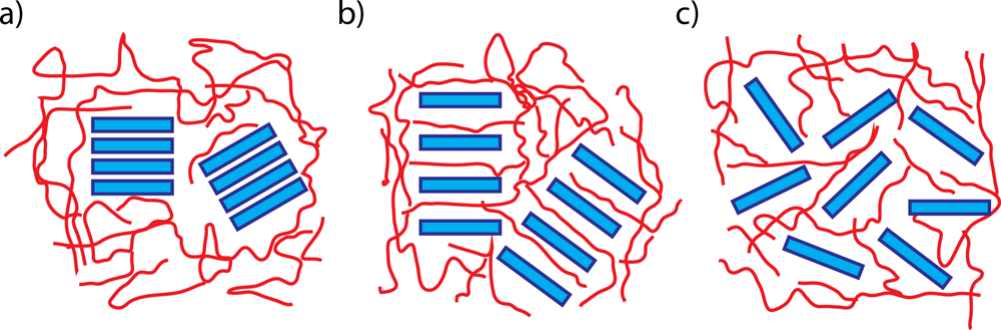
A schematic representation of the different layered silicate-organic composites. a) Phase-separated microcomposites, b) nanocomposites, and c) exfoliated nanocomposites.

Composite materials with small amounts of inorganic fillers dispersed in a continuous polymer phase have been extensively studied and have shown improved mechanical properties at low filler levels.^566^ However, the nature-inspired strategy of adding small amounts of organics to improve the properties of inorganic materials has not been widely explored. In general, three main types of polymer-crystallite layer composites can be considered based on the distribution of polymer chains and inorganic layer structures (Figure 77):^567^

- Polymer chains surround the micrometer-size layered crystallite composed of stacked individual silicate particles, so-called phase-separated microcomposites (Figure 77a).
- Polymer chains are intercalated in the interlayer space between the individual particles without disrupting the layered structure, so-called nanocomposites (Figure 77b).
- Polymer chains disrupt the order of the stacked individual particles and are separated, so-called exfoliated nanocomposites (Figure 77c).

#### 6.2.1 Fabrication of Cement-Polymer Nanocomposite Materials

**Figure 78:**
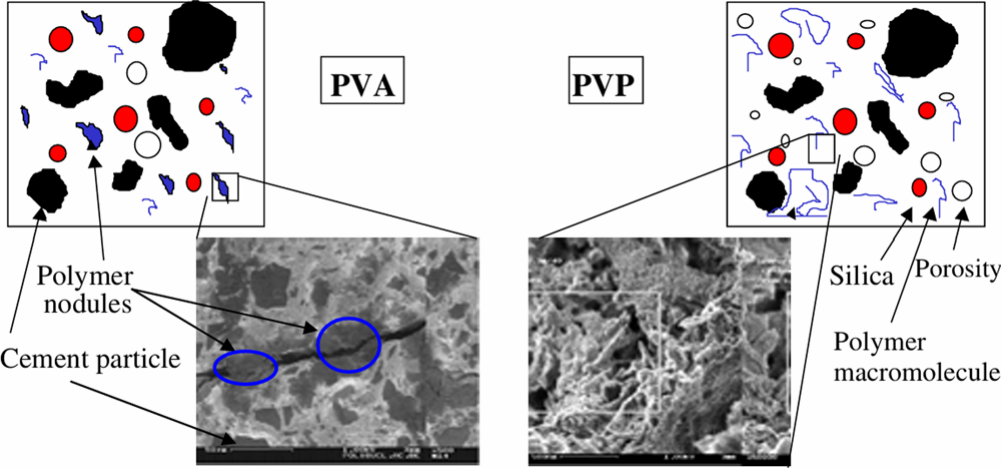
Upper part: schematic representations of the resultant microstructure of the composites obtained by using poly(vinyl alcohol) (PVA) or (poly(vinylpyrrolidone) (PVP). Lower part: SEM micrographs of polymer-modified cement pastes (<4 wt % polymer) for PVA and PVP. A dense hydrate microstructure with polymer nodules was observed in the presence of PVA. In the presence of PVP, a porous hydrate microstructure was noted, where polymer-rich nodules were absent. Reproduced with permission from ref (568). Copyright 2007 Springer Nature.

Cement hydrates and polymers often combine at the micrometer scale to form phase-separated microcomposites, which, in some cases, exhibit superior mechanical properties due to various mechanisms. For example, the use of small amounts of high molecular weight water-soluble polymers (poly(vinylpyrrolidone) (PVP) or poly(vinyl alcohol) (PVA)) increases the total fracture energy mainly by modifying crack growth.^568^ In the case of PVP, the fracture mechanism seems to be affected by the alteration of the microstructure of the C–S–H hydrates. Conversely, in the case of PVA, the presence of polymer-rich nodules seems to control and inhibit crack propagation (Figure 78).^568^

Going one step further and coupling the organics and cement hydrates not only at the millimeter and micrometer scale but also at the nanoscale, superior performance is expected. In the case of phyllosilicates, organic molecules have been successfully intercalated into them at the atomic level, resulting in smectite/polymer nanocomposites with good mechanical performance^566^ that have even been produced industrially.^569^ Based on the similarities between smectite clays and C–S–H, the intercalation of polymers between the nanometer C–S–H lamellae has also been pursued in recent decades with the goal of producing materials with superior mechanical properties.

**Figure 79:**
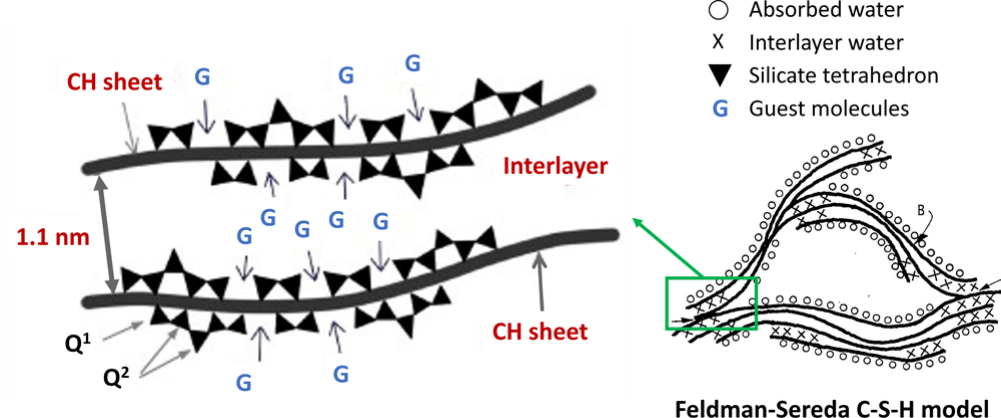
A schematic representation of the C–S–H nanostructure highlights the possible sites (G) for grafting guest molecules (organic and inorganic). Reproduced with permission from ref (574). Copyright 2008 Elsevier.

Matsuyama and Young pioneered the development of C–S–H/polymer nanocomposites in the late 1990s. They reported the incorporation of anionic (poly(acrylic acid) and poly(methacrylic acid)), cationic (poly(diallyldimethylammonium chloride)), and nonionic (poly(vinyl alcohol)) polymers into the C–S–H structure. The measured increase in the interlayer spacing was attributed to the incorporation of the organics between the C–S–H sheets^570−572^ Subsequent investigations by Mojumdar and Raki further corroborated these findings.^573^ They observed an increased interlayer C–S–H spacing when synthesized in the presence of poly(acrylic acid), which was again attributed to the incorporation of the polymer into the interlayer. Additionally, they reported an increase in the degree of polymerization of the silicate chains, as the Si–O vibration characteristic of Q^1^ tetrahedra was absent in the presence of poly(acrylic acid).^573^^29^Si magic angle spinning–nuclear magnetic resonance (MAS NMR) also elucidated this increase in the connectivity between the silicate tetrahedra in C–S–H due to the interaction with another organic species (e.g., polyethylene glycol, hexadecyltrimethylammonium, methylene blue, poly(acrylic acid)) with previously synthesized C–S–H powder.^574^ The organics were suggested to be grafted at missing bridging silica tetrahedra sites, incorporated in the C–S–H interlayer, or both (Figure 79).^574^

In contrast, Merlin et al. argued that C–S–H cannot swell like smectites, and therefore, it might be impossible for polymers to intercalate into the C–S–H layers, and the changes in interlayer spacing were simply due to the use of different drying methods in sample preparation.^575^ They concluded from their studies that the introduction of polymers into the interlayer space during C–S–H formation, either by precipitation or pozzolanic reaction, is ineffective. This agrees with Popova et al., who did not observe any significant structural changes in the C–S–H products by X-ray diffraction or 29Si MAS NMR.^576^ Nevertheless, the interaction between the inorganic C–S–H material and organic substances was not ruled out. Polymers still have the potential to adsorb on the surface and in the voids and to interact with the stacking order of the C–S–H sheets. Consequently, the resulting material is considered a mesocomposite in which the building blocks are not the individual C–S–H nanolayers but the C–S–H crystallites.^575^

Following the research of Matsuyama and Young and Merlin et al., an alternative approach was developed to produce cementitious nanocomposites by covalent bonding between the C–S–H and organic phases to induce hybridization. In this way, the dispersion of the organic phase and the bonding between the two phases, which are thought to be relevant to the fracture toughness of biominerals, could be improved. In this method, the bonding of the organic molecules to the C–S–H is achieved by silane functions.^577^ First, calcium organosilicates were prepared by a sol–gel process from the reaction of a Ca salt with various alkoxysilanes in a strongly alkaline medium. Condensation of the organotrialkoxysilane (R_9_Si(OR)_3_) in the presence of calcium ions resulted in a layered calcium silicate that intercalates small organotrialkoxysilane molecules in the interlayer, increasing the basal distance to values consistent with a bilayer chain arrangement of the organic moiety. However, it is important to note that the structure of the inorganic portion of the nanocomposite differs significantly from the calcium silicate hydrate (C–S–H) structure.^577^

**Figure 80:**
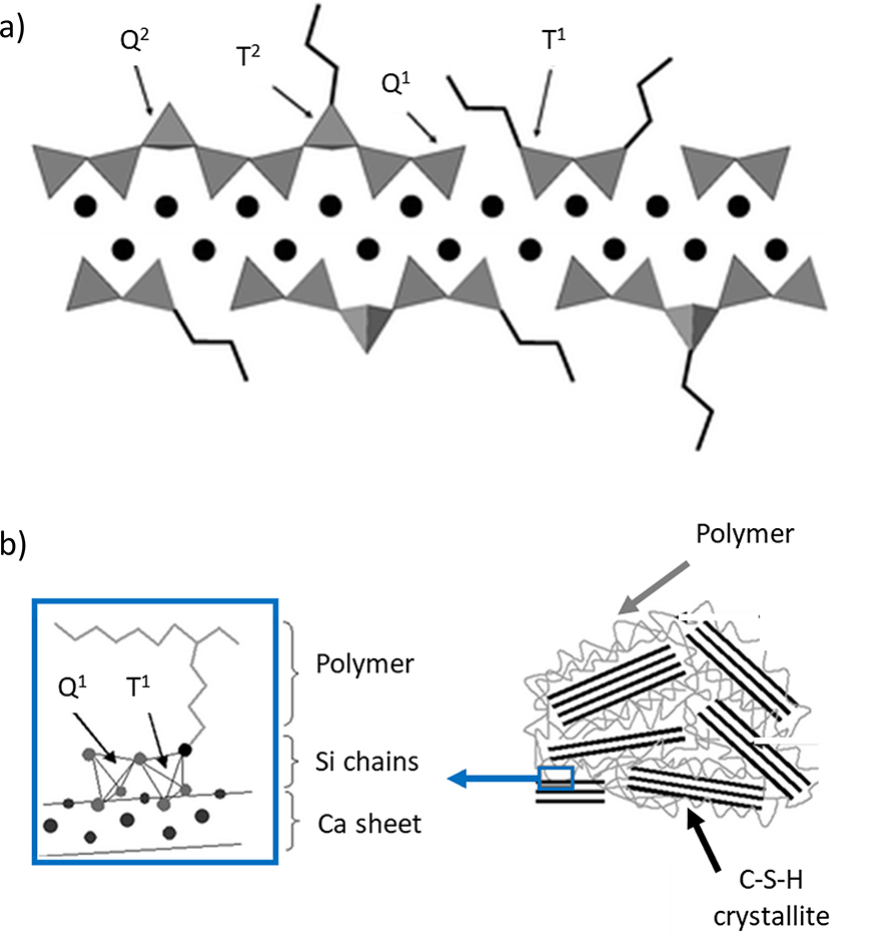
a) A schematic representation of a layer of hybrid calcium silicate materials with the incorporation of organotriethoxysilane. Some silicate tetrahedra (Q) have been replaced by trialkoxysilane (T), leading to the incorporation of organic moieties in the interlayer. (Black circles = calcium atoms). Reproduced with permission from ref (578). Copyright 2006 Royal Society of Chemistry. b) The proposed structure explains the formation of a covalent linkage between the T-silane and the C–S–H crystallites in the composites. Reproduced with permission from ref (579). Copyright 2007 Royal Society of Chemistry.

Posterior attempts aimed to obtain hybrid nanocomposites without disrupting the inorganic C–S–H framework by the coprecipitation of tetraethoxysilane (TEOS, Si(OC_2_H_5_)_4_) and organotrialkoxysilane mixtures in the presence of calcium in aqueous/ethanolic basic media. NMR (^29^Si CP-MAS and ^1^H–^29^Si HETCOR) confirmed that the silanes were incorporated in the silicate chains of C–S–H (Figure 80a). Importantly, this incorporation preserved the integrity of the inorganic structure for hybrid materials synthesized with values up to 40% of organosilanes. The incorporation failed in the case of large-sized organic groups, resulting in phase separation and leading to a mixture of inorganic C–S–H and 100% organosilane calcium hybrid phase.^578^

Using the sol–gel synthesis method, larger polymeric structures (*M*_w_ > 300 000 g/mol) can be covalently linked to C–S–H by grafted silane groups on the polymer chains.^579^ However, the linkage seemed to occur only on the surface of C–S–H crystallites, and incorporation in the interlayer was excluded (Figure 80b). More recent studies have revealed that poly(diallyldimethylammonium chloride) (PolyDADMAC) affects not only the C–S–H nanostructure by intercalating between the lamellae but also the C–S–H crystallite packing density, which proved to be detrimental to the material properties by decreasing Young’s modulus and the hardness measured by nanoindentation.^580^ Similar results were also reported for PVA^581^ and poly methacrylic acid^582^ by these authors.

In-situ polymerization has also been explored as a method to produce cement organic–inorganic nanocomposites.^583^ Here, C–S–H was synthetically produced by mixing stoichiometric amounts of calcium oxide and silica in the presence of aniline monomers polymerized by an oxidant. The C–S–H/polyaniline composites exhibit a higher silicate polymerization, depending on the Ca/Si ratio used. The authors concluded that, although the monomers were intercalated within the nanostructure of C–S–H (increased basal spacing), once polymerization occurred, the monomers might have been detached from the interlayer (decreased basal distance) to form polyaniline. In terms of performance, the initial response of the C–S–H/polyaniline nanocomposites subjected to dynamic mechanical analysis seemed to be improved compared to the polymer-free system.^584^ Later, researchers further confirmed the enhanced properties of C–S–H/polymer composites achieved via in situ polymerization approaches. They successfully created an inorganic–organic interlocking microcomposite with a remarkable improvement in compressive strength (15%) and flexural strength (200%). This was attained by in situ polymerization of acrylate monomers, with optimization of the monomer-to-cement ratio and initiator-to-monomer ratio.^585^

The feasibility of developing polymer-cement composites has been demonstrated, and many studies have indicated a significant improvement in flexural strength. However, the incorporation of polymers often has a negative effect on the compressive strength and modulus of these composites.^586^ To overcome this challenge, a homogeneous distribution of organic compounds can be achieved by integrating them between the individual C–S–H layers, thus ensuring association at the nanoscale. Nevertheless, modifying the nanostructure of C–S–H by incorporating polymeric additives between the individual C–S–H lamellae is challenging, and therefore, considerable effort must be devoted to unraveling the complex interaction between the polymer and the cement hydrates, both from an experimental and theoretical perspective (Section 6.2.2).^587^

#### 6.2.2 Organic–Inorganic Interactions

In general, the interface between the inorganic and organic phases plays a crucial role in the bulk properties of the hybrid system. Therefore, the identification of organic additives that can ensure a strong bond between the two phases is the first step toward improving C–S–H composites. While numerous publications have addressed the adsorption of polymers, primarily PCEs, into the cement phases, most have focused primarily on evaluating the effect on the paste’s particle dispersion and rheological properties.^588^ From the experimental perspective, only a few publications focused on understanding the specific absorption of organic additives to C–S–H surfaces due to the obvious instrumental constraints.

Some of the most groundbreaking experimental studies that shed light on the specific interaction between organics and C–S–H surfaces are the AFM investigations by Flatt et al.^589^ This study showed that PCE superplasticizers greatly reduce the strong ionic correlation forces that exist between C–S–H surfaces, and also provided a model for the conformational adsorption of PCEs on C–S–H surfaces. Another notable contribution is the multimethod approach by Ferrari et al., which combined the insights obtained from rheology, adsorption, AFM, and ζ-potential measurements. This approach transitioned from macroscopic rheological observations to exploring details of superplasticizer behavior at the solid–liquid interface (i.e., adsorption and dispersion forces).^590,591^ Furthermore, we wanted to highlight the work of Picker et al., which introduced a novel application of the phage display method, commonly used in biology, to identify the “must-have” characteristics of strong absorbing additives onto C–S–H surfaces.^592^ These endeavors collectively illuminate the complex and critical relationship between organic additives and C–S–H surfaces, marking, as we discuss below, important advances in our understanding of these interactions.

On a macroscopic scale, an improvement in the flexural strength and a slight reduction in the compressive strength are commonly observed in polymer-cement mortars.^586^ Yet, the mechanism underlying the improved behavior and molecular insights into the polymers’ role remains to be discovered after more than 40 years of research.^563^ Most polymers currently used in the cement industry work mainly through electrostatic interactions.^590^ However, the most recent investigations (see below) have evidenced that enhanced interfacial adhesive strength is ascribed not only to the formation of ionic and covalent bonds but also to the specific adsorption of polymers through H-bonds and their integration into defective regions of the silicate chains within C–S–H.

**Figure 81:**
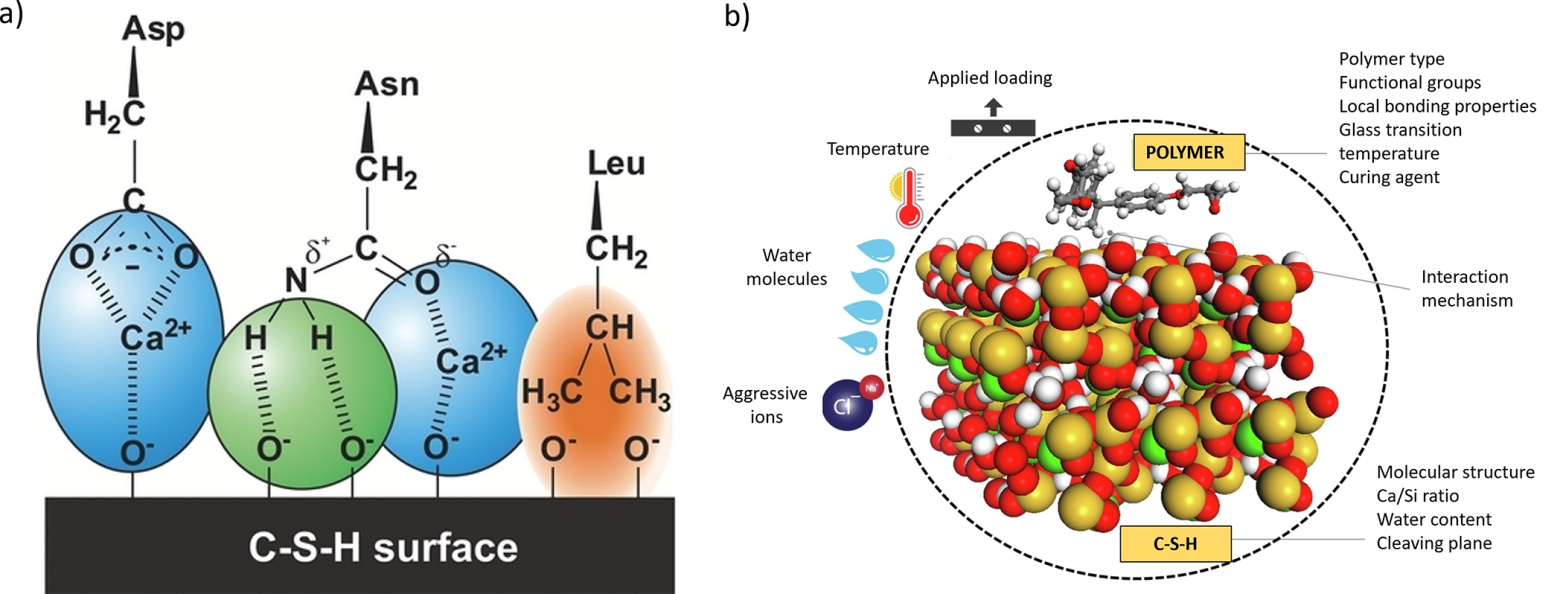
a) Proposed interaction model of the selected amino acids with the C–S–H surface. Negatively charged residues (Asp = aspartic acid) interact electrostatically via Ca^2+^ bridged ions (left), amide groups (Asn = asparagine) via both H-bonds and Ca^2+^ bridging due to a partial negative charge on the oxygen and hydrophobic residues (Leu = leucine) via van der Waals interactions (right) Reproduced with permission from ref (592). Copyright 2014 John Wiley & Sons. b) The most relevant factors influencing the polymer-cement interactions. Reproduced with permission from ref (596). Copyright 2022 Elsevier.

The selection of an appropriate polymeric additive with specific properties that ensure strong interfacial interaction with C–S–H surfaces is critical to the development of nanocomposites with superior performance. Experimentally, Picker et al.^592^ used a biological method known as phage display, which applies modified bacteriophages to detect the peptides that bind strongly to a substrate. Their investigation explored the interaction of C–S–H surfaces with a wide range of synthesized peptides, using different Ca(OH)_2_ concentrations to confer different properties to the surfaces. They concluded that an organic additive that strongly absorbs onto C–S–H should have a negatively charged part to adsorb onto calcium counterions, H-donating and accepting functions for interactions with silanol groups in C–S–H and a hydrophobic part (Figure 81a).^592^ Importantly, their conclusions aligned with MD simulations conducted in later studies.^593,594^

Due to the complexity of the cement hydration process, the interactions between the organic and inorganic phases, and the highly ionic solution in which all of these interactions occur, computational methods have been positioned as a powerful tool to provide valuable information about cement nanocomposites.^595^ Recently, they have helped to understand the physical and chemical interaction mechanisms between polymer and cement hydrates and to predict the properties of hybrid cement-based nanocomposites.^596^ Typically, computational studies first develop a cement-polymer nanocomposite model and study the interface between the two phases. They then perform simulated mechanical tests that provide information on the mechanical properties and molecular response in different directions when a load is applied. The trickiest part of this approach is constructing an accurate nanocomposite model and accurately describing the multiple interactions between C–S–H, polymers, water, and ions in solution. This is a challenging task because several key factors influence polymer-cement interactions that would need to be considered in simulations to develop rigorous models (Figure 81b).^596^ For a comprehensive overview of the most pertinent computational research in this field, we direct the reader to the review by Bahraq et al., which covers the most relevant computational work in this area.^596^

Herein, we have included the most relevant results obtained by computational methods used to study the organic–inorganic interface of various cement-polymer composites. We consider these results as essential information for the advancement of C–S–H/polymer composites since they characterize the material’s interfacial properties and mechanical response when subjected to uniaxial stress, both at the molecular level.

Hou et al. found that incorporating PEG, PVA, and PAA increases the interfacial binding energy between the polymers and the C–S–H substrate and, consequently, the cohesive strength and ductility of the composites.^595^ Simulations showed that calcium ions near the C–S–H surface play a crucial role in the strengthening of the interface by connecting the functional groups of the polymers to the oxygen in the C–S–H,^595^ which other computational studies have also evidenced.^597−599^ The interfacial bonding is further reinforced through distinct mechanisms. First, the oxygen atoms in the functional groups offer sites for forming H-bonds with the C–S–H substrate. Additionally, the polymers heal defective regions within the silicate chains in C–S–H.^595^ Thus, the polarity of the functional groups and the diffusivity and aggregation tendency of the polymers can significantly affect the connection between the polymer and the cement.^594^

**Figure 82:**
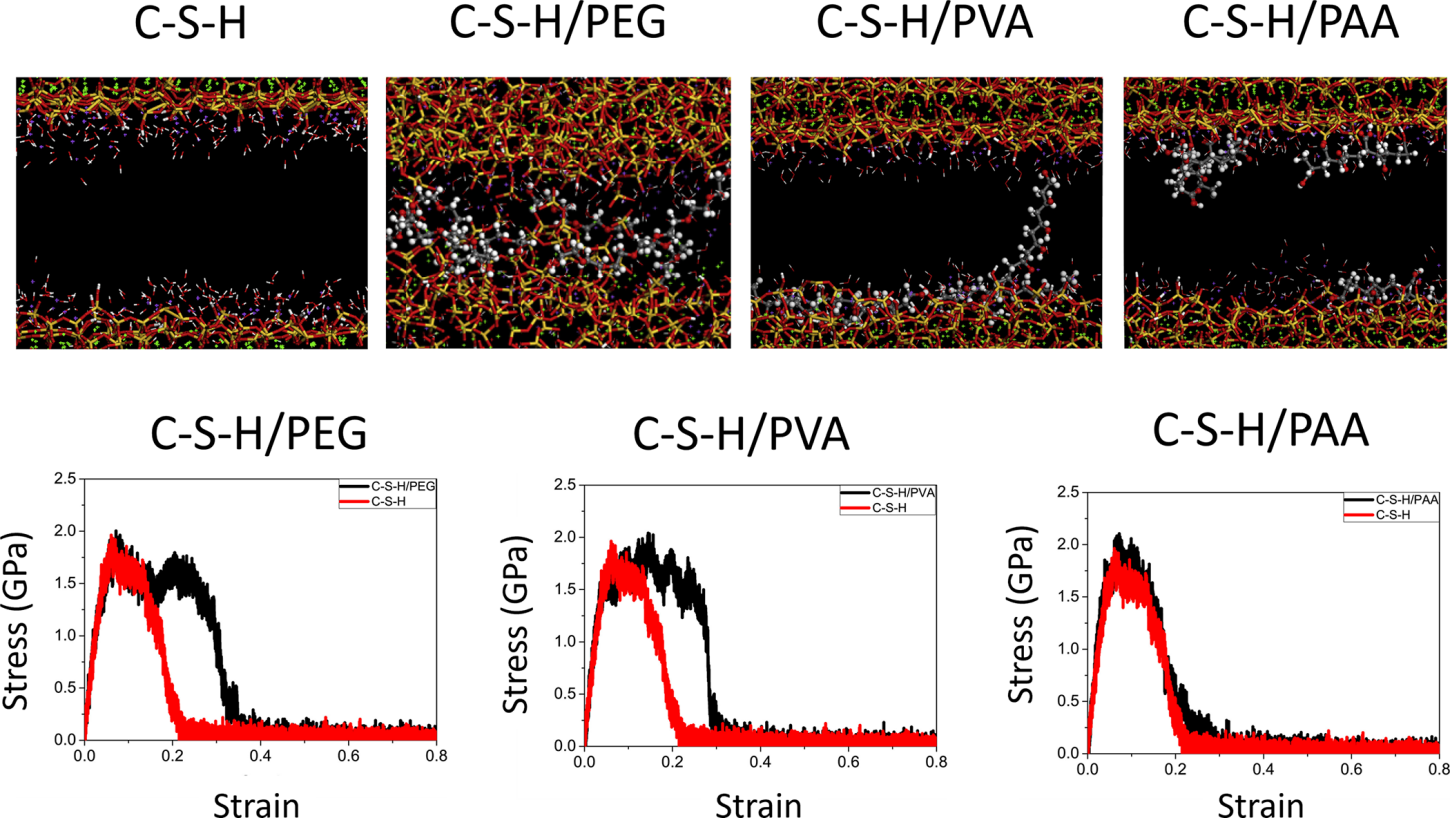
Upper row: snapshots of the configuration of pure C–S–H, C–S–H/PEG, C–S–HC–S–H/PVA and C–S–H/PAA nanocomposites in z direction tensile process (strain level = 0.40 Å/Å). Lower row: stress–strain relations of C–S–H with C–S–H/PEG, C–S–H with C–S–H/PVA and C–S–H with C–S–H/PAA under tension loading along *z* direction. Reproduced with permission from ref (595). Copyright 2019 Elsevier.

The interplay between the different molecular interactions resulted in the following order in the calculated interfacial binding energies (E): E(PAA) > E(PVA) > E(PEG). Interestingly, this ranking correlated with the highest values of Young’s modulus, tensile strength, and fracture strain for the case of PAA, followed by PVA and PEG.^595^ In terms of ductility, there was a significant increase when the polymers were intercalated, with PEG showing the highest value (Figure 82).^595,600^ This enhancement has been attributed, based on the simulations, to the reaction between C–S–H and PEG, in which Ca^2+^ ions from the substrate are reported to break the C–O bonds, allowing a Ca–C connection and, thus, a stronger bond with C–S–H.^601^

All these recent advances in understanding the molecular mechanisms by which polymers improve the properties of cement-based materials certainly provide valuable insights. These advances can contribute to the development of highly efficient polymer-cement composites using a bioinspired approach to address brittleness at the nanoscale. The goal is to achieve this while maintaining or even improving the material’s compressive strength. Although the bottom-up approach for manipulating the C–S–H nanostructure at a large scale remains a central challenge, successful feasibility has been demonstrated in microsized systems, as outlined in Section 7.2.

## 7 Controlled Growth and Self-Assembly Applied to C–S–H

While top-down approaches, such as reducing pores and defects, have demonstrated success on a larger scale with Macro Defect Free (MDF) cement,^602^ the exploration of bottom-up approaches involving the self-assembly of C–S–H nanoparticles into ordered and defect-free structures is still in its early stages. These self-assembled structures have the potential to exhibit exceptionally high flexural and compressive strength and could even reduce the need for steel reinforcement, thereby mitigating associated corrosion problems. This concept is up-to-date science fiction for material scientists, and thus, our aim in this section is to present and discuss the achievements already made by the controlled self-assembly of C–S–H nanoplates, yielding to mesocrystals. To set the stage, we will first introduce some basic concepts about mesocrystalline structures in general and particle-based nonclassical crystallization processes. Finally, we will present the controlled growth and self-assembly of C–S–H, a process pioneered by our group. This has resulted in the formation of a C–S–H mesocrystalline structure with remarkable mechanical properties.

### 7.1 Mesocrystalline Structures

**Figure 83:**
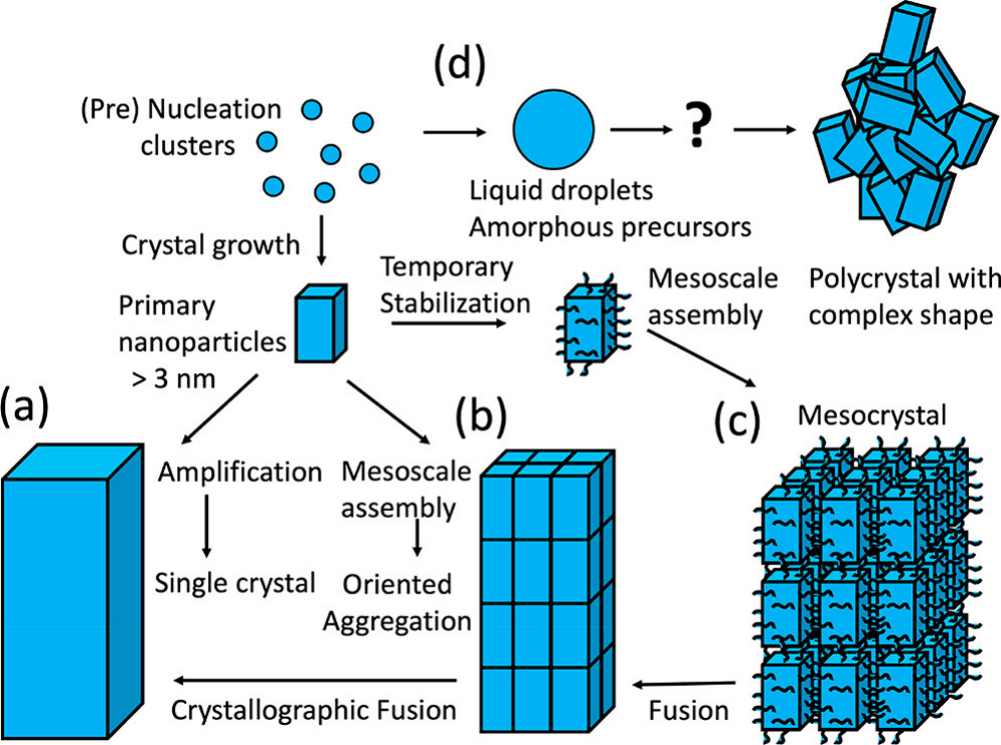
Schematic representation of classical and nonclassical crystallization. (a) Classical crystallization pathway involving layer-by-layer growth by atom/ion/molecule addition, (b) oriented aggregation of primary nanoparticles forming an iso-oriented crystal, (c) mesocrystal formation via self-assembly of primary nanoparticles covered with organics, and (d) crystallization via liquid droplets or amorphous precursor phases. For clarity, please note that the nanoparticles were drawn to the same size. Reproduced with permission from ref (437). Copyright American Chemical Society. Figure partially adapted with permission from ref (438) and ref (603) with permissions.

To understand the self-assembly of C–S–H platelets, basic understanding of the concepts of mesocrystals and particle-based nonclassical crystallization are essential.^125^ For extensive details on nonclassical crystallization pathways (Figure 83)^437^ the reader is referred to ref.^126^

As outlined in Section 3, the crystallization pathway involves the initial formation of a nucleus, either through monomer addition according to classical nucleation theory,^8^ or via prenucleation clusters along the PNC pathway.^136,138,145^ This nucleus has the potential to crystallize later, initiating layer-by-layer growth on exposed crystal faces, per the Kossel-Stranski theory.^604^ This growth (Figure 83(a)) occurs as building units attach to the growth face, followed by surface diffusion to the site of attachment where the highest energy gain is achieved, i.e., steps and kinks. Various theories, such as the BCF theory,^605^ Franks kinematic theory,^606,607^ classical Wulff construction,^418^ or more recently, the symmetry-based kinematic theory,^608^ can be applied to estimate and predict the crystal shape. It is crucial to note that, according to classical crystal growth models, the building units consist of atoms, ions, or molecules.

However, the primary nucleated nanoparticles may not only grow but also interact with each other, leading to a nonclassical crystallization process. In this scenario, these nanoparticles can serve as building units. When nanocrystals interact in a controlled manner through equivalent crystal faces, they have the potential to form a mosaic superstructure through oriented assembly or oriented aggregation, illustrated in Figure 83(b) where all nanocrystals align with each other. Nevertheless, such structures present a drawback—they exhibit a high internal surface, which is energetically unfavorable. Consequently, the already-oriented nanocrystals tend to fuse into a single crystal, thereby minimizing internal surface energy. This phenomenon, known as oriented attachment (OA), is a frequently observed mechanism in crystal growth.

Another mode of interaction for the initially formed nanocrystals occurs when they are weakly colloidally stabilized, either through charge or molecules, as depicted in Figure 83. In cases where a weak attraction prevails, nanoparticles can engage in a controlled interaction to create a superstructure. In this arrangement, all nanocrystal building units align in a crystallographic register, resulting in a diffraction pattern resembling a single crystal. Such structures are called mesocrystals, an abbreviation for mesoscopically structured crystals.^413,438,443−445,447−449,609^ Despite reduced spaces between the nanoparticle building units (Figure 83(c)), mesocrystals retain a significant inner surface area.

Mesocrystals are precisely defined as nanostructured materials that exhibit distinct indications of comprising individual nanoparticle-building units with a well-defined order on the atomic scale, as evidenced by the presence of a substantially sharp wide-angle diffraction pattern. While some defects may interrupt the long-range order, giving rise to the formation of a mosaic structure within the solid, the clear diffraction pattern establishes the organized nature of the mesocrystal.^446^

**Figure 84:**
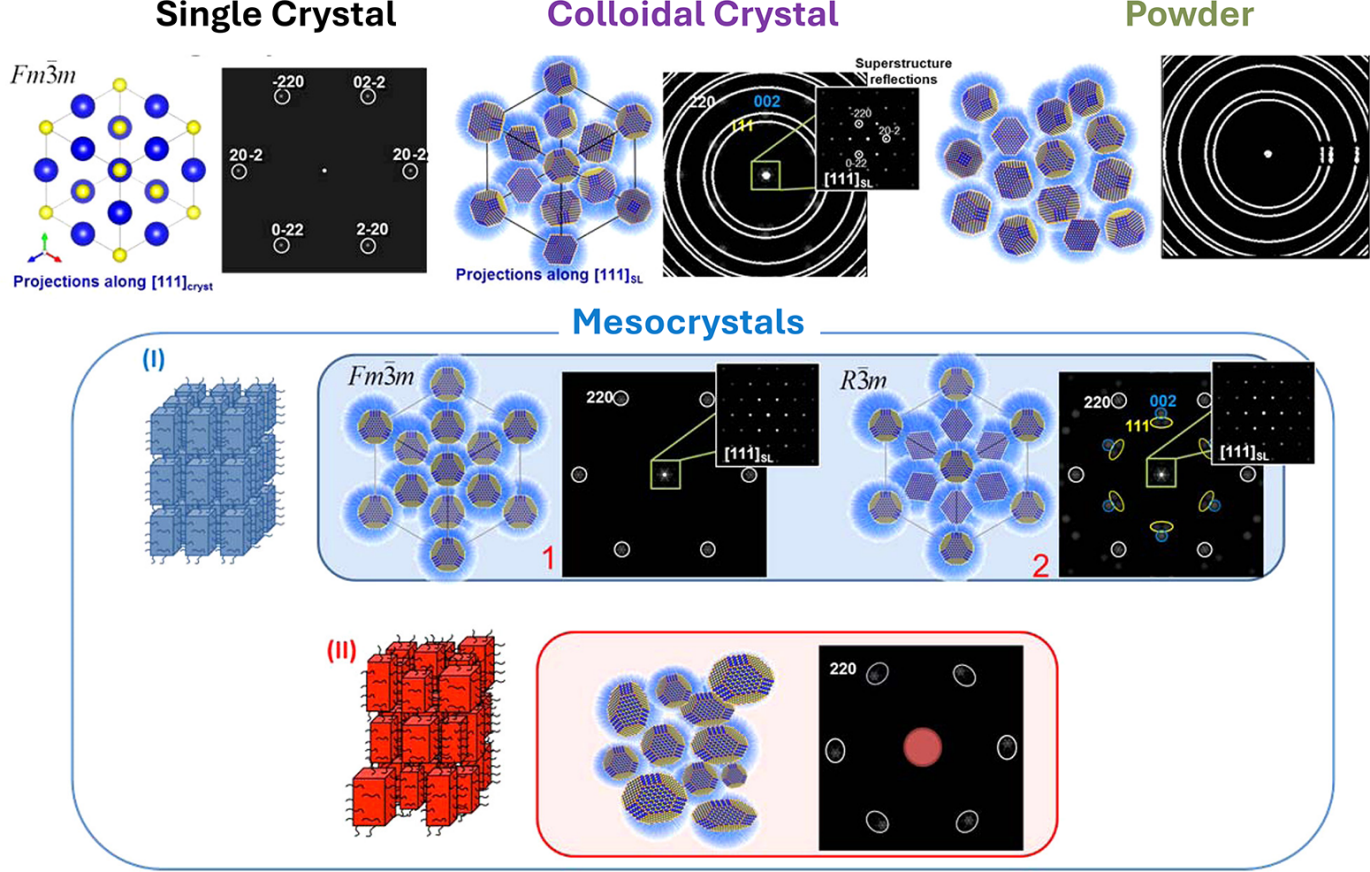
Schematic illustration of different types of crystalline materials with the corresponding diffraction patterns. For illustrational purposes, the crystalline material (nanoparticles) has a rock salt-type crystal structure (S.G. *Fm*3̅*m*), and in the case of colloidal crystals, the crystalline nanoparticles are stabilized by organic molecules (blue shell) and arranged in a face-centered cubic (fcc) superlattice (S.G. *Fm3̅m*). (Top row) Single crystal, disordered colloidal aggregates e.g., “‘powder’”, colloidal crystal with the corresponding diffraction patterns. (Bottom row) Mesocrystals type II (red frame) and type I (blue frame)—colloidal crystals with mutually oriented monodisperse nanocrystals (characterized by a single crystal-like diffraction pattern in the small angle region and a single crystalline (1) or texture-like (2) pattern in the wide-angle region); type II—colloidal aggregates with mutually oriented polydisperse nanocrystals with a possible certain degree of orientational mismatch. Reproduced from ref (443) under a 4.0 Creative Commons Attribution License (CC BY 4.0). Copyright 2015 American Chemical Society. https://creativecommons.org/licenses/.

The differences between a single crystal, colloidal crystal, and mesocrystal are illustrated in Figure 84. A single crystal exhibits a wide-angle diffraction pattern with spots that signify order on the atomic scale but lacks diffraction peaks at small-angle due to the absence of long-range order. Conversely, a colloidal crystal displays a small-angle diffraction pattern with spots resulting from long-range order. Yet, only diffraction rings indicate random atomic-scale order, as the orientation of each crystal is random. Additionally, a crystalline powder presents identical diffraction rings in the wide-angle data due to the random crystal orientation. Still, it lacks a small-angle diffraction signal due to the absence of long-range order. Mesocrystals combine the diffraction patterns of single and colloidal crystals, and the two types are distinguished. In highly ordered mesocrystals (type I), diffraction spots are observed in both small and wide-angle patterns. In the more prevalent type of mesocrystals (type II), only wide-angle diffraction spots are present, omitting the small-angle range due to variations in nanocrystal sizes. In such cases, additional evidence for nanocrystal building units is typically sought through electron microscopy.

The orientation of nanocrystalline building units is typically not perfect, resulting in arced wide-angle diffraction patterns. Recently, advancements have allowed for the quantitative determination of the 3D structure and its defects in a gold mesocrystal through coherent X-ray diffraction imaging.^610^ Despite this, the orientation of building units in a mesocrystal is generally high enough to enable the fusion of these units through oriented attachment. This process eliminates surface molecules and leads to the formation of a final single crystal, albeit one that may possess defects. The occurrence of this fusion process could be revealed for iron oxides^611^ and dl-alanine mesocrystals^612^ through electron microscopy and small-angle neutron scattering, respectively.

Mesocrystalline materials offer several advantages. First, they possess a high inner surface area, which is particularly advantageous for catalytic applications.^613−615^ Additionally, they combine the properties of nanocrystals with micrometer size, rendering them easy to handle while avoiding concerns related to nanotoxicity. Importantly, the functional properties of the nanocrystals, such as quantum-size effects, surface plasmon resonance, or superparamagnetism, remain intact and may even be enhanced through the coupling of multiple nanocrystals.^616^

As discussed in Section 6.1, mesocrystalline structures manifest in nature through evolution-optimized biominerals. Various organisms leverage mesocrystal structures, including magnetotactic bacteria, mussels (nacre), sea urchins (spines), corals, and even humans (bones).^443,617^ In most cases, the mechanical properties of these mesocrystalline hybrid structures are optimized by a hard–soft arrangement, with ordered nanocrystals separated by polymeric or amorphous matter. One remarkable example illustrating the enhancement of mechanical properties through a hierarchical mesocrystalline structure is nacre, which is 3000 times more fracture-resistant than aragonite, constituting 95% of the material.^502^ Considering the need to improve the mechanical properties and overall durability of cementitious materials to improve their sustainability, developing a bioinspired mesocrystalline structure using C–S–H building units as an inorganic component, combined with specific polymeric additives presents a promising opportunity. These additives play a dual role, initially ensuring the colloidal stabilization of the building units and subsequently driving the controlled destabilization to yield self-assembled ordered C–S–H structures.

### 7.2 Ordered C–S–H Nanoscale Composites

**Figure 85:**
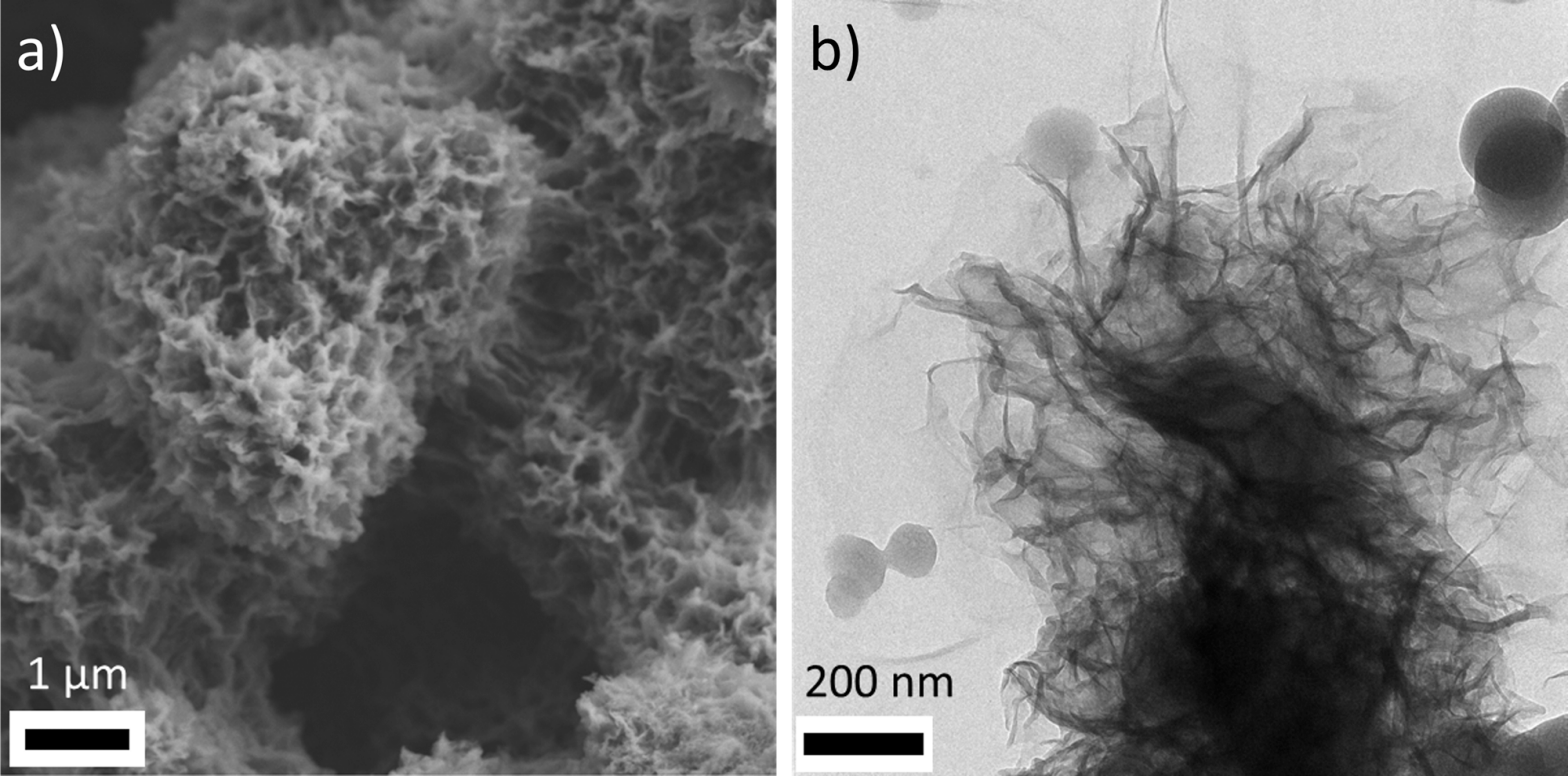
a) Scanning electron micrograph of C–S–H microstructure showing the unordered aggregate of C–S–H nanoplatelets. b) A transmission electron microscopy micrograph shows the disordered C–S–H networks composed of lamellar subunits. Images courtesy of Marc Staiger (PhD candidate at the University of Konstanz).

Nature offers valuable insights into optimizing structures for optimal mechanical performance. One such structural principle is the brick-and-mortar arrangement, involving hard and brittle inorganic bricks paired with soft but ductile organic mortar. Achieving similar structurization in cementitious materials could enhance their performance. However, a significant challenge arises from the pronounced interaction tendency of C–S–H, which adheres to various surfaces, including itself.^79^ This tendency often results in the formation of disordered aggregates, as depicted in Figure 85. Conventional dispersants can be used to prevent aggregation of C–S–H platelets.^244^ However, to achieve perfectly ordered structures, it is necessary to use optimal additives that minimize any uncontrolled aggregation.

Preventing undesired aggregation of the platelets involves a two-step process: first, colloidally stabilizing C–S–H nanoplatelets (see Section 3.4.2 and 6.2), and then driving their controlled assembly into an ordered mesocrystalline superstructure, promising superior mechanical properties. Due to the varying charges (both negative and positive) on different facets of the C–S–H nanoplatelets, with an overall charge dependent on pH, electrostatic stabilization proves unpromising. Instead, the most effective method for stabilization is steric stabilization using polymeric molecules. However, the challenge lies in finding C–S–H-specific molecules that selectively adhere to C–S–H under specific conditions. Currently, the most prevalent stabilizers in the construction industry are polycarboxylate ethers, primarily operating through Coulomb attraction between opposite charges of C–S–H and the polymer.^590^

As described in the previous section, we selected the specific binding molecules using a biological method called phage display. This method employs phages containing a combinatorial library of peptides of a certain length on their heads to assess their interaction with surfaces. Through repeated adsorption cycles, peptides with the highest affinity for a given surface are identified.^618^ Despite the practical challenge posed by the highly alkaline pH of C–S–H preparations, where hydrolysis might destroy phages and peptides, phage display libraries have displayed remarkable resistance to elevated pH levels. Tests conducted at pH 12.5 revealed that a sufficient number of phages survived, allowing the identification of the best binding sequences through repeated biopanning cycles.^592,619^

As the Ca to Si ratio in C–S–H is pH-dependent, with the Ca content increasing as the pH rises, the charge of C–S–H undergoes a transition from negative at a low pH of 8.9 (at a ratio of 0.66) to positive at a ratio of 1.7 and pH 12.5.^592^ his shift is distinctly reflected in the amino acid composition of the peptides identified as the best binders, as detailed (see Table 3).

**Table 3:** Groups of Identified Amino Acids by Phage Display at Various Conditions for a 12 Amino Acid Peptide^592^

Sample	Negatively charged (%)	Positively charged (%)	H-bond formers (%)	Hydrophobic (%)
C–S–H 0.66	7.5	34.6	26.2	31.7
C–S–H 1.0	9.2	4.8	47.4	38.6
C–S–H 1.5	10.1	7.1	28.6	54.2
C–S–H 1.7	13.1	4.6	35.2	47.1

While for C–S–H 0.66, it is evident that positively charged amino acids are abundant, for C–S–H 1.0, 1.5, and 1.7, surprisingly, negatively charged amino acids do not dominate for the positively charged C–S–H particles, as one might expect due to the electrostatic attraction between opposite charges. Instead, a significant amount of amino acids capable of forming H-bonds is detected, regardless of the C–S–H charge. This important result demonstrates that H-bonds play a significant role in binding molecules to the C–S–H surface. Additionally, a high amount of hydrophobic amino acids is observed in the best-binding peptides. While in the original publication,^592^ van der Waals binding of the hydrophobic amino acids to the C–S–H surface was mentioned, the role of the hydrophobic amino acids could also involve the folding of the peptide chain to reach an optimal conformation for binding of the charged and H-bond forming amino acids to the C–S–H surface. However, this could only be revealed through molecular modeling. Remarkably, three peptide sequences were responsible for more than 60% of the best-binding peptides across all investigated C–S–H samples. In the case of C–S–H 1.5, the best-binding peptide sequence alone was composed of 64% of the best-binding peptides.^592^ This demonstrates a clear selectivity for certain peptide sequences.

Although these results identified the best binding peptide sequences, peptides are not stable against the hydrolysis of the amide bonds at high pH. Thus, the design of polymers with attached binding peptides would be impractical for their use in the construction industry. The binding concept needs to be abstracted and transferred to C–C bond-based polymer backbones resistant to hydrolysis at high pH. Based on the results from the phage display study,^592^ as we discussed earlier, promising C–S–H binding polymers should have negatively charged groups that bind Ca^2+^ on the C–S–H surface, as also shown in simulations.^597^ Importantly, the polymers should also incorporate hydrogen donors and acceptors to maximize the binding. Fortunately, commercial polymers that meet these requirements are available.

**Figure 86:**
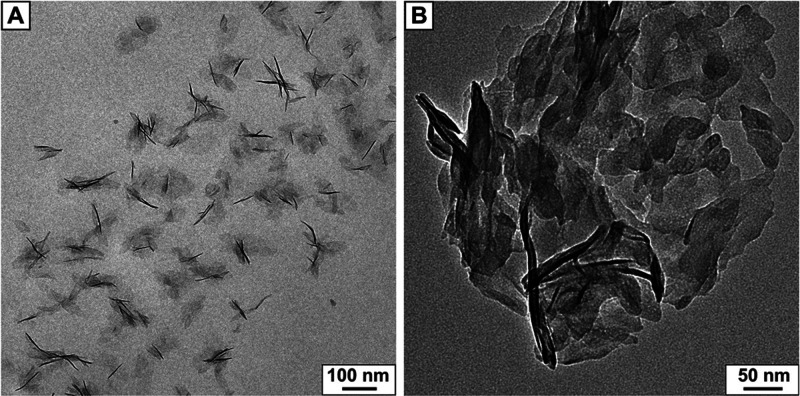
TEM analysis of C–S–H. A) Cryo-TEM of colloidally stabilized C–S–H crystallites at pH 12 and B) TEM analysis of aggregated C–S–H crystallites in the absence of stabilizing agents. The approximate literature known size of 60 × 30 × 5 nm^3,57^ can be assumed in both micrographs. Reproduced from ref (13) under a Creative Commons Attribution Non-Commercial License 4.0 (CC BY-NC). Copyright 2017 American Association for the Advancement of Science. https://creativecommons.org/licenses/by-nc/4.0/.

In a follow-up study, copolymers of poly(acrylamide-*co*-acrylic acid) (PAAm-*co*-PAA) containing a hydrogen bond donor and acceptor in the acrylamide group and poly(1-vinylpyrrolidone*-co*-acrylic acid) (PVP-*co*-PAA), with a hydrogen bond acceptor were used for the stabilization of C–S–H immediately after formation.^13^ Indeed, the colloidal stabilization of C–S–H nanoplatelets after their formation was achieved through the use of PAAm-*co*-PAA as well as by PVP-*co*-PAA. This was demonstrated by analytical ultracentrifugation (AUC) in solution and visualized by TEM, as illustrated in Figure 86A, in contrast to the aggregated sample formed in the absence of the polymers (Figure 86B).

The few aggregates observed in Figure 86 are likely drying artifacts since AUC detected no aggregates. The stability of the dispersions depends on the polymer and its concentration, offering various options for tunability.^619^ For example, the stability of the C–S–H dispersion at pH 12 in the presence of PVP-*co*-PAA (96.000 g/mol) exceeded 6 months at 1 g/L, but it was reduced to a few hours at 0.1 g/L. Whereas PAAm-coPAA with considerably higher molecular weight (200.000 g/mol and 520.000 g/mol) stabilized the dispersion for several months at 1.0 g/L and 1 day at 0.1 g/L.

**Figure 87:**
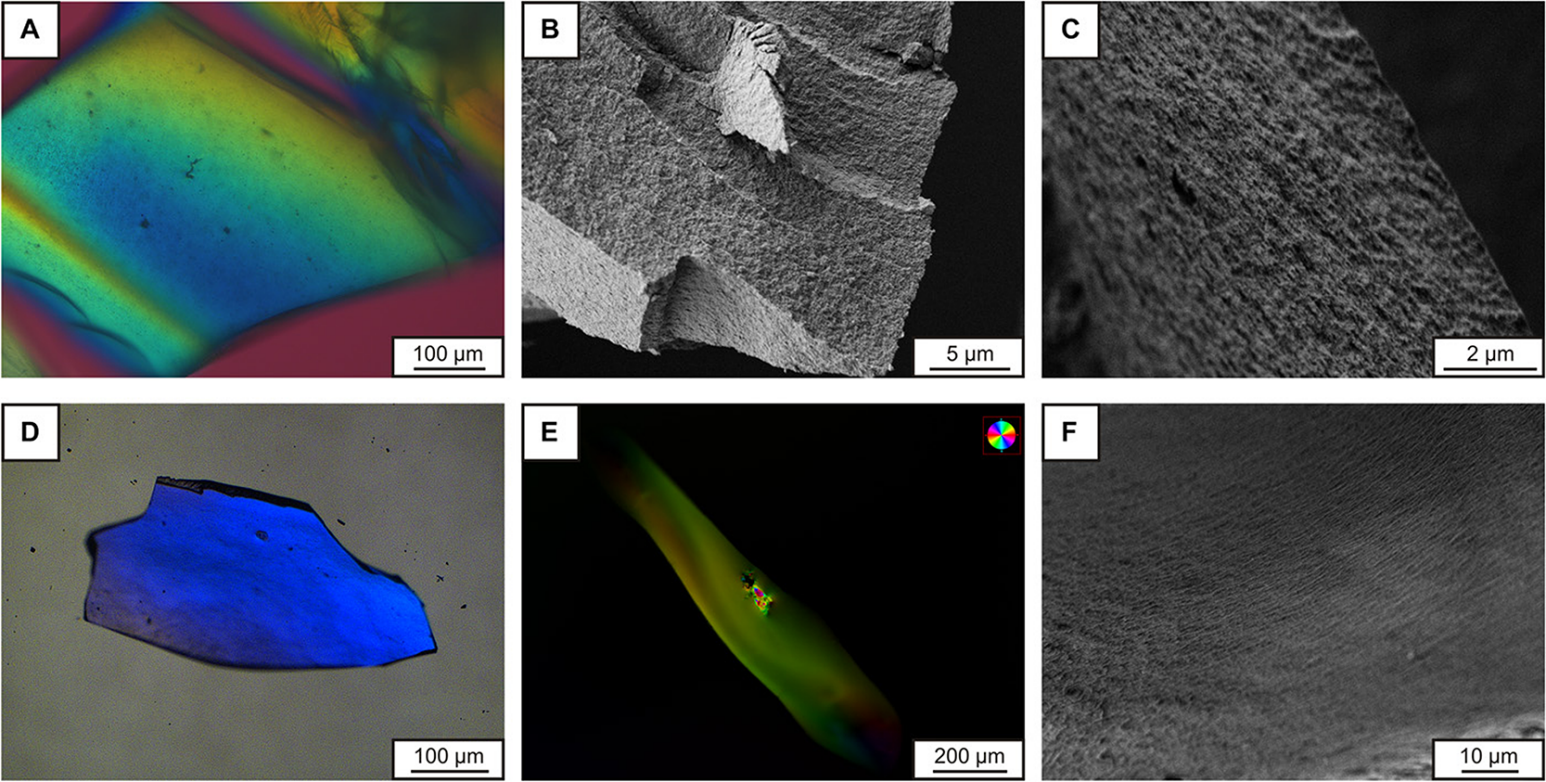
Polarized optical and scanning electron micrographs of C–S–H mesocrystals. A) to C) were obtained from approach A, and D) to F) from approach B. For A), D), and E), the same colors indicate the same orientations. The POM analysis suggests a long-range order of the agglomerated C–S–H crystallites over several hundreds of micrometers. B) reveals a secondary structuring of the C–S–H superstructures, whereas C) and F) show the alignment of the single C–S–H crystallites into layers, and no microporosity can be detected. Reproduced from ref (13) under a Creative Commons Attribution Non-Commercial License 4.0 (CC BY-NC). Copyright 2017 American Association for the Advancement of Science. https://creativecommons.org/licenses/by-nc/4.0/.

Raising the pH from 12 to 12.8 decreases the colloidal stability of C–S–H. This occurs due to the desorption of the polymer from the particles, leading to slow nanoparticle aggregation as demonstrated by dynamic light scattering data and the observed sedimentation of mesocrystals at the bottom of the reaction vessel after 1–3 days (Approach A).^13^ Alternatively, the pH can be directly adjusted to pH 13 after synthesis to facilitate the formation of the mesocrystals (Approach B). This slow aggregation process, triggered by decreasing steric nanoparticle stabilization, is important for achieving ordered mesocrystals. In a slow self-assembly scenario characterized by weak attractive forces between the particles, the nanoparticles have the chance to find their optimum position and orientation within the particle aggregate. This result is a mesocrystalline brick-and-mortar structure formed by C–S–H building units, with the surface adsorbed polymer being the ductile phase between the brittle C–S–H platelets. The drying of the swollen gel-like phase led to volume contraction, resulting in severe cracks. Hence, only mesocrystalline pieces in the order of a few hundred micrometers could be obtained, as depicted in Figure 87.

**Figure 88:**
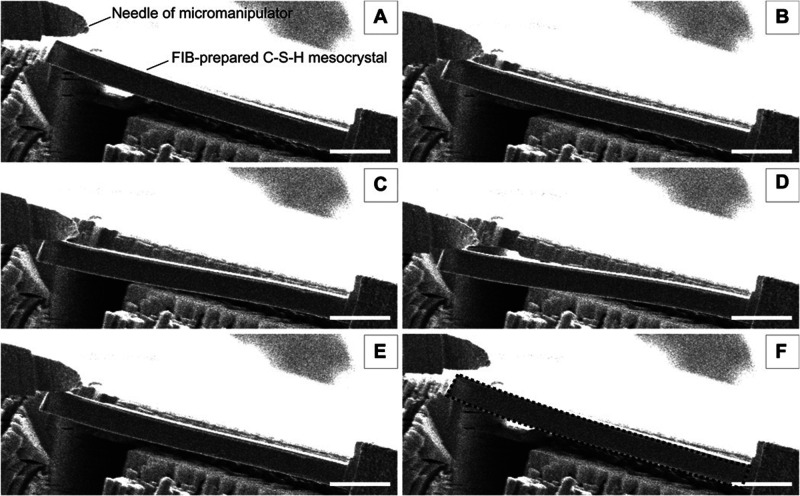
Visualization of the pronounced flexibility and elasticity of a C–S–H mesocrystal lever prepared by FIB (focused ion beam). A) to F) Picture series of the bending video (available as movie S1 of ref (13)) under the scanning electron microscope. The elastic deformation is revealed because the C–S–H mesocrystal cantilever fully relaxes after the application of mechanical stress by a micromanipulator (upper left corner). The dashed line in F) indicates the position of the C–S–H mesocrystal cantilever before bending. Scale bars, 10 μm. Reproduced from ref (13) under a Creative Commons Attribution Non-Commercial License 4.0 (CC BY-NC). Copyright 2017 American Association for the Advancement of Science. https://creativecommons.org/licenses/by-nc/4.0/.

Polarized optical microscopy reveals highly ordered regions indicated by the same color (Figure 87). SEM images confirm the expected layered structure (Figure 87). In addition, TEM revealed the high orientation of the C–S–H nanoplatelets in the mesocrystal. On the micrometer scale, no pores were identified. BET porosity measurements revealed a high inner surface area of the mesocrystals of 145 m^2^/g and a very small average porosity of only 3.9 nm. Consequently, good mechanical properties can be expected from the mesocrystalline C–S–H. Micromechanical tests were conducted using a micromanipulator on a C–S–H bar of defined dimensions to determine those. The bar was cut out of a mesocrystal particle by focused ion beam (FIB). Notably, the bar possessed significant flexibility and showed elastic deformation by returning to its initial position after the release of the force from the micromanipulator (Figure 88). This demonstrated the strong polymer binding to the C–S–H platelets and the absence of polymer gliding upon mesocrystal deformation.

The calculation of the flexural strength of mesocrystalline C–S–H revealed an exceptionally high value of up to 200 MPa.^13^ This figure is very close to the 210 MPa found for nacre and the 220 MPa found for bovine bone, natural archetypes for strong and tough materials.^620^ This outperforms the flexural strength of normal concrete by a factor of 40–100 and even the flexural strength of MDF cement, which typically ranges from 60–70 MPa,^113^ by a factor of 3. Moreover, mesocrystalline C–S–H surpasses the very high flexural strength of MDF cement (150 MPa) obtained by pressure and heat treatments.^565^ When considering the yield flexural strength of typical ASTM A36 building steel, which is around 250 MPa, and the ultimate flexural strength of 400–550 MPa, it is evident that the flexural strength of mesocrystalline C–S–H is close to the dimension of the yield flexural strength of steel.

**Figure 89:**
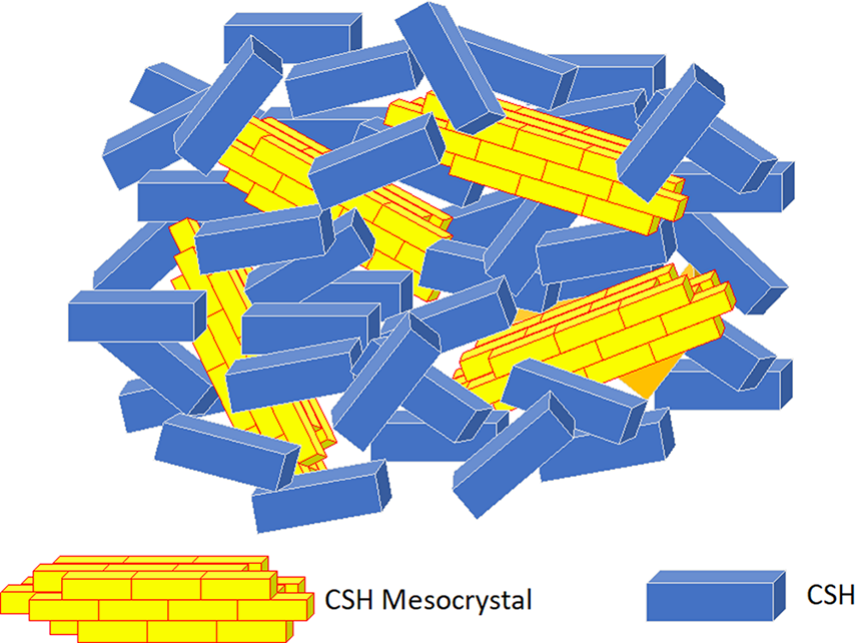
Mesocrystal microparticles as reinforcement in an unordered C–S–H structure.

Given these outstanding values, it is worth exploring further bottom-up approaches to obtaining ordered C–S–H structures with high strength, which will result in lower cement consumption to meet equivalent requirements. The C–S–H particles, similar in size to sea urchin spines^562^ or bones,^501^ fall within the nanometer scale, ensuring optimal strength and a high level of flaw tolerance.^621^ In addition, the arrangement of C–S–H subunits in a mesocrystalline structure allows for dense packing of platelets intercalated with polymers, thus optimizing the interactions between the individual building blocks. Although it is not yet possible to produce large-scale structures using a bottom-up self-assembly approach, mesocrystalline particles could serve as effective reinforcements or templates for controlling C–S–H formation in cement-based materials (Figure 89).

## 8 Conclusion and Outlook

To meet the ambitious global emission targets, cement materials inevitably need to be redesigned to be more efficient, thus reducing the amount of Portland cement used in the construction industry. Cement paste consists of a network of binding phases that play a major role in the setting and performance of cement-based materials. The properties of this network, including phase composition, crystallinity, thermodynamic stability, and nano- and microstructure, collectively affect the mechanical behavior and durability of the material. Therefore, understanding and controlling the formation of binding phases at the molecular and nanoscale levels, especially during the onset of precipitation (nucleation), is critical to the optimization of cementitious materials. In this regard, there is much to learn from nature, where the precise control of nucleation, mineralization/crystallization events, and hierarchical structuring over different length scales produces materials with exceptional mechanical or optical properties, as seen in natural structures such as bone, nacre, or glass sponges.

Over the past two decades, alternative theories have emerged to explain the formation of crystalline materials that go beyond the traditional textbook understanding of crystallization. These new crystallization models, referred to as **nonclassical crystallization** pathways, differ from classical theories that involve only atomic or molecular building blocks. The basis of these pathways is that the building blocks are larger, i.e., prenucleation solute species, liquid droplets, amorphous and crystalline nanoparticles, and often manifest as multistep processes that combine various of these. Applied to the crystallization of cementitious phases, the multistep character of nonclassical pathways offers new possibilities for synthetic control of the kinetics of binding phase formation and modulation of the properties of the resulting final phase. This control could be achieved by adjusting the conditions of the crystallization media or by introducing specific additives that interact with a particular stage.

According to recent research, **C–S–H** formation is likely to follow a nonclassical crystallization pathway. C–S–H prenucleation species and subsequent precursor intermediates, including liquid and amorphous phases, from which crystalline C–S–H emerges, have been experimentally identified. Isolation of individual C–S–H building blocks from the amorphous phase remains a challenge due to the rapid nature of the process, which may overlap with the aggregation-based mechanism of C–S–H building blocks. This ultimately leads to the observed C–S–H networks with two different packing densities. Although we are beginning to understand how C–S–H forms and its interactions with other species, there is still a long way to go to control the process of C–S–H formation and its nanostructure.

Due to the widespread use of SCMs, **C-A-S-H** has become the main phase of blended cements, with different characteristics compared to simple C–S–H. At the molecular level, fundamental research suggests that incorporating Al into the gel increases the aluminosilicate’s average chain length, forming more interconnected structures and increasing Si/Ca in the formed phase. These important changes are thought to be responsible for the changes in the morphology of the C-A-S-H phases, manifested in the observed transformations from fibrillar to sheet-like structures. Despite the recent progress in the characterization of C-A-S-H from experimental and computational approaches, there is a significant gap in understanding its early crystallization, the properties of the C-A-S-H primary building block, and the effect of additives. These areas have received less attention compared to C–S–H. Application of this knowledge could accelerate the formation of C-A-S-H in less reactive systems, thereby reducing their setting time and allowing higher clinker substitution levels.

The crystallization of **alternative binding phases** also deserves strong research attention as they represent a key strategy for CO_2_ reduction in the cement industry. The formation mechanism of the cementing phase in AA, CASC, and MOMS binders (e.g., N-A-S-H, CaCO_3_, M–S–H, MOC, MOS, and MP) has been shown to be a complex multistep process involving many of the precursors mentioned above. Similar to the C–S–H phase, these findings provide new strategies to control and optimize the formation of these binders. With respect to the identified prenucleation species in alternative binding phases, future research should investigate the characteristics, role, and evolution of the oligomeric species, especially with the goal of regulating the setting. A thorough investigation of the discovered amorphous phase is also essential, focusing on aspects such as composition, formation process, and stability. This is important for the selection of additives that interact with both ion associates and/or amorphous precursors, effectively inhibiting or promoting crystallization. In systems with multiple crystalline phases, stabilizing metastable phases such as nesquehonite (MCs) or the 5-phase in MOCs is beneficial because their transformation to more stable phases compromises the stability of the material. In contrast, in the MOS system, promoting the transformation of the metastable 3-phase to the most stable 5-phase is critical to achieve optimal strength development. Although effective in some cases, the use of additives for this purpose requires a deeper understanding of their mode of action during crystallization, which currently hinders the optimization of alternative binders through appropriate additive selection.

The use of **additives** to control the properties of cementitious materials is a common practice, but often in an empirical manner. Within the framework of nonclassical crystallization mechanisms, there are several additive-control mechanisms that are not accounted for in classical theories. Modifying the properties of the binding phase formed involves a dynamic interaction between additives and prenucleation species, liquid and amorphous intermediates, metastable phases, and nascent particles. In the case of C–S–H, organic additives can influence its formation from very early stages, and their demonstrated interaction with NCPs and precursor phases should be a key consideration in our understanding of the cement hydration process.

In the case of C–S–H, organic additives can influence its formation from very early stages. Therefore, the already established interaction of additives with PNCs and precursor phases should be considered to improve our understanding of the cement hydration process. This extension can be applied to almost all other binding phases discussed in this review (e.g., CH, C-A-S-H, N-A-S-H, CaCO_3_, HMCs, M–S–H, MOC, MOS, MPs), as most of them have been demonstrated to form via nonclassical crystallization mechanisms. The use of organic compounds has proven fundamental in stabilizing the primary C–S–H building blocks, which is the first step in achieving well-ordered C–S–H structures with improved properties. Extending this strategic approach to other binding phases promises to optimize their properties and performance.

Following a **bioinspired approach**, the mechanical properties of cementitious materials can be improved by directing the assembly of individual C–S–H nanoplatelets, resulting in well-ordered microstructures. This process involves the strategic intercalation of soft matter into the system, bridging the gap between the inorganic and brittle components. Inspired by the biomineralization process of sea urchin spines, a C–S–H mesocrystal characterized by aligned nanoplatelets of C–S–H interspersed with polymers has been developed, mimicking nature’s design for improved material performance. To achieve this, individual C–S–H nanoplatelets must first be stabilized, followed by controlled aggregation through pH adjustment or regulation of the C–S–H/polymer ratio. The resulting composite exhibits flexural strength comparable to nacre, highlighting the significant impact of combining organic additives and C–S–H platelets at the nanoscale. Although strategies for controlling the C–S–H microstructure are not currently applicable at the construction scale and have only been achieved at the microscopic level, a knowledge base is being built that may soon enable their implementation.

There is enormous pressure to accelerate the application of existing knowledge on the various topics covered in this review to bulk cementitious materials to reduce their carbon footprint. Therefore, close collaboration between experts from different disciplines and sectors should be pursued, as well as a multiscale approach that bridges different lengths and time scales to conduct more efficient research. From a bottom-up perspective, the possibilities are endless. However, as emphasized in this review, the success of such strategies remains questionable, relying solely on empirical methods in the absence of comprehensive knowledge of the formation of binding phases from ions in solution to the final material. In our view, the vision of manipulating cement at the nanoscale is closer than ever, thanks to deeper insights into the nucleation mechanisms of primary binding phases, self-assembly processes, bioinspired materials research, and the continued development of advanced experimental techniques that allow us to gain unprecedented insights into the formation of the binding phases.

## Notes

This paper is an additional review for Chem. Rev.2023, volume 123, issue (5), , “Sustainable Materials”.

## Dedication

There are no words to express my gratitude for Helmut’s unconditional support over the past years. Under his guidance, I had the opportunity to establish my own junior research group at the Department of Chemistry at the University of Konstanz, focusing on innovative multidisciplinary research that bridges nano-, micro-, and macro-scale knowledge with the goal of developing sustainable cementitious materials. Helmut’s continued support and confidence in me and my research, as well as his generosity, have allowed me to pursue my scientific goals. He had a special way of making people feel valued, sharing his enthusiasm for science, and encouraging us all to become better researchers. Working closely with him was an extraordinary experience that contributed significantly to my scientific growth. Beyond scientific mentorship, Helmut has taught me invaluable life lessons about strength, determination, optimism, commitment, and respect for others. I will carry these guiding principles with me throughout my life. When I think back to the many times I spent with him, what stands out is his friendly smile that comforted us, the members of his group, even in the most difficult moments of his illness. After a few months, his loss still weighs heavily on us because we have lost an outstanding researcher and mentor and, above all, an extraordinary, warm-hearted person and leader of our band.

## Abbreviations

A: Aluminum Oxide
AA: Alkali-Activated
ACC: Amorphous Calcium Carbonate
Afm: Monosulfoaluminoferrite Hydrate
AFM: Atomic Force Microscopy
Aft: Trisulfoaluminoferrite or Ettringite
BFS: Blast Furnace Slag
C: Calcium Oxide
C_2_(A,F): Calcium Aluminoferrite
C_2_S: Dicalcium Silicate
C_3_S: Tricalcium Silicate
Ca-ISE: Calcium Ion-Selective Electrode
C-A-S-H: Calcium Aluminosilicate Hydrate
CASC: Carbonatable Calcium Silicate Cements
CCUS: Carbon Capture, Utilization, and Storage
CH: Calcium Hydroxide
CM: Colloidal Model
CNT: Classical Nucleation Theory
cryo-TEM: Cryogenic Transmission Electron Microscopy
CS: Calcium Silicate (Wollastonite)
CSA: Calcium Sulfoaluminate Cements
C–S–H: Calcium Silicate Hydrate
CTAB: Cetyltrimethylammonium Bromide
DBM: Dense Branching Morphology
DFT: Density Functional Theory
EDX: Energy Dispersive X-ray
F: Iron Oxide
FA: Fly Ash
FCC: Face Centered Cubic
GBFS: Granulated Blast Furnace Slag
GCCA: Global Cement and Concrete Association
GHG: Greenhouse Gases
H: Water/Hydrate
HMCs: Hydrated Magnesium Carbonates
HD C–S–H: High-Density C–S–H
IEA: International Energy Agency
IGP: Intra-Globule Pores
K: Potassium oxide
LC^3^: Limestone Calcined Clay Cement
LD C–S–H: Low-Density C–S–H
LGP: Large Gel Pores
M: Magnesium Oxide
MAS NMR: Magic Angle Spinning-Nuclear Magnetic Resonance
MC: Magnesium Carbonate
MDF: Macro Defect Free
MOC: Magnesium Oxychloride Cements
MOMS: Magnesium Oxide Cements Derived from Magnesium Silicates
MOS: Magnesium Oxysulfate Cements
MP: Magnesium Phosphate
MS: Magnesium Silicate
N: Sodium Oxide
N-A-S-(H): Sodium Aluminosilicate Hydrate
NOx: Nitrogen Oxides
OPC: Ordinary Portland Cement
PAA: Poly(Acrylic Acid)
PAAm-*co*-PAA: Poly(Acrylamide-*co*-Acrylic Acid)
PC: Portland Cement
PCE: Polycarboxylate Ethers
PILP: Polymer Induced Liquid Precursor
PNC: Prenucleation Cluster
PolyDADMAC: Poly(Diallyldimethylammonium Chloride)
PVA: Poly(Vinyl Alcohol)
PVP: Poly(Vinylpyrrolidone)
PVP-*co*-PAA: Poly(1-Vinylpyrrolidone-*co*-Acrylic Acid)
RBPC: Reactive Belite-Rich Portland Clinkers
RCA: Recycled Concrete Aggregates
rhcp: Random Hexagonal Close-Packed
S: Silicon Dioxide
SAED: Selected Area Diffraction
SALs: Surface Altered Layers
SAXS: Small-Angle X-ray Scattering
SBKT: Symmetry-Based Kinematic Theory
SCMs: Supplementary Cementitious Materials
SEM: Scanning Electron Microscopy
SGP: Small Gel Pores
TEM: Transmission Electron Microscopy
TEOS: Tetraethoxysilane
THF: Tetrahydrofuran
$: Sulfate
